# Regioselective Multiboration and Hydroboration of Alkenes and Alkynes Enabled by a Platinum Single-Atom Catalyst

**DOI:** 10.1021/acscatal.5c03767

**Published:** 2025-10-03

**Authors:** Paweł Huninik, Priti Sharma, Vitthal B. Saptal, Martin Slaby, Rostislav Langer, Pawan Kumar, Ali Shayesteh Zeraati, Xiyang Wang, Martin Petr, Michal Otyepka, Manoj B. Gawande, Radek Zbořil, Stepan Kment, Jędrzej Walkowiak

**Affiliations:** a Center for Advanced Technologies, Adam Mickiewicz University, Uniwersytetu Poznańskiego 10, Poznań 61-614, Poland; b Faculty of Chemistry, 467899 Adam Mickiewicz University, Uniwersytetu Poznańskiego 8, Poznań 61-614, Poland; c Regional Centre of Advanced Technologies and Materials, Czech Advanced Technology and Research Institute (CATRIN), Palacký University Olomouc, Olomouc 779 00, Czech Republic; d Jerzy Haber Institute of Catalysis and Surface Chemistry, Polish Academy of Sciences, Niezapominajek 8, Krakow 30-239, Poland; e IT4Innovations, VSB-Technical University of Ostrava, 17. listopadu 2172/15, Ostrava-Poruba 708 00, Czech Republic; f Department of Mechanical & Industrial Engineering, University of Toronto, 5 King’s College Rd, Toronto, Ontario M5S 3G8, Canada; g Department of Mechanical and Mechatronics Engineering, Waterloo Institute for Nanotechnology, Materials Interface Foundry, 8430 University of Waterloo, Waterloo, Ontario N2L 3G1, Canada; h Nanotechnology Centre, Centre for Energy and Environmental Technologies, 48278 VSB−Technical University of Ostrava, Ostrava-Poruba 708 00, Czech Republic; i Department of Industrial and Engineering Chemistry, Institute of Chemical Technology, Mumbai-Marathwada Campus, Jalna-431213, Maharashtra, India

**Keywords:** heterogeneous catalysis, single-atom catalyst (SAC), diboration, triboration, hydroboration

## Abstract

Selective multiboration including di- and triboration and hydroboration of alkynes and alkenes face significant challenges in organic synthesis, including achieving high regioselectivity, functional group tolerance, and catalyst stability while requiring mild conditions to maintain reactivity. These transformations have been predominantly explored by using homogeneous catalysts. In this study, we report the scalable synthesis of heterogeneous platinum single-atom catalyst (Pt-SAC) supported on ultrathin nanosheets of graphitic carbon nitride via a rapid microwave-assisted method. The Pt-SAC enables 1,2-diboration of sterically hindered alkenes and 1,2,2-triboration of alkynes with B_2_pin_2_ under mild conditions. For the diboration of styrene, the catalyst achieves 99% yield with 95% selectivity, a turnover number (TON) of 3711, and a turnover frequency (TOF) of 247 h^–1^. The catalyst also promotes the regioselective hydroboration of alkenes and alkynes, yielding *anti*-Markovnikov alkylboranes and vinylboranes, respectively. Computational calculations reveal that the enhanced reactivity on the Pt-SAC catalyst arises from adsorption-induced weakening of key bonds (C=C and B–H), thereby significantly lowering the activation energy barriers. The Pt-SAC exhibits stability and recyclability, maintaining performance over at least eight consecutive runs without detectable Pt leaching. This study highlights the potential of Pt-SAC as a robust and versatile platform for organoboron transformations under mild conditions, with relevance to applications in pharmaceutical, agrochemical, and polymer synthesis.

## Graphical abstract

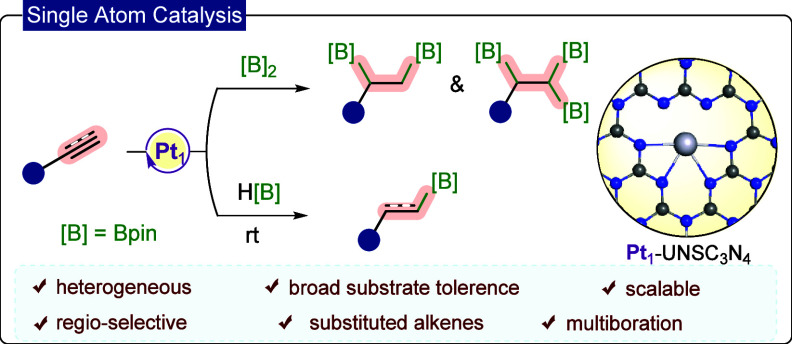

## Introduction

Organoboron compounds are a vital reagent in modern organic synthesis, serving as versatile carbon nucleophiles for the strategic installation of functional groups and forming diverse chemical bonds such as C–C, C–N, C–O, and C–S.^1–4^ Their remarkable versatility, ease of handling, and pivotal role in synthesizing natural products, pharmaceuticals, agrochemicals, and polymers establish them as highly valued intermediates.^5–8^ Among the diverse classes of organoborons, the selective synthesis of multiborated scaffolds, such as 1,2-diborated and 1,2,2-triborated compounds, which featuring multiple boronate moieties within a single molecular framework, stands as a fundamentally significant yet synthetically challenging objective^9,10^ These compounds are indispensable building blocks for the construction of complex molecules via precise, stereodefined cross-coupling reactions, facilitating the formation of multiple C–C bonds in a single synthetic operation.^11^ These molecules can be accessed through the borylation of premonoborylated intermediate or via the diboration of C–C multiple bonds.^12–15^

The pioneering discovery of platinum-catalyzed alkyne diboration by Suzuki and Miyaura in 1993 laid the foundation for significant advancements in diboration reactions, which paved the way for the extensive application of homogeneous transition-metal catalysts.^16,17^ These catalysts have since been employed to diborate alkynes, diynes, dienes, alkenes, and alkenes, yielding a wide range of bis­(boronate) compounds.^18–22^ While triboration is synthetically attractive due to its ability to generate tri­(boryl)­alkanes-versatile building blocks in organic synthesis, it remains significantly more challenging.^23^ Only a limited number of homogeneous catalytic systems have been reported, where issues such as reactivity, regioselectivity, use of equivalent amount of additives/bases, and chemo selectivity persist.^24–28^ However, while homogeneous catalysts for diboration are well-developed, progress in the development of heterogeneous catalysis remains crucially limited.^29,30^ This disparity arises from the challenges of achieving selectivity with heterogeneous systems, where multiple active sites can complicate control over reaction pathways.^31^ Heterogeneous diboration focuses primarily on alkynes, while there is only one report on alkene diboration using a heterogeneous nanocatalyst, specifically Pt/TiO_2_, which demonstrated a limited substrate scope.^32,33^ These challenges highlight the need for advancements in designing and optimizing heterogeneous systems for broader diboration applications.

In the field of metal-catalyzed hydroboration reactions, several significant advancements have been achieved recently. These include the development of copper-cluster catalysts for the hydroboration of alkynes, as well as cobalt­(II)-catalyzed asymmetric hydroboration of alkenes, and the dihydroboration of nitriles, delivering diverse hydroboration products in good yields.^34,35^ A copper-catalyzed enantioselective hydroboration method has been developed for constructing diverse chiral 1,2-benzazaborines, offering high yields and enantioselectivity for over >60 examples.^36^ Additionally, a cobalt-catalyzed system with a CNC pincer ligand allows the Z-selective hydroboration of terminal alkynes with high efficiency, scalability, and rare time-dependent stereoselectivity.^37^ However, challenges remain in the development of heterogeneous catalysts, including achieving high activity and selectivity for diverse and inactivated substrates, still rather elevated reaction temperature, reducing reliance or the amount on precious metals, and designing universal catalytic systems that tolerate complex molecular environments while maintaining efficiency and stability.

Recent advancements in SACs provide a promising solution to the above-mentioned limitations by serving as a bridge between homogeneous and heterogeneous catalysts. By exposing nearly 100% of metal atoms, SACs maximize the utilization of catalytic active sites, offering unique coordination environments as well as distinct geometric and electronic properties that enable efficient and highly selective catalysis.^38–40^ Furthermore, when firmly anchored to a suitable support, SACs exhibit thermal stability, ease of recovery, and significantly reduced precious metal usage, making them a sustainable choice for modern catalysis.^41–44^ Therefore, SACs have demonstrated remarkable performance in various applications, including organic synthesis, electrochemistry, energy, and photocatalysis.^45–47^ Notably, SACs have shown potential in diboration reactions. For example, Pt-SACs supported on nickel hydroxide nanoboards (Pt_1_/Ni­(OH)*x*) and polyoxometalate frameworks (Pt_1_-PMo@MIL-101) outperformed nanoparticle counterparts for alkyne diboration.^48^ Another example reported Pt-SACs supported on a composite material of reduced graphene oxide and Fe_2_O_3_ for alkyne diboration.^49^ Despite these advantages, reported SACs face challenges, such as undefined coordination environments, harsh reaction conditions, and limited substrate compatibility, restricting their application primarily to the reactive alkynes. These issues underscore the need for more efficient and selective heterogeneous SACs for diboration, particularly for challenging substrates such as substituted alkenes.

In this study, we report the first example of a Pt-based single-atom catalyst that enables highly regioselective 1,2-diboration of substituted alkenes, 1,2,2-triboration of alkynes, and monohydroboration of electronically and sterically challenging alkenes and alkynes. The catalyst, Pt_1_-UNSC_3_N_4_, features platinum single atoms uniformly anchored on ultrathin nanosheets of graphitic carbon nitride and was synthesized using a scalable microwave-assisted method. This SAC exhibits exceptional catalytic activity and selectivity under mild conditions, enabling efficient hydroboration and diboration of alkenes as well as hydroboration and triboration of alkynes. Pt_1_-UNSC_3_N_4_ demonstrates a broad substrate scope, high turnover, and excellent recyclability over eight consecutive cycles with negligible loss of performance. These features represent a significant advancement toward practical design catalysts for sustainable multiboration and hydroboration reactions using a robust and fully recoverable active catalyst of precious metals. Our approach to multiboration and hydroboration is summarized in Scheme 1 and benchmarked against previously reported, less efficient catalytic systems.

**Scheme 1:**
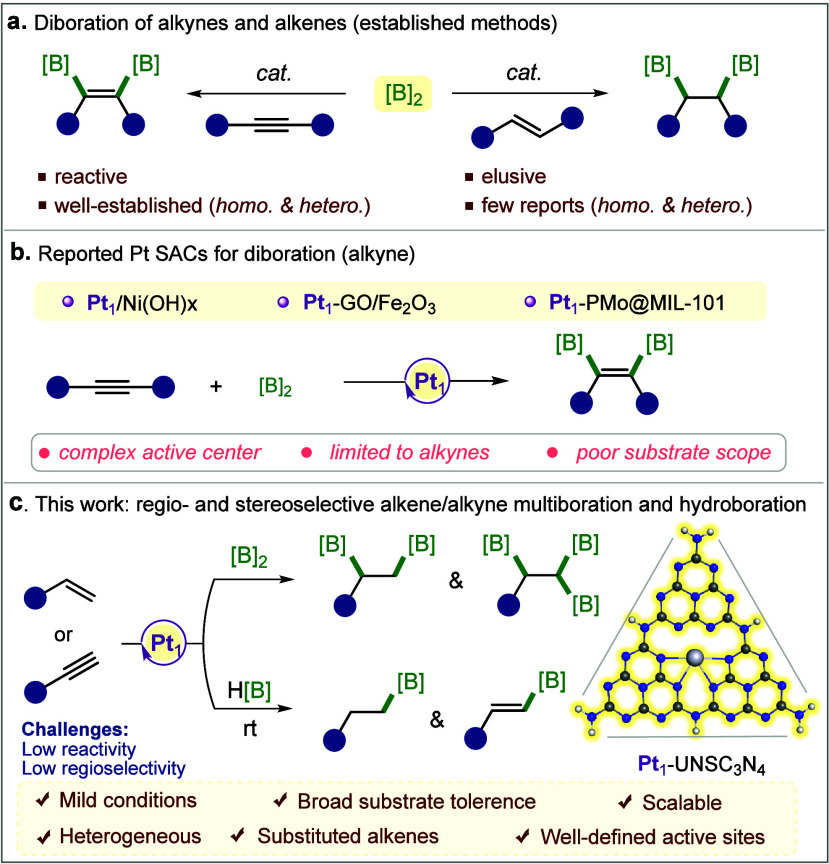
(a) Schematic Representation of Diboration of Alkynes and Alkenes, (b) Previously Utilized Pt-SACs for Alkyne Diboration, and (c) Our Innovative Approach for the Pt Single-Atom-Catalyzed Diboration of Alkene, Triboration of Alkyne, and Hydroboration of Alkene and Alkyne Functional Groups

## Results and Discussion

### Synthesis and Characterization of the Pt_1_-UNSC_3_N_4_ Catalyst

The preparation of the catalyst, featuring homogeneously distributed single Pt atoms embedded within 2D graphitic carbon nitride (g-C_3_N_4_) sheets, involved a sequential process illustrated in Figure 1a. This method specifically employs a three-step mild microwave treatment, during which the bulk g-C_3_N_4_ is exfoliated into C_3_N_4_ nanosheets and ultimately into ultrasmall nanosheets (ultrananosheets, UNSC_3_N_4_). In the final step, Pt salt (hexachloroplatinic acid) is added under sonication, resulting in the formation of a single-atom Pt catalyst assigned as Pt_1_-UNSC_3_N_4_. For comparison, a Pt catalyst deposited onto C_3_N_4_ in the form of Pt(0) nanoparticles was also prepared using a high Pt precursor loading; this sample is coded as Pt_*N*_-UNSC_3_N_4_ (detailed experimental procedures are provided in the Supporting Information, Section 2.1).

**Figure 1:**
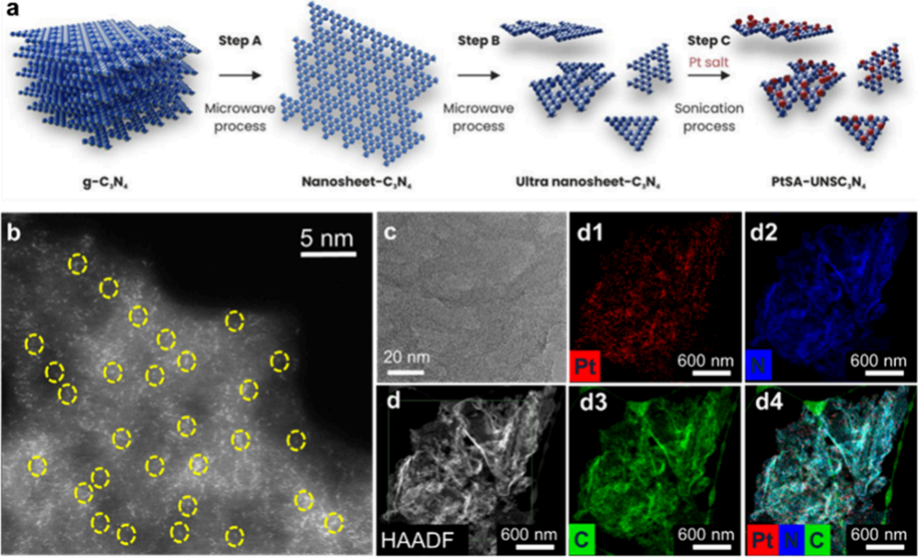
(a) Schematic illustration of the synthetic protocol for the platinum single-atom catalyst (Pt_1_-UNSC_3_N_4_) supported on ultrathin nanosheets of graphitic carbon nitride (C_3_N_4_). (b) STEM image (scale bar: 5 nm) highlighting atomically dispersed Pt single atoms on the C_3_N_4_ nanosheets (isolated atoms indicated by yellow circles). (c) TEM image (scale bar: 20 nm) showing the absence of Pt nanoparticles or aggregates. (d) HAADF-TEM image along with HAADF-STEM elemental mapping illustrating the spatial distribution of Pt (d1), N (d2), and C (d3), with the combined elemental map shown in panel (d4).

The scanning transmission electron microscopy (STEM) image in Figure 1b clearly demonstrates that the Pt_1_-UNSC_3_N_4_ samples exhibit a uniform and homogeneous distribution of Pt single atoms across the UNC_3_N_4_ support. The TEM image of the catalyst (Figure 2c) confirms the absence of Pt nanoparticles (NPs). The TEM comparison of Pt_1_-UNSC_3_N_4_ and Pt_*N*_-UNSC_3_N_4_ (Figure S1) further clearly illustrates the structural differences between the samples, specifically highlighting the presence of Pt in single-atom form versus NPs form. To further confirm the presence of Pt single atoms and the chemical composition of Pt_1_-UNSC_3_N_4_, aberration-corrected high-angle annular dark-field STEM (HAADF-STEM) measurements were performed (Figure 1d). These measurements demonstrate that all Pt species exist exclusively as isolated single atoms, with no evidence of NPs formation. Finally, energy-dispersive X-ray (EDX) elemental mapping (Figure 1d, d_1_–d_4_) provides the chemical maps for Pt, N, and C, confirming their uniform distribution across the catalyst.

**Figure 2:**
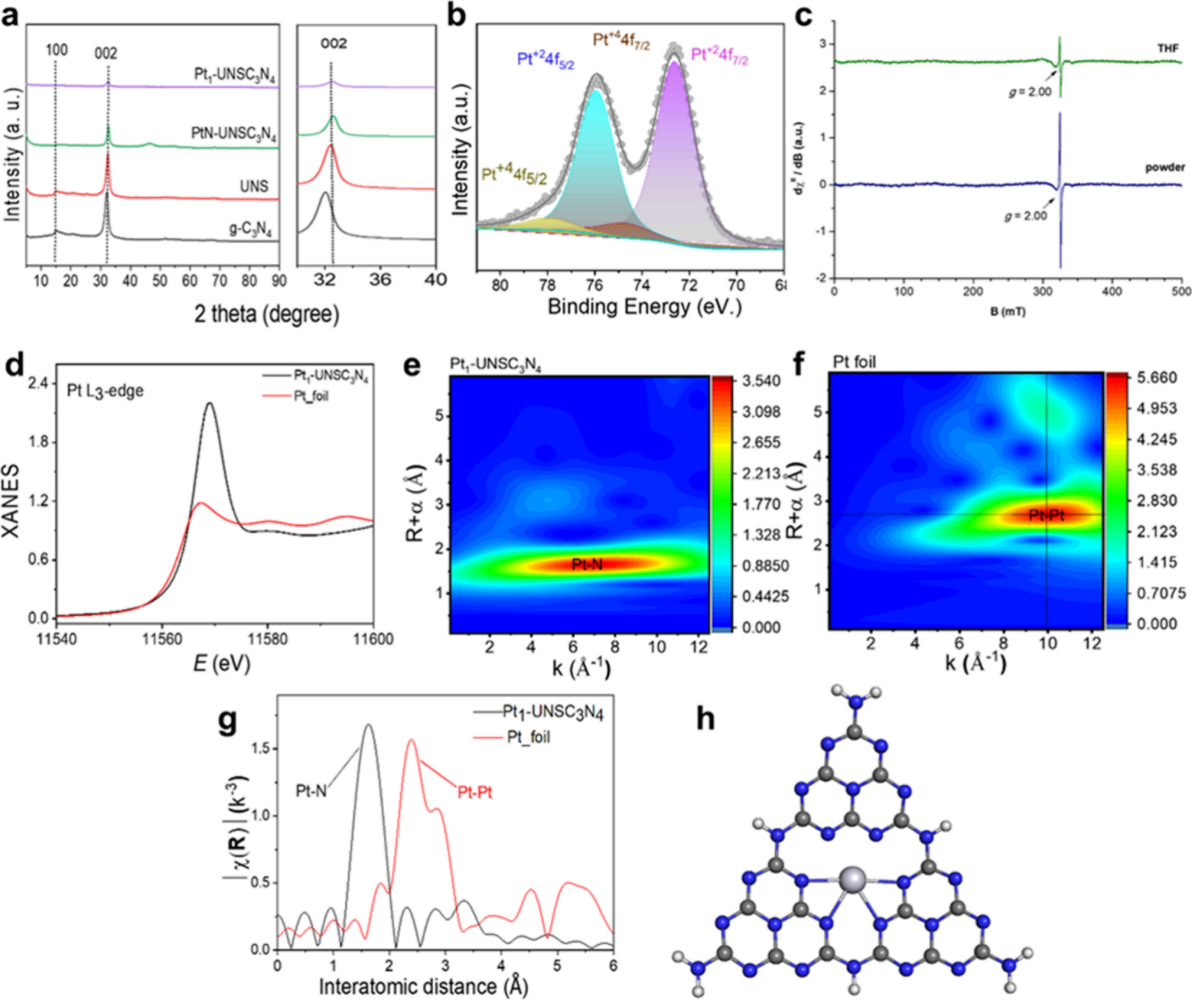
Characterizations of Pt_1_-UNSC_3_N_4_ and related materials. (a) Wide-angle XRD patterns with an inset showing the shift of the (002) peak to higher angles. (b) Pt 2p XPS spectrum of Pt_1_-UNSC_3_N_4_. (c) X-band EPR spectra of the Pt_1_-UNSC_3_N_4_ recorded in a powder form and upon dispersion in dry THF. Experimental acquisition parameters: ∼9.08–9.09 GHz, 2.00 mW applied microwave power, 0.03 s time constant, 1.0 mT modulation width, 8 min acquisition time, *T* = 90 K, structure model Pt_1_-UNSC_3_N_4_ single-atom catalyst. (d) XANES spectrum of Pt_1_-UNSC_3_N_4_ and Pt foil. (e, f) WT EXAFS maps of Pt_1_-UNSC_3_N_4_ and Pt foil. (g) FT-EXAFS spectrum of Pt_1_-UNSC_3_N_4_ and Pt foil. (h) Structural model of Pt_1_-UNSC_3_N_4_ as derived from experimental data.

The X-ray diffraction (XRD) patterns were recorded to analyze the bulk composition, phase purity, and possible reflections associated with Pt species in the synthesized samples, including bulk C_3_N_4_, nanosheets of UNSC_3_N_4_, Pt_1_-UNSC_3_N_4_, and Pt_*N*_-UNSC_3_N_4_ (Figure 2a). Two distinct diffraction peaks at 2θ values of 15.15 and 32.00° correspond to the (100) and (002) planes of graphite-like carbon nitride in a planar-packed system (JCPDS 87-1526).^50^ The sharp diffraction peak at 32.00° signifies the interplanar stacking of aromatic structures (π–π stacked sheets), while the weaker peak at 15.15° corresponds to interplanar structural packing involving the trigonal nitrogen linkage of the tris-triazine motif.^42,50^ Compared to g-C_3_N_4_, the (002) diffraction peak shifts slightly to higher 2θ values in Pt_1_-UNSC_3_N_4_ (see the inset in Figure 2a), suggesting an increased interplanar distance due to the incorporation of Pt into the UNSC_3_N_4_ scaffold during the microwave-assisted process.^51^ Furthermore, the (100) peak at 15.15° nearly disappears in Pt_1_-UNSC_3_N_4_, indicating a loss of long-range ordering due to the formation of 2D ultrasmall sheets.^52^ Notably, no additional peaks corresponding to metallic or oxidized Pt species are observed in Pt_1_-UNSC_3_N_4_, indicating a uniform dispersion of Pt single atoms. While Pt_*N*_-UNSC_3_N_4_ exhibits a small reflection at 46.12°, which is attributed to metallic Pt nanoparticles. The X-ray photoelectron spectroscopy (XPS) of the Pt 2p region (Figure 2b) for Pt_1_-UNSC_3_N_4_ showed two distinct peaks at 72.58 and 75.90 eV, corresponding to Pt^2+^ 4f_7/2_ and Pt^2+^ 4f_5/2_, respectively, confirming the dominant presence of single atoms Pt^2+^ species. The intense satellite peaks suggest that Pt^2+^ is coordinated with nitrogen in the carbon nitride structure.^53,54^ In addition to Pt^2+^, minor contribution of Pt^4+^ species is evident in the Pt 2p XPS spectrum, as demonstrated via peaks at 74.80 and 78.20 eV. Notably, the absence of the Pt(0) oxidation state in XPS analysis aligns with findings from microscopic analyses XRD, EXAFS, and wavelet transform (WT) EXAFS spectra (Figure 2a–g). Detailed XPS analysis of the samples is further provided in Figure S2 and Tables S1–S4. The X-band electron paramagnetic resonance (EPR) spectrum of the catalyst, recorded in powder form at *T* = 90 K, is shown in Figure 2c (lower spectrum) and displays a strong and isotropic resonance signal at *g* = 2.00. This signal remains unchanged upon dispersion of the powder in dry tetrahydrofuran (THF) (Figure 2c, upper trace). The EPR signal in THF is, however, lower in intensity due to diamagnetic dilution; the signal is consistent with an *S* = 1/2 system centered on the carbon support. No other signals ascribable to paramagnetic Pt centers were detected; therefore, the Pt-SA catalyst, besides spin defects contained in the support, includes metal centers adopting the closed-shell as expected for Pt­(II) or Pt­(IV) configurations, in line with XPS results.

To investigate the oxidation state, electronic charge distribution, and coordination environment of the Pt single atoms, X-ray absorption near-edge structure (XANES) spectra of Pt_1_-UNSC_3_N_4_ were recorded in Pt L_3_-edge (Figure 2d). The XANES spectra originate from unoccupied Pt 5d states. A significantly high white line intensity for Pt_1_-UNSC_3_N_4_ compared to Pt foil (Pt^0^) demonstrates a reduced electronic concentration on 5d orbitals. These observations corroborate that the presence of electron deficient Pt sites originates from metal to ligand charge transfer (MLCT) in the Pt–N-bonded state. The absence of any pre-edge feature and significantly intense white line intensity also suggests the presence of Pt in the (II) oxidation state.

The presence of single-atomic Pt sites was further confirmed by Fourier-transform extended X-ray absorption fine structure (EXAFS) spectroscopy (Figure 2g). The FT-EXAFS spectrum of Pt_1_-UNSC_3_N_4_ displayed a sharp peak at ∼1.6 Å attributed to Pt–N first-shell scattering suggesting Pt atoms coordinated to secondary nitrogens (C_2_N:) in C_6_N_7_ (tri-*s*-triazine) composed cavity of CN sheets. Notably, no second-shell peak corresponding to Pt–Pt (2.39 Å) scattering or Pt–O–Pt was detected deciphering the absence of any Pt/PtO_2_ NPs and validating that the Pt site exists as isolated atoms.^55^ EXAFS fitting of Pt_1_-UNSC_3_N_4_ spectra revealed Pt–N coordination number (CN) of 4.77 (±0.02) with a uniform bond length of 2.14 Å suggest Pt–N_4_ coordination (see Table S5). The slightly elevated coordination number might have arisen due to weak coordination of electron deficient Pt sites with oxygen/H_2_O low electron density 5d orbitals. Wavelet transform (WT) EXAFS analysis of Pt_1_-UNSC_3_N_4_ (Figure 2e) exhibits a single sharp scattering zone centered at *K* = 6.86 A^–1^ and *R* = 1.66 A, attributed to Pt–N coordination. The absence of any Pt–Pt scattering (observed at *K* = 9.97 Å^–1^ and *R* = 2.67 Å as observed for Pt foil) further confirms that Pt exists as single-atom species (Figure 2f).^56,57^ Based on these findings, a structural model of Pt_1_-UNSC_3_N_4_ is proposed in Figure 2h, depicting Pt single atoms in a tetracoordinated configuration within the carbon nitride matrix.

### Catalytic Performance of Pt_1_-UNSC_3_N_4_ for Diboration Reactions

To evaluate the catalytic activity of the Pt_1_-UNSC_3_N_4_ catalyst, we initially tested its performance in the diboration of styrene (**1**) using bis­(pinacolato)­diboron (B_2_pin_2_) as the borylating reagent (Figure 3). The optimization process began with the use of 10 mg of Pt_1_-UNSC_3_N_4_ (5.0 × 10^–4^ mol % Pt) at 100 °C, a reaction time of 15 h, and toluene as the solvent (Figure 3a and Table S6). Under these conditions, styrene conversion reached 20% (**2**), with an exceptional selectivity of 99% for the diboration product and 1% for the olefin reduction product (**4**). Switching to the THF solvent led to lower yields and poor selectivity for product **2**. We then tested binary solvent mixtures of toluene and polar solvents (ethanol, water, or methanol) in a 3:1 ratio. The toluene–ethanol mixture provided improved 50% conversion with a selectivity of 57% for diboration and 43% for the monoborylation product (**3**). In contrast, the toluene–water mixture yielded only 12% conversion, albeit with 99% selectivity for the diboration product. Notably, using a toluene–methanol mixture resulted in over 99% conversion, with a selectivity ratio of 86:14:0 for diboration, monoborylation, and olefin reduction, respectively. Importantly, when methanol was used as the sole solvent at 70 °C, we achieved 99% conversion with 99% selectivity for the diboration product. Even at a reduced temperature of 50 °C, conversion remained high (95%), with a selectivity of 99% (Figure 3b, also see Table S7). Further lowering the temperature resulted in decreased conversions.

**Figure 3:**
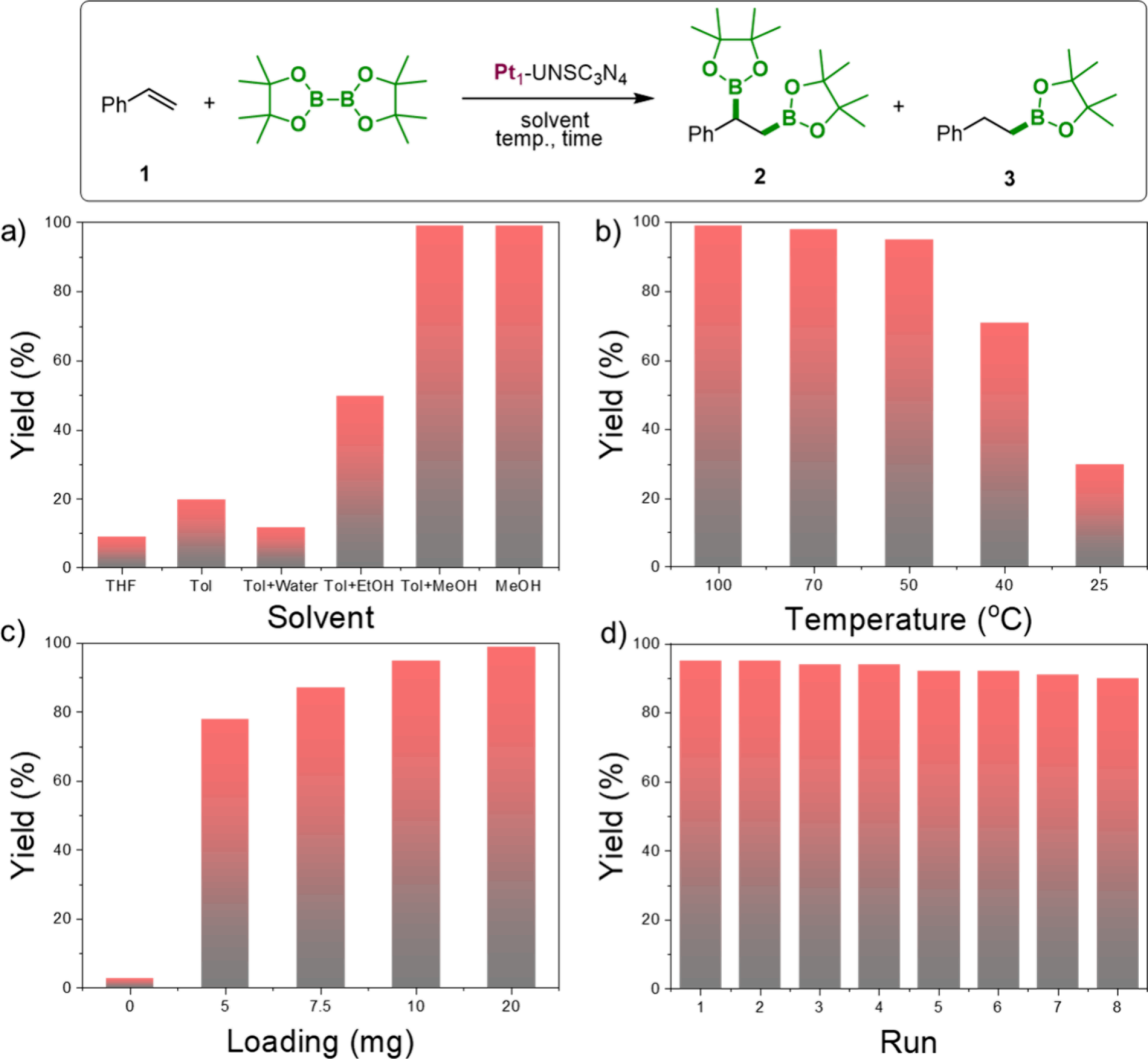
Reaction scheme for the diboration of styrene with B_2_pin_2_ catalyzed by Pt_1_-UNSC_3_N_4_. Optimization of reaction conditions: (a) effect of solvent, (b) effect of temperature, (c) catalyst loading effect, and (d) recyclability of Pt_1_-UNSC_3_N_4_. Reaction conditions: all diboration reactions were performed in a dram vial under an air atmosphere, styrene (1.0 mmol), B_2_pin_2_ (1.0 equiv), solvent (0.25 mL), and Pt_1_-UNSC_3_N_4_ as a catalyst. Yields were determined by ^1^H NMR analyses using mesitylene as the internal standard.

We also investigated the effect of the catalyst loading. Increasing the Pt_1_-UNSC_3_N_4_ catalyst amount to 20 mg resulted in excellent conversion and selectivity within 10 h (Figure 3c and Table S8), whereas reducing the catalyst loading to 5 mg led to a 78% conversion. Catalyst efficiency was further evaluated by calculating the turnover number (TON) and turnover frequency (TOF). Using 10 mg of catalyst (5.0 ppm Pt) led to a 95% yield, with a TON of 3711 and a TOF of 247 h^–1^. Reducing the catalyst loading to 5 mg (2.5 ppm of Pt) resulted in a 78% yield, with a significantly higher TON of 7143 and a TOF of 476 h^–1^, highlighting the catalyst’s efficiency even at lower loadings. No conversion was observed in the absence of Pt_1_-UNSC_3_N_4_, showcasing the essential role of the catalyst (Table S8, entry 1). The effect of B_2_pin_2_ loading was also examined. Increasing its amount to 1.2 equiv resulted in excellent conversion, whereas reducing it to 0.5 equiv led to lower yield and selectivity (Table S9). Interestingly, no diboration occurred when alternative diboron reagent bis­(catecholato)­diboron (B_2_cat_2_) was used. However, bis­(neopentyl glycolato) diboron (B_2_neop_2_) gave a 40% diboration yield (Table S10). We evaluated the recyclability of Pt_1_-UNSC_3_N_4_ for the diboration of styrene (Figure 3d). The catalyst demonstrated excellent recyclability, maintaining its activity for up to eight cycles with a negligible loss in performance. Furthermore, inductively coupled plasma mass spectrometry (ICP-MS) analysis confirmed that no detectable platinum leaching occurred during the recycling process, underscoring the robustness of the heterogeneous catalyst system. Finally, we compared catalytic activity of Pt nanoparticles supported on Pt_*N*_-UNSC_3_N_4_ where lower yields noted and no significant product formation was observed when employed a pure UNSC_3_N_4_ (Table S11). These observations underscore the importance of the presence of single-atom Pt species for activity and sustainability. The easy recovery and reuse of the heterogeneous catalyst not only reduce precious metal waste and contamination but also simplify product isolation, representing key practical advantages over homogeneous systems. These features collectively demonstrate the potential of our catalyst for scalable, sustainable applications in selective borylation reactions.

After optimizing the reaction conditions, we extensively explored the potential of Pt_1_-UNSC_3_N_4_ across various alkenes under an air atmosphere (Scheme 2). Styrene achieved 95% conversion with an isolated yield of 88% (**2a**). Additionally, aryl alkenes bearing electron-donating functional groups, such as 4-*tert*-butylstyrene and 4-methoxystyrene, exhibited high tolerance, resulted in 94% (**2b**) and 86% (**2c**) conversions, respectively. Similarly, halide-substituted styrenes, such as *para*-bromostyrene and *ortho*-chlorostyrene, gave conversions of 90 and 84% respectively (**2d, 2e**). Internal alkenes such as *trans*-β-methylstyrene (**1f**) and *trans*-stilbene (**1g**) exhibited high reactivity, achieving conversions of 87 and 85%, respectively. The cyclic aromatic alkene like 1*H*-indene (**1h**) also showed good conversion at 84%. Additionally, the aliphatic cyclic olefin like cyclohexene (**1i**) underwent efficient diboration and afforded the corresponding product (**2i**) with a high conversion of 90%. Importantly, the Pt_1_-UNSC_3_N_4_ catalyst exhibited impressive conversions reaching up to 91% even for challenging 1,1-disubstituted alkenes such as α-methylstyrene (**1j**), which contains both steric phenyl and methyl groups around the olefinic bond. Its derivatives including 4-Me and 4-F also displayed good conversions (**1k**, **1l**), showcasing the catalyst’s robustness and versatility with 1,1-disubstituted aromatic alkenes. Notably, the naturally available allyl functional group such as eugenol (**1m**), which comprises a hydroxyl functional group, achieved an excellent conversion of 97%. In the case of 4-vinylaniline (**1n**), the presence of a free amine significantly suppressed the reactivity, resulting in only 35% conversion. In contrast, the protected amine derivative allylisoindoline-1,3-dione, despite its steric bulk and electronic complexity, achieved a conversion of 69% (**2o**). However, a styrene derivative bearing a cyano group (**1p**) was proven to be unreactive under the standard reaction conditions, with no detectable formation of the diborylated product (**2p**). The catalyst efficiently facilitated the diboration of pinacol vinylboronate (**1q**), affording product **2q** in a high isolated yield of 86%. For aliphatic alkenes such as allylbenzene (**1r**) and 1-octene (**1s**), conversions were similarly high, achieving 96% (**2r**) and 94% (**2s**), respectively. Importantly, the catalyst demonstrated chemoselectivity: in the case of allylacetone, only the C=C bond was transformed, affording the diboration product in 85% isolated yield (**2t**).

**Scheme 2:**
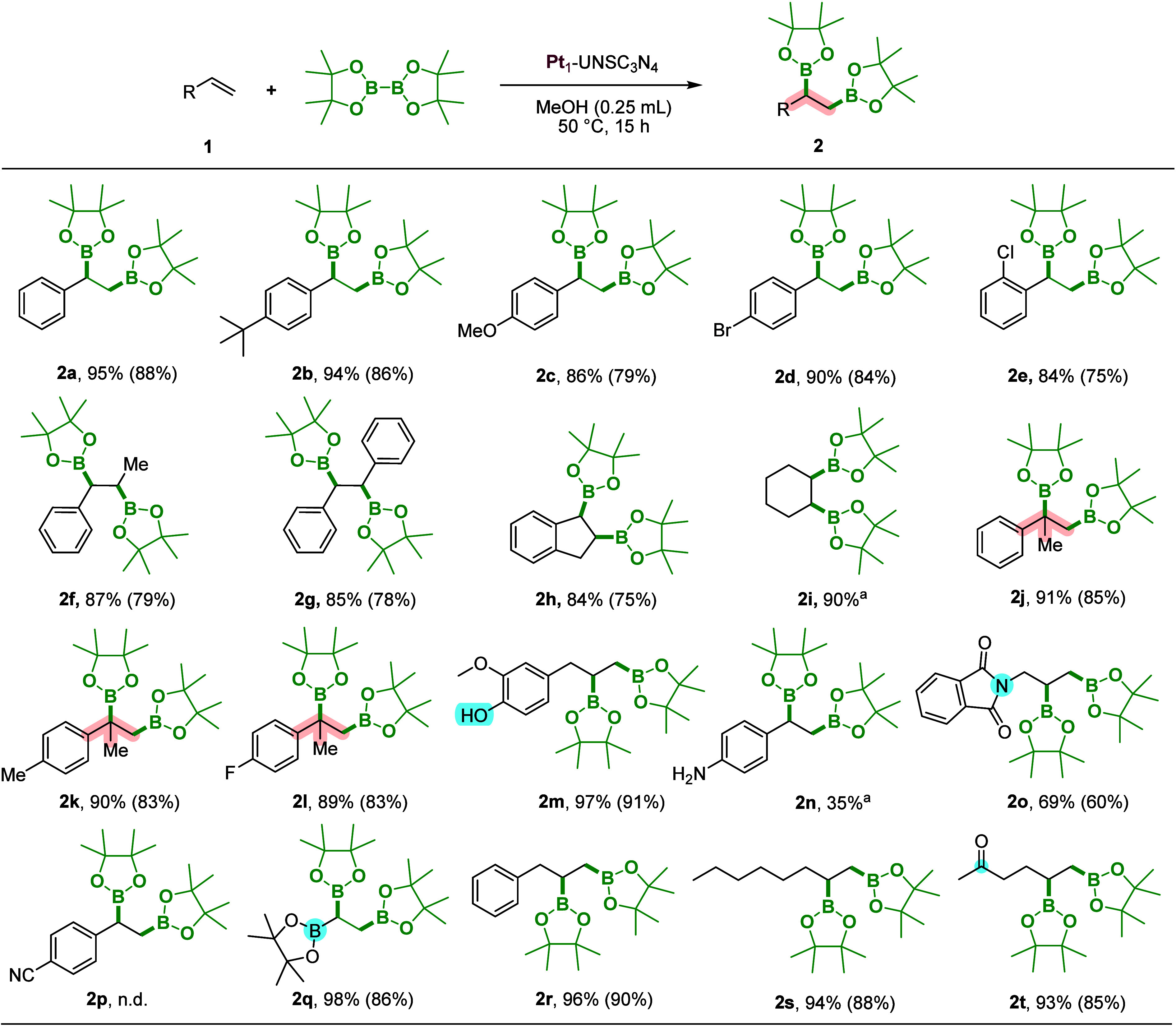
Substrate Scope for the Diboration of Olefins^a^

a Isolated yields are presented in parentheses. All diboration reactions were performed in dram vials under an air atmosphere in 50 °C for 15 h, using a reaction mixture containing the particular olefin (1.0 mmol), B_2_pin_2_ (1.0 equiv), MeOH (0.25 mL), and Pt_1_-UNSC_3_N_4_ as a catalyst (5.0 × 10^–4^ mol % Pt, 10 mg). Conversion was determined by ^1^H NMR analysis using mesitylene as an internal standard. ^a^The Product was not isolated, and yield determined by ^1^H NMR analysis using mesitylene as an internal standard is presented.

### Catalytic Performance of Pt_1_-UNSC_3_N_4_ for Triboration Reactions

After establishing an efficient Pt_1_-UNSC_3_N_4_-catalyzed protocol for the diboration of alkenes, we next extended our investigation to the triboration of alkynes to evaluate the broader utility of the described methodology. To the best of our knowledge, there are no prior reports on the triboration of alkynes employing a heterogeneous catalyst. This work therefore represents the first example of such a transformation, highlighting a new avenue for metal-catalyzed C–B bond formation under heterogeneous conditions. The reaction was carried out using a 1.5-fold excess of the diboron reagent relative to the alkyne under the same conditions previously optimized for the diboration of alkenes (Scheme 3). Building on the broad substrate scope already established for alkene diboration, we investigated a targeted set of six alkyne substrates for triboration. Terminal aromatic alkynes, such as phenylacetylene, yielded the desired triborylated product in 81%. Substituents on the aromatic ring were well tolerated, with *para*-OMe, *ortho*-Me, and *para*-Br phenylacetylenes furnishing products **7b** in 73%, **7c** in 71%, and **7d** in 79% yields. The aliphatic terminal alkyne 1-octyne (**6e**) gave the highest yield at 88%. However, internal alkynes such as diphenylacetylene (**6f**) and 4-octyne (**6g**) did not afford the triborylated products under the same reaction conditions, underscoring a current limitation of the methodology.

**Scheme 3:**
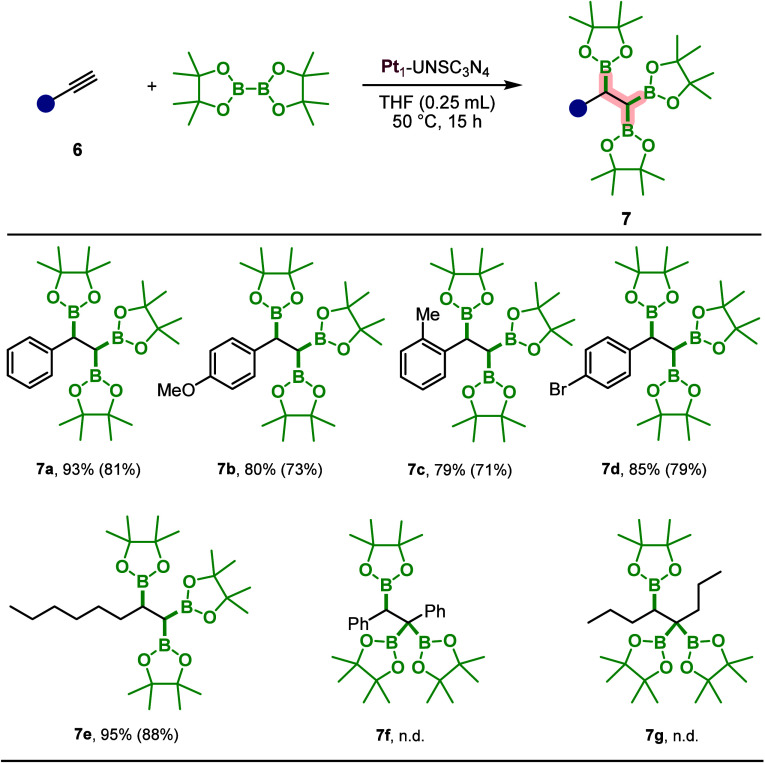
Substrate Scope for the Triboration of Alkynes^a^

a Isolated yields are presented in parentheses. All triboration reactions were performed in dram vials under an air atmosphere in 50 °C for 15 h, using a reaction mixture containing the particular olefin (1.0 mmol), B_2_pin_2_ (1.5 equiv), MeOH (0.25 mL), and Pt_1_-UNSC_3_N_4_ as a catalyst (5.0 × 10^–4^ mol % Pt, 10 mg). Conversion was determined by ^1^H NMR analysis using mesitylene as an internal standard.

### Catalytic Application of Pt_1_-UNSC_3_N_4_ for Hydroboration

Given the high catalytic activity of Pt_1_-UNSC_3_N_4_ for alkene and alkyne di- and triboration, we envisioned synthesizing monoborylated products via a hydroboration reaction. We expanded the application of Pt_1_-UNSC_3_N_4_ to facilitate the hydroboration of alkenes, producing monoborylated alkylboranes using pinacolborane (HBpin) as the hydroboration reagent (Scheme 4). This method offers a powerful strategy for the selective synthesis of monosubstituted alkylboranes. While a wide range of homogeneous catalysts, including both noble and first-row transition metals, have been explored for alkene hydroboration, achieving high yields and selectivity under mild conditions with heterogeneous catalysts remains a significant challenge. We conducted a meticulous optimization of reaction conditions for the hydroboration of styrene (**1**, 1.0 mmol) with HBpin (1.0 mmol) in the presence of Pt_1_-UNSC_3_N_4_ s (Tables S12–S15). When we utilized 10 mg of Pt_1_-UNSC_3_N_4_ in THF as the solvent at 80 °C, after 15 h, we achieved a total conversion of 99% with a selectivity breakdown of *anti*-Markovnikov = 95% (**3**), Markovnikov = 1% (**5**), and ethylbenzene = 4% (**4**) (Table S12). Interestingly, employing a nonpolar solvent like toluene resulted in a lower yield and with a slight shift in selectivity toward ethylbenzene (yield = 62%, *anti*-Markovnikov = 92%, Markovnikov = 2%, ethylbenzene = 6%). On the other hand, the use of polar protic solvents like MeOH failed to initiate the hydroboration reaction (Table S12, entry 5). When we reduced the reaction temperature from 80 to 25 °C, while keeping THF as the solvent, we achieved a 98% yield of the hydroborated product with an exceptional selectivity of 99% toward the *anti*-Markovnikov product. We obtained the best optimization results performing the reaction in 18 h at 25 °C with 98% conversion and selectivity of 99% (Table S13, entry 4). We also investigated the effect of HBpin loading on the hydroboration reaction of alkene and observed that the use of 1.5 equiv of HBpin resulted in the highest conversion in 15 h of reaction time (Table S14, entry 1). Increasing the catalyst loading to 20 mg resulted in a substantial improvement in yield, reaching 99%, and a notable increase in *anti*-Markovnikov (**3**) selectivity to 99% (Table S15). Additionally, increased catalyst loading (20 mg) reduced the reaction time to 10 h (Table S15, entry 2). However, when the catalyst loading was reduced from 7 to 5 mg (2.5 ppm of Pt), a gradual decrease in conversion was observed, while selectivity remained consistent. This suggests that the reaction still proceeds efficiently even with a parts per million level of catalyst loading (Table S15, entries 4 and 5).

**Scheme 4:**
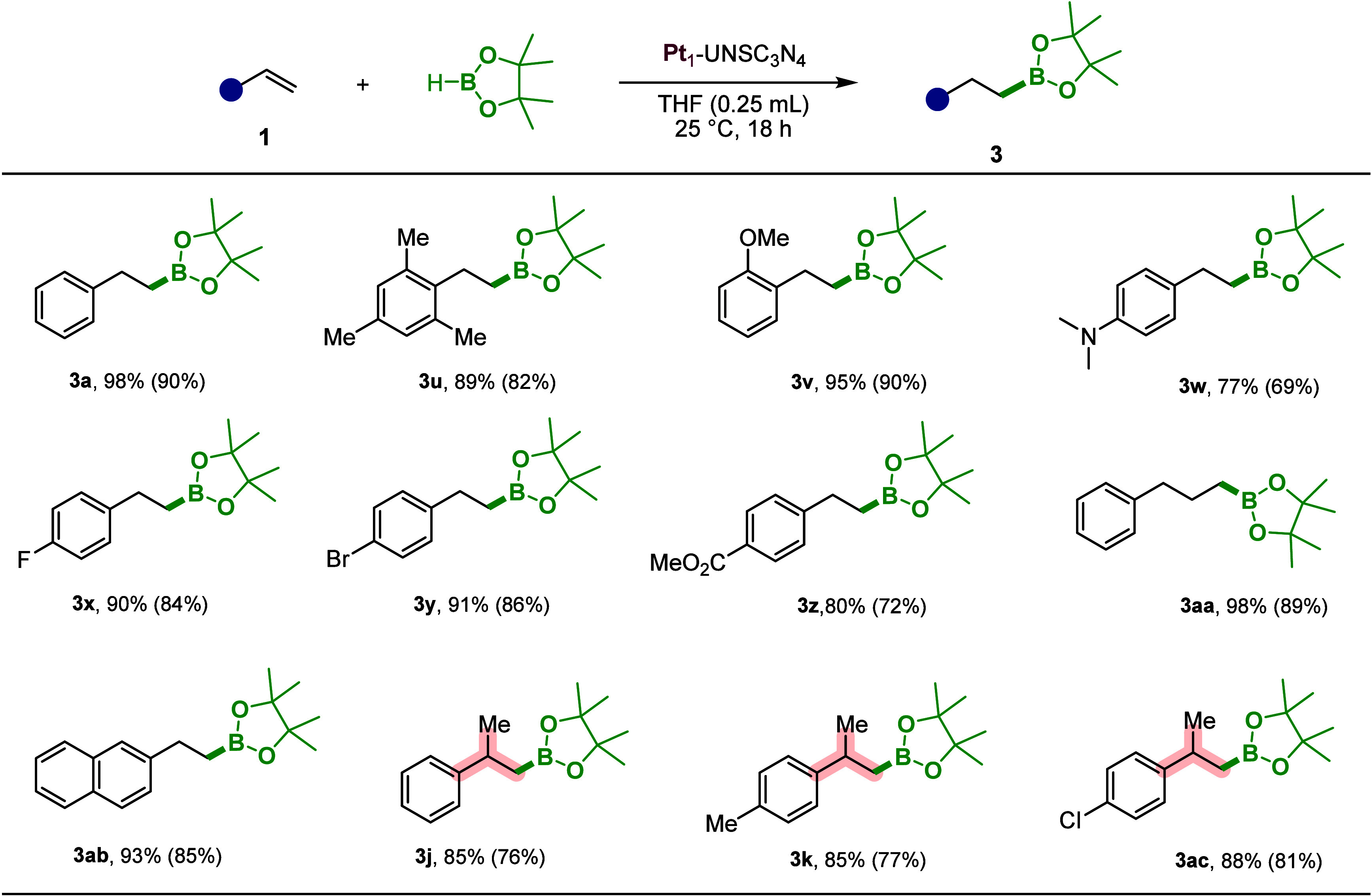
Substrate Scope for the Hydroboration of Alkenes^a^

a Isolated yields are presented in parentheses. Reactions were carried out at optimized conditions: alkene (1.0 mmol), HBpin (1.0 equiv), THF (0.25 mL), Pt_1_-UNSC_3_N_4_ (5.0 × 10^–4^ mol % Pt, 10 mg), 18 h reaction time, temp. (25 °C), argon atmosphere. Conversion was determined by ^1^H NMR analysis using mesitylene as an internal standard.

Following the optimization of reaction conditions (1.0 mmol of alkene, 1.0 equiv of HBpin, 0.25 mL of THF, temperature of 25 °C, and 10 mg of Pt_1_-UNSC_3_N_4_ catalyst in an argon atmosphere), we expanded the scope of our study to explore a variety of styrenes for hydroboration reaction (Scheme 4). Simple styrene (**1a**) reached 98% conversion and 90% isolation yield (**3a**). We then examined styrene with electron-donating groups, such as bulky 1,3,5-trimethyl and 2-methoxy styrene, both of them provided excellent yields (**3u**, **3v**), 82 and 90%, respectively. Next, *N*,*N*-dimethyl-4-vinylaniline was tested leading to 77% conversion (**3w**). We proceeded to investigate substrates with *para*-substituted halides, including fluoro and bromo (**3x**, **3y**), which exhibited excellent isolated yields, reaching up to 84 and 86%, respectively. Furthermore, a substrate with an electron-withdrawing group, such as methyl 4-vinylbenzoate was found to tolerate the reaction with a 72% yield (**3z**). When we tested allylbenzene as a substrate, selective hydroboration occurred at the beta position with an impressive 89% yield (**3aa**). Alkene substituted with a naphthalene group was efficiently hydroborated, resulting in an 85% yield (**3ab**). Remarkably, even sterically hindered alkenes bearing a methyl substituent, such as α-methylstyrene (**3j**) and its *para*-substituted derivatives with methyl (**3k**) and chloro (**3ac**) groups, were well tolerated, affording isolated yields ranging from 77 to 81%.

Furthermore, we applied the Pt_1_-UNSC_3_N_4_ catalyst to the selective hydroboration of alkynes, successfully synthesizing vinylboranes using the same reaction conditions as those for alkenes (Scheme 5). These vinylboranes serve as essential building blocks in organic synthesis. Using phenylacetylene, we achieved 95% conversion (**8a**). We also screened substituted aryl alkynes with electron-donating and -withdrawing groups such as −OMe, −CN, and −3,5-bis­(trifluoromethyl), obtaining excellent yields for the respective vinylboranes (**8h**–**8j**). Additionally, the catalyst produced a 3-thiophene vinylborane with 90% conversion, demonstrating its efficacy in hydroborating heterocyclic compounds (**8k**). The catalyst showed outstanding performance not only for terminal alkynes but also for internal alkynes. For instance, the hydroboration of diphenylacetylene gave an impressive 91% conversion (**8f**). In the case of 3-phenyl-1-propyne (**6l**), product **8l** was obtained in 83% isolated yield as a mixture of α- and β-isomers. Moreover, aliphatic and silyl-substituted alkynes also tolerated the reaction conditions, delivering excellent yields of the desired products (**8e**, **8m**).

**Scheme 5:**
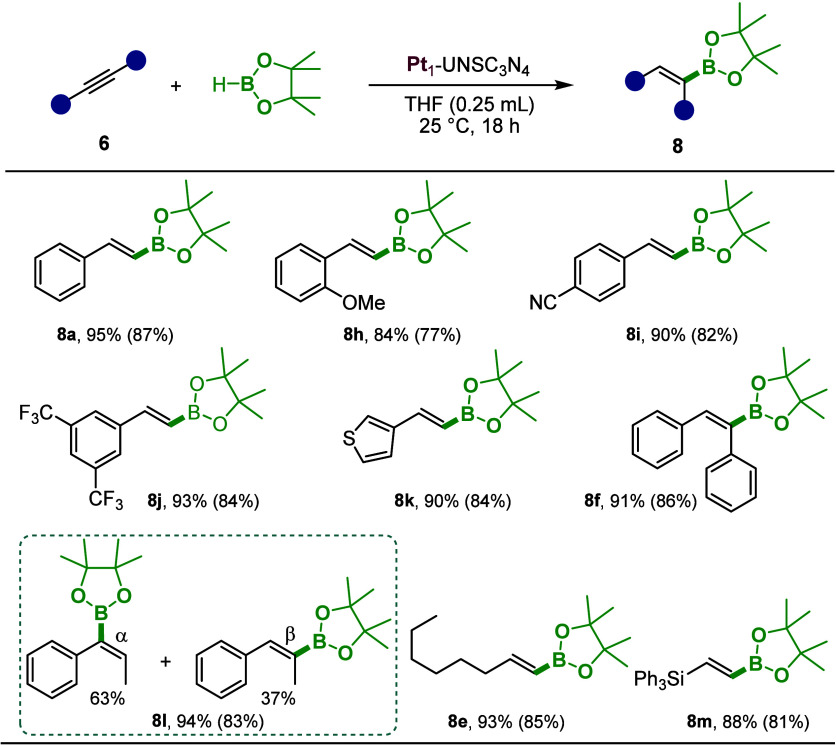
Substrate Scope for the Hydroboration of Alkynes^a^

a Isolated yields are presented in parentheses. Reaction conditions: alkyne (1.0 mmol), HBpin (1.0 equiv), THF (0.25 mL), Pt_1_-UNSC_3_N_4_ (5.0 × 10^–4^ mol % Pt, 10 mg), 18 h reaction time, temp. (25 °C), argon atmosphere. Conversion and selectivity were determined by ^1^H NMR analysis using mesitylene as an internal standard.

### Mechanistic Investigations

To gain mechanistic insights into the catalyzed reaction, we performed density functional theory (DFT) calculations to compute the Gibbs free energy profiles along the reaction pathways. We compared reaction profiles of the Pt_1_-UNSC_3_N_4_-catalyzed diboration and hydroboration in methanol with the uncatalyzed reference reaction, which displayed high reaction barriers reaching up to 73 kcal/mol (Figure S4).

The catalytic cycle begins with the adsorption of reactants onto the Pt_1_-UNSC_3_N_4_ active site (Figure 4). Specifically, we investigated the adsorption of styrene, B_2_pin_2_, and HBpin onto Pt_1_-UNSC_3_N_4_ to determine, which reactant interacts most strongly with the catalyst, identifying the preferred adsorbate. Styrene has shown the strongest adsorption of −24.3 kcal/mol, followed by B_2_pin_2_ (−22.8 kcal/mol) and HBpin (−6.3 kcal/mol), indicating that styrene preferentially binds to the catalytic center. This preferential adsorption of styrene activates the double bond, as evidenced by the Wiberg bond index (WBI) analysis, which shows a decrease in the C_α_–C_β_ bond order from 1.92 in the free molecule to 1.46 when adsorbed. This activation is facilitated by π-electron donation to Pt­(II) orbitals, rendering the styrene double bond more susceptible to diboration and hydroboration reactions. For the sake of completeness, it should be noted that for the boron-containing reagents, the B–B bond order in adsorbed B_2_pin_2_ remained nearly constant (0.95 vs 0.96), while the B–H bond order in adsorbed HBpin decreased from 0.96 to 0.68, weakening is attributed to the interaction between the hydrogen atom and the Pt ion, as supported by a Pt–H WBI of 0.23 and an increase in the B–H bond length from 1.20 to 1.33 Å.

**Figure 4:**
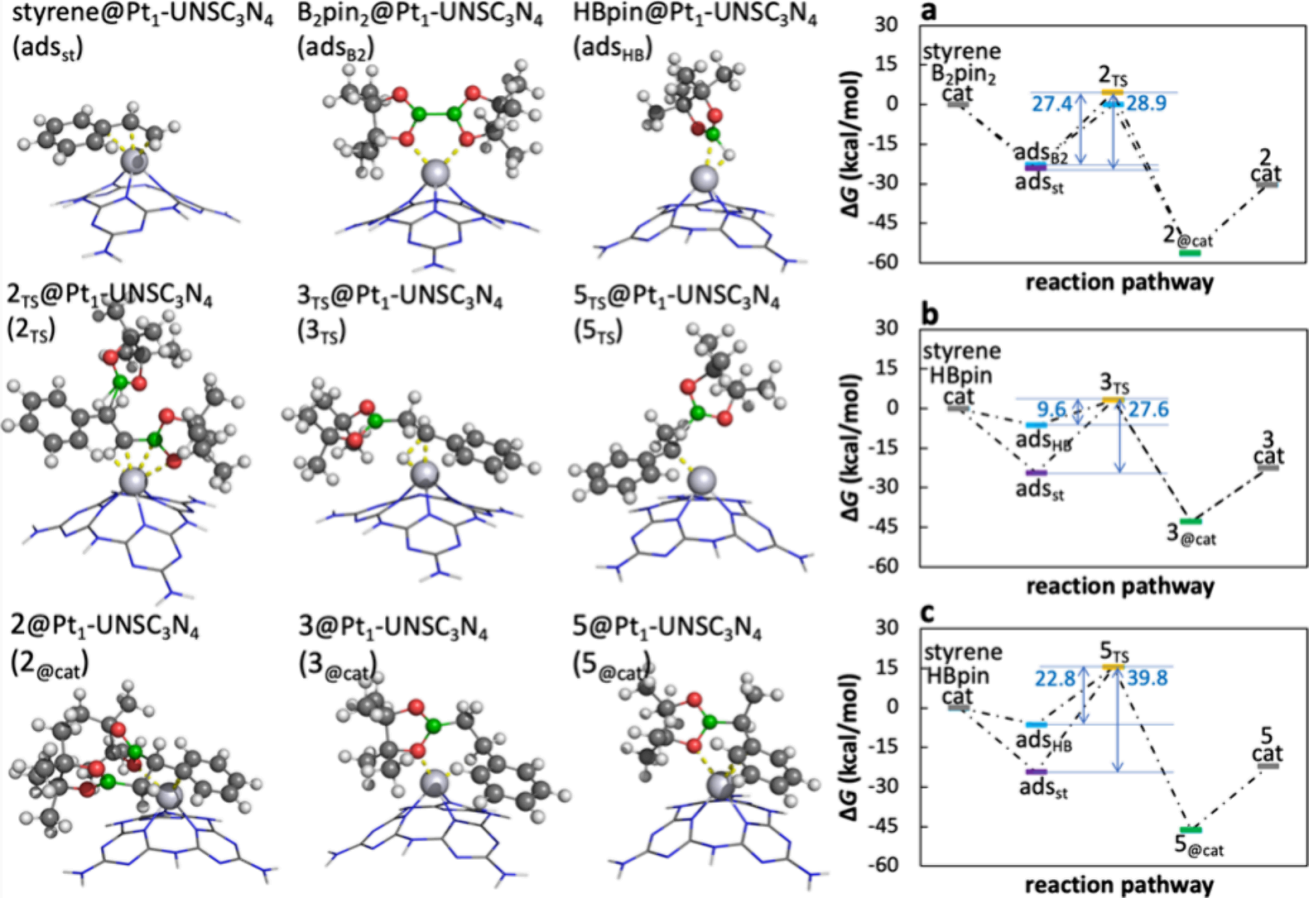
Molecular structures and corresponding energy profiles of the diboration and hydroboration of styrene catalyzed by Pt_1_-UNSC_3_N_4_. Left panels show the optimized geometries of reactants, products, and TSs adsorbed at the catalyst. The labeling of the structures is consistent with the labeling of the synthesized products. Platinum shown as a light gray ball, carbon atoms in dark gray, hydrogen in white, boron in green, and oxygen in red. The right panels show associated reaction profiles with calculated standard Gibbs energies (323 K, 1 atm) in methanol. The reaction involves adsorption of the reactant to the catalyst, chemical transformation to the product via the TS, and product desorption, i.e., the catalyst regeneration. (a) Initial adsorption of either styrene or B_2_pin_2_ onto Pt_1_-UNSC_3_N_4_ (denoted as cat) followed by the reaction with the other reactant (either styrene or B_2_pin_2_) toward product 2 via TS. (b) Initial adsorption of either styrene or HBpin onto Pt_1_-UNSC_3_N_4_ followed by the reaction with the other reactant (either styrene or HBpin) toward product 3 via TS. (c) Initial adsorption of either styrene or HBpin onto Pt_1_-UNSC_3_N_4_ followed by the reaction with the other reactant (either styrene or HBpin) toward product 5 via TS.

Since all reactants demonstrated negative adsorption energies on the catalyst, two possible mechanistic pathways were considered; (i) the interaction of adsorbed styrene on Pt_1_-UNSC_3_N_4_ with B_2_pin_2_ or HBpin, and (ii) the interaction of either adsorbed B_2_pin_2_ or adsorbed HBpin on Pt_1_-UNSC_3_N_4_ with styrene. Adsorption energy evaluations suggest that the first scenario is more favorable. The energy barriers for the Pt_1_-UNSC_3_N_4_ catalyzed reactions ranged from 10 to 40 kcal/mol (Figure 4), being significantly reduced compared to the reference reactions in the absence of the catalyst (Figure S4). The decrease in reaction barriers is attributed to the weakening of the C_α_–C_β_ double bond of styrene and the B–H bond in HBpin due to their interactions with the catalyst (*vide supra*). The desorption energies required for product release and catalyst regeneration ranged from 20 to 26 kcal/mol (Figure 4).

In summary, although styrene exhibited stronger adsorption on the catalyst, energy barriers were lowered when HBpin was adsorbed onto Pt_1_-UNSC_3_N_4_ first, followed by the interaction with styrene. Therefore, we propose a mechanism in which Pt­(II) weakens both the double bond in styrene and the H–B bond in HBpin, thereby facilitating the reaction. The proposed reaction schemes, illustrating possible reaction mechanisms, are presented in Scheme 6 for the diboration reaction and for hydroboration in Scheme S1.

**Scheme 6:**
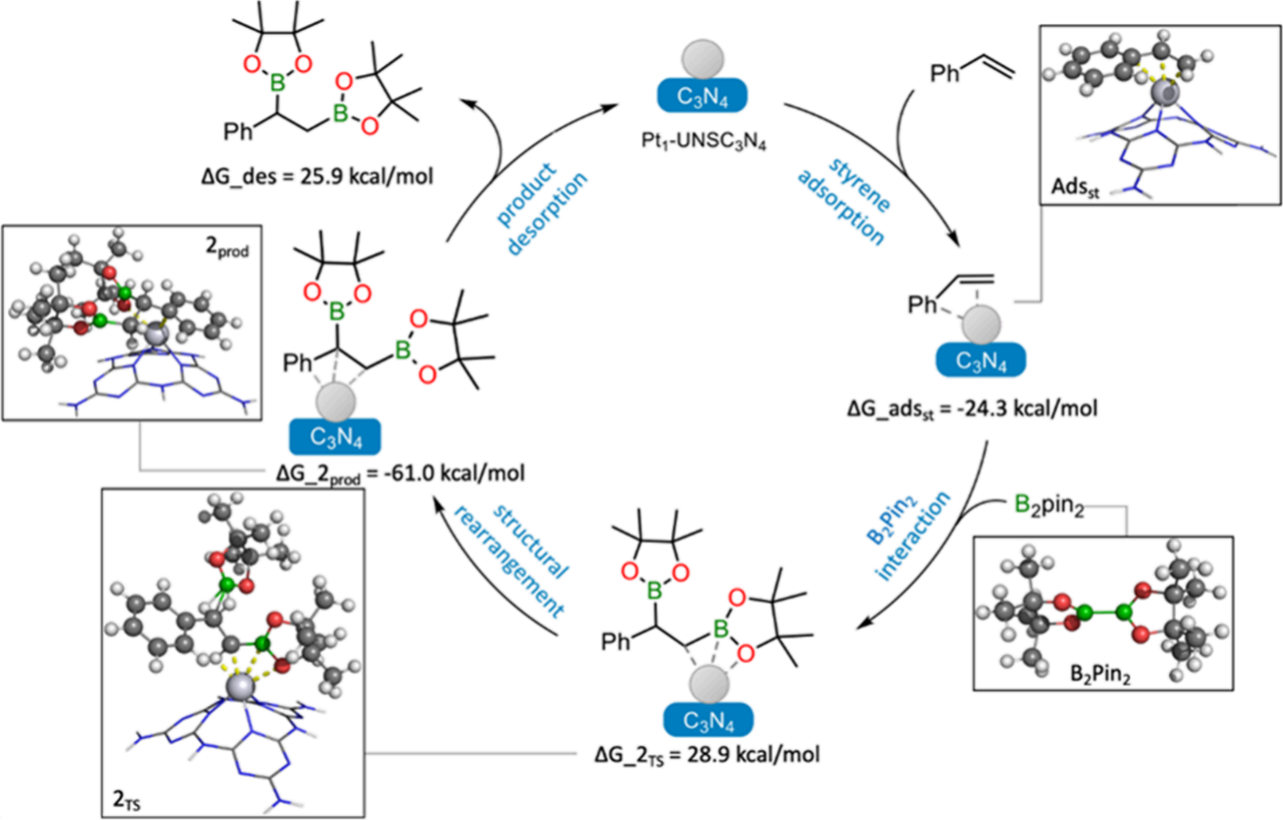
Possible Reaction Mechanism for the Diboration of Styrene Catalyzed by Pt_1_-UNSC_3_N_4_, Based on DFT Calculations

### Applications of the Pt_1_-UNSC_3_N_4_ Catalyst

To demonstrate the utility of the Pt_1_-UNSC_3_N_4_-catalyzed diboration of alkenes, we conducted a gram-scale reaction using the challenging alkene α-methylstyrene and B_2_pin_2_ (Scheme 7a). The desired product **2j** was obtained in an 81% yield. Furthermore, this methodology proved to be highly effective for the synthesis of challenging substituted diols, delivering product **2ja** in an excellent yield of 92% (Scheme 7b). Additionally, the protocol was successfully extended to other transformations, such as the one-pot hydroboration of alkynes and a tandem Suzuki-Miyaura coupling reaction (Scheme 7c), both of which provided excellent yields for the corresponding C–C coupled product (**6ia**).

**Scheme 7:**
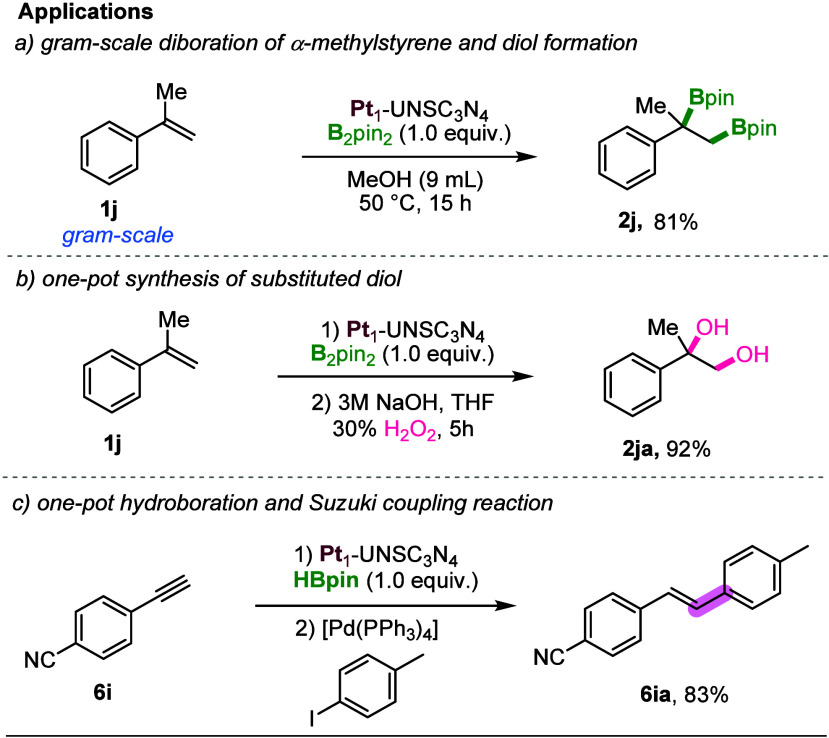
Applications of the Pt_1_-UNSC_3_N_4_ Catalyst for the (a) Large-Scale Applications of Developed Protocol to Synthesize Challenging Diboron; (b) One-Pot Synthesis of Substituted Diols; and (c) One-Pot Hydroboration and Suzuki Coupling Reaction, **6i** (1.0 mmol), HBpin (1.0 equiv), Pd­(PPh_3_)_4_ (5 mol %), 4-Iodotoluene (1.2 equiv), THF (1 M), 3 M Cs_2_CO_3_, 70 °C, 24 h, Argon Atmosphere; Isolated Yields Are Presented

## Conclusions

This study introduces the first heterogeneous platinum single-atom catalyst capable of achieving the highly regioselective multiboration and hydroboration of alkenes and alkynes under exceptionally mild conditions. The catalyst enables 1,2-diboration of alkenes, 1,2,2-triboration of alkynes, and selective anti-Markovnikov hydroboration reactions across a wide range of substituted and sterically challenging substrates, tolerating diverse functional groups and highlighting its broad applicability and potential for sustainable synthesis. The Pt-SAC, supported on ultrathin nanosheets of C_3_N_4_, is scalable, stable, and recyclable-operating effectively even at extremely low platinum loadings. Notably, it retains exceptional efficiency even at very low catalyst loading, without the use of base and any other activator or additive. Using just 10 mg (5.0 ppm Pt), a 95% yield was achieved with a TON of 3711 and TOF of 247 h^–1^. When the catalyst loading was further reduced to 5 mg (2.5 ppm of Pt), the reaction still proceeded efficiently, achieving a 78% yield with an impressive TON of 7143 and TOF 476 h^–1^. These results highlight the catalyst’s sustainability, efficiency, and scalability for potential industrial applications. Theoretical insights revealed that the enhanced reactivity of Pt-SAC arises from adsorption-induced weakening of key bonds (C=C and B–H), significantly reducing reaction barriers. Additionally, the catalyst exhibits excellent recyclability, maintaining its single-atom character and activity over eight cycles without any Pt leaching or loss of efficiency. The protocols developed in this work are compatible with large-scale applications, as demonstrated by gram-scale production and the synthesis of substituted diols and other complex molecules. Furthermore, this heterogeneous catalyst uniquely enables the selective 1,2,2-triborylation of alkynes, a transformation not previously achieved using any other heterogeneous system. Overall, these findings underscore the potential of Pt-SACs to advance organoboron chemistry, with immediate implications for pharmaceutical, polymer, and agrochemical industries. These results position Pt-SAC as a promising platform for sustainable and highly efficient catalytic transformations.

## Methods

A sequential synthesis approach was employed to prepare the g-C_3_N_4_, UNSC_3_N_4_, Pt_1_-UNSC_3_N_4_, and Pt_*N*_-UNSC_3_N_4_ catalysts. Bulk g-C_3_N_4_ was synthesized via thermal polymerization of dicyandiamide, followed by exfoliation into nanosheets (nC_3_N_4_) through controlled heating. Further sonication of nC_3_N_4_ led to the formation of ultrananosheets (UNSC_3_N_4_), which served as a photoactive support for platinum dispersion. The Pt_1_-UNSC_3_N_4_ single-atom catalyst was obtained by the dropwise addition of hexachloroplatinic acid to UNSC_3_N_4_, followed by sonication, reduction with NaBH_4_, and microwave treatment. A nanoparticle-based Pt catalyst (Pt*_N_*-UNSC_3_N_4_) was synthesized by using incipient wetness impregnation. A complete list of reagents can be found in the Supporting Information, Section 1, which also details the characterization techniques (XRD, XPS, XANES, EXAFS, TEM, ICP-MS. NMR, GCMS. HRMS. FT-IR, etc.) and instruments used. A step-by-step description of the synthetic procedure is provided in the Supporting Information, Section 2.1.

## Supplementary Material



## Notes

Additional experimental details, optimization of reaction conditions, catalyst characterization data, NMR spectra and DFT calculation details, materials, methods, compound characterization (PDF)
