# Supplementary material for: Regioselective Multiboration and Hydroboration of Alkenes and Alkynes Enabled by a Platinum Single-Atom Catalyst
Source: ACS Catal. 2025 Oct 3;15(20):17347–60. doi: 10.1021/acscatal.5c03767 (PMC12538553; doi:10.1021/acscatal.5c03767)
Supplement: Supplementary file 1 [file cs5c03767_si_001.pdf]

Supporting Information for

Regioselective Multiboration and Hydroboration of Alkenes and Alkynes Enabled by Platinum Single Atom Catalyst

Paweł Huninik,[‡a,b] Priti Sharma,[‡c,d] Vitthal B. Saptal,[*a] Martin Slaby,[c] Rostislav Langer,[e] Pawan Kumar,[f] Ali Shayesteh Zeraati,[f] Xiyang Wang,[g] Martin Petr,[c] Michal Otyepka,[c,e] Manoj B. Gawande,[h] Radek Zbořil,[*c,h] Stepan Kment,[*c,h] Jędrzej Walkowiak[*a]

[a]*Center for Advanced Technologies, Adam Mickiewicz University, Uniwersytetu Poznańskiego 10, 61-614 Poznań, Poland.*

[b]*Faculty of Chemistry, Adam Mickiewicz University, Uniwersytetu Poznańskiego 8, 61-614 Poznań, Poland.*

[c]*Regional Centre of Advanced Technologies and Materials, Czech Advanced Technology and Research Institute (CATRIN), Palacký University Olomouc, 779 00 Olomouc, Czech Republic.*

[d]*Jerzy Haber Institute of Catalysis and Surface Chemistry, Polish Academy of Sciences, Niezapominajek 8, 30-239 Krakow, Poland.*

[e]*IT4Innovations, VSB-Technical University of Ostrava, 17. listopadu 2172/15, 708 00 Ostrava-Poruba, Czech Republic.*

[f]*Department of Mechanical & Industrial Engineering, University of Toronto, 5 King's College Rd, Toronto, M5S 3G8 Ontario, Canada.*

[g]*Department of Mechanical and Mechatronics Engineering, Waterloo Institute for Nanotechnology, Materials Interface Foundry, University of Waterloo, Waterloo, N2L 3G1 Ontario, Canada.*

[h]*Nanotechnology Centre, Centre for Energy and Environmental Technologies, VSB–Technical University of Ostrava, 708 00 Ostrava-Poruba, Czech Republic.*

*E-mail: vitthalsaptal87@gmail.com, radek.zboril@upol.cz, stepan.kment@upol.cz, jedrzej.walkowiak@amu.edu.pl

Table of Contents

1. Methods ... 4

1.1. Reagents and Materials ... 4

1.2. Physico-chemical characterization ... 4

1.2.1. X-ray diffraction (XRD) ... 4

1.2.2. Transmission electron microscopy (TEM) ... 5

1.2.3. X-ray photoelectron spectroscopy (XPS) ... 5

1.2.4. X-ray absorption near edge structure (XANES) and Extended X-ray absorption fine structure (EXAFS) ... 5

1.2.5. Operando X-ray absorption spectroscopy (XAS) ... 6

1.2.6. Inductively Coupled Plasma-Mass spectrometry (ICP-MS) ... 6

1.2.7. NMR analysis ... 6

1.2.8. GC-MS analysis ... 6

1.2.9. High resolution mass spectrometry (HRMS) ... 7

1.2.10. FT-IR analysis ... 7

1.2.11. Elemental analysis ... 7

2. Preparation of materials ... 7

2.1. Synthesis of $UNSC_3N_4$ and Platinum single atom Pt_1-$UNSC_3N_4$ over ultra-nanosheet $UNSC_3N_4$ ... 7

3. Materials characterization ... 8

3.1. TEM analysis ... 8

3.2. XPS analysis ... 9

3.3. XAS analysis ... 11

4. Catalytic part ... 12

4.1. Optimization of reaction conditions ... 12

4.1.1. Diboration of alkenes catalyzed by Pt_1-$UNSC_3N_4$ ... 12

4.1.2. Hydroboration of alkenes catalyzed by Pt_1-$UNSC_3N_4$ ... 15

4.1.3. Diboration of alkenes catalysed by Pt_1-$UNSC_3N_4$ under optimized reaction conditions 16

4.1.4. Hydroboration of alkenes catalyzed by Pt_1-$UNSC_3N_4$ under optimized reaction conditions ... 17

4.1.5. Hydroboration of alkynes catalyzed by Pt_1-$UNSC_3N_4$ ... 17

4.1.6. Triboration of alkynes catalyzed by Pt_1-$UNSC_3N_4$ ... 18

4.2. One-pot oxidation. Synthesis of 2-phenyl-1,2-propanediol (2ja) 18
4.3. One-pot Suzuki-Miyaura coupling. Synthesis of (*E*)-4-(4-methylstyryl)benzonitrile (6ia) .. 18
5. Computational details 19
6. Product characterization 21
7. References 80

1. Methods

1.1. Reagents and Materials

Styrene (99%, Sigma-Aldrich), 4,4,4',4',5,5,5'5,'-octamethyl-2,2'-bi(1,3,2-dioxaborolane) (99.93%, AmBeed), bis(neopentyl glycolato)diboron (96%, Sigma-Aldrich), bis(catecholato)diboron (96%, Sigma-Aldrich), pinacolborane (98%, Apollo Scientific), 2-vinylnaphtalene (98%, J&K), 2,4,6-trimethylstyrene (95%, Alfa Aesar), 2-vinylanisole (98%, Sigma-Aldrich), 4-fluorostyrene (99%, Sigma-Aldrich), 4-bromostyrene (95%, Apollo Scientific), 4-methoxystyrene (97%, Sigma-Aldrich), *N*,*N*-dimethyl-4-vinylaniline (97%, AmBeed), *α*-methylstyrene (99%, Thermo Scientific), 4-fluoro-*α*-methylstyrene (97%, AmBeed), 4-chloro-α-methylstyrene (97%, AmBeed), *N*-allylphthalimide (95%, Angene), *trans*-stilbene (96%, Sigma-Aldrich), allylbenzene (98%, Thermo Scientific), *α*,4-dimethylstyrene (99.4%, Thermo Scientific), 1-octene (99%, Thermo Scientific), eugenol (≥98%, Sigma-Aldrich), 4-*tert*-butylstyrene (>98%, TCI), 5-hexen-2-one (>98%, TCI), methyl 4-vinylbenzoate (98%, Angene), phenylacetylene (98%, Apollo Scientific), 3',5'-bis-trifluoromethylphenyl acetylene (97%, Fluorochem), (triphenylsilyl)acetylene (98%, Sigma-Aldrich), diphenylacetylene (98%, AmBeed), ethynylbenzonitrile (97%, ABCR), 3-ethynylthiophene (96%, Sigma-Aldrich), 1-ethynyl-2-methoxybenzene (97%, AmBeed), 4-octyne (99%, Sigma-Aldrich), *trans*-β-Methylstyrene (99%, Sigma-Aldrich), 1H-indene (95%, Fluorochem), cyclohexene (99%, Sigma-Aldrich), 4-cyanostyrene (97%, Fluorochem), 4-ethynylanisole (97%, Sigma-Aldrich), 1-bromo-4-ethynylbenzene (97%, Sigma-Aldrich), 1-octyne (99%, Thermo Scientific), diphenylacetylene (98%, Sigma-Aldrich), 1-phenyl-1-propyne (99%, Sigma-Aldrich), 2-ethynyltoluene (97%, Sigma-Aldrich), pinacol vinylboronate (95%, Sigma-Aldrich), 2-chlorostyrene (97%, Sigma-Aldrich), 4-iodotoluene (98%, Sigma-Aldrich), [$Pd(PPh_3)_4$] (99%, Angene), caesium carbonate (99%, Sigma-Aldrich), toluene anhydrous (99.8%, Sigma-Aldrich), tetrahydrofuran anhydrous (≥99.9%, Sigma-Aldrich), methanol anhydrous (99.8%, Sigma-Aldrich), *n*-hexane (99%, Sigma-Aldrich), silica gel (MN-Kieselgel 60, 0.04-0.063 mm (230-400 mesh ASTM; Sigma-Aldrich)) were used as received. Hexanes (≥99.9%, hexane isomers + isopentane), ethyl acetate (≥99.7%), ethanol (99.9%), methanol (99.8%) were purchased from Avantor Performance Materials Poland. The chloroform-d_1 (99.8% + Ag) was purchased from Deutero and dried over 4Å molecular sieves.

1.2. Physico-chemical characterization

1.2.1. X-ray diffraction (XRD)

Powder X-ray diffraction (XRD) patterns of material were determined by X'Pert PRO MPD diffractometer (PANalytical) in the Bragg–Brentano geometry, equipped with an X'Celerator detector and programmable divergence and diffracted beam anti-scatter slits at room temperature using iron-

filtered Co-Kα radiation (40 kV, 30 mA, λ = 0.1789 nm). The angular range of measurement was set as 2θ = 5–90°, with a step size of 0.017°.

1.2.2. Transmission electron microscopy (TEM)

Microscopic TEM images were obtained by HRTEM TITAN 60-300 with X-FEG type emission gun, operating at 80 kV. This microscope is equipped with Cs image corrector and a STEM high-angle annular dark-field detector (HAADF). The point resolution is 0.06 nm in TEM mode. The elemental mappings were obtained by STEM-energy dispersive X-ray spectroscopy (EDS) with acquisition time 20 min. For HRTEM analysis, the powder samples were dispersed in ethanol and ultra-sonicated for 5 min. One drop of this solution was placed on a copper grid with holey carbon film.

1.2.3. X-ray photoelectron spectroscopy (XPS)

XPS surface investigation has been performed on the PHI 5000 Versa Probe II XPS system (Physical Electronics) with monochromatic Al-Kα source (15 kV, 50 W) and photon energy of 1486.7 eV. Dual beam charge compensation was used for all measurements. All the spectra were measured in the vacuum of 1.3×10^{-7} Pa and at the room temperature of 21 °C. The analysed area on each sample was a spot of 200 μm in diameter. The survey spectra were measured with pass energy of 187.850 eV and electronvolt step of 0.8 eV, while for the high-resolution spectra, pass energy of 23.500 eV and electronvolt step of 0.2 eV were used. The spectra were evaluated with the MultiPak (Ulvac - PHI, Inc.) software. All binding energy (BE) values were referenced to the carbon peak C1s at 284.80 eV.

1.2.4. X-ray absorption near edge structure (XANES) and Extended X-ray absorption fine structure (EXAFS)

The valence state, local chemical environment, and coordination pattern of samples were determined by X-ray absorption near edge structure (XANES) and Extended X-ray absorption fine structure (EXAFS) on 06ID-1 Hard X-ray MicroAnalysis (HXMA) beamline of Canadian light source. The energy range for the beamline was 5-40 KeV with a superconducting Wiggler source and photon flux of 1012@12 keV. The spot size was 0.8 x 1.5 mm while the spectral resolution was $1x10^{-4}$. For the measurement, samples were mounted on a hollow plastic holder by depositing samples on a Kapton® tape. Before the measurement, the energies were calibrated with standard samples with a $^{-1}$ lower atomic number metal. The measurement was done in transmittance mode and the Co edge was measured in the energy range of 7510-8350 eV. Few samples were analysed in the BioXAS-Spectroscopy sector of the Canadian light source operating in an energy range of 5-32 keV using a 22-poles (11 periods), 2.1 Tesla, Flat-top Wiggler source. The photon flux of the main beamline was 1 x 1012@12 keV with a spot size of 3 x 0.5 mm and spectral resolution of 1×10^{-4}. The optics and detector for BioXAS beamlines were: M1 mirror: Toroidal, 1 m, Si, Rh-coated, Sagittal radius: 33 mm. Water-cooled. Monochromator: LN2 cooled, Si(220), ϕ = 0o and 90o , double-crystal, non-fixed

exit slit (see mono gitch database) M2 mirror: Flat Bent, vertically focusing, 1.1 m. Si, Rh-coated Detectors: Ionization chambers, PIPS, Canberra 2 x 32-element HPGe solid-state (Main BL) and 32-element HPGe (Side BL). The acquired data were analysed using Athena software.

1.2.5. Operando X-ray absorption spectroscopy (XAS)

To discern the oxidation state and coordination structure change during the electrocatalytic conditions operando XAS was performed at the Hard X-ray MicroAnalysis (HXMA) beamline of the Canadian light source. For the measurement, a custom-made electrochemical cell was used which contains a plastic window fixed at 45º with respect to the beam. The whole assembly was made up of stainless-steel coated Teflon and an electrode connection was linked to the window cell. The sample deposited on carbon paper was mounted on the plastic window using Kapton® tape to make a connection with the electrode. The cell was filled with 1.0 M KOH and closed with a lid that has a Pt counter and Ag/AgCl electrode. The cell was fixed in a holder while keeping the window parallel to X-ray beams and ensuring that the beam is falling on the sample surface. After that XANES spectra were collected in transmittance mode at an open-circuit voltage (OCV) and 1.773 V vs RHE.

1.2.6. Inductively Coupled Plasma-Mass spectrometry (ICP-MS)

The metal content of fresh and reused catalyst was determined with ICP-MS (Agilent 7700x, Agilent, Japan). A weighted amount of sample from the catalyst (on a 0.01 mg read-out balance, Kern ABT 220-5DNM) was digested with nitric acid in microwave digester followed by dilution with water.

1.2.7. NMR analysis

^{1}H, ^{11}B and ^{13}C NMR spectra were recorded at 25 °C on Bruker UltraShield 300, Bruker Ascend™ 400 MHz NANOBAY with a number of scans (NS) for ^{1}H NMR = 16 or 32, ^{13}C NMR = 512 or 1024 (unless otherwise stated). Chemical shifts were reported in ppm with the reference to the residue portion solvent peak for ^{1}H, ^{13}C NMR or BF_3-Et_2O for ^{11}B, respectively. Chloroform-d_1, benzene-d_6, THF-d_8 were used as solvents and for internal deuterium lock. The multiplicities were reported as follows: singlet (s), doublet (d), doublet of doublets (dd), triplet (t), pentet (p), multiplet (m).

1.2.8. GC-MS analysis

The mass spectra of the products were obtained by GC-MS analysis on a Varian 431-GC with a 30 m Agilent J&W VF-200ms 0.25 mm capillary column and a Varian 220-MS mass spectrometry detector giving fragment ions in m/z with relative intensities (%) in parentheses. Temperature program used: 60 °C (3 min), 60 to 280 °C (10 °C/min), 280 °C (8 min).

1.2.9. High resolution mass spectrometry (HRMS)

High resolution mass spectra (HRMS) were obtained using Impact HD mass spectrometer (Q-TOF type instrument equipped with electrospray ion source; Bruker Daltonics, Germany). The sample solutions (DCM:MeOH) were infused into the ESI source by a syringe pump (direct inlet) at the flow rate of 3 μL/min. The instrument was operated under the following optimized settings: end plate voltage 500 V; 12 capillary voltage 4.2 kV; nebulizer pressure 0.3 bar; dry gas (nitrogen) temperature 200 °C; dry gas flow rate 4 L/min. The spectrometer was previously calibrated with the standard tune mixture.

1.2.10. FT-IR analysis

FT-IR spectra were measured on a Nicolet iS50 FT-IR spectrometer (Thermo Scientific) equipped with a built-in ATR accessory with ATR diamond unit. In all experiments, 16 scans at a resolution of 2 cm^{-1} were performed.

1.2.11. Elemental analysis

Elemental analyses were performed using the Vario EL III instrument. The content of hydrogen and carbon was obtained as data in percentage.

2. Preparation of materials

2.1. Synthesis of $UNSC_3N_4$ and Platinum single atom Pt_1-$UNSC_3N_4$ over ultra-nanosheet $UNSC_3N_4$

For the formulation of materials such as g-C_3N_4, $UNSC_3N_4$, Pt_1-$UNSC_3N_4$, and Pt_N-$UNSC_3N_4$. The procedure was as follows; Typically, 20 g dicyandiamide was followed by drying in an oven at 90°C. 20 g of the oven-dried powder was placed in a boat crucible and heated to 550 °C at a rate of 10 °C min-1 and maintained for 2 h. The resultant yellow material was characterized as g-C_3N_4. (Yield ~ 5.00 g). The nC_3N_4 nanosheets were prepared as reported elsewhere. Briefly, 1.00 g of gC_3N_4 was dispersed into the alumina crucible with good contact between gC_3N_4 and air and then heated at 520 °C for 4.5 h, using a heating rate of 2 °C min-1. A pale-yellow powdered material was obtained (yield ~ 200 mg) named as nanosheets of carbon nitride (nC_3N_4). The nanosheets of carbon nitride were used as such as photoactive support for the dispersion of platinum species, and a schematic representation of the process is shown in Figure 1a. Before addition of platinum aqueous solution nanosheets of carbon nitride were kept under sonication (24 min) for ultra-nanosheet $UNSC_3N_4$ formulation. In particular, hexachloroplatinic acid solid in (Millipore) water (0.02 mg, 10 mL) was added dropwise to ultra-nanosheet $UNSC_3N_4$ (500 mg), and the suspension was kept under sonication for 30 min. At the end of this step, the suspension was left for 12 h at ambient temperature. Furthermore, 1.5 g of sodium

borohydride ($NaBH_4$) was added, and the reaction mixture was stirred for another 12 h at 80 °C, followed by 10 runs (each of 2 min) of rapid microwave heating (LG, Power 1000 Watt; P/No MEZ66853207). The final product was washed with DI water twice using high-speed Centrifugation and kept for fridge drying for 24 h before receiving the final product. (Yield ~520 mg). The authenticity of the obtained single-atom catalyst (herein, indicated as Pt_1-$UNSC_3N_4$) was confirmed through the in-depth characterization. Platinum nanoparticles over $UNSC_3N_4$ were formulated by conventional incipient wetness impregnation of $UNSC_3N_4$ with an aqueous solution (10 mL) of hexachloroplatinic acid solid (0.02 mg), followed by ($NaBH_4$) addition and drying at 100 °C for 10 h. The latter catalyst is indicated as Pt_N-$UNSC_3N_4$.

3. Materials characterization

3.1. TEM analysis

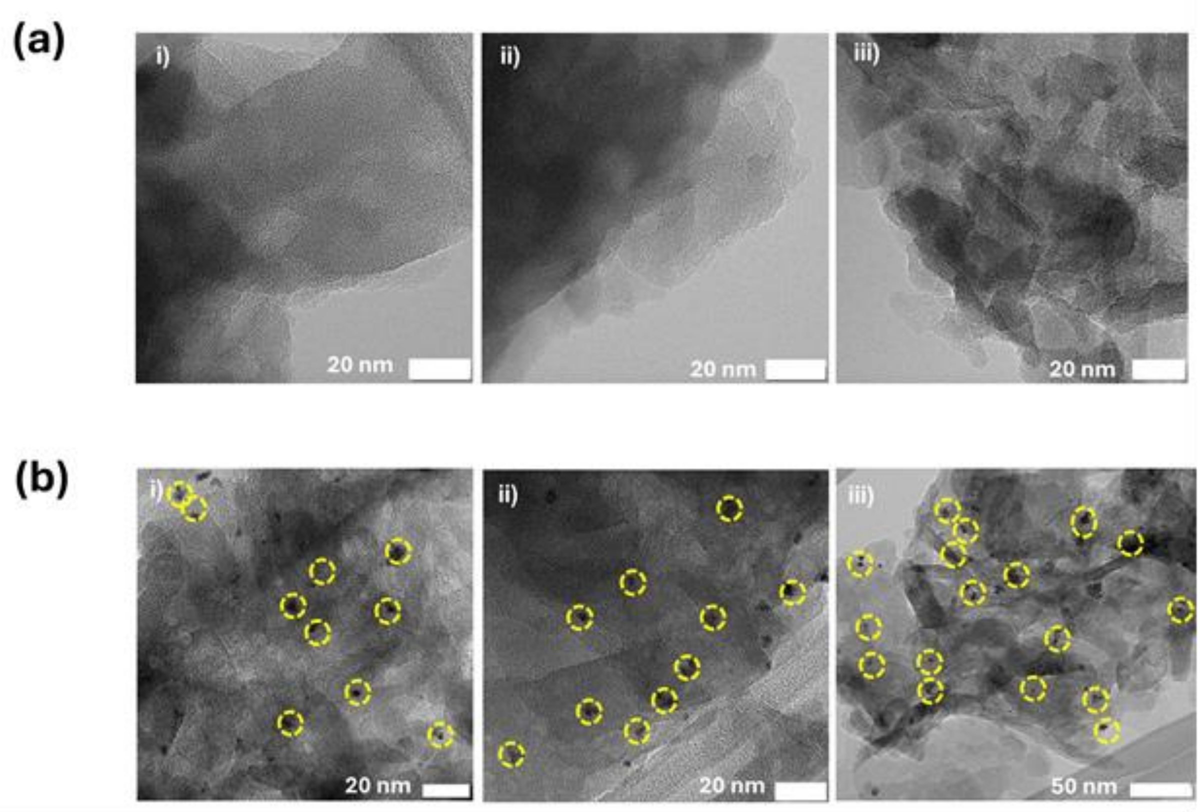

Figure S1: TEM images of a) Pt_1-$UNSC_3N_4$ (single atom) and b) Pt_N-$UNSC_3N_4$ at 20 nm 50 nm magnification (nanoparticle).

3.2. *XPS analysis*

Table S1: Pt_1-UNSC_3N_4, XPS SCAN data sheets.

Name	Start BE	Peak BE	End BE	Height CPS	FWHM eV	Area (P) CPS.eV	Area (N) TPP-2M	Atomic %
Pt4f7 Pt(II)	86.9	72.61	58.1	8873.7	1.9	20588.5	27.3	**89.1**
Pt4f7 Pt(IV)	86.9	74.75	58.1	732	2.5	2507.8	3.3	**10.9**

Table S2: Pt_1-UNSC_3N_4, C1s, XPS SCAN data sheets.

Name	Peak BE	FWHM eV	Area (P) CPS.eV	Atomic %	Q
C1s C-C	284.74	1.57	16775.3	**8.57**	1
C1s C-O/C-N	286.59	1.65	7251.58	**3.71**	1
**C1s C=O/C=N**	287.83	1.1	116591.4	**59.69**	1

Table S3: Pt_1-UNSC_3N_4, N1s, XPS SCAN data sheets.

Name	Peak BE	FWHM eV	Area (P) CPS.eV	Atomic %	Q
N1s Pyridinic N	398.41	1.25	261681.1	**63.93**	1
N1s Pyrrolic N	399.35	1.37	81802.49	**20**	1
N1s Graphitic N	400.69	1.56	65680.9	**16.07**	1

Table S4: N1s Peak in UNSC_3N_4.

Name	Start BE	Peak BE	End BE	Height CPS	FWHM eV	Area (P) CPS.eV	Area (N) TPP-2M	Atomic %
N1s C-N=C	410	398.33	392	364770.4	1.2	510805.4	4610.9	**73.2**
N1s N-C(3)	410	399.71	392	67696.5	1.8	129940.1	1174.1	**18.6**
N1s C-N-H	410	400.86	392	42504.6	1.3	57000.5	515.4	**8.2**

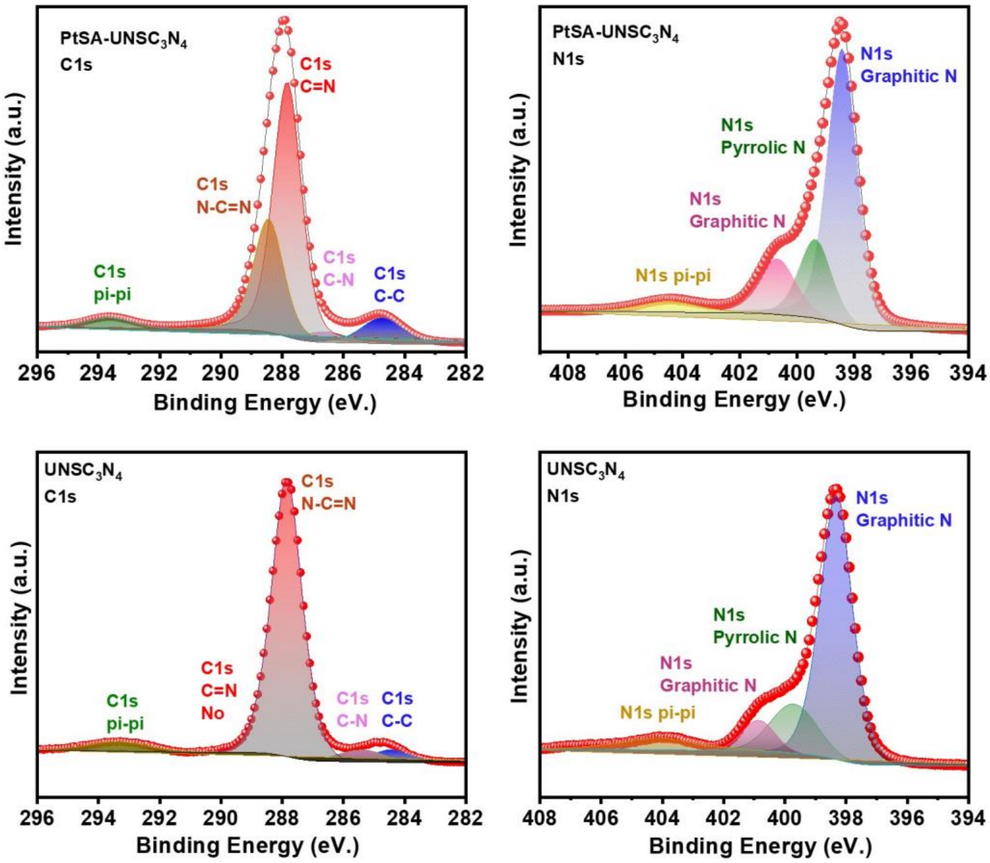

Figure S2: High-resolution XPS spectra of C 1s, N 1s, of Pt_1-$UNSC_3N_4$, and $UNSC_3N_4$.

3.3. *XAS analysis*

Table S5. The coordination number for the Pt_1-$UNSC_3N_4$and Pt_N-$UNSC_3N_4$ catalyst.

Sample	bond	length	CN number	△E	s² (10⁻³)	R-factor
**Pt foil**	Pt-Pt	2.77(0.02)	**12***	7.2(0.3)	5.4 (0.4)	0.005
**PtSA-UNSC$_3$N$_4$**	Pt-N/O	2.14(0.02)	**4.7 (0.2)**	7.0 (0.4)	4.6 (0.2)	0.01
**PtN-UNSC$_3$N$_4$**	Pt-N/O	2.10 (0.04)	**4.1 (0.4)**	7.9 (0.9)	9.2 (0.8)	0.01
	Pt-Pt	2.75 (0.03)	**10.1 (0.5)**	3.6 (0.5)	4.8 (0.3)	

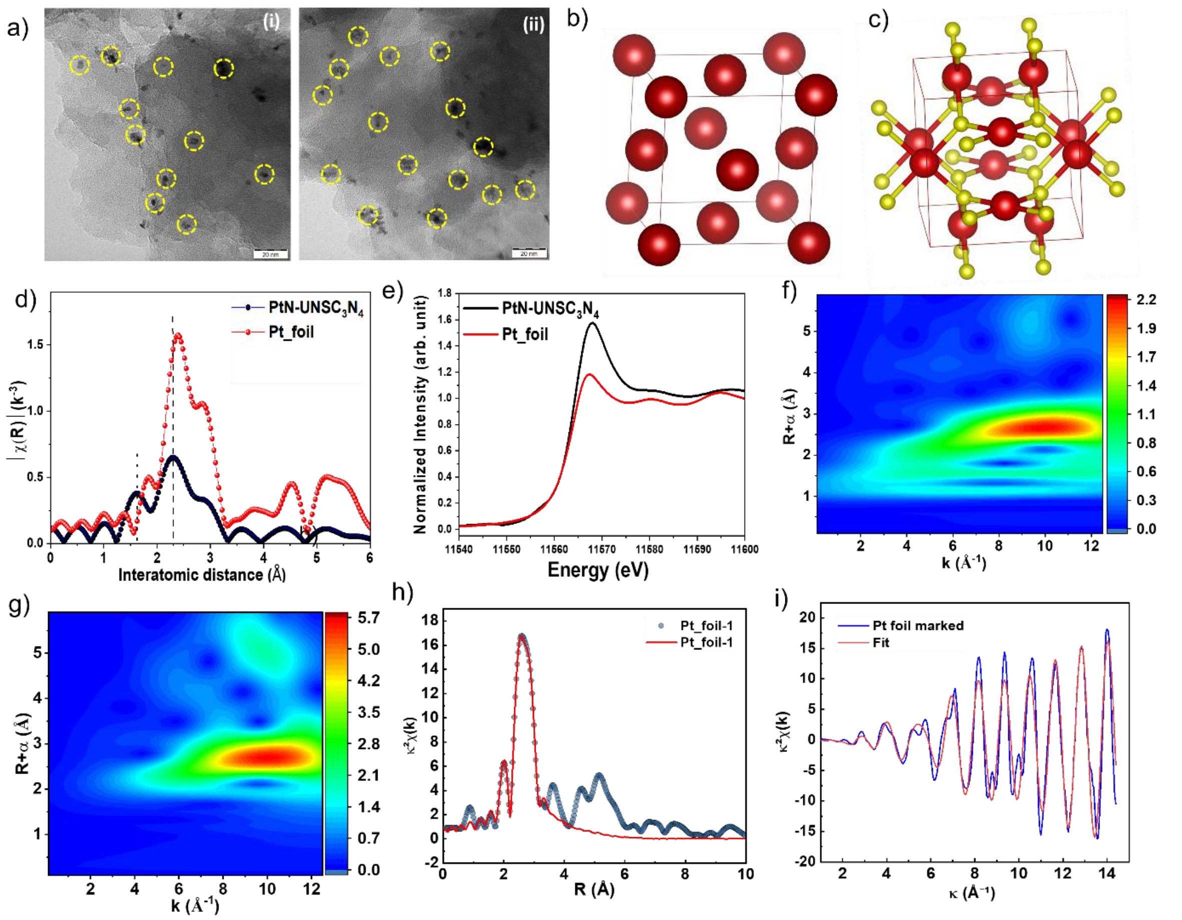

Figure S3. Characterizations of PtN-$UNSC_3N_4$ a) TEM images of PtN-$UNSC_3N_4$ at 20 nm magnification (nanoparticle and single atoms); b) unit structure of Pt metal, c) unit structure of PtO_2, d) Fourier-transform EXAFS spectrum of Pt foil and PtN-$UNSC_3N_4$; e) Pt K-edge XANES spectrum of PtSI-NP-$UNSC_3N_4$ and Pt foil; f, and g) Fitted and experimental Fourier transforms of the EXAFS spectra for PtSI-NP-$UNSC_3N_4$ (solid line: experimental data, dots: fit).; h) Wavelet transform (WT) of PtSI-NP-$UNSC_3N_4$; is the i) structure model for PtSI-NP-$UNSC_3N_4$.

4. Catalytic part

4.1. Optimization of reaction conditions

4.1.1. Diboration of alkenes catalyzed by Pt_1-UNSC$_3$N$_4$

In a standard procedure, a dram vial was charged with Pt_1-UNSC$_3$N$_4$ in the air atmosphere. Subsequently, styrene (**1**, 1.0 mmol), bis(pinacolato)diboron pinacolborane (1.0 mmol, 1.0 equiv.) and solvent (0.25 mL) were added. The reaction mixture was stirred under the conditions outlined in Tables **S6–S11**. Upon completion of the reaction, the crude mixture was first analyzed by gas chromatography-mass spectrometry (GC-MS), which was used primarily for qualitative purposes-to confirm product formation and to detect any side products. Quantitative analysis, including determination of conversion, yield, and selectivity, was performed using ^{1}H NMR spectroscopy, with mesitylene added as an internal standard. The relevant signals for the product and mesitylene were integrated, and the relative integrals were used to calculate conversion, selectivity and yield.

Table S6. Influence of the solvent on Pt_1-UNSC$_3$N$_4$ catalyzed diboration of styrene.

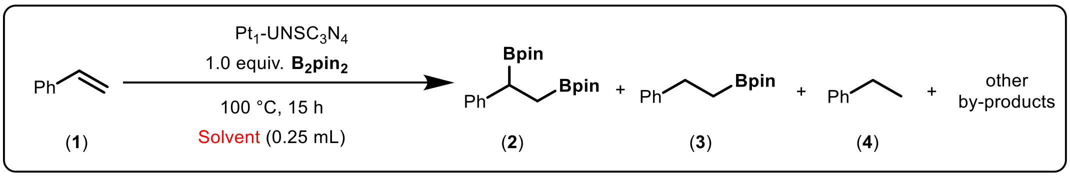

Entry	Solvent	Conv. of **1** [%][a]	Selectivity (**2**/**3**/**4**)[a]	Yield of **2** [%][a]
1	Toluene:MeOH (3:1)	>99	86/14/0	86
2[b]	Toluene:MeOH (3:1)	99	99/1/0	98
3[c]	Toluene:MeOH (3:1)	95	99/1/0	94
4	Toluene:EtOH (3:1)	50	57/43/0	29
5	Toluene:H_2O (3:1)	12	99/0/1	12
6	Toluene	20	99/0/1	20
7[b]	THF	5	5/0/95	0
**8**[c]	**MeOH**	**95**	**99/1/0**	**94**
9[b]	neat	n.r.	-	-

Reaction conditions: [Alkene]:[B_2pin_2]:[Pt_1-UNSC$_3$N$_4$] = 1:1:(5.0×10^{-4} mol% Pt), solvent (0.25 mL), 100 °C, 15 h. [a]Determined by ^{1}H NMR analysis using mesitylene as an internal standard. [b]70 °C. [c]50 °C.

Table S7. Influence of the temperature on Pt_1-UNSC$_3$N$_4$ catalyzed diboration of styrene.

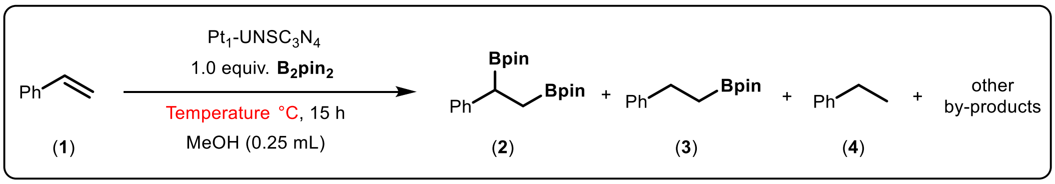

Entry	Temp. [°C]	Conv. of **1** [%][a]	Selectivity (**2**/**3**/**4**)[a]	Yield of **2** [%][a]
**1**	**50**	**95**	**99/1/0**	**94**
2	40	71	99/1/0	70
3	25	30	99/1/0	30

Reaction conditions: [Alkene]:[B_2pin_2]:[Pt_1-UNSC$_3$N$_4$] = 1:1:(5.0×10^{-4} mol% Pt), MeOH (0.25 mL), 15 h. [a]Determined by ^{1}H NMR analysis using mesitylene as an internal standard.

Table S8. Influence of the catalyst loading on Pt_1-UNSC$_3$N$_4$ catalyzed diboration of styrene.

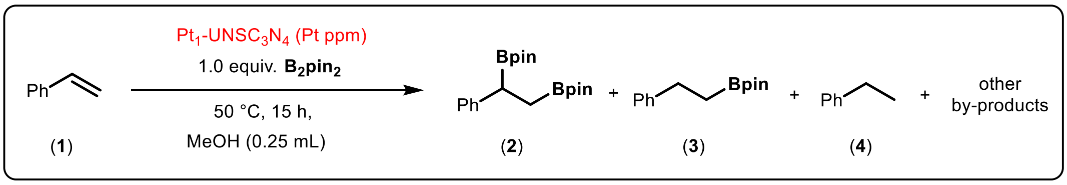

Entry	Cat. loading [Pt ppm]	Conv. of **1** [%][a]	Selectivity (**2**/**3**/**4**)[a]	Yield of **2** [%][a]
1	none	n.r.	-	-
2	5 mg (Pt: 2.5 ppm) – 2.5×10^{-4} mol% Pt	78	95/5/0	74
**3**	**10 mg (Pt: 5 ppm)** – 5.0×10^{-4} mol% Pt	**95**	**99/1/0**	**94**
4	20 mg (Pt: 10 ppm) – 1.0×10^{-3} mol% Pt	99	99/1/0	98
5[b]	20 mg (Pt: 10 ppm) – 1.0×10^{-3} mol% Pt	98	99/1/0	97

Reaction conditions: [Alkene]:[B_2pin_2]:[Pt_1-UNSC$_3$N$_4$] = 1:1:X, MeOH (0.25 mL), 50 °C, 15 h. [a]Determined by ^{1}H NMR analysis using mesitylene as an internal standard. [b]10 h.

Table S9. Influence of the B_2pin_2 loading on Pt_1-$UNSC_3N_4$ catalyzed diboration of styrene.

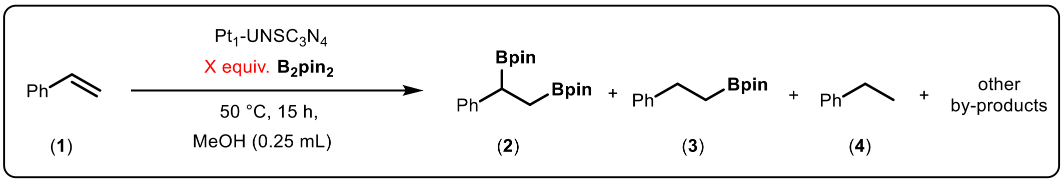

Entry	B_2pin_2 (equiv.)	Conv. of **1** [%][a]	Selectivity (**2**/**3**/**4**)[a]	Yield of **2** [%][a]
1	0.5	47	99/1/0	47
**2**	**1.0**	**95**	**99/1/0**	**94**
3	1.2	99	99/1/0	98
4[b]	1.2	96	99/1/0	95

Reaction conditions: [Alkene]:[B_2pin_2]:[Pt_1-$UNSC_3N_4$] = 1:X:(5.0×10^{-4} mol% Pt), MeOH (0.25 mL), 50 °C, 15 h. [a]Determined by ^{1}H NMR analysis using mesitylene as an internal standard. [b]12 h.

Table S10. Influence of the diborane on Pt_1-$UNSC_3N_4$ catalyzed diboration of styrene.

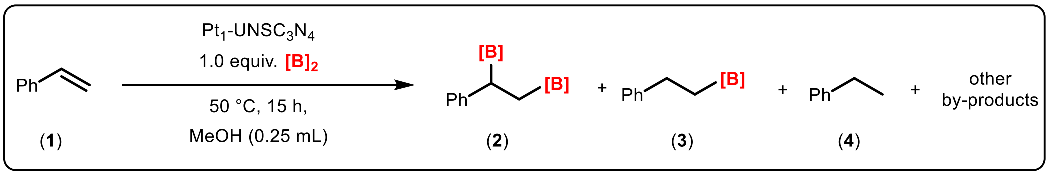

Entry	Diborane	Conv. of **2** [%][a]	Selectivity (**3**/**4**/**5**)[a]	Yield of **2** [%][a]
**1**	$\mathbf{B_2pin_2}$	**95**	**99/1/0**	**94**
2	B_2cat_2	n.r.	-	-
3	B_2neop_2	40	>99/0/0	40

Reaction conditions: [Alkene]:[diborane]:[Pt_1-$UNSC_3N_4$] = 1:1:(5.0×10^{-4} mol% Pt), MeOH (0.25 mL), 50 °C, 15 h. [a]Determined by ^{1}H NMR analysis using mesitylene as an internal standard.

Table S11. Influence of the catalyst type on diboration of styrene.

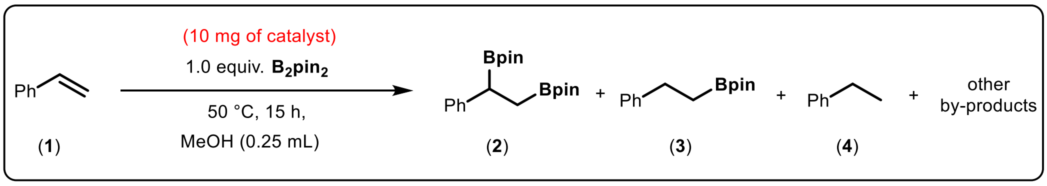

Entry	Catalyst	Conv. of **1** [%][a]	Selectivity (**2**/**3**/**4**)[a]	Yield of **5** [%][a]
**1**	**Pt_1-$UNSC_3N_4$ (5.0×10^{-4} mol% Pt)**	**95**	**99/1/0**	**94**
2	Pt_N-$UNSC_3N_4$ (1.15×10^{-3} mol% Pt)	19	95/5/0	18
3[b]	Pt_N-$UNSC_3N_4$ (2.30×10^{-3} mol% Pt)	84	92/8/0	77
4	g-C_3N_4 (0 mol% Pt)	n.r.	-	-

Reaction conditions: [Alkene]:[B_2pin_2]:[catalyst] = 1:1:X, MeOH (0.25 mL), 50 °C, 15 h. [a]Determined by ^{1}H NMR analysis using mesitylene as an internal standard. [b]Pt: 23 ppm (20 mg of catalyst was used)

4.1.2. Hydroboration of alkenes catalyzed by Pt_1-UNSC$_3$N$_4$

A Schlenk's vessel was charged with Pt_1-UNSC$_3$N$_4$ under an argon atmosphere. Subsequently, styrene (**1**, 1.0 mmol), anhydrous solvent (0.25 mL) and pinacolborane (1.0 mmol, 1.0 equiv.) were then added. The reactions were carried out in the conditions listed in Tables **S12–S15**. Upon completion of the reaction, the crude mixture was first analyzed by gas chromatography–mass spectrometry (GC–MS), which was used primarily for qualitative purposes—to confirm product formation and to detect any side products. Quantitative analysis, including determination of conversion, yield, and selectivity, was performed using ^{1}H NMR spectroscopy, with mesitylene added as an internal standard. The relevant signals for the product and mesitylene were integrated, and the relative integrals were used to calculate conversion, selectivity and yield.

Table S12. Influence of the solvent on Pt_1-UNSC$_3$N$_4$ catalyzed hydroboration of styrene.

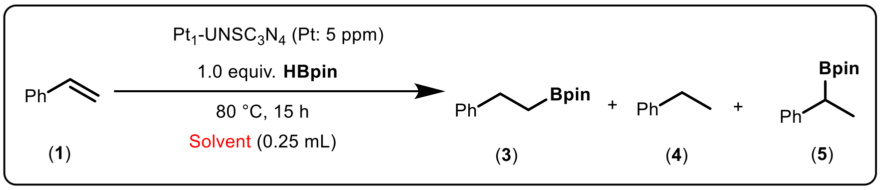

Entry	Solvent	Conv. of **1** [%][a]	Selectivity (**3/5/4**)[a]	Yield of **3** [%][a]
1	Toluene	67	92/2/6	62
2	1,4-Dioxane	75	95/0/5	71
**3**	**THF**	**>99**	**95/1/4**	**95**
4[b]	THF	19	94/1/5	18
5	MeOH	n.r.	-	-

Reaction conditions: [Alkene]:[HBpin]:[Pt_1-UNSC$_3$N$_4$] = 1:1:(5.0×10^{-4} mol% Pt), solvent (0.25 mL), 80 °C, 15 h, Ar. [a]Determined by ^{1}H NMR analysis using mesitylene as an internal standard. [b]reaction performed in the air atmosphere.

Table S13. Influence of the temperature on Pt_1-UNSC$_3$N$_4$ catalyzed hydroboration of styrene.

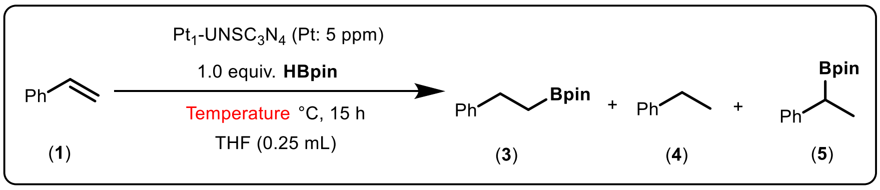

Entry	Temperature [°C]	Conv. of **1** [%][a]	Selectivity (**3/5/4**)[a]	Yield of **3** [%][a]
1	80	>99	95/1/4	95
2	50	98	>99/0/0	98
3	25	90	>99/0/0	90
**4[b]**	**25**	**98**	**>99/0/0**	**98**
5[c]	25	84	>99/0/0	84

Reaction conditions: [Alkene]:[HBpin]:[Pt_1-UNSC$_3$N$_4$] = 1:1:(5.0×10^{-4} mol% Pt), THF (0.25 mL), 15 h. [a]Determined by ^{1}H NMR analysis using mesitylene as an internal standard. [b]18 h. [c]12 h.

Table S14. Influence of the HBpin loading on Pt_1-UNSC_3N_4 catalyzed hydroboration of styrene.

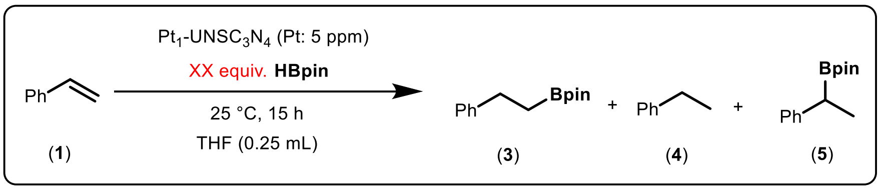

Entry	HBpin (equiv.)	Conv. of **1** [%][a]	Selectivity (**3/5/4**)[a]	Yield of **3** [%][a]
1	1.5	>99	>99/0/0	99
2[b]	1.5	98	>99/0/0	98
3	1.2	99	>99/0/0	99
**4**	**1.0**	**98**	**>99/0/0**	**98**

Reaction conditions: [Alkene]:[HBpin]:[Pt_1-UNSC_3N_4] = 1:XX:(5.0×10^{-4} mol% Pt), THF (0.25 mL), 25 °C, 15 h. [a]Determined by ^{1}H NMR analysis using mesitylene as an internal standard.[b]12 h.

Table S15 Influence of the catalyst loading on Pt_1-UNSC_3N_4 catalyzed hydroboration of styrene.

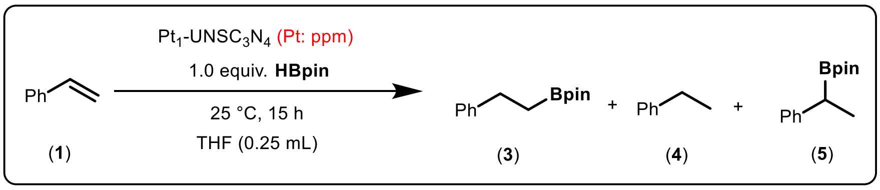

Entry	Catalyst loading [Pt ppm]	Conv. of **1** [%][a]	Selectivity (**3/5/4**)[a]	Yield of **3** [%][a]
1	20 mg (Pt: 10 ppm)	99	>99/0/0	99
2[b]	20 mg (Pt: 10 ppm)	98	>99/0/0	98
**3**	**10 mg (Pt: 5.0 ppm)**	**98**	**>99/0/0**	**98**
4	7 mg (Pt: 3.5 ppm)	90	99/0/0	90
5	5 mg (Pt: 2.5 ppm)	71	99/0/0	71
6	none	n.r.	-	-
7	10 mg g-C_3N_4	n.r.	-	-

Reaction conditions: [Alkene]:[HBpin]:[catalyst] = 1:1:(X mol% Pt),, THF (0.25 mL), 25 °C, 15 h. [a]Determined by ^{1}H NMR analysis using mesitylene as an internal standard. [b]10 h.

4.1.3. Diboration of alkenes catalysed by Pt_1-UNSC_3N_4 under optimized reaction conditions

A dram vial with screw cap was charged with Pt_1-UNSC_3N_4 (10 mg, Pt: 5 ppm). Subsequently, olefin (**1a–1t**, 1.0 mmol), bis(pinacolato)diboron (1.0 equiv., 1.0 mmol) and MeOH (0.25 mL) were added under an air atmosphere. The reaction was carried out for 15 h at 50 °C. The reaction mixture was cooled down and characterized by GC-MS and ^{1}H NMR analyses using mesitylene as an internal standard to confirm the product formation and determine conversion, selectivity and yield. The products (**2a–2h**, **2j–2m,** **2o**, **2q–t**) were purified by silica gel column chromatography (phase: hexanes/ethyl acetate = 100:0 → 95:5) to afford the desired products. The known compounds were

characterized by ^{1}H and ^{13}C NMR analyses. The new compounds were additionally characterized by HRMS, FT-IR and ^{11}B NMR analyses.

Gram-scale diboration of α-methylstyrene ***1j*:**

Pt_1-UNSC_3N_4 (100 mg, Pt: 50 ppm, 5.0×10^{-3} mol% Pt), *α*-methylstyrene **1j** (10 mmol), bis(pinacolato)diboron (10 mmol, 1.0 equiv.) and MeOH (2.5 mL) were added into dram vial with screw cap and stirred for 15 h at 50 °C under an air atmosphere. The reaction mixture was cooled down and crude reaction mixture was analysed by GC-MS and ^{1}H NMR analyses. Volatile components were evaporated under vacuum (10^{-3} mbar), and the product was purified by silica gel column chromatography (phase: hexanes/ethyl acetate = 100:0 → 97:5) to afford **2j** as a white solid (3.01 g, isolation yield: 81%).

*Repetitive diboration of styrene (**1a**) with Pt_1-UNSC_3N_4:*

A dram vial with screw cap was charged with Pt_1-UNSC_3N_4 (10 mg, Pt: 5 ppm, 5.0×10^{-4} mol% Pt). Subsequently, olefin (**1a**, 1.0 mmol), bis(pinacolato)diboron (1.0 equiv., 1.0 mmol) and MeOH (0.25 mL) were added under an air atmosphere. The reaction was carried out for 15 h at 50 °C. The reaction mixture was cooled and the catalyst was filtered through filter paper. Filter paper was washed with MeOH (3 × 5 mL). Filtrates were combined and solvent was evaporated. After evaporation, the filtrates were weighed and characterized by GC–MS and ^{1}H NMR analyses. Pt_1-UNSC_3N_4 was recovered, dried in oven at 80 °C for 3 hours and used in the next reaction.

4.1.4. Hydroboration of alkenes catalyzed by Pt_1-UNSC_3N_4 under optimized reaction conditions

A Schlenk's vessel was charged with Pt_1-UNSC_3N_4 (10 mg, Pt: 5 ppm, 5.0×10^{-4} mol% Pt) in an argon atmosphere. Subsequently, olefin (1.0 mmol), THF (0.25 mL) and pinacolborane (1.0 equiv., 1.0 mmol) were added under argon atmosphere. The reaction was carried out for 18 h at 25 °C. The reaction mixture was cooled down and characterized by GC-MS and ^{1}H NMR analyses. The products (**3a**, **3j**, **3k**, **3u–z**, **3aa-ac**) were purified by silica gel column chromatography (phase: hexanes/ethyl acetate = 100:0 → 95:5) to afford the desired products. The known compounds were characterized by ^{1}H and ^{13}C NMR analyses.

4.1.5. Hydroboration of alkynes catalyzed by Pt_1-UNSC_3N_4

A Schlenk's vessel was charged with Pt_1-UNSC_3N_4 (10 mg, Pt: 5 ppm, 5.0×10^{-4} mol% Pt) in an argon atmosphere. Subsequently, alkyne (1.0 mmol), anhydrous THF (0.25 mL) and pinacolborane (1.0 equiv., 1.0 mmol) were added under an argon atmosphere. The reaction was carried out for 18 h at 25 °C. The reaction mixture was cooled down and characterized by GC-MS and ^{1}H NMR analyses. The products (**8a**, **8e**, **8f**, **8h–8m**) were purified by silica gel column chromatography (phase: hexanes/ethyl

acetate = 100:0 → 95:5) to afford the desired products. The known compounds were characterized by ^{1}H and ^{13}C NMR analyses.

4.1.6. Triboration of alkynes catalyzed by Pt_1-UNSC$_3$N$_4$

A dram vial with screw cap was charged with Pt_1-UNSC_3N_4 (10 mg, Pt: 5 ppm, 5.0×10^{-4} mol% Pt). Subsequently, alkyne (1.0 mmol), bis(pinacolato)diboron (1.5 equiv., 1.5 mmol) and MeOH (0.25 mL) were added under air atmosphere. The reaction was carried out for 15 h at 50 °C. The reaction mixture was cooled down and characterized by GC-MS and ^{1}H NMR analyses. The products (**7a–e**) were purified by silica gel column chromatography (phase: hexanes/ethyl acetate = 100:0 → 98:2) to afford the desired products. The known compounds were characterized by ^{1}H and ^{13}C NMR analyses.

4.2. One-pot oxidation. Synthesis of 2-phenyl-1,2-propanediol (2ja)

A dram vial with screw cap was charged with Pt_1-UNSC_3N_4 (10 mg, Pt: 5 ppm, 5.0×10^{-4} mol% Pt). Subsequently, olefin (**1j**, 1.0 mmol), bis(pinacolato)diboron (1.0 equiv., 1.0 mmol) and MeOH (0.25 mL) were added under air atmosphere. The reaction was carried out for 15 h at 50 °C. Afterward, volatiles were evaporated and THF (1 mL) at 0 °C (ice bath) was added. Then, solution of 2 M aq. NaOH and 30% aq. H_2O_2 (2:1, 3 mL) was added dropwise. The resulting solution was allowed to stir for 5 hours at room temperature. Afterward, the solution was diluted with water (2 mL) and extracted with ethyl acetate (3 x 2 mL). The combined organic layers were dried over Na_2SO_4 and solvent was evaporated under vacuo. The product was purified by silica gel column chromatography (phase: hexanes/ethyl acetate = 100:0 → 50:50) to afford **2ja** (140 mg, isolation yield: 92%) as a colorless oil. The product was characterized by ^{1}H and ^{13}C NMR analyses.

4.3. One-pot Suzuki-Miyaura coupling. Synthesis of (E)-4-(4-methylstyryl)benzonitrile (6ia)

A Schlenk's vessel was charged with Pt_1-UNSC_3N_4 (10 mg, Pt: 5 ppm, 5.0×10^{-4} mol% Pt) in an argon atmosphere. Subsequently, 4-ethynylbenzonitrile (**6i**, 1.0 mmol), THF (0.25 mL) and pinacolborane (1.0 equiv., 1.0 mmol) were added under argon atmosphere. The reaction was carried out for 18 h at 25 °C. Afterwards, the reaction mixture was cooled down and volatiles were evaporated. Subsequently, [Pd(PPh_3)$_4$] (0.005 mmol), and 4-iodotoluene (1.2 equiv., 1.2 mmol) were placed in the Schlenk vessel and evacuated. Then THF (3 mL) and Cs_2CO_3 (1.5 equiv., 1.5 mmol) were added under argon atmosphere and stirred for 24 h at 70 °C. Afterwards, the mixture was cooled to room temperature. Then, water was added (10.0 mL), and the mixture was extracted with EtOAc (3 × 15 mL). The combined organic layers were washed with brine, dried over Na_2SO_4 and solvent was evaporated under vacuo. The product was purified by silica gel column chromatography (phase: hexanes/ethyl acetate = 100:0 → 95:5) to afford **6ia** (182 mg, isolation yield: 83%) as a white solid. The product was characterized by ^{1}H and ^{13}C NMR analyses.

5. Computational details

The density functional theory (DFT) calculations were performed to elucidate the possible reaction path of the selective alkene diboration and hydroboration reaction catalyzed by Pt_1-UNSC_3N_4. The structures using finite-size models of all investigated species were optimized by the *ω*B97X-D functional[1] coupled with the Karlsruhe basis sets def2-SVP[2] as implemented in using Gaussian software.[3] For open-shell systems, the spin-unrestricted formalism was applied. The solvent effects were treated by using the universal continuum solvation model based on electron density (SMD)[4] and the relative permittivity of 32.7 to simulate the methanol solvent. Transition states (TSs) were checked to display one imaginary frequency. All standard Gibbs energies were calculated at 323 K and 1 atm using rigid-rotor, harmonic oscillator and ideal gas approximations and principles of statistical thermodynamics. The analysis of Wiberg bond indices (WBI)[5-6] was performed to elucidate the bond orders.

We employed a finite-size model of triazine-based graphitic carbon nitride with bridge nitrogen atoms saturated by hydrogen atoms and with Pt embedded in the center, with charge 2+ and multiplicity 3 (Pt_1-UNSC_3N_4). The size of the model was chosen as a compromise between the computational demands and accuracy to provide chemically meaningful results. Due to the larger atomic radius of the Pt ion, it protruded from the C_3N_4 plane with Pt–N bond distances of 2.2 Å.

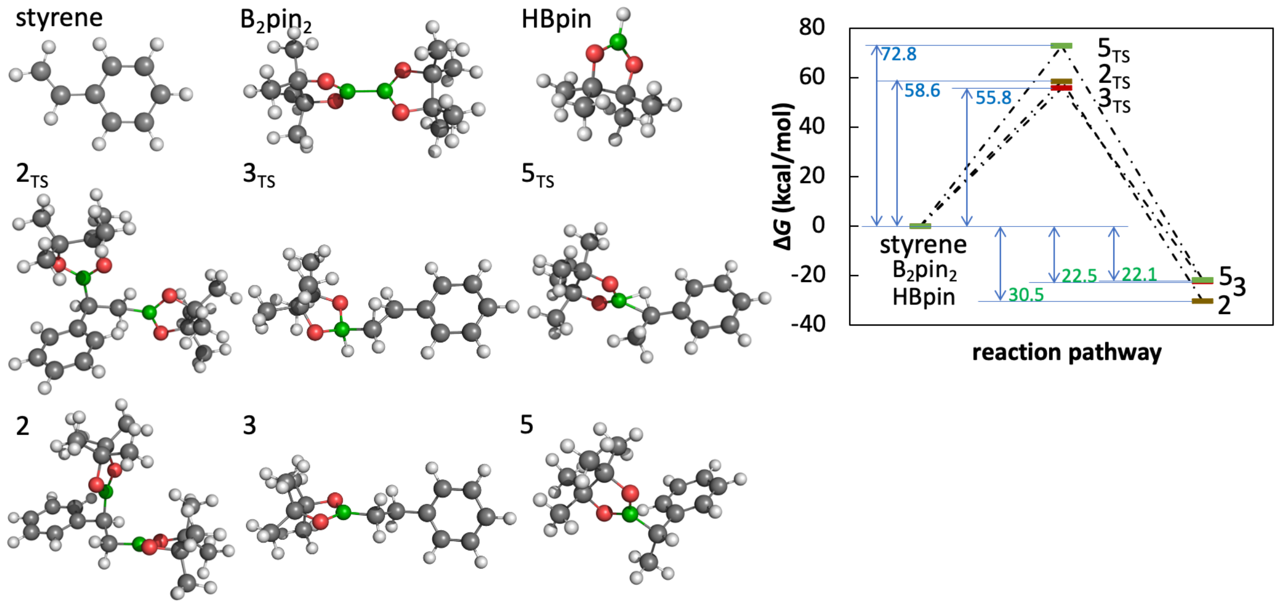

Figure S4. Molecular structures and corresponding energy profile of an uncatalyzed diboration and hydroboration of styrene. Left panels show the optimized geometries of reactants, products, and TSs.

The labeling of the structures is consistent with the labeling of the synthesized products. Carbon atoms shown as dark grey balls, hydrogen in white, boron in green, oxygen in red. The right panel shows an associated reaction profile with calculated standard Gibbs energies (323 K, 1 atm) in methanol.

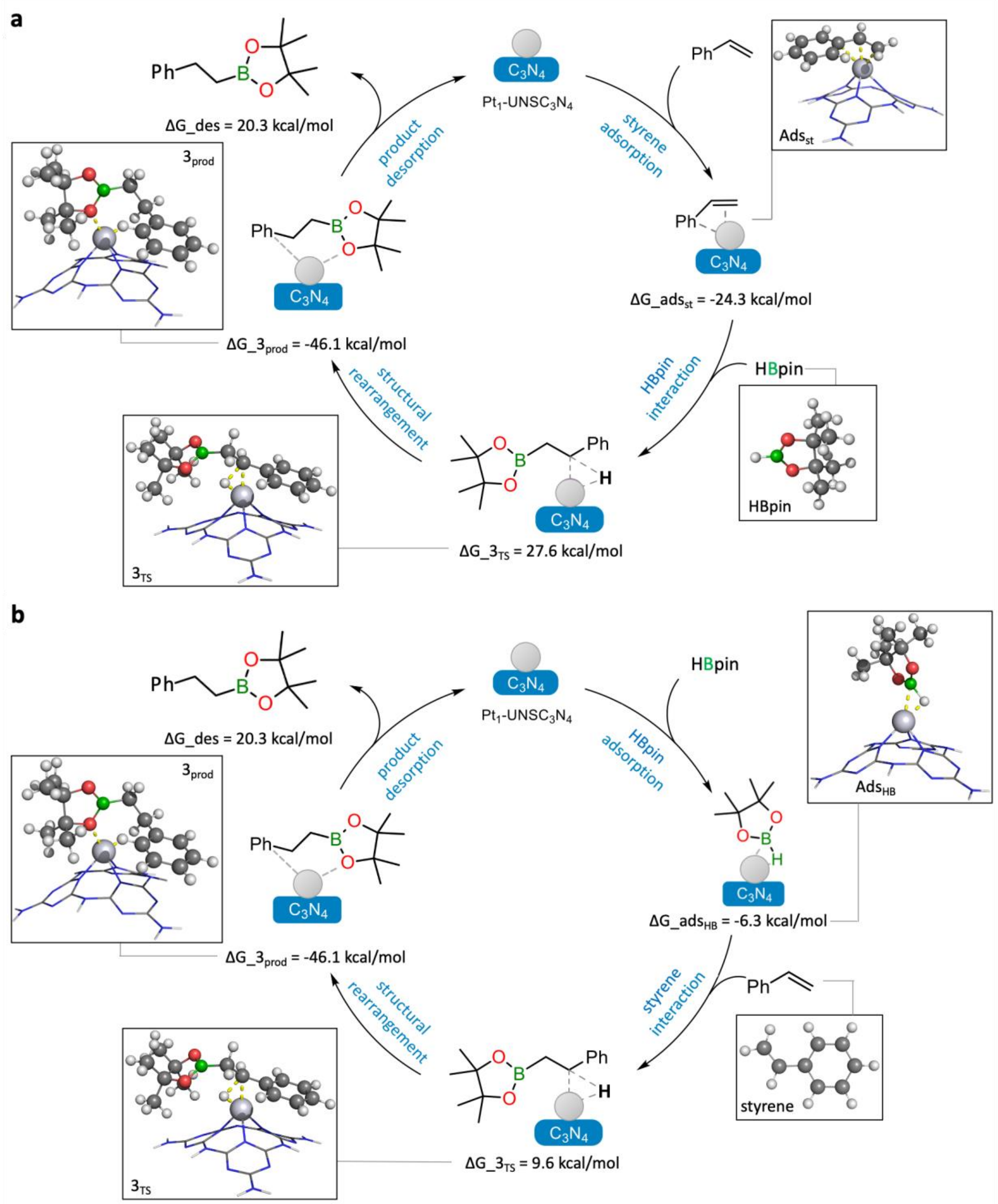

Scheme S1. Possible reaction mechanism for the hydroboration of styrene catalyzed by Pt_1-$UNSC_3N_4$. a) Styrene is adsorbed on the catalyst and interacts with HBpin, b) HBpin is adsorbed on the catalyst and interacts with styrene.

6. Product characterization

2,2'-(1-Phenylethane-1,2-diyl)bis(4,4,5,5-tetramethyl-1,3,2-dioxaborolane) (**2a**)

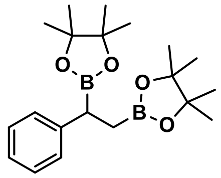

^{1}H NMR (300 MHz, $CDCl_3$, δ, ppm): 7.22 (4H, d, $J_{H\text{-}H}$ = 4.4 Hz), 7.14 – 7.06 (1H, m), 2.52 (1H, dd, $J_{H\text{-}H}$ = 11.0, 5.7 Hz), 1.38 (1H, dd, $J_{H\text{-}H}$ = 16.0, 11.0 Hz), 1.20 (12H, s), 1.19 (6H, s), 1.18 (6H, s), 1.12 (1H, dd, $J_{H\text{-}H}$ = 16.0, 5.7 Hz). **^{13}C NMR** (75 MHz, $CDCl_3$, δ, ppm): 145.5, 128.2, 128.0, 125.0, 83.3, 83.1, 25.1, 24.8, 24.8, 24.6. Cα to boron atom was not observed. White solid. Isolated yield: (315 mg, 88%). Analytical data are in agreement with the literature.[7]

2,2'-(1-(4-(Tert-butyl)phenyl)ethane-1,2-diyl)bis(4,4,5,5-tetramethyl-1,3,2-dioxaborolane) (**2b**)

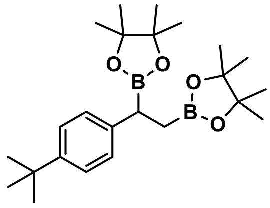

^{1}H NMR (300 MHz, $CDCl_3$, δ, ppm): 7.26 – 7.21 (2H, m), 7.17 – 7.11 (2H, m), 2.48 (1H, dd, $J_{H\text{-}H}$ = 11.0, 5.7 Hz,) 1.38 (1H, dd, $J_{H\text{-}H}$ = 15.5, 4.4 Hz), 1.28 (9H, s), 1.22 – 1.16 (24H, m), 1.10 (1H, dd, $J_{H\text{-}H}$ = 16.0, 5.7 Hz). **^{13}C NMR** (75 MHz, $CDCl_3$, δ, ppm): 147.6, 142.3, 127.6, 125.2, 83.3, 83.1, 34.4, 31.6, 25.1, 24.9, 24.8, 24.6. Cα to boron atom was not observed. White solid. Isolated yield: (356 mg, 86%). Analytical data are in agreement with the literature.[8]

2,2'-(1-(4-Methoxyphenyl)ethane-1,2-diyl)bis(4,4,5,5-tetramethyl-1,3,2-dioxaborolane) (**2c**)

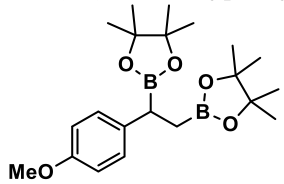

^{1}H NMR (300 MHz, $CDCl_3$, δ, ppm): 7.16 – 7.11 (2H, m), 6.81 – 6.75 (2H, m), 3.76 (3H, s), 2.46 (1H, dd, $J_{H\text{-}H}$ = 10.9, 5.8 Hz), 1.33 (1H, dd, $J_{H\text{-}H}$ = 16.0, 10.9), 1.20 (12H, s), 1.19 (6H, s), 1.17 (6H, s), 1.08 (1H, dd), $J_{H\text{-}H}$ = 16.0, 5.8 Hz). **^{13}C NMR** (75 MHz, $CDCl_3$, δ, ppm): 157.2, 137.5, 128.8, 113.7, 83.2, 83.1, 55.2, 25.0, 24.8, 24.8, 24.6. Cα to boron atom was not observed. White solid. Isolated yield: (306 mg, 79%). Analytical data are in agreement with the literature.[8]

2,2'-(1-(4-Bromophenyl)ethane-1,2-diyl)bis(4,4,5,5-tetramethyl-1,3,2-dioxaborolane) (**2d**)

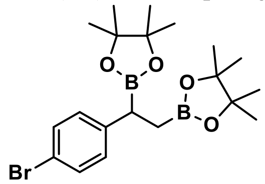

^{1}H NMR (300 MHz, $CDCl_3$, δ, ppm): 7.37 – 7.31 (2H, m), 7.13 – 7.07 (2H, m), 2.48 (1H, dd, $J_{H\text{-}H}$ = 11.0, 6.0 Hz), 1.31 (1H, dd, $J_{H\text{-}H}$ = 16.3, 5.7 Hz), 1.20 (12H, s), 1.18 (6H, s), 1.17 (6H, s), 1.08 (1H, dd, $J_{H\text{-}H}$ = 16.0, 5.9 Hz). **^{13}C NMR** (75 MHz, $CDCl_3$, δ, ppm): 144.6, 131.3, 129.9, 118.7, 83.5, 83.3, 25.1, 24.8, 24. Cα to boron atom was not observed. White solid. Isolated yield: (367 mg, 84%). Analytical data are in agreement with the literature.[9]

2,2'-(1-(2-chlorophenyl)ethane-1,2-diyl)bis(4,4,5,5-tetramethyl-1,3,2-dioxaborolane) (**2e**)

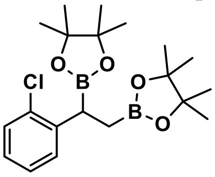

^{1}H NMR (300 MHz, $CDCl_3$, δ, ppm): 7.30 – 7.26 (2H, m), 7.18 – 7.11 (1H, m), 7.08 – 7.01 (1H, m), 2.94 – 2.85 (1H, m), 1.44 – 1.33 (1H, m), 1.22 (6H, s), 1.21 (6H, s), 1.20 (6H, s), 1.19 (6H, s), 1.15 – 1.07 (1H, m). **^{13}C NMR** (101 MHz, $CDCl_3$, δ, ppm): 143.5, 133.9, 129.7, 129.3, 126.8, 126.4, 83.5, 83.2, 25.0, 24.9, 24.8, 24.8. Cα to boron atom was not observed. White solid. Isolated yield: (294 mg, 75%). Analytical data are in agreement with the literature.[8]

2,2'-(1-Phenylpropane-1,2-diyl)bis(4,4,5,5-tetramethyl-1,3,2-dioxaborolane) (**2f**)

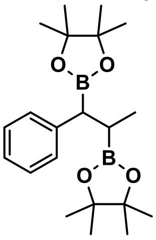

^{1}H NMR (300 MHz, $CDCl_3$, δ, ppm): 7.26 – 7.14 (4H, m), 7.13 – 7.07 (1H, m), 2.22 (1H, d, J_{H-H} = 12.0 Hz), 1.26 (12H, s), 1.17 (6H, s), 1.16 (6H, s), 0.75 (3H, d, J_{H-H} = 7.5 Hz).
^{13}C NMR (75 MHz, $CDCl_3$, δ, ppm): 143.0, 129.1, 128.2, 125.1, 83.2, 83.2, 25.1, 25.0, 24.7, 24.4, 14.7. Cα to boron atom was not observed. Colorless oil. Isolated yield: (293 mg, 79%). Analytical data are in agreement with the literature.[9]

1,2-Diphenyl-1,2-bis(4,4,5,5-tetramethyl-1,3,2-dioxaborolan-2-yl)ethane (**2g**)

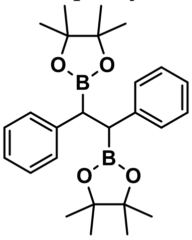

^{1}H NMR (300 MHz, $CDCl_3$, δ, ppm): 7.07 – 6.90 (10H, m), 2.86 (2H, s), 1.22 (12H, s), 1.19 (12H, s).
^{13}C NMR (75 MHz, $CDCl_3$, δ, ppm): 142.3, 128.9, 127.9, 124.9, 83.6, 25.2, 24.3. Cα to boron atom was not observed. White solid. Isolated yield: (339 mg, 78%). Analytical data are in agreement with the literature.[10]

2,2'-(2,3-Dihydro-1H-indene-1,2-diyl)bis(4,4,5,5-tetramethyl-1,3,2-dioxaborolane) (**2h**)

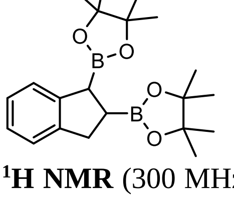

^{1}H NMR (300 MHz, $CDCl_3$, δ, ppm): 7.28 – 7.14 (2H, m), 7.11 – 7.00 (2H, m), 3.13 – 2.94 (2H, m), 2.90 (1H, d, J_{H-H} = 9.0 Hz), 2.06 – 1.93 (1H, m), 1.28 (12H, s), 1.18 (6H, s), 1.10 (6H, s).
^{13}C NMR (151 MHz, $CDCl_3$, δ, ppm): 146.2, 144.5, 125.7, 125.2, 124.1, 124.0, 83.3, 83.2, 35.1, 25.3, 25.0, 24.6, 24.3.Cα to boron atom was not observed. White solid. Isolated yield: (277 mg, 75%). Analytical data are in agreement with the literature.[9]

2,2'-(2-Phenylpropane-1,2-diyl)bis(4,4,5,5-tetramethyl-1,3,2-dioxaborolane) (**2j**)

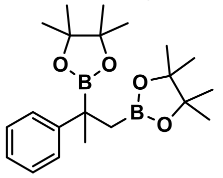

^{1}H NMR (300 MHz, $CDCl_3$, δ, ppm): 7.34 – 7.26 (2H, m), 7.20 – 7.14 (2H, m), 7.05 – 6.98 (1H, m), 1.40 (1H, d, $J_{H\text{-}H}$ = 15.6 Hz), 1.33 (3H, s), 1.14 – 1.11 (17H, m), 1.10 (6H, s), 1.06 (1H, d, $J_{H\text{-}H}$ = 12.6 Hz).
^{13}C NMR (75 MHz, $CDCl_3$, δ, ppm): 149.3, 128.0, 126.6, 124.9, 83.4, 83.1, 25.2, 24.9, 24.7, 24.6. Cα to boron atom was not observed. White solid. Isolated yield: (316 mg, 85%). Analytical data are in agreement with the literature.[7]

2,2'-(2-(p-Tolyl)propane-1,2-diyl)bis(4,4,5,5-tetramethyl-1,3,2-dioxaborolane) (**2k**)

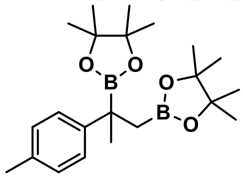

^{1}H NMR (300 MHz, $CDCl_3$, δ, ppm): 7.29 – 7.25 (2H, m), 7.09 – 7.03 (2H, m), 2.29 (3H, s), 1.47 (1H, d, $J_{H\text{-}H}$ = 15.6 Hz), 1.38 (3H, s), 1.22 (12H, s), 1.20 (6H, s), 1.18 (6H, s), 1.10 (1H, d, $J_{H\text{-}H}$ = 15.6 Hz). **^{13}C NMR** (75 MHz, $CDCl_3$, δ, ppm): 146.3, 134.2, 128.8, 126.4, 83.3, 83.1, 25.3, 25.0, 24.9, 24.7, 24.6, 21.0. Cα to boron atom was not observed. White solid. Isolated yield: (320 mg, 83%). Analytical data are in agreement with the literature.[9]

2,2'-(2-(4-Fluorophenyl)propane-1,2-diyl)bis(4,4,5,5-tetramethyl-1,3,2-dioxaborolane) (**2l**)

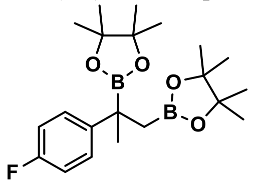

^{1}H NMR (300 MHz, $CDCl_3$, δ, ppm): 7.37 – 7.29 (2H, m), 6.97 – 6.88 (2H, m), 1.42 (1H, d, $J_{H\text{-}H}$ = 15.6 Hz) 1.38 (3H, s), 1.19 (17H, s), 1.18 (6H, s), 1.13 (1H, d, $J_{H\text{-}H}$ = 15.5 Hz). **^{13}C NMR** (75 MHz, $CDCl_3$, δ, ppm): 162.4, 159.2, 144.9, 144.8, 128.1, 128.0, 114.7, 114.4, 83.5, 83.1, 77.6, 76.7, 25.2, 25.0, 24.8, 24.7, 24.6. Cα to boron atom was not observed. Colorless oil. Isolated yield: (324 mg, 83%). Analytical data are in agreement with the literature.[9]

4-(2,3-Bis(4,4,5,5-tetramethyl-1,3,2-dioxaborolan-2-yl)propyl)-2-methoxyphenol (**2m**)

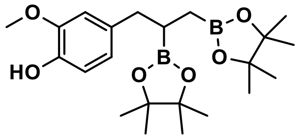

^{1}H NMR (300 MHz, $CDCl_3$, δ, ppm): 6.88 – 6.66 (3H, m), 3.85 (3H, s), 2.73 (1H, dd, $J_{H\text{-}H}$ = 13.5, 7.2 Hz), 2.51 (1H, dd, $J_{H\text{-}H}$ = 13.6, 8.5 Hz), 1.46 – 1.35 (1H, m), 1.22 (12H, s), 1.19 (6H, s), 1.18 (6H, s), 0.82 (2H, d, $J_{H\text{-}H}$ = 7.7 Hz). **^{13}C NMR** (75 MHz, $CDCl_3$, δ, ppm): 146.2, 143.6, 134.5, 121.9, 113.9, 111.8, 83.1, 83.0, 55.9, 39.3, 25.1, 25.0, 25.0, 24.9. Cα to boron atom was not observed. **^{11}B NMR** (128 MHz, $CDCl_3$, δ, ppm): 33.68. **High-resolution MS** (ESI): calcd. for $C_{22}H_{36}B_2O_6$ $[M+Na]^+$ = 441.2596; found 441.2610. **FT-IR** (neat, cm^{-1}): 3422, 2977, 2931, 2162, 1739, 1603, 1514, 1464, 1370, 1311, 1267, 1234, 1139, 1035, 967, 847, 794, 735, 673, 559. **Elemental Anal.** For $C_{22}H_{36}B_2O_6$ (%): calcd.: C, 63.19; H, 8.68; found: 63.25; H, 8.73. White solid. Isolated yield: (380 mg, 91%). *The compound **2m** has been synthesized for the first time.*

2-(2,3-Bis(4,4,5,5-tetramethyl-1,3,2-dioxaborolan-2-yl)propyl)isoindoline-1,3-dione (**2o**)

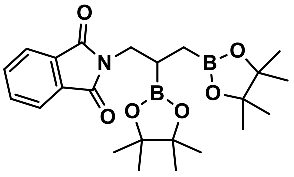

^{1}H NMR (300 MHz, $CDCl_3$, δ, ppm): 7.85 – 7.76 (2H, m), 7.71 – 7.62 (2H, m), 3.86 – 3.67 (2H, m), 1.78 – 1.68 (1H, m), 1.20 (12H, s), 1.18 (12H, s), 0.88 – 0.85 (2H, m). **^{13}C NMR** (75 MHz, $CDCl_3$, δ, ppm): 168.81, 133.65, 132.60, 123.08, 83.37, 83.18, 41.10, 25.09, 25.07, 24.89, 24.81. Cα to boron atom was not observed. **^{11}B NMR** (128 MHz, $CDCl_3$, δ, ppm): 34.0. **High-resolution MS** (ESI): calcd. for $C_{23}H_{33}B_2NO_6$ $[M+Na]^+$ = 464.2392; found 464.2389. **FT-IR** (neat, cm^{-1}): 2977, 2928, 2161, 1979, 1771, 1615, 1467, 1442, 1315, 1272, 1215, 1139, 1083, 967, 847, 715, 670, 529. **Elemental Anal.** For $C_{23}H_{33}B_2NO_6$ (%): calcd.: C, 62.62; H, 7.54; found: 62.68; H, 7.61. Colorless oil. Isolated yield: (265 mg, 60%). *The compound **2o** has been synthesized for the first time*.

2,2',2''-(ethane-1,1,2-triyl)tris(4,4,5,5-tetramethyl-1,3,2-dioxaborolane) (**2q**)

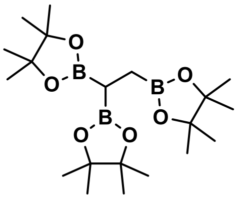

^{1}H NMR (300 MHz, $CDCl_3$, δ, ppm): 1.26 – 1.17 (36H, m), 0.93 (2H, d, $J_{H\text{-}H}$ = 7.6 Hz), 0.76 (1H, t, $J_{H\text{-}H}$ = 7.6 Hz).**^{13}C NMR** (75 MHz, $CDCl_3$, δ, ppm): 82.9, 82.9, 24.9, 24.9, 24.7.White solid. Isolated yield: (350 mg, 86%). Analytical data are in agreement with the literature.[40]

2,2'-(3-Phenylpropane-1,2-diyl)bis(4,4,5,5-tetramethyl-1,3,2-dioxaborolane) (**2r**)

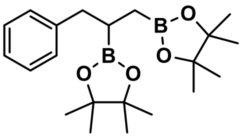

^{1}H NMR (300 MHz, $CDCl_3$, δ, ppm): 7.26 – 7.17 (4H, m), 7.17 – 7.08 (1H, m), 2.80 (1H, dd, $J_{H\text{-}H}$ = 13.4, 7.5 Hz), 2.61 (1H, dd, $J_{H\text{-}H}$ = 13.4, 8.3 Hz), 1.52 – 1.42 (1H, m), 1.22 (12H, s), 1.19 (6H, s), 1.17 (6H, s), 0.83 (2H, d, $J_{H\text{-}H}$ = 7.7 Hz). **^{13}C NMR** (75 MHz, $CDCl_3$, δ, ppm): 142.5, 129.2, 128.0, 125.6, 83.0, 83.0, 39.6, 25.0, 25.0, 24.9, 24.9. Cα to boron atom was not observed. Colorless oil. Isolated yield: (335 mg, 90%). Analytical data are in agreement with the literature.[8]

2,2'-(Octane-1,2-diyl)bis(4,4,5,5-tetramethyl-1,3,2-dioxaborolane) (**2s**)

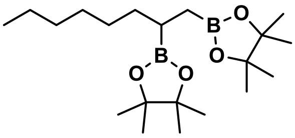

^{1}H NMR (300 MHz, $CDCl_3$, δ, ppm): 1.43 (1H, s), 1.28 – 1.24 (8H, m), 1.23 (12H, s), 1.22 (12H, s), 1.17 – 0.97 (2H, m), 0.89 – 0.79 (5 H, m). **^{13}C NMR** (75 MHz, $CDCl_3$, δ, ppm): 82.9, 82.9, 34.0, 32.0, 29.7, 29.0, 25.1, 25.0, 24.9, 24.9, 22.8, 14.2. Cα to boron atom was not observed. Colorless oil. Isolated yield: (322 mg, 88%). Analytical data are in agreement with the literature.[11]

5,6-Bis(4,4,5,5-Tetramethyl-1,3,2-dioxaborolan-2-yl)hexan-2-one (**2t**)

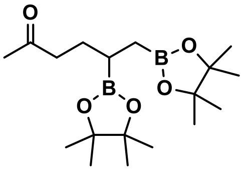

^{1}H NMR (300 MHz, $CDCl_3$, δ, ppm): 2.47 – 2.37 (2H, m), 2.09 (3H, s), 1.77 – 1.52 (2H, m), 1.23 – 1.18 (24H, m), 1.14 – 1.02 (1H, m), 0.88 – 0.72 (2H, m). **^{13}C NMR** (75 MHz, $CDCl_3$, δ, ppm): 209.7, 83.1, 83.0, 43.34, 29.9, 27.9, 25.0, 24.9, 24.8. Cα to boron atom was not observed. Colorless oil. Isolated yield: (299 mg, 85%). Analytical data are in agreement with the literature.[8]

2,2',2''-(2-phenylethane-1,1,2-triyl)tris(4,4,5,5-tetramethyl-1,3,2-dioxaborolane) (**7a**)

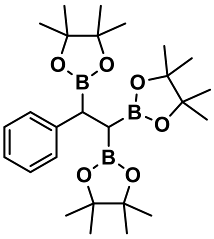

^{1}H NMR (300 MHz, $CDCl_3$, δ, ppm): 7.23 – 7.12 (4H, m), 7.06 – 6.99 (1H, m), 1.24 (6H, s), 1.23 (6H, s), 1.15 (6H, s), 1.13 (6H, s), 0.94 (6H, s), 0.92 (6H, s). **^{13}C NMR** (75 MHz, $CDCl_3$, δ, ppm): 145.45, 128.65, 127.96, 124.8, 83.2, 83.2, 82.8, 25.0, 25.0, 24.8, 24.6, 24.5, 24.4. White solid. Isolated yield: (392 mg, 81%). Analytical data are in agreement with the literature.[22]

2,2',2''-(2-(4-Methoxyphenyl)ethane-1,1,2-triyl)tris(4,4,5,5-tetramethyl-1,3,2-dioxaborolane) (**7b**)

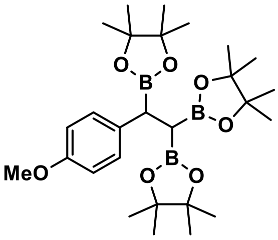

^{1}H NMR (300 MHz, $CDCl_3$, δ, ppm): 7.16 – 7.08 (2H, m), 6.75 – 6.70 (2H, m), 3.73 (3H, s), 2.61 (1H, d, $J_{H\text{-}H}$ = 12.0 Hz), 1.23 (6H, s), 1.22 (6H, s), 1.15 (6H, s), 1.13 (6H, s), 0.96 (6H, s), 0.95 (6H, s). **^{13}C NMR** (75 MHz, $CDCl_3$, δ, ppm): 157.2, 137.6, 129.5, 113.5, 83.1, 83.1, 82.7, 55.3, 25.0, 24.8, 24.6, 24.5, 24.4. Cα to boron atom was not observed. Colorless oil. Isolated yield: (375 mg, 73%). Analytical data are in agreement with the literature.[38]

2,2',2''-(2-(o-tolyl)ethane-1,1,2-triyl)tris(4,4,5,5-tetramethyl-1,3,2-dioxaborolane) (**7c**)

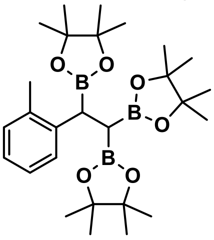

^{1}H NMR (300 MHz, $CDCl_3$, δ, ppm): 7.19 – 7.13 (1H, m), 7.06 – 6.98 (2H, m), 6.95 – 6.88 (1H, m), 2.88 (1H, d, $J_{H\text{-}H}$ = 12.7 Hz), 2.39 (3H, s), 1.50 (1H, d, $J_{H\text{-}H}$ = 12.6 Hz), 1.24 (6H, s), 1.23 (6H, s), 1.14 (6H, s), 1.11 (6H, s), 0.91 (6H, s), 0.88 (6H, s). **^{13}C NMR** (75 MHz, $CDCl_3$, δ, ppm): 143.9, 136.5, 129.6, 125.6, 124.5, 83.1, 83.1, 82.7, 24.9, 24.9, 24.9, 24.5, 24.4, 20.7. Cα to boron atom was not

observed. **^{11}B NMR** (128 MHz, $CDCl_3$, δ, ppm): 34.08. **High-resolution MS** (ESI): calcd. for $C_{27}H_{45}B_3O_6$ $[M+Na]^+$ = 521.3393; found 521.3399. **FT-IR** (neat, cm^{-1}): 2975, 2929, 1620, 1465, 1371, 1348, 1328, 1307, 1264, 1132, 1102, 967, 893, 846, 734, 562. **Elemental Anal.** For $C_{27}H_{45}B_3O_6$ (%): calcd.: C, 65.11; H, 9.11; found: 65.13; H, 9.10. Yellow oil. Isolated yield: (354 mg, 71%). *The compound **7c** has been synthesized for the first time*.

2,2',2''-(2-(4-bromophenyl)ethane-1,1,2-triyl)tris(4,4,5,5-tetramethyl-1,3,2-dioxaborolane) (**7d**)

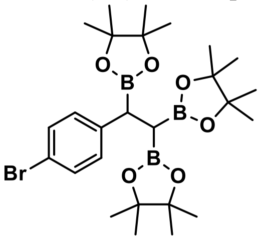

^{1}H NMR (300 MHz, $CDCl_3$, δ, ppm): 7.31 – 7.26 (2H, m), 7.12 – 7.06 (2H, m), 2.63 (1H, d, J_{H-H} = 12.7 Hz), 1.41 (1H, d, J_{H-H} = 6.0 Hz), 1.23 (6H, s), 1.22 (6H, s), 1.14 (6H, s), 1.13 (6H, s), 0.98 (6H, s), 0.94 (6H, s). **^{13}C NMR** (75 MHz, $CDCl_3$, δ, ppm): 144.7, 131.0, 130.4, 118.5, 83.3, 83.3, 82.9, 25.2, 25.0, 25.0, 24.8, 24.6, 24.5, 24.4. Cα to boron atom was not observed. Colorless oil. Isolated yield: (444 mg, 79%). Analytical data are in agreement with the literature.[38]

2,2',2''-(Octane-1,1,2-triyl)tris(4,4,5,5-tetramethyl-1,3,2-dioxaborolane) (**7e**)

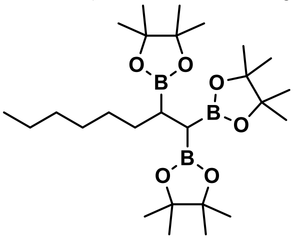

^{1}H NMR (400 MHz, $CDCl_3$, δ, ppm): 1.47 – 1.33 (2H, m), 1.33 – 1.15 (43H, m), 0.92 – 0.81 (4H, m). **^{13}C NMR** (75 MHz, $CDCl_3$, δ, ppm): 82.9, 82.9, 82.7, 33.5, 32.0, 29.8, 28.8, 25.2, 25.0, 24.9, 24.8, 24.8, 22.8, 14.2. Colorless oil. Isolated yield: (433 mg, 88%). Analytical data are in agreement with the literature.[39]

4,4,5,5-Tetramethyl-2-phenethyl-1,3,2-dioxaborolane (**3a**)

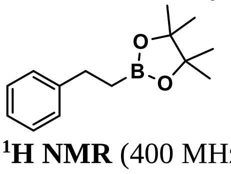

^{1}H NMR (400 MHz, $CDCl_3$, δ, ppm): 7.28 – 7.20 (4H, m), 7.19 – 7.12 (1H, m), 2.75 (2H, t, J_{H-H} = 8.0 Hz) 1.22 (12H, s), 1.15 (2H, t, J_{H-H} = 8.0 Hz). **^{13}C NMR** (75 MHz, $CDCl_3$, δ, ppm): 144.5, 128.3, 128.1, 125.6, 83.2, 30.1, 24.9. Cα to boron atom was not observed. White solid. Isolated yield: (209 mg, 90%). Analytical data are in agreement with the literature.[12]

4,4,5,5-Tetramethyl-2-(2,4,6-trimethylphenethyl)-1,3,2-dioxaborolane (**3u**)

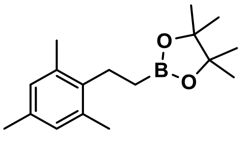

^{1}H NMR (300 MHz, $CDCl_3$, δ, ppm): 6.86 (2H, s), 2.78 – 2.66 (2H, m), 2.34 (6H, s), 2.28 (3H, s), 1.31 (12H, s), 1.05 – 0.97 (2H, m). **^{13}C NMR** (75 MHz, $CDCl_3$, δ, ppm): 138.6, 135.7, 134.7, 128.9,

83.2, 25.0, 23.4, 20.9, 19.7. Cα to boron atom was not observed. Colorless oil. Isolated yield: (236 mg, 82%). Analytical data are in agreement with the literature.[13]

2-(2-Methoxyphenethyl)-4,4,5,5-tetramethyl-1,3,2-dioxaborolane (**3v**)

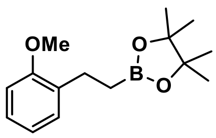

^{1}H NMR (300 MHz, $CDCl_3$, δ, ppm): 7.21 – 7.12 (2H, m), 6.90 – 6.80 (2H, m), 3.82 (3H, s), 2.74 (2H, t, J_{H-H} = 8.0 Hz), 1.24 (12H, s), 1.13 (2H, t, J_{H-H} = 8.0 Hz). **^{13}C NMR** (75 MHz, $CDCl_3$, δ, ppm): 157.5, 132.9, 129.2, 126.8, 120.4, 110.2, 83.1, 55.3, 25.0, 24.5. Colorless oil. Isolated yield: (235 mg, 90%). Analytical data are in agreement with the literature.[12]

N,N-Dimethyl-4-(2-(4,4,5,5-tetramethyl-1,3,2-dioxaborolan-2-yl)ethyl)aniline (**3w**)

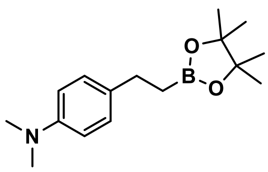

^{1}H NMR (300 MHz, $CDCl_3$, δ, ppm): 7.16 – 7.07 (2H, m), 6.77 – 6.69 (2H, m), 2.91 (6H, s), 2.66 (2H, t, J_{H-H} = 8.2 Hz), 1.23 (12H, s), 1.11 (2H, t, J_{H-H} = 8.2 Hz). **^{13}C NMR** (75 MHz, $CDCl_3$, δ, ppm): 149.0, 133.3, 128.7, 113.3, 83.1, 41.2, 29.0, 25.0.Cα to boron atom was not observed. Yellow oil. Isolated yield: (190 mg, 69%). Analytical data are in agreement with the literature.[14]

2-(4-Fluorophenethyl)-4,4,5,5-tetramethyl-1,3,2-dioxaborolane (**3x**)

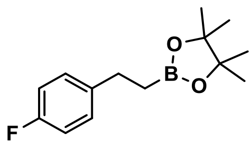

^{1}H NMR (300 MHz, $CDCl_3$, δ, ppm): 7.19 – 7.12 (2H, m), 6.97 – 6.89 (2H, m), 2.72 (2H, t, J_{H-H} = 8.0 Hz), 1.21 (12H, s), 1.12 (2H, t, J_{H-H} = 8.0 Hz). **^{13}C NMR** (151 MHz, $CDCl_3$, δ, ppm): 161.2 (d, J^1_{C-F} = 242.5 Hz), 140.1 (d, J^2_{C-F} = 3.3 Hz), 129.4 (d, J^3_{C-F} = 7.6 Hz), 114.9 (d, J^4_{C-F} = 21.0 Hz), 83.1, 29.2, 24.8. Cα to boron atom was not observed. Colorless oil. Isolated yield: (210 mg, 84%). Analytical data are in agreement with the literature.[15]

2-(4-Bromophenethyl)-4,4,5,5-tetramethyl-1,3,2-dioxaborolane (**3y**)

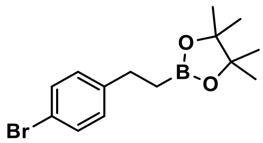

^{1}H NMR (300 MHz, $CDCl_3$, δ, ppm): 7.39 – 7.33 (2H, m), 7.11 – 7.05 (2H, m), 2.69 (2H, t, J_{H-H} = 8.0 Hz), 1.21 (12H, s), 1.11 (2H, t, J_{H-H} = 8.0 Hz). **^{13}C NMR** (151 MHz, $CDCl_3$, δ, ppm): 143.5, 131.3, 129.9, 119.3, 83.3, 29.5, 24.9. Cα to boron atom was not observed. Colorless oil. Isolated yield: (267 mg, 86%). Analytical data are in agreement with the literature.[12]

Methyl 4-[2-(4,4,5,5-tetramethyl-1,3,2-dioxaborolan-2-yl)ethyl]benzoate (**3z**)

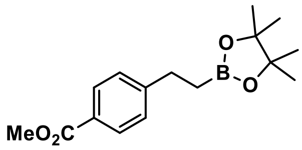

^{1}H NMR (300 MHz, $CDCl_3$, δ, ppm): 7.97 – 7.88 (2H, m), 7.30 – 7.26 (2H, m), 3.89 (3H, s), 2.80 (2H, t, J_{H-H} = 8.0 Hz), 1.21 (12H, s), 1.15 (2H, t, J_{H-H} = 8.2 Hz). **^{13}C NMR** (151 MHz, $CDCl_3$, δ, ppm): 167.4, 150.1, 129.7, 128.2, 127.7, 83.4, 52.1, 30.2, 24.9. Cα to boron atom was not observed. Colorless oil. Isolated yield: (209 mg, 72%). Analytical data are in agreement with the literature.[16]

4,4,5,5-Tetramethyl-2-[3-(2-methylphenyl)propyl]-1,3,2-dioxaborolane (**3aa**)

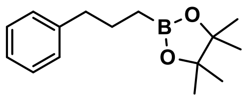

^{1}H NMR (300 MHz, $CDCl_3$, δ, ppm): 7.30 – 7.24 (2H, m), 7.23 – 7.12 (3H, m), 2.62 (2H, t, J_{H-H} = 7.8 Hz), 1.75 (2H, p, J_{H-H} = 7.8 Hz), 1.25 (12H, s), 0.84 (2H, t, J_{H-H} = 7.9 Hz). **^{13}C NMR** (75 MHz, $CDCl_3$, δ, ppm): 142.8, 128.7, 128.3, 125.7, 83.1, 38.7, 26.2, 25.0. Cα to boron atom was not observed. Colorless oil. Isolated yield: (219 mg, 89%). Analytical data are in agreement with the literature.[13]

2-(2-(Naphthalen-2-yl)ethyl)-4,4,5,5-tetramethyl-1,3,2-dioxaborolane (**3ab**)

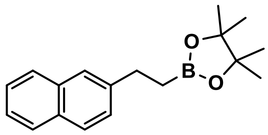

^{1}H NMR (300 MHz, $CDCl_3$, δ, ppm): 7.84 – 7.73 (3H, m), 7.66 (1H, s), 7.48 – 7.35 (3H, m), 2.94 (2H, t, J_{H-H} = 8.0 Hz), 1.28 (2H, t, J_{H-H} = 8.0 Hz), 1.23 (12H, s). **^{13}C NMR** (151 MHz, $CDCl_3$, δ, ppm): 142.1, 133.8, 132.0, 127.8, 127.7, 127.5, 127.4, 125.8, 125.8, 125.0, 83.3, 30.3, 24.9. Cα to boron atom was not observed. White solid. Isolated yield: (240 mg, 85%). Analytical data are in agreement with the literature.[12]

4,4,5,5-Tetramethyl-2-(2-phenylpropyl)-1,3,2-dioxaborolane (**3j**)

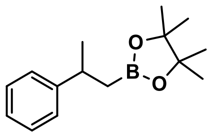

^{1}H NMR (300 MHz, $CDCl_3$, δ, ppm): 7.30 – 7.24 (4H, m), 7.19 – 7.13 (1H, m), 3.12 – 2.98 (1H, m), 1.30 (3H, d, J_{H-H} = 6.9 Hz), 1.22 – 1.13 (14H, m). **^{13}C NMR** (75 MHz, $CDCl_3$, δ, ppm): 149.4, 128.3, 126.8, 125.8, 83.1, 35.9, 25.0, 24.9, 24.8. Cα to boron atom was not observed. Colorless oil. Isolated yield: (182 mg, 76%). Analytical data are in agreement with the literature.[13]

4,4,5,5-Tetramethyl-2-(2-(p-tolyl)propyl)-1,3,2-dioxaborolane (**3k**)

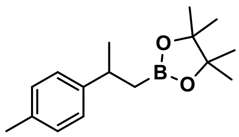

^{1}H NMR (300 MHz, $CDCl_3$, δ, ppm): 7.18 (2H, d, J_{H-H} = 8.3 Hz), 7.11 (2H, d, J_{H-H} = 8.0 Hz), 3.14 – 2.98 (1H, m), 2.34 (3H, s), 1.31 (3H, d, J_{H-H} = 7.0 Hz), 1.28 – 1.12 (14H, m). **^{13}C NMR** (75 MHz, $CDCl_3$, δ, ppm): 146.3, 135.0, 128.9, 126.5, 83.0, 35.4, 25.0, 24.9, 24.8, 21.0. Cα to boron atom was

not observed. Colorless oil. Isolated yield: (200 mg, 77%). Analytical data are in agreement with the literature.[17]

2-(2-(4-Chlorophenyl)propyl)-4,4,5,5-tetramethyl-1,3,2-dioxaborolane (**3ac**)

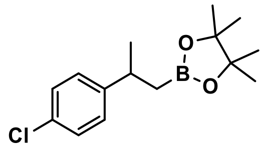

^{1}H NMR (300 MHz, $CDCl_3$, δ, ppm): 7.26 – 7.10 (4H, m), 3.10 – 2.92 (1H, m), 1.24 (3H, d, $J_{H\text{-}H}$ = 7.0 Hz), 1.16 (12H, s), 1.12 (2H, d, $J_{H\text{-}H}$ = 8.0 Hz). **^{13}C NMR** (75 MHz, $CDCl_3$, δ, ppm): 147.8, 131.3, 128.4, 128.2, 83.2, 35.4, 25.0, 24.9, 24.8. Colorless oil. Isolated yield: (227 mg, 81%). Analytical data are in agreement with the literature.[17]

(E)-4,4,5,5-Tetramethyl-2-styryl-1,3,2-dioxaborolane (**8a**)

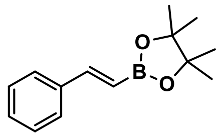

^{1}H NMR (300 MHz, $CDCl_3$, δ, ppm): 7.51 – 7.46 (2H, m), 7.40 (1H, d, $J_{H\text{-}H}$ = 18.3 Hz), 7.36 – 7.28 (3H, m), 6.17 (1H, d, $J_{H\text{-}H}$ = 18.3 Hz), 1.32 (12H, s). **^{13}C NMR** (75 MHz, $CDCl_3$, δ, ppm): 149.7, 137.6, 129.0, 128.7, 127.2, 83.5, 25.0. Cα to boron atom was not observed. Colorless oil. Isolated yield: (200 mg, 87%). Analytical data are in agreement with the literature.[13]

(E)-2-(2-Methoxystyryl)-4,4,5,5-tetramethyl-1,3,2-dioxaborolane (**8h**)

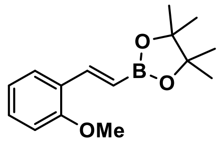

^{1}H NMR (300 MHz, $CDCl_3$, δ, ppm): 7.77 (1H, d, $J_{H\text{-}H}$ = 18.6 Hz), 7.58 – 7.52 (1H, m), 7.30 – 7.23 (1H, m), 6.96 – 6.84 (2H, m), 6.18 (1H, d, $J_{H\text{-}H}$ = 18.6 Hz), 3.85 (3H, s), 1.31 (12H, s). **^{13}C NMR** (75 MHz, $CDCl_3$, δ, ppm): 157.5, 144.2, 130.1, 127.2, 126.7, 120.7, 111.0, 83.3, 55.5, 25.0. Cα to boron atom was not observed. Colorless oil. Isolated yield: (200 mg, 77%). Analytical data are in agreement with the literature.[18]

(E)-4-(2-(4,4,5,5-Tetramethyl-1,3,2-dioxaborolan-2-yl)vinyl)benzonitrile (**8i**)

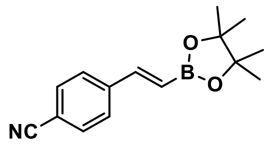

^{1}H NMR (300 MHz, $CDCl_3$, δ, ppm): 7.65 – 7.60 (2H, m), 7.57 – 7.52 (2H, m), 7.36 (1H, d, $J_{H\text{-}H}$ = 18.4 Hz), 6.28 (1H, d, $J_{H\text{-}H}$ = 18.4 Hz), 1.32 (12H, s). **^{13}C NMR** (75 MHz, $CDCl_3$, δ, ppm): 147.3, 141.8, 132.6, 127.6, 119.0, 112.1, 83.9, 25.0. Cα to boron atom was not observed. White solid. Isolated yield: (209 mg, 82%). Analytical data are in agreement with the literature.[13]

(E)-2-(3,5-Bis(trifluoromethyl)styryl)-4,4,5,5-tetramethyl-1,3,2-dioxaborolane (**8j**)

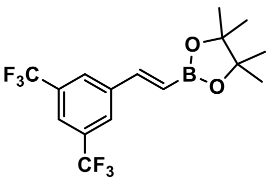

^{1}H NMR (300 MHz, $CDCl_3$, δ, ppm): 7.88 (2H, s), 7.78 (1H, s), 7.41 (1H, d, $J_{H\text{-}H}$ = 18.4 Hz,) 6.31 (1H, d, $J_{H\text{-}H}$ = 18.4 Hz), 1.32 (12H, s). **^{13}C NMR** (75 MHz, $CDCl_3$, δ, ppm): 146.0, 139.7, 132.8, 132.4, 132.0, 131.5, 126.9, 126.9, 125.2, 122.3, 122.2, 122.2, 122.1, 121.6, 84.0, 25.0. Cα to boron atom was not observed. Colorless oil. Isolated yield: (307 mg, 84%). Analytical data are in agreement with the literature.[19]

(E)-4,4,5,5-Tetramethyl-2-(2-(thiophen-3-yl)vinyl)-1,3,2-dioxaborolane (**8k**)

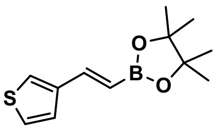

^{1}H NMR (300 MHz, $CDCl_3$, δ, ppm): 7.35 (1H, m, $J_{H\text{-}H}$ = 18.4 Hz), 7.29 – 7.22 (3H, m), 5.92 (1H, d, $J_{H\text{-}H}$ = 18.4 Hz) 1.28 (12H, s). **^{13}C NMR** (75 MHz, $CDCl_3$, δ, ppm): 143.3, 141.4, 126.3, 125.2, 125.0, 83.5, 24.9. Cα to boron atom was not observed. Colorless oil. Isolated yield: (198 mg, 84%). Analytical data are in agreement with the literature.[13]

(Z)-2-(1,2-Diphenylvinyl)-4,4,5,5-tetramethyl-1,3,2-dioxaborolane (**8f**)

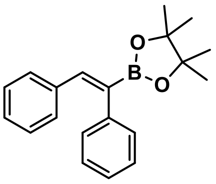

^{1}H NMR (300 MHz, $CDCl_3$, δ, ppm): 7.38 (1H, s), 7.26 – 7.21 (2H, m), 7.19 – 7.14 (3H, m), 7.10 – 7.01 (5H, m), 1.28 (12H, s). **^{13}C NMR** (75 MHz, $CDCl_3$, δ, ppm): 143.3, 140.5, 137.0, 130.0, 128.9, 128.3, 127.9, 127.7, 126.3, 83.8, 24.9. Cα to boron atom was not observed. White solid. Isolated yield: (263 mg, 86%). Analytical data are in agreement with the literature.[13]

(Z)-4,4,5,5-tetramethyl-2-(1-phenylprop-1-en-1-yl)-1,3,2-dioxaborolane and *(Z)-4,4,5,5-tetramethyl-2-(1-phenylprop-1-en-2-yl)-1,3,2-dioxaborolane* (**8l**)

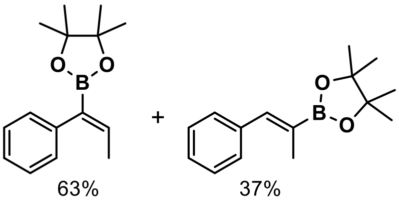

^{1}H NMR (both isomers) (300 MHz, $CDCl_3$, δ, ppm): 7.42 – 7.29 (4H, m), 7.28 – 7.13 (5H, m), 6.79 – 6.68 (1H, m), 2.01 (2H, d, $J_{H\text{-}H}$ = 1.8 Hz), 1.78 (3H, d, $J_{H\text{-}H}$ = 7.0 Hz), 1.33 (7H, s), 1.28 (12H, s).. **^{13}C NMR** (75 MHz, $CDCl_3$, δ, ppm): 142.8, 142.5, 139.9, 138.1, 129.5, 129.2, 128.2, 127.9, 127.2, 126.0, 83.6, 83.6, 25.0, 24.9, 16.1, 16.0. Cα to boron atom was not observed. Colorless oil. Isolated yield of both isomers: (202 mg, 83%). Analytical data are in agreement with the literature.[19]

(E)-4,4,5,5-Tetramethyl-2-(oct-1-en-1-yl)-1,3,2-dioxaborolane (**8e**)

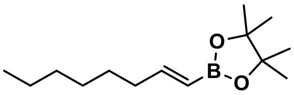

^{1}H NMR (300 MHz, $CDCl_3$, δ, ppm): 6.73 – 6.54 (1H, m), 5.50 – 5.33 (1H, m), 2.18 – 2.10 (2H, m), 1.44 – 1.36 (2H, m), 1.26 (18H, m), 0.90 – 0.85 (3H, m). **^{13}C NMR** (75 MHz, $CDCl_3$, δ, ppm): 155.0, 83.1, 36.0, 31.9, 29.1, 28.3, 24.9, 22.7, 14.2. Cα to boron atom was not observed. Colorless oil. Isolated yield: (202 mg, 85%). Analytical data are in agreement with the literature.[13]

(E)-Triphenyl(2-(4,4,5,5-tetramethyl-1,3,2-dioxaborolan-2-yl)vinyl)silane (**8m**)

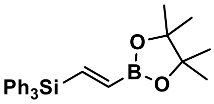

^{1}H NMR (300 MHz, $CDCl_3$, δ, ppm): 7.55 (1H, d, $J_{H\text{-}H}$ = 21.6 Hz), 7.50 – 7.45 (6H, m), 7.39 – 7.26 (10H, m), 6.34 (1H, d, $J_{H\text{-}H}$ = 21.7 Hz), 1.22 (12H, s). **^{13}C NMR** (75 MHz, $CDCl_3$, δ, ppm): 150.7, 136.2, 134.0, 129.7, 128.0, 83.6, 25.0. Cα to boron atom was not observed. White solid. Isolated yield: (334 mg, 81%). Analytical data are in agreement with the literature.[20]

2-phenylpropane-1,2-diol (**2ja**)

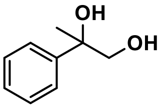

^{1}H NMR (300 MHz, $CDCl_3$, δ, ppm): 7.46 – 7.37 (2H, m), 7.37 – 7.30 (1H, m), 3.76 (1H, d, $J_{H\text{-}H}$ = 11.1 Hz), 3.59 (1H, d, $J_{H\text{-}H}$ = 11.1 Hz), 2.16 (2H, s), 1.50 (3H, s). **^{13}C NMR** (75 MHz, $CDCl_3$, δ, ppm): 145.1, 128.6, 127.3, 125.2, 75.0, 71.2, 26.2. White solid. Isolated yield: (138 mg, 92%). Analytical data are in agreement with the literature.[21]

(E)-4-(4-methylstyryl)benzonitrile (**6ia**)

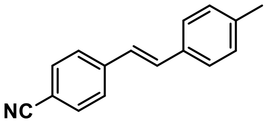

^{1}H NMR (300 MHz, $CDCl_3$, δ, ppm): 7.63 – 7.54 (4H, m), 7.43 (2H, d, $J_{H\text{-}H}$ = 8.3 Hz), 7.23 – 7.15 (3H, m), 7.04 (1H, d, $J_{H\text{-}H}$ = 16.3 Hz), 2.38 (3H, s). **^{13}C NMR** (75 MHz, $CDCl_3$, δ, ppm): 142.2, 138.9, 133.7, 132.6, 132.5, 129.7, 127.0, 126.9, 125.9, 119.3, 110.5, 21.5. White solid. Isolated yield: (182 mg, 83%). Analytical data are in agreement with the literature.[23]

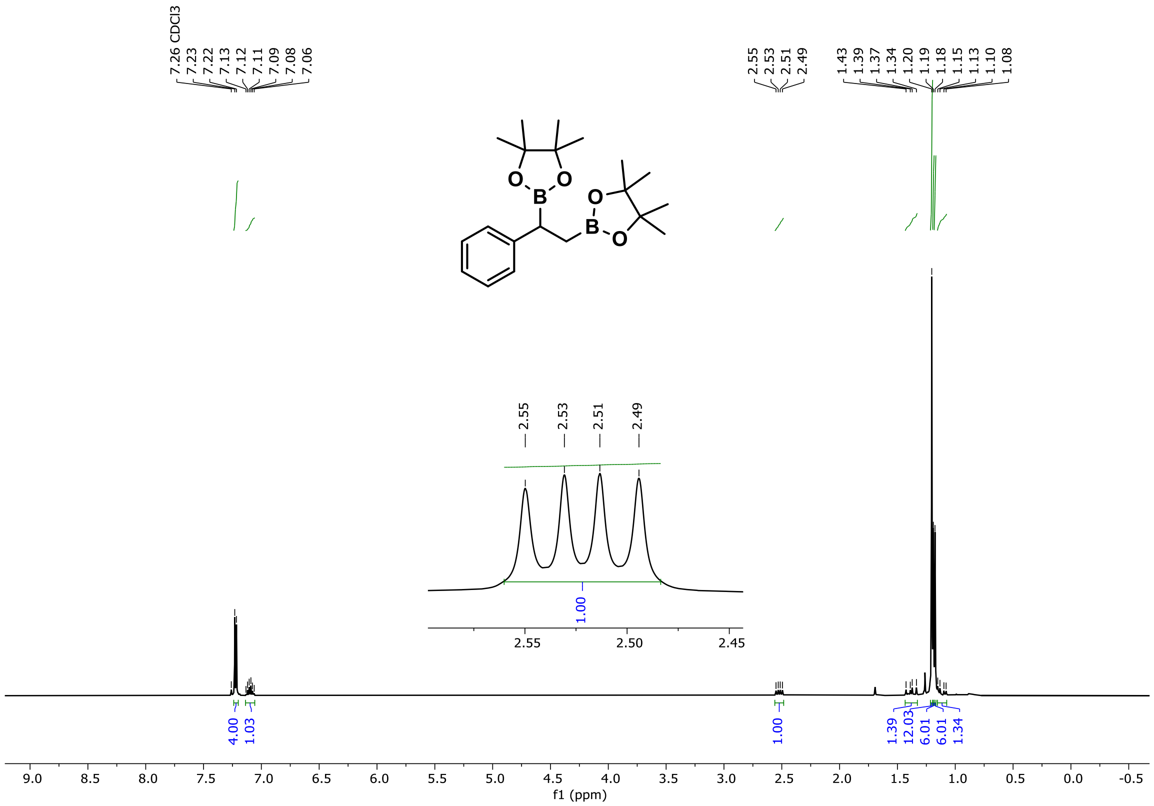

Figure S5. ^{1}H NMR spectrum of *2,2'-(1-phenylethane-1,2-diyl)bis(4,4,5,5-tetramethyl-1,3,2-dioxaborolane)* (**2a**).

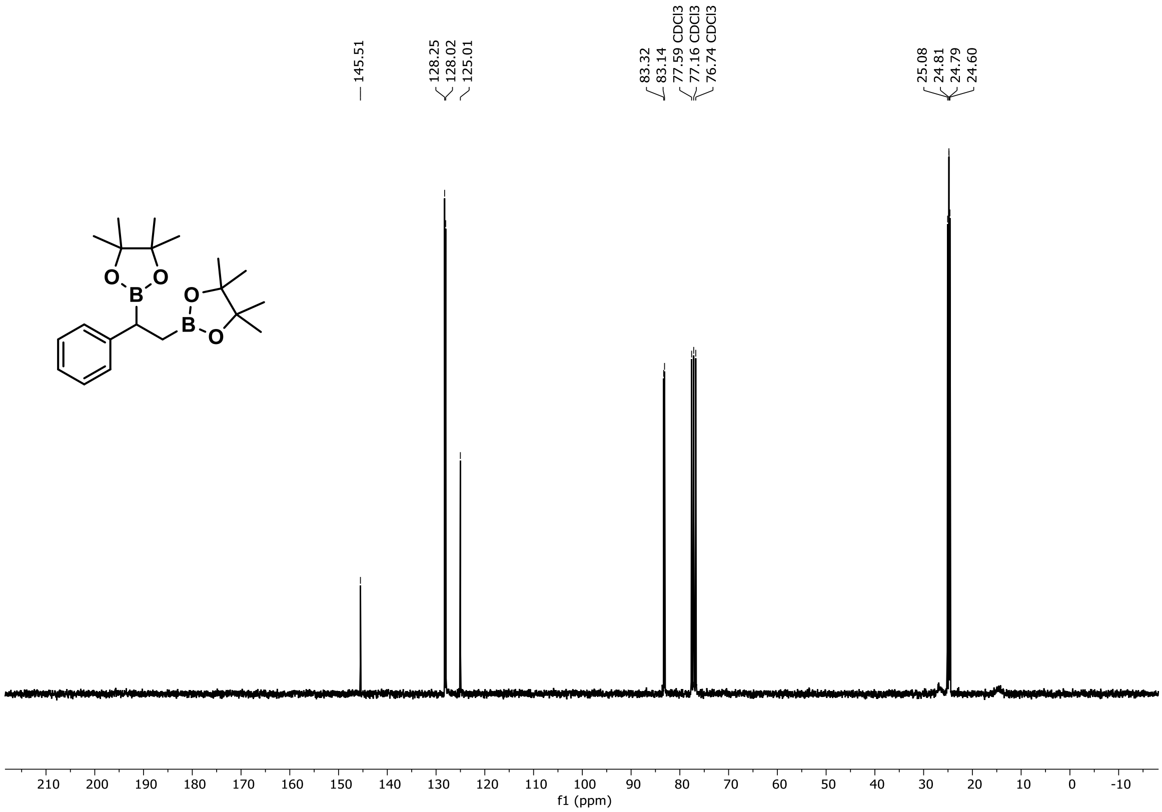

Figure S6. ^{13}C NMR spectrum of *2,2'-(1-phenylethane-1,2-diyl)bis(4,4,5,5-tetramethyl-1,3,2-dioxaborolane)* (**2a**).

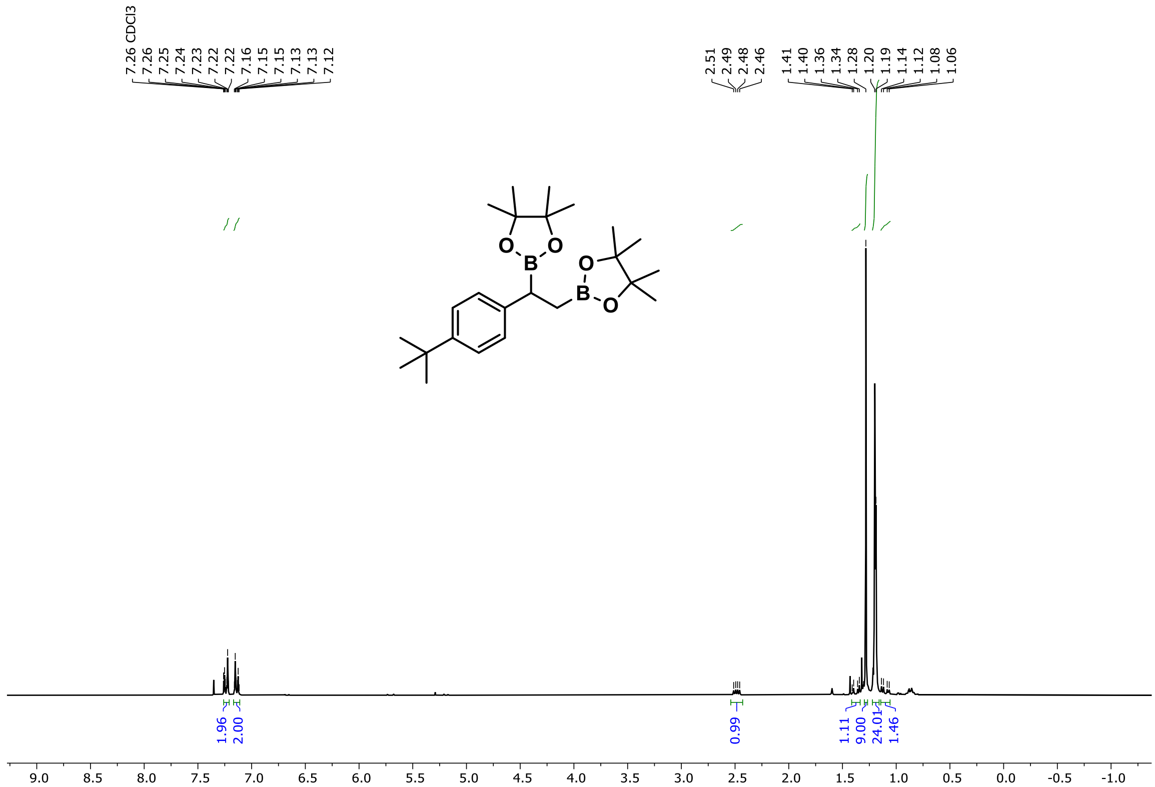

Figure S7. ^{1}H NMR spectrum of *2,2'-(1-(4-(tert-butyl)phenyl)ethane-1,2-diyl)bis(4,4,5,5-tetramethyl-1,3,2-dioxaborolane)* (**2b**).

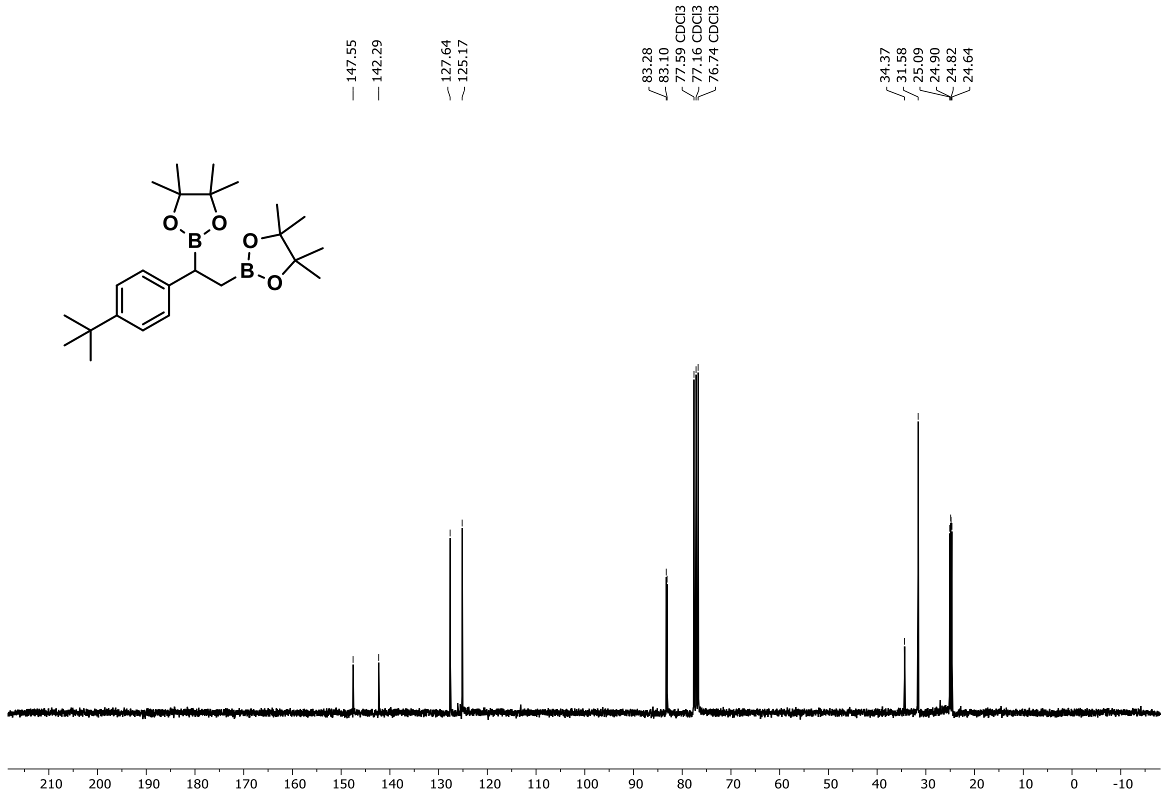

Figure S8. ^{13}C NMR spectrum of *2,2'-(1-(4-(tert-butyl)phenyl)ethane-1,2-diyl)bis(4,4,5,5-tetramethyl-1,3,2-dioxaborolane)* (**2b**).

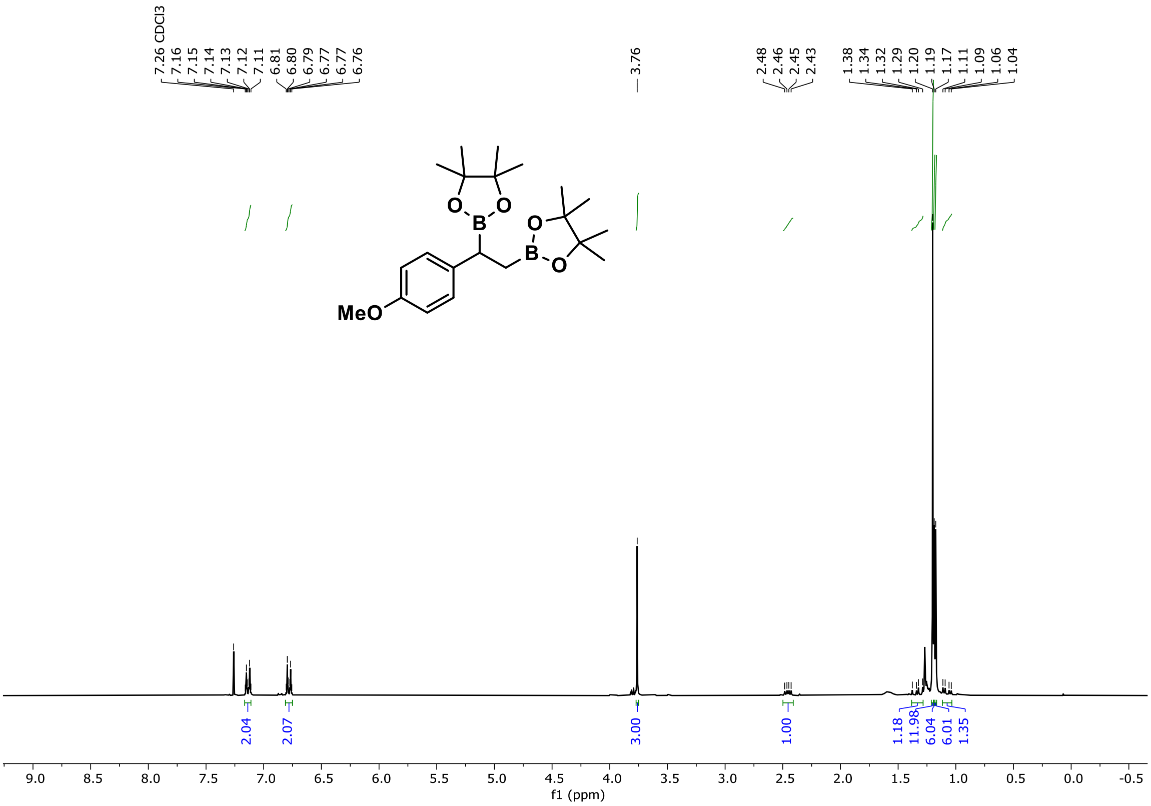

Figure S9. ^{1}H NMR spectrum of *2,2'-(1-(4-methoxyphenyl)ethane-1,2-diyl)bis(4,4,5,5-tetramethyl-1,3,2-dioxaborolane)* (**2c**).

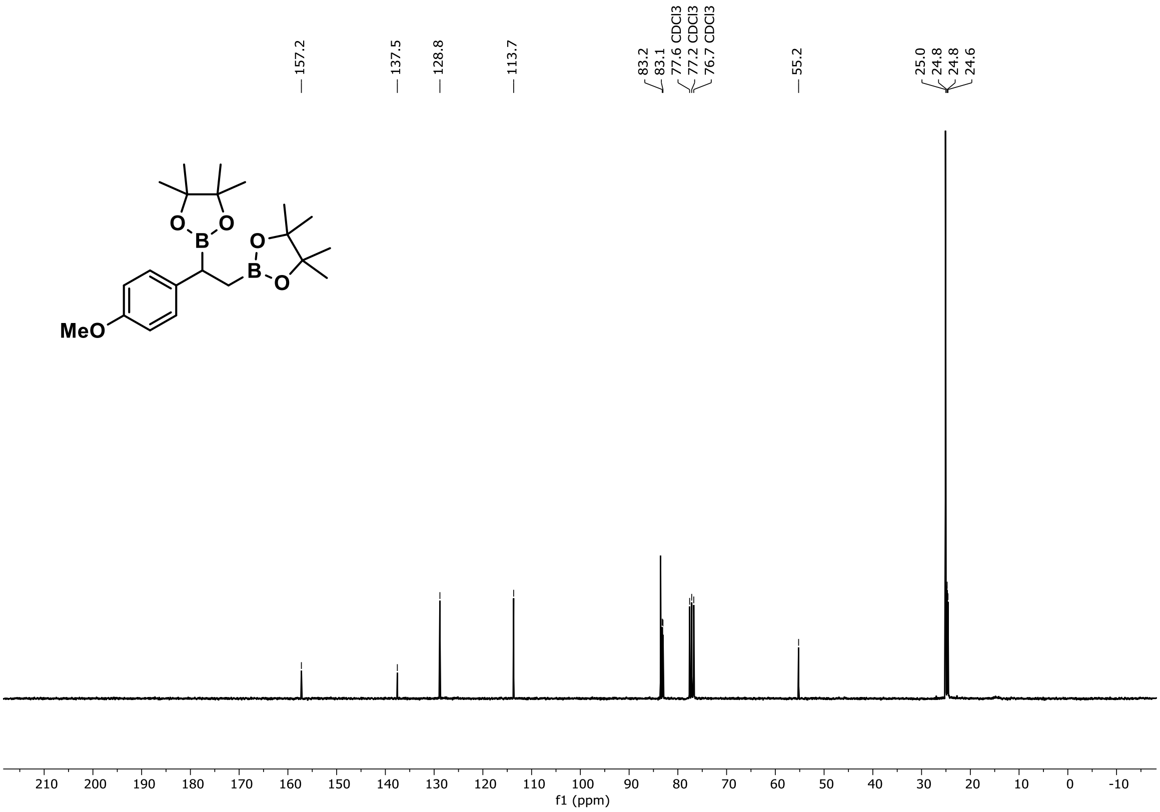

Figure S10. ^{13}C NMR spectrum of *2,2'-(1-(4-methoxyphenyl)ethane-1,2-diyl)bis(4,4,5,5-tetramethyl-1,3,2-dioxaborolane)* (**2c**).

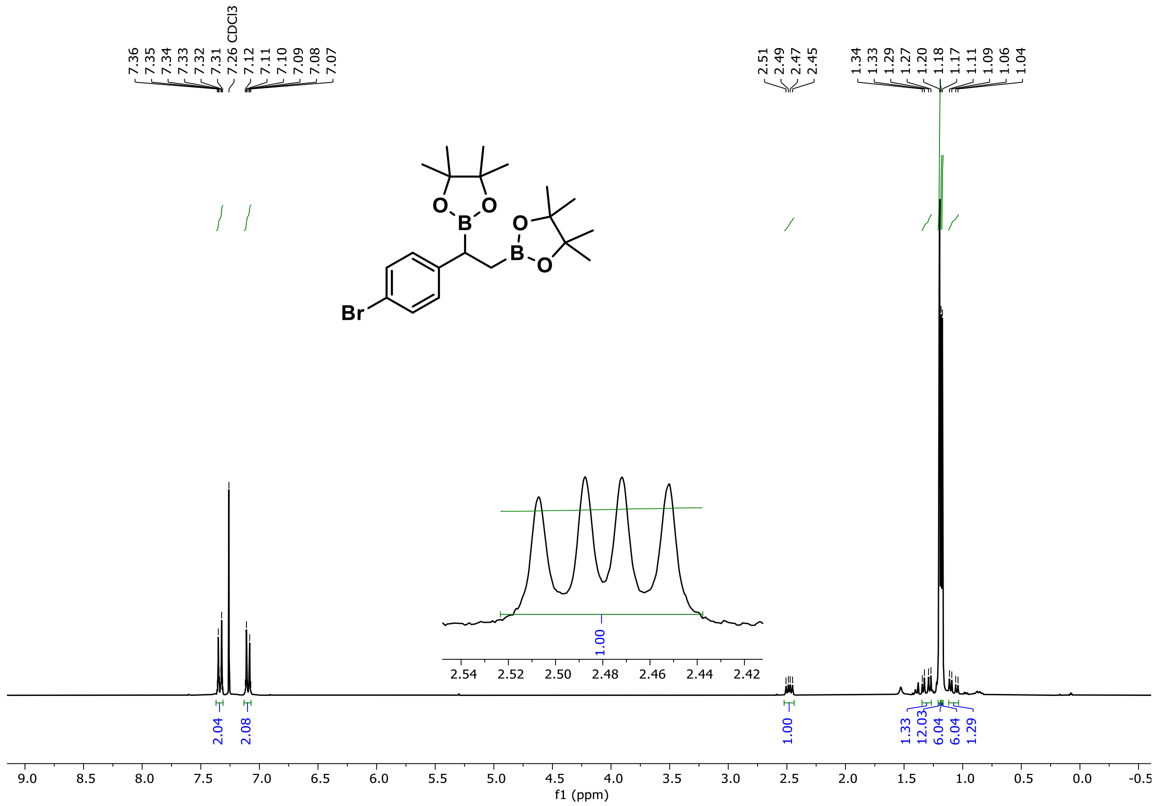

Figure S11. ^{1}H NMR spectrum of *2,2'-(1-(4-bromophenyl)ethane-1,2-diyl)bis(4,4,5,5-tetramethyl-1,3,2-dioxaborolane)* (**2d**).

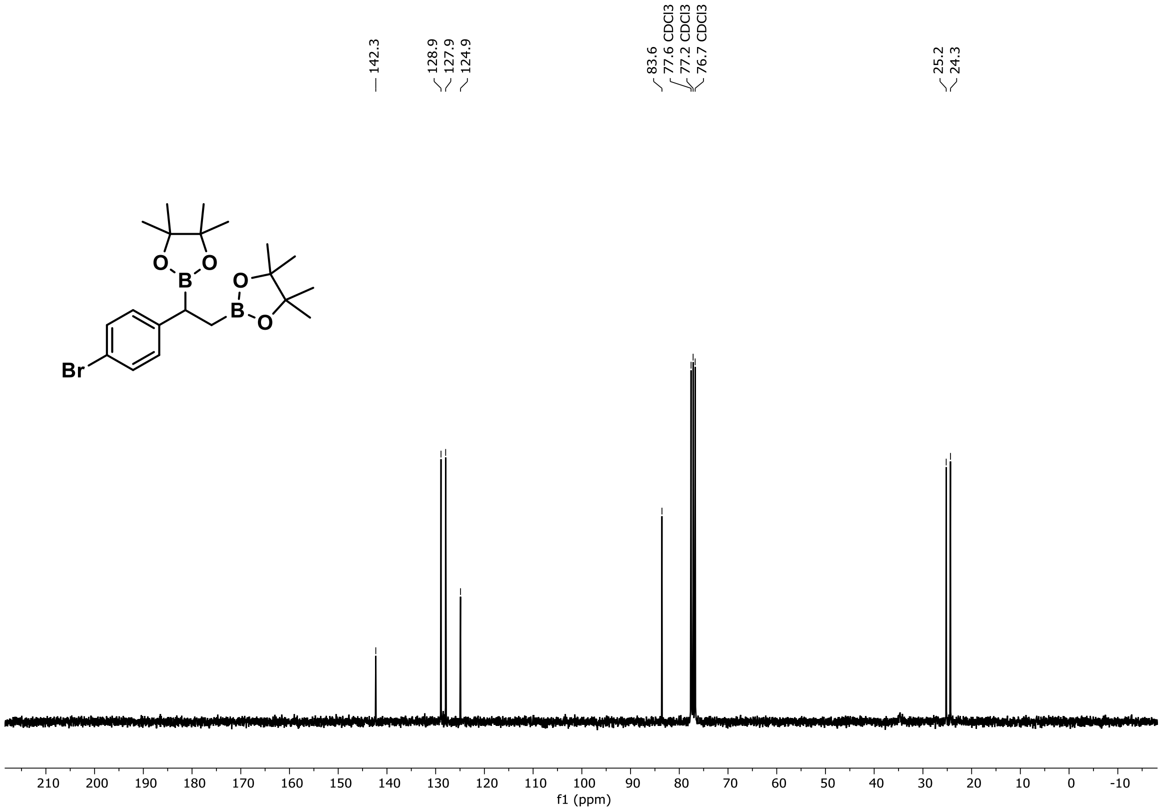

Figure S12. ^{13}C NMR spectrum of *2,2'-(1-(4-bromophenyl)ethane-1,2-diyl)bis(4,4,5,5-tetramethyl-1,3,2-dioxaborolane)* (**2d**).

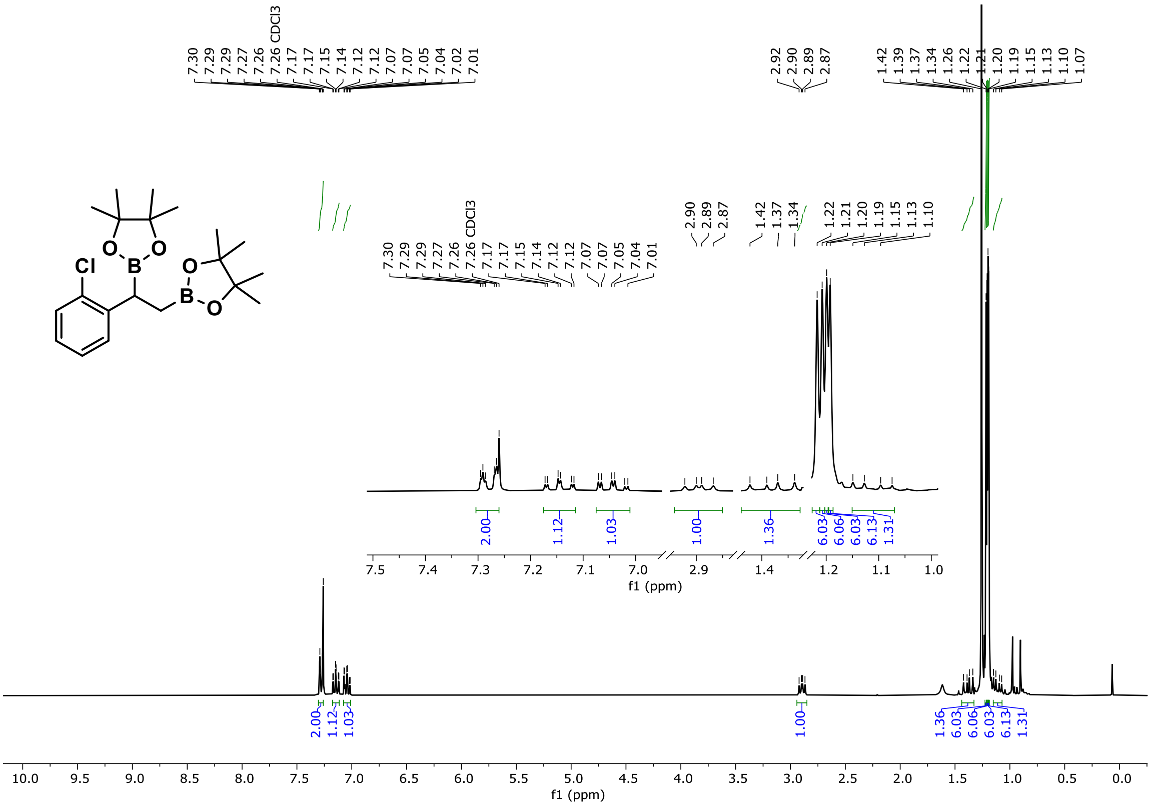

Figure S13. ^{1}H NMR spectrum of *2,2'-(1-(2-chlorophenyl)ethane-1,2-diyl)bis(4,4,5,5-tetramethyl-1,3,2-dioxaborolane)* (**2e**).

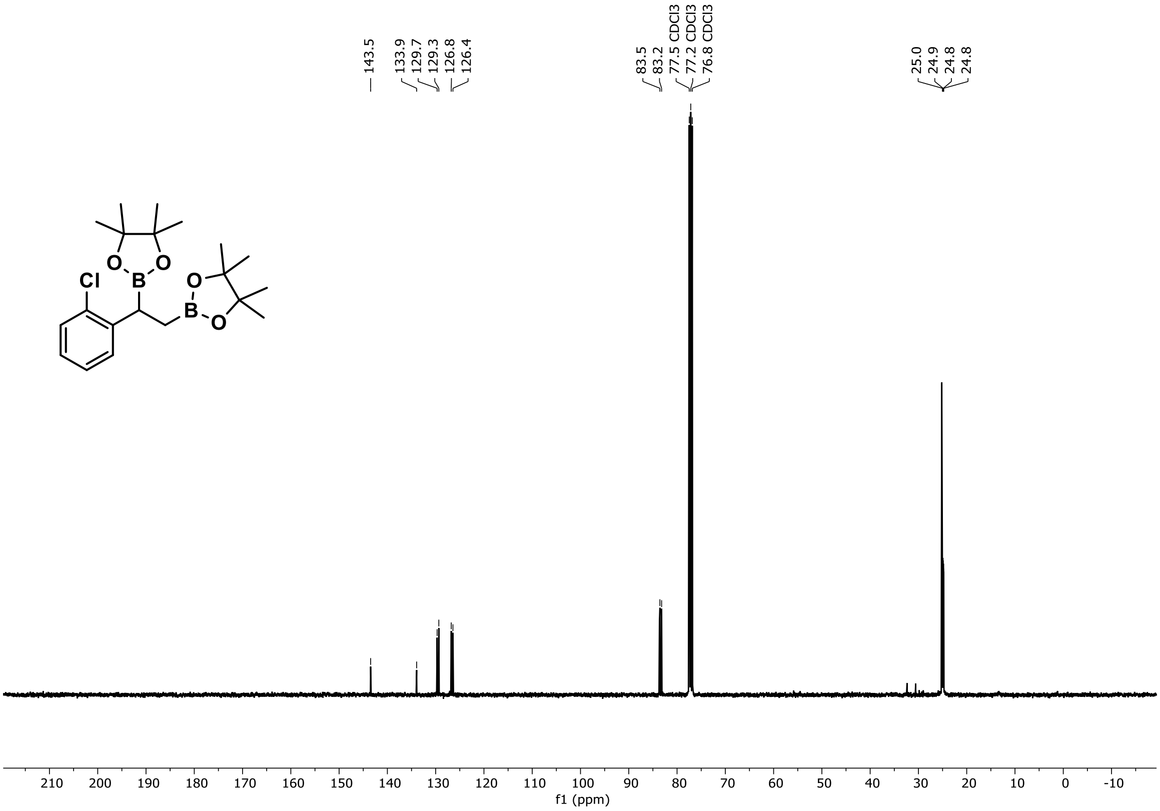

Figure S14. ^{13}C NMR spectrum of *2,2'-(1-(2-chlorophenyl)ethane-1,2-diyl)bis(4,4,5,5-tetramethyl-1,3,2-dioxaborolane)* (**2e**).

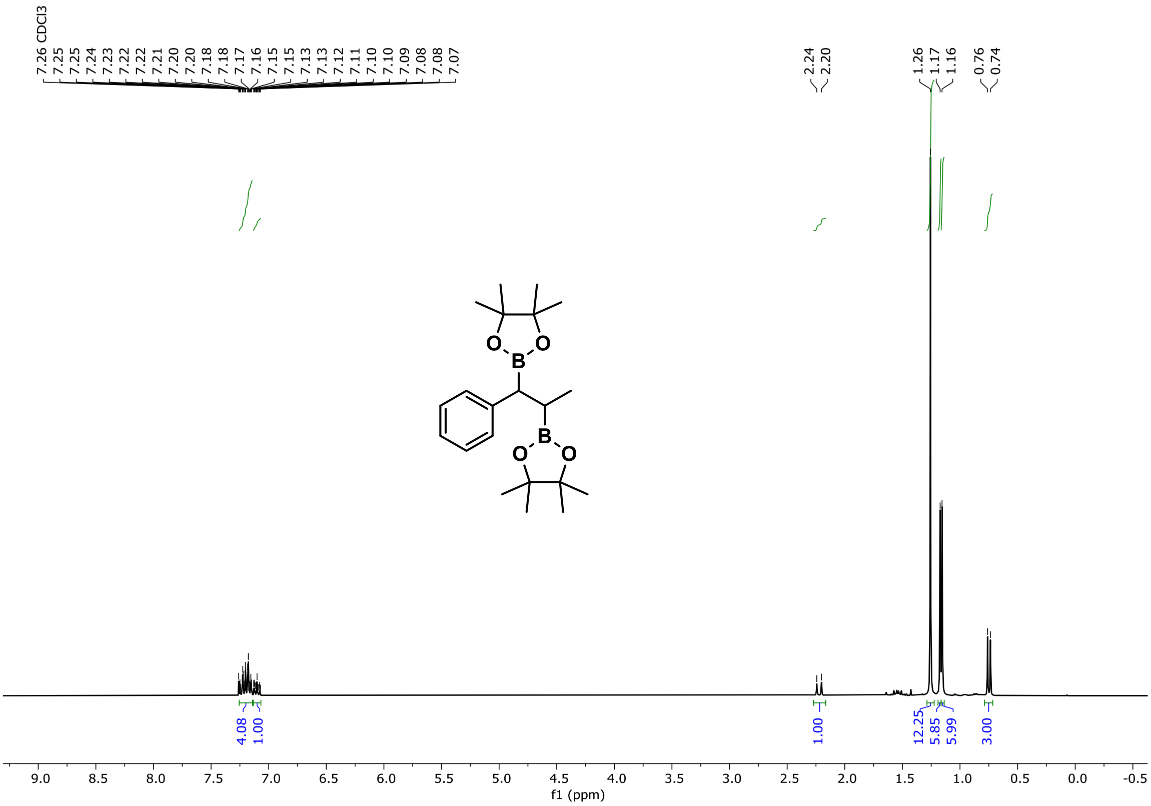

Figure S15. ^{1}H NMR spectrum of *2,2'-(1-phenylpropane-1,2-diyl)bis(4,4,5,5-tetramethyl-1,3,2-dioxaborolane)* (**2f**).

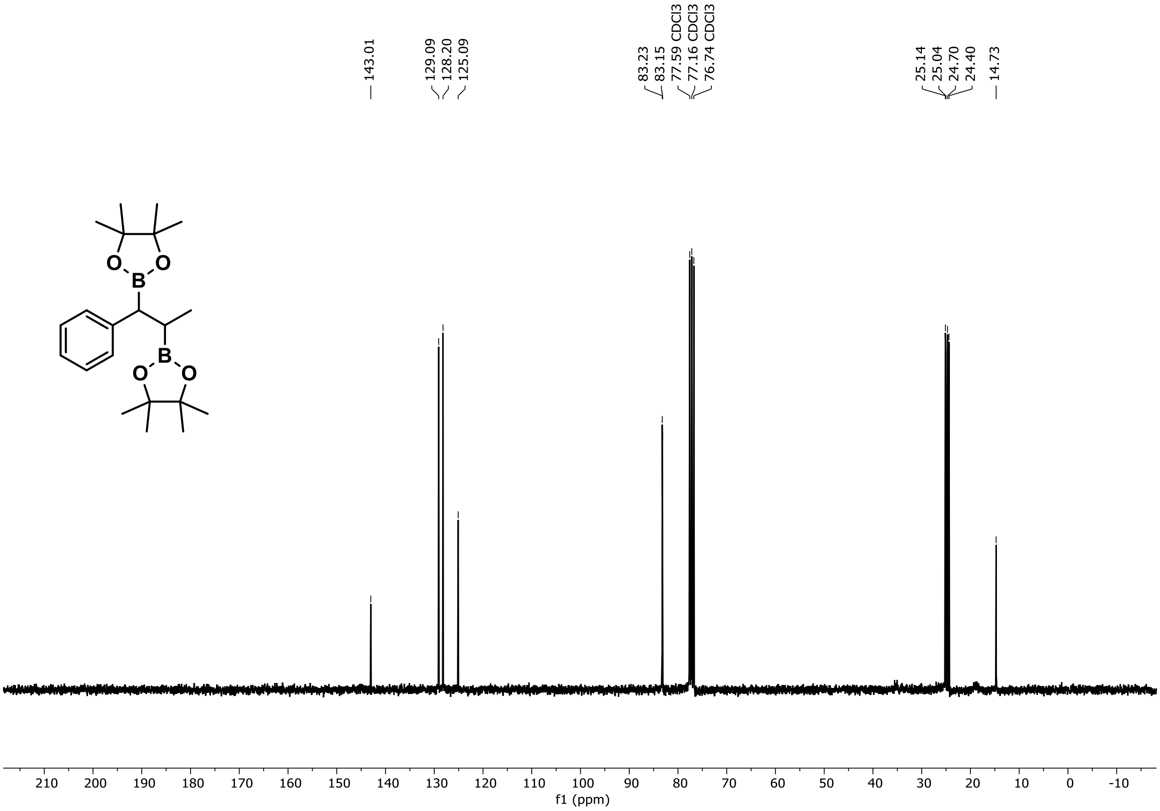

Figure S16. ^{13}C NMR spectrum of *2,2'-(1-phenylpropane-1,2-diyl)bis(4,4,5,5-tetramethyl-1,3,2-dioxaborolane)* (**2f**).

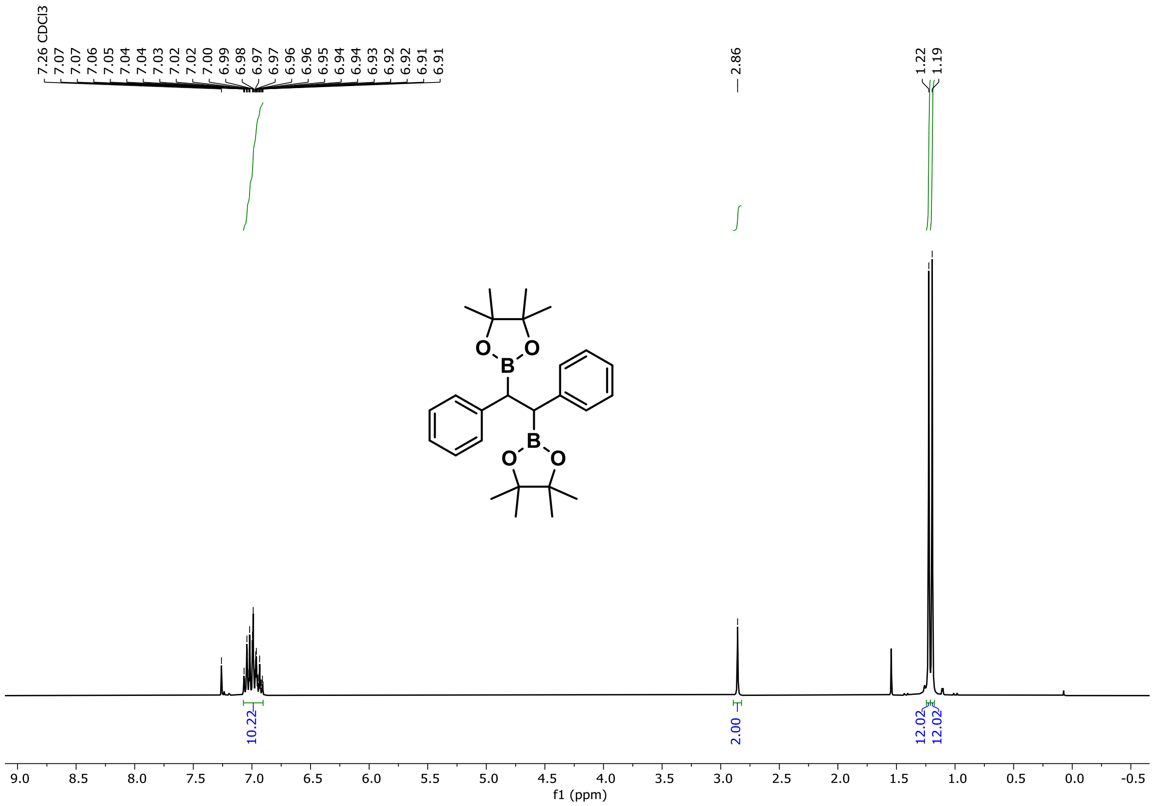

Figure S17. ^{1}H NMR spectrum of *1,2-diphenyl-1,2-bis(4,4,5,5-tetramethyl-1,3,2-dioxaborolan-2-yl)ethane* (**2g**).

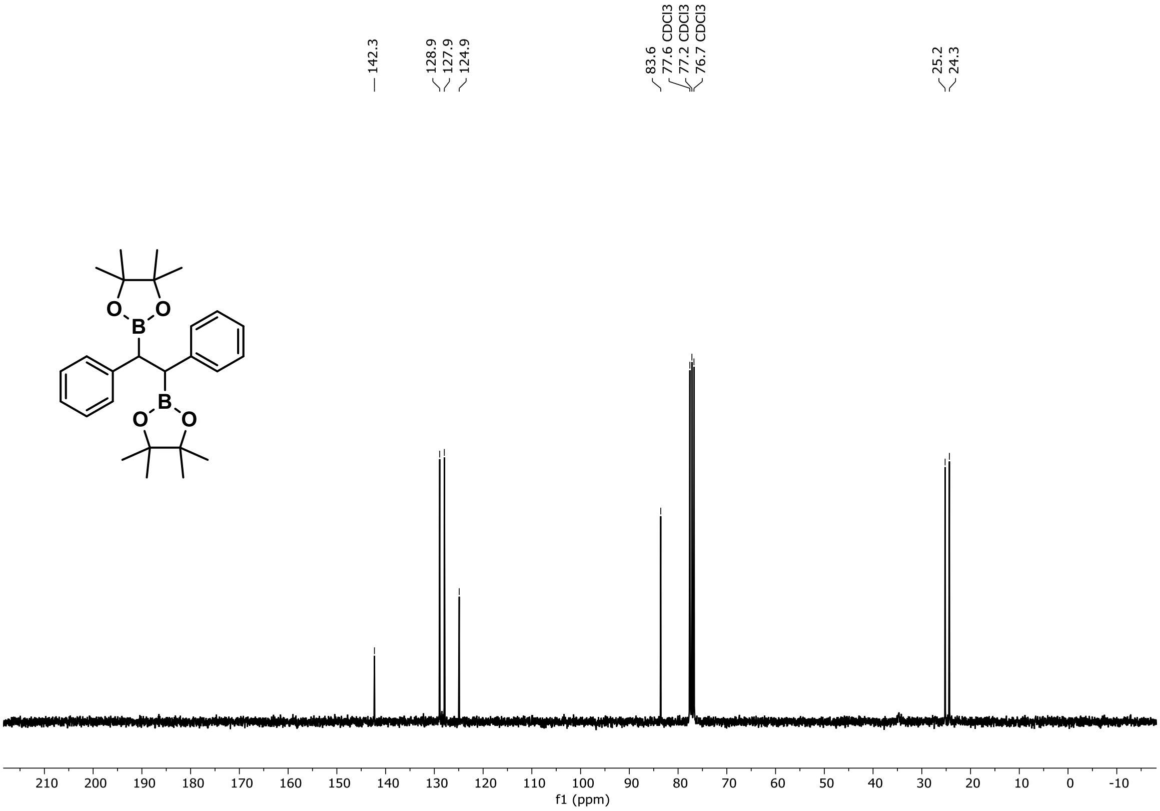

Figure S18. ^{13}C NMR spectrum of *1,2-diphenyl-1,2-bis(4,4,5,5-tetramethyl-1,3,2-dioxaborolan-2-yl)ethane* (**2g**).

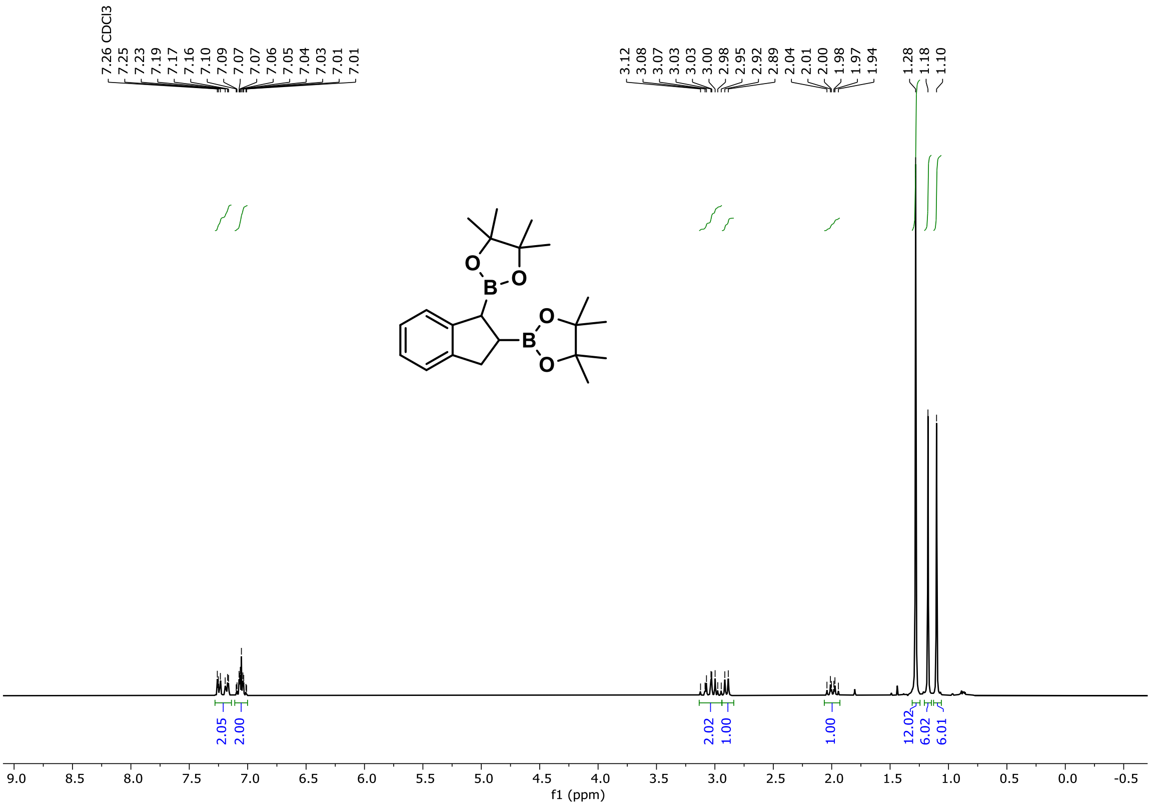

Figure S19. ^{1}H NMR spectrum of *2,2'-(2,3-dihydro-1H-indene-1,2-diyl)bis(4,4,5,5-tetramethyl-1,3,2-dioxaborolane)* (**2h**).

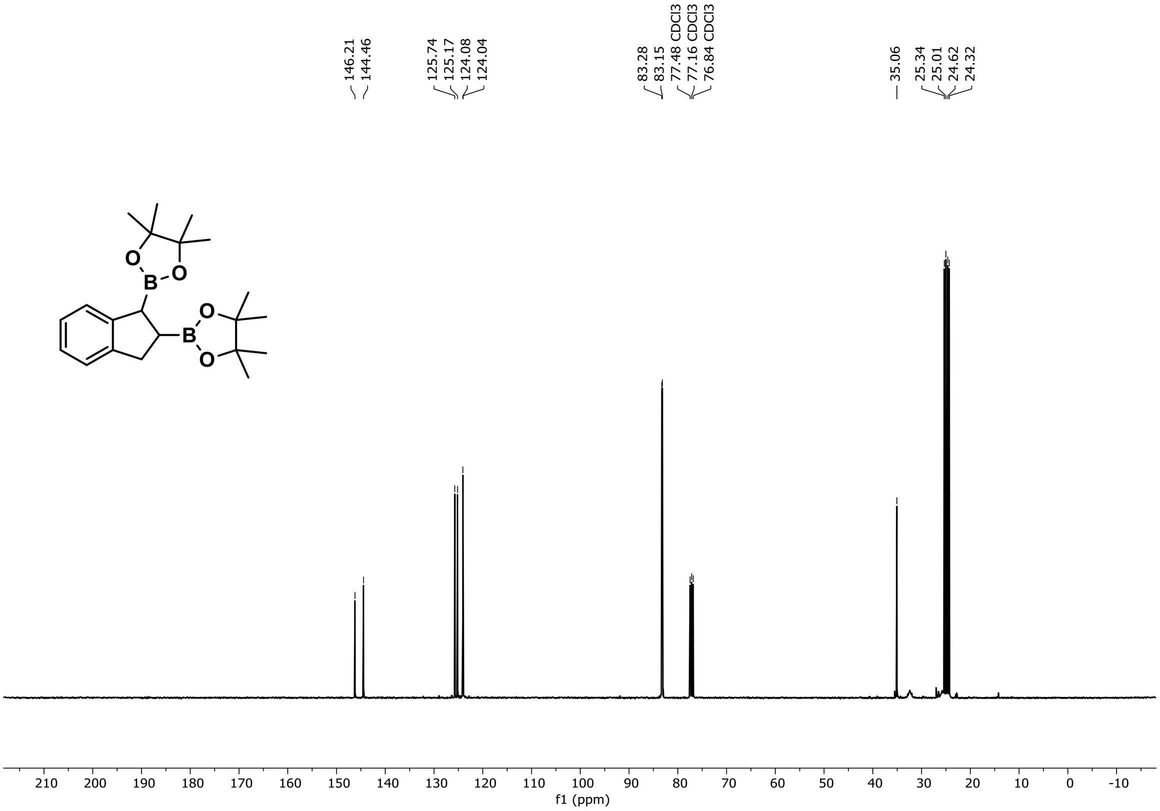

Figure S20. ^{13}C NMR spectrum of *2,2'-(2,3-dihydro-1H-indene-1,2-diyl)bis(4,4,5,5-tetramethyl-1,3,2-dioxaborolane)* (**2h**).

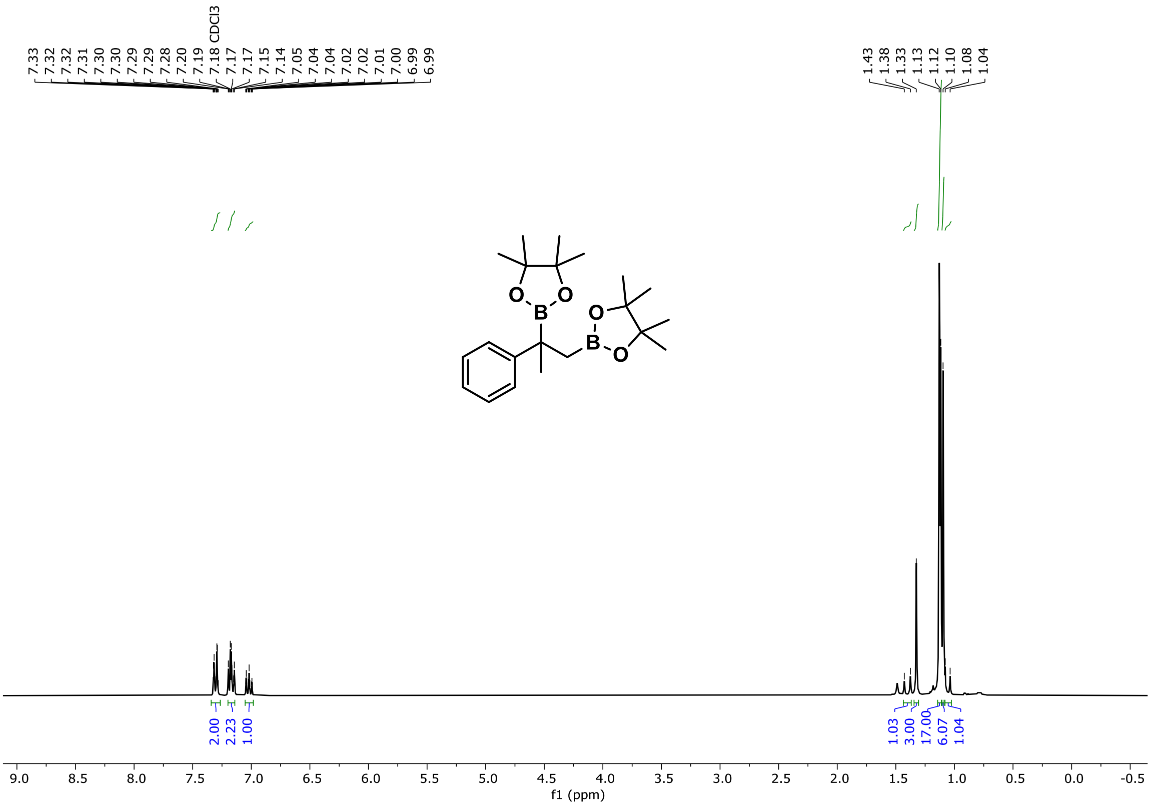

Figure S21. ^{1}H NMR spectrum of *2,2'-(2-phenylpropane-1,2-diyl)bis(4,4,5,5-tetramethyl-1,3,2-dioxaborolane)* (**2j**).

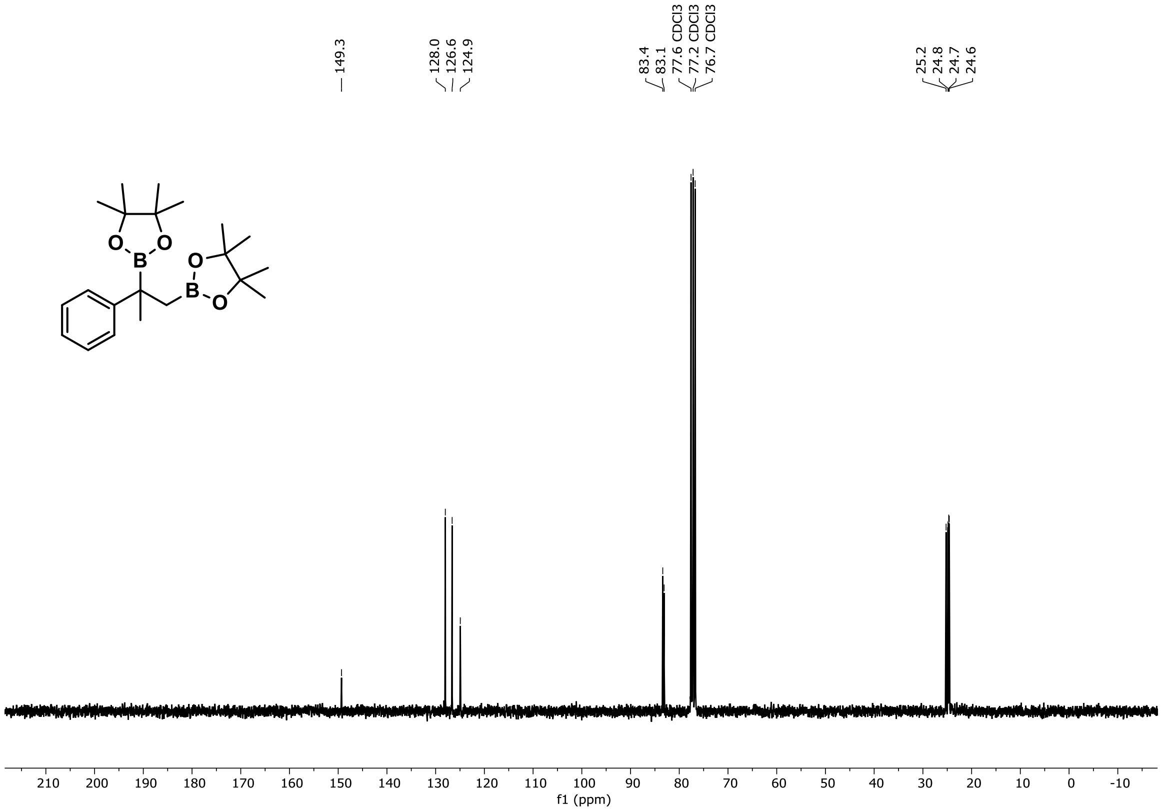

Figure S22. ^{13}C NMR spectrum of *2,2'-(2-phenylpropane-1,2-diyl)bis(4,4,5,5-tetramethyl-1,3,2-dioxaborolane)* (**2j**).

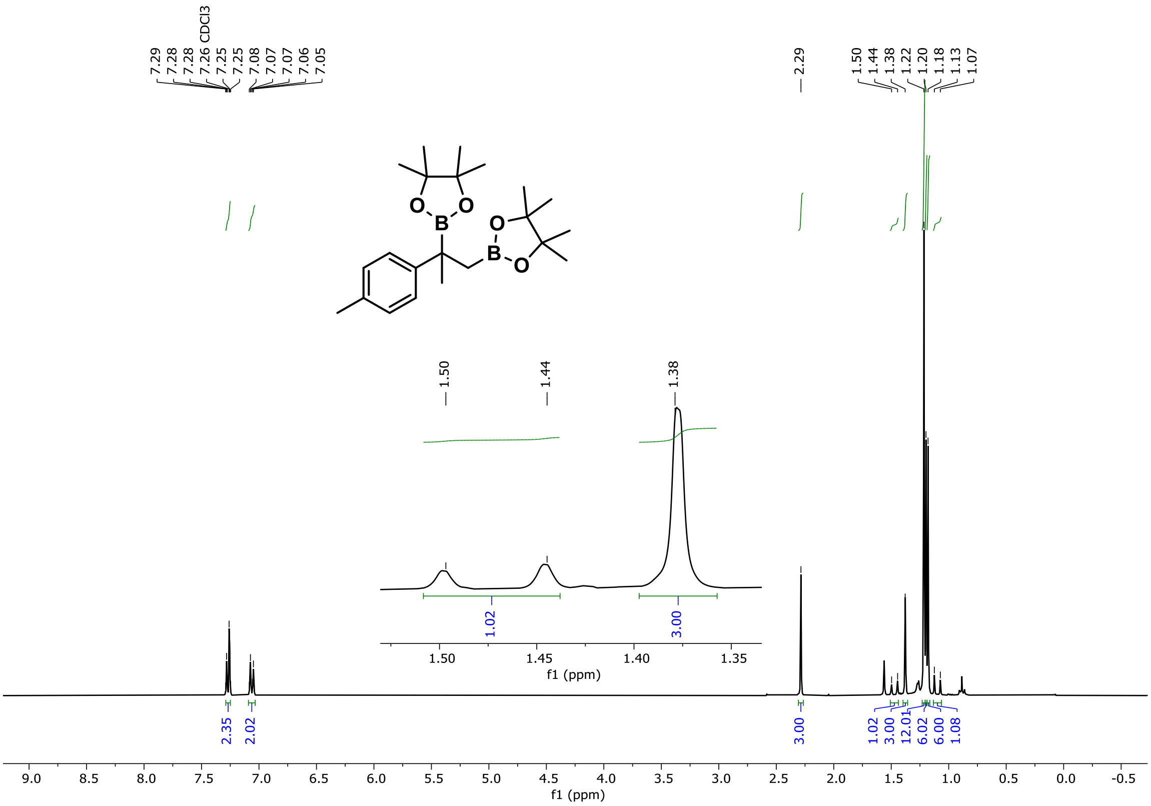

Figure S23. ^{1}H NMR spectrum of *2,2'-(2-(p-tolyl)propane-1,2-diyl)bis(4,4,5,5-tetramethyl-1,3,2-dioxaborolane)* (**2k**).

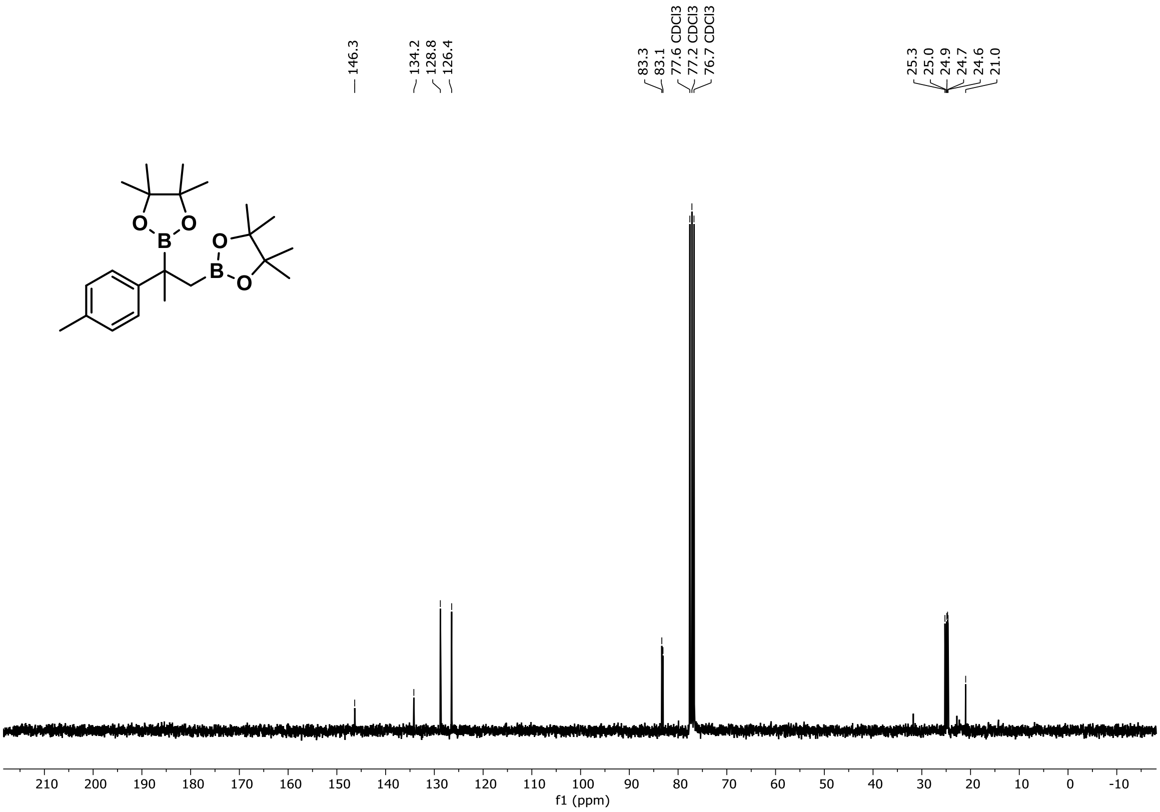

Figure S24. ^{13}C NMR spectrum of *2,2'-(2-(p-tolyl)propane-1,2-diyl)bis(4,4,5,5-tetramethyl-1,3,2-dioxaborolane)* (**2k**).

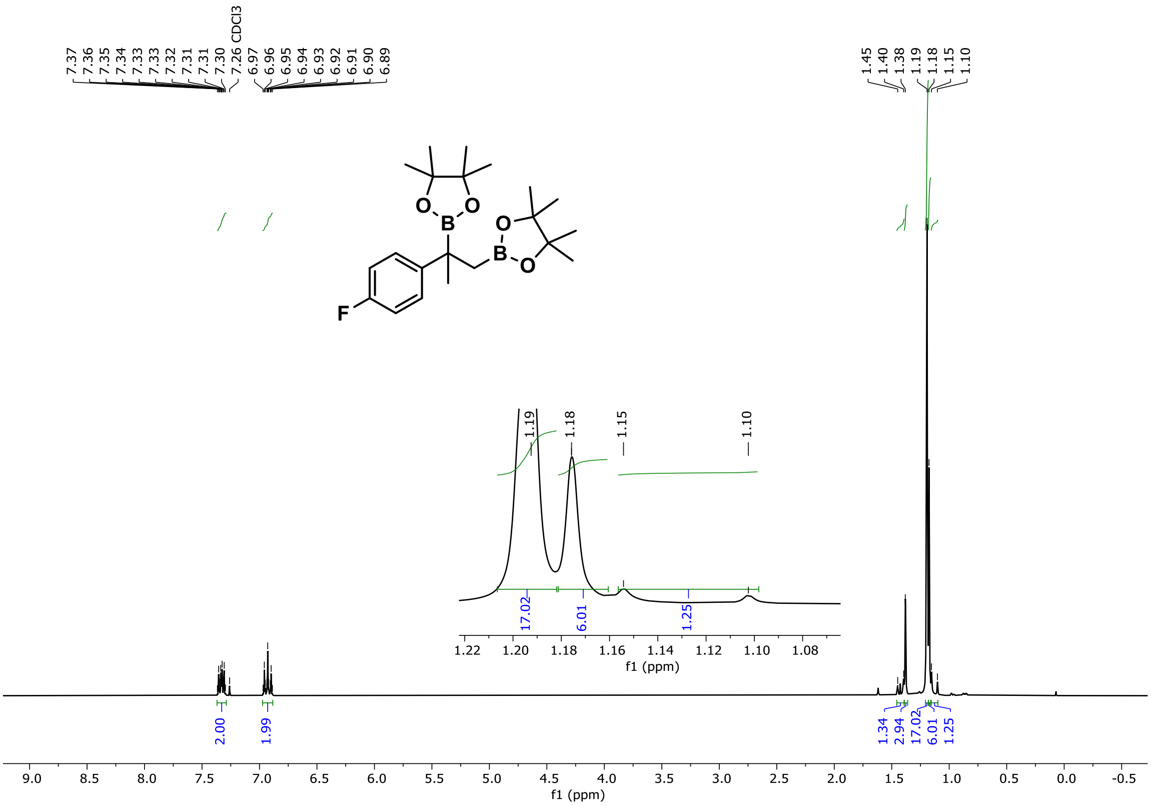

Figure S25. ^{1}H NMR spectrum of *2,2'-(2-(4-fluorophenyl)propane-1,2-diyl)bis(4,4,5,5-tetramethyl-1,3,2-dioxaborolane)* (**2l**).

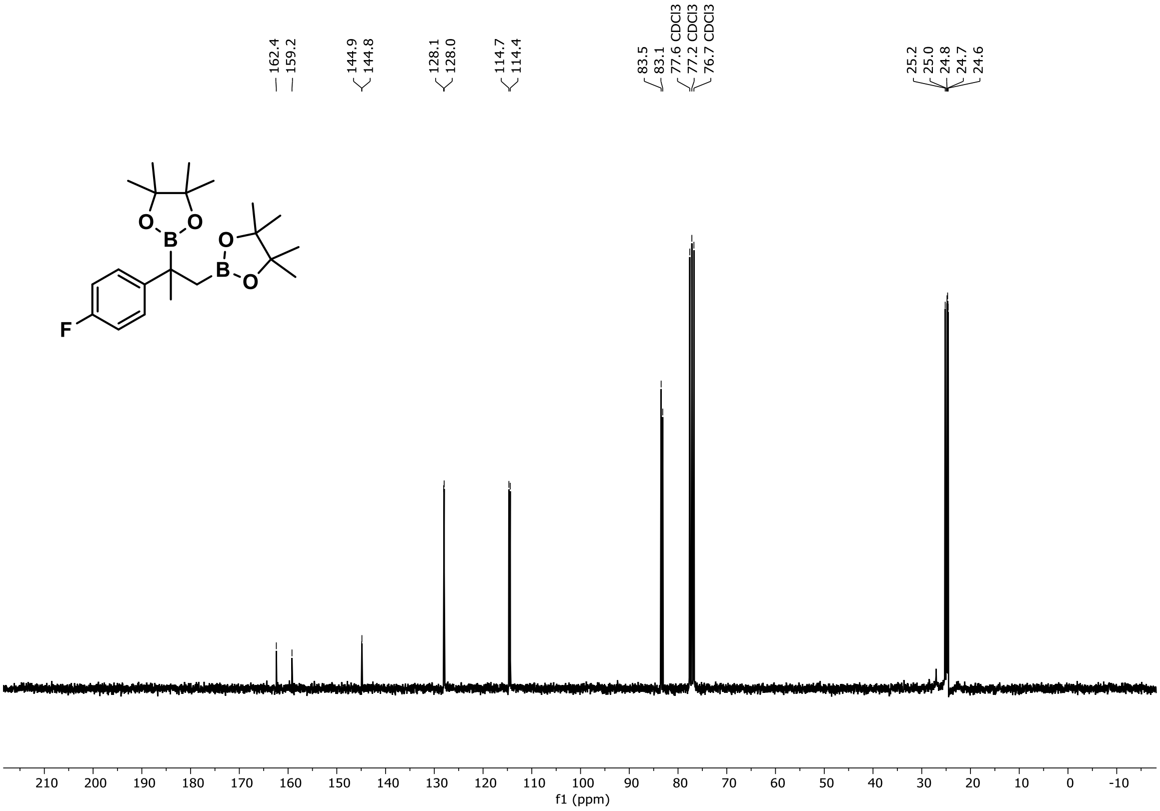

Figure S26. ^{13}C NMR spectrum of *2,2'-(2-(4-fluorophenyl)propane-1,2-diyl)bis(4,4,5,5-tetramethyl-1,3,2-dioxaborolane)* (**2l**).

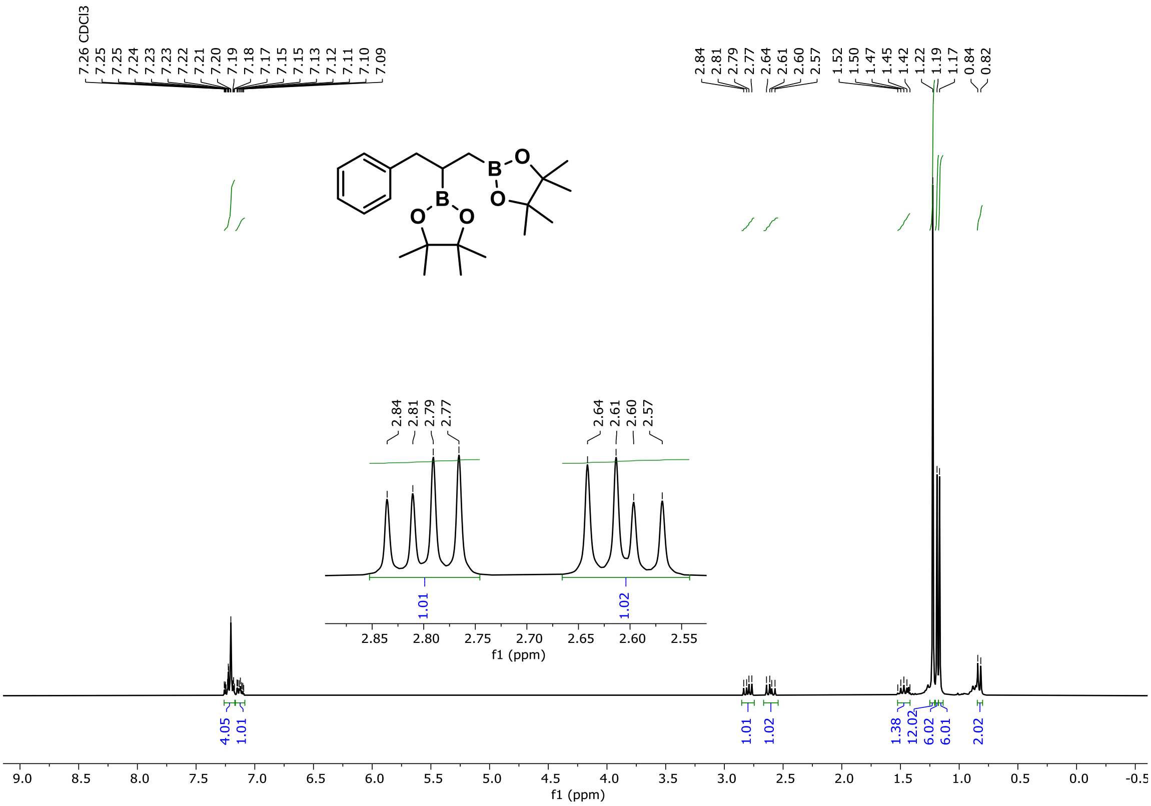

Figure S27. ^{1}H NMR spectrum of *2,2'-(3-phenylpropane-1,2-diyl)bis(4,4,5,5-tetramethyl-1,3,2-dioxaborolane)* (**2r**).

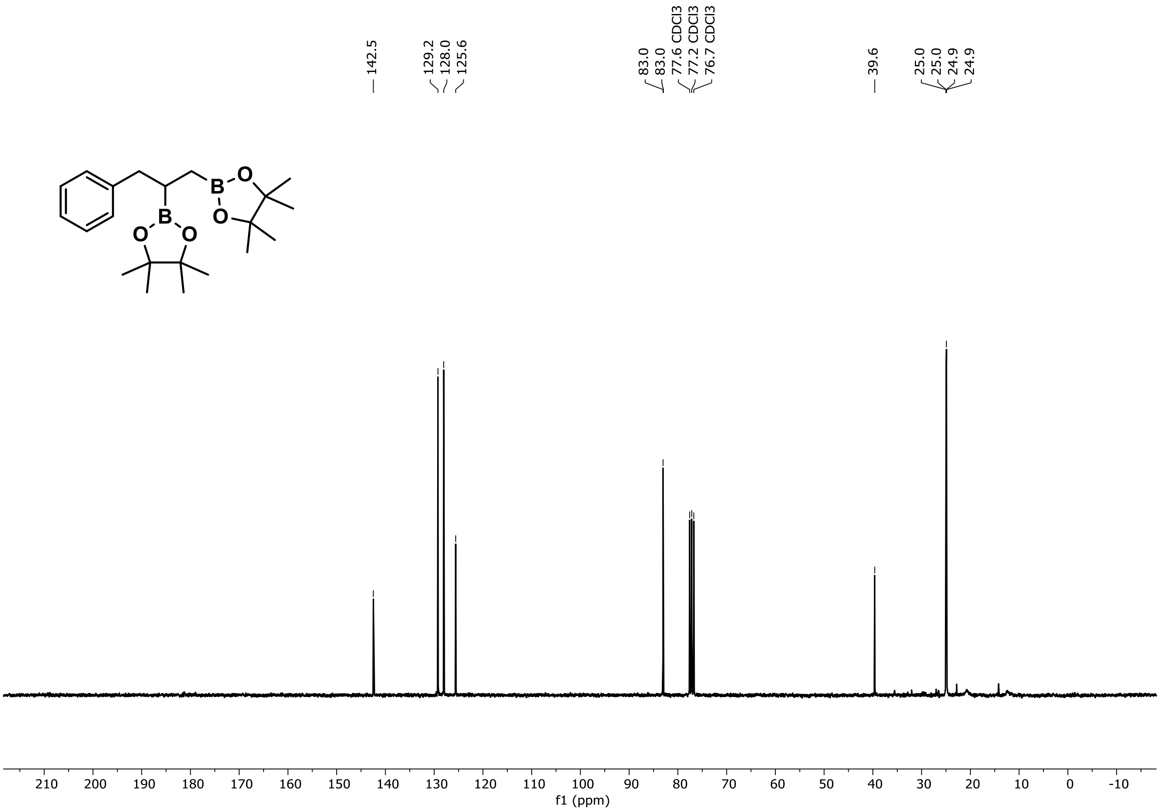

Figure S28. ^{13}C NMR spectrum of *2,2'-(3-phenylpropane-1,2-diyl)bis(4,4,5,5-tetramethyl-1,3,2-dioxaborolane)* (**2r**).

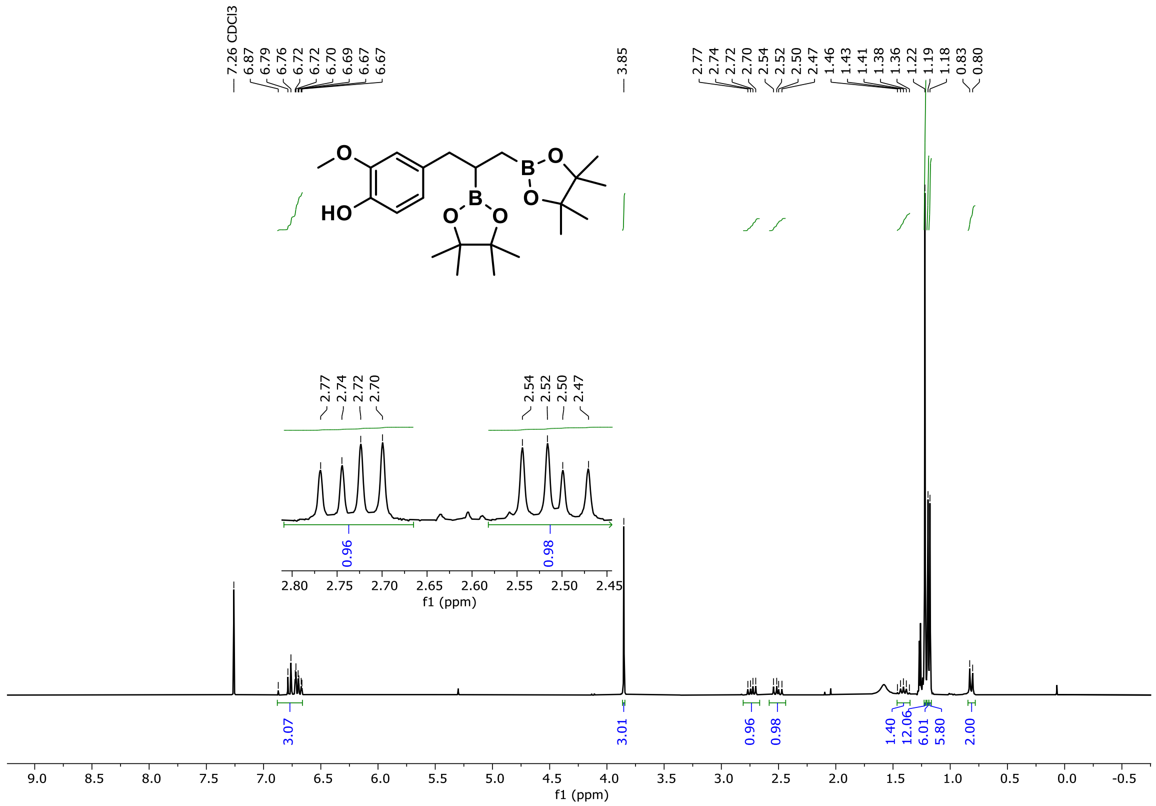

Figure S29. ^{1}H NMR spectrum of *4-(2,3-bis(4,4,5,5-tetramethyl-1,3,2-dioxaborolan-2-yl)propyl)-2-methoxyphenol* (**2m**).

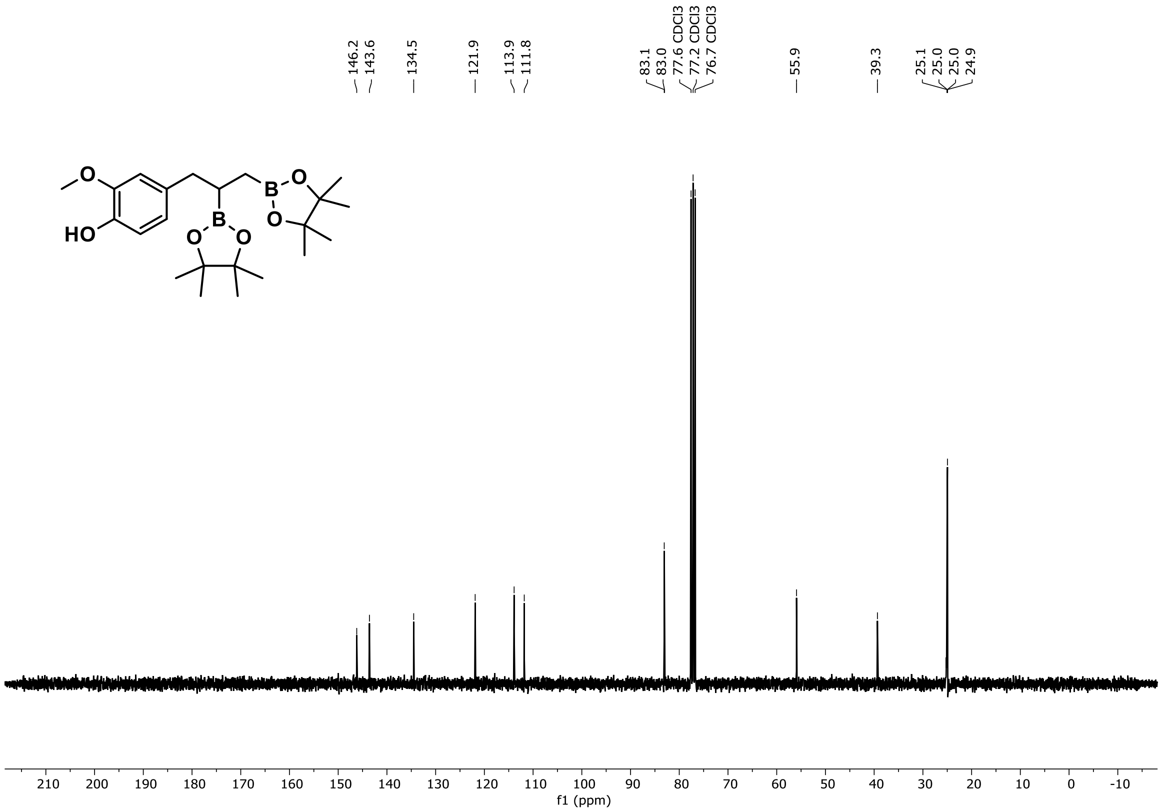

Figure S30. ^{13}C NMR spectrum of *4-(2,3-bis(4,4,5,5-tetramethyl-1,3,2-dioxaborolan-2-yl)propyl)-2-methoxyphenol* (**2m**).

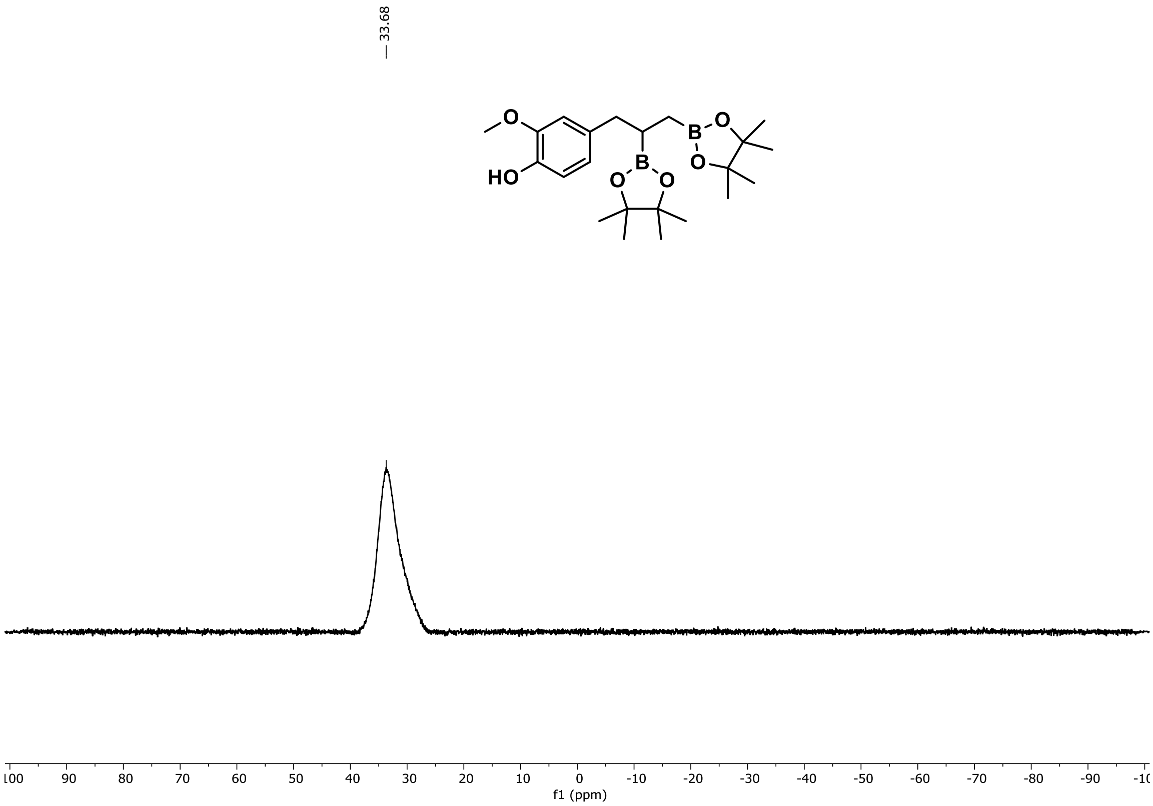

Figure S31. ^{11}B NMR spectrum of *4-(2,3-bis(4,4,5,5-tetramethyl-1,3,2-dioxaborolan-2-yl)propyl)-2-methoxyphenol* (**2m**).

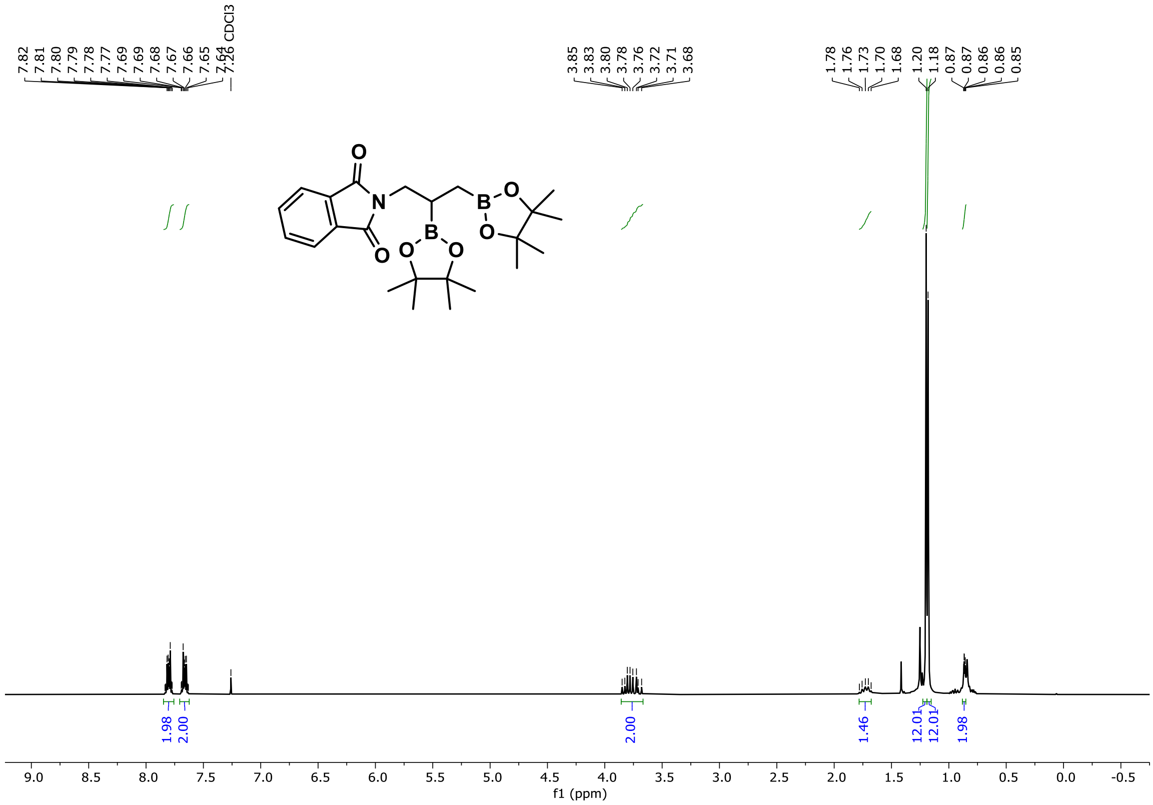

Figure S32. ^{1}H NMR spectrum of *2-(2,3-bis(4,4,5,5-tetramethyl-1,3,2-dioxaborolan-2-yl)propyl)isoindoline-1,3-dione* (**2o**).

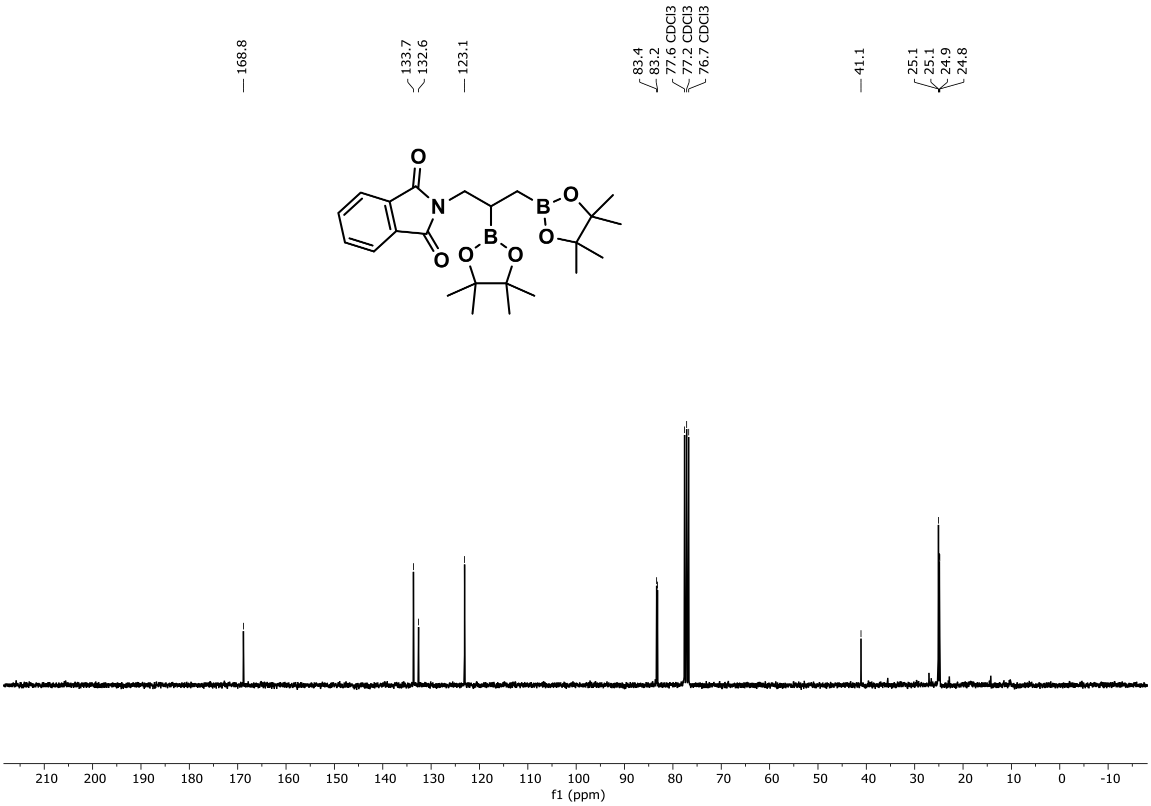

Figure S33. ^{13}C NMR spectrum of *2-(2,3-bis(4,4,5,5-tetramethyl-1,3,2-dioxaborolan-2-yl)propyl)isoindoline-1,3-dione* (**2o**).

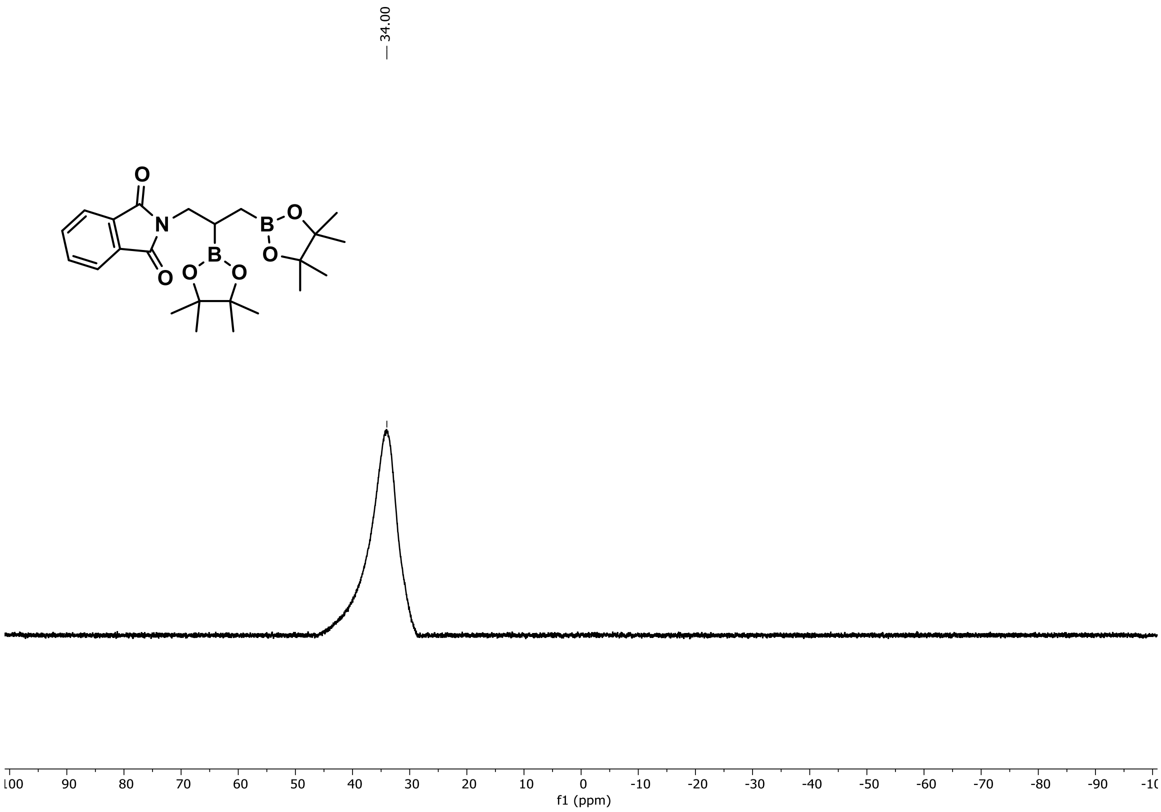

Figure S34. ^{11}B NMR spectrum of *2-(2,3-bis(4,4,5,5-tetramethyl-1,3,2-dioxaborolan-2-yl)propyl)isoindoline-1,3-dione* (**2o**).

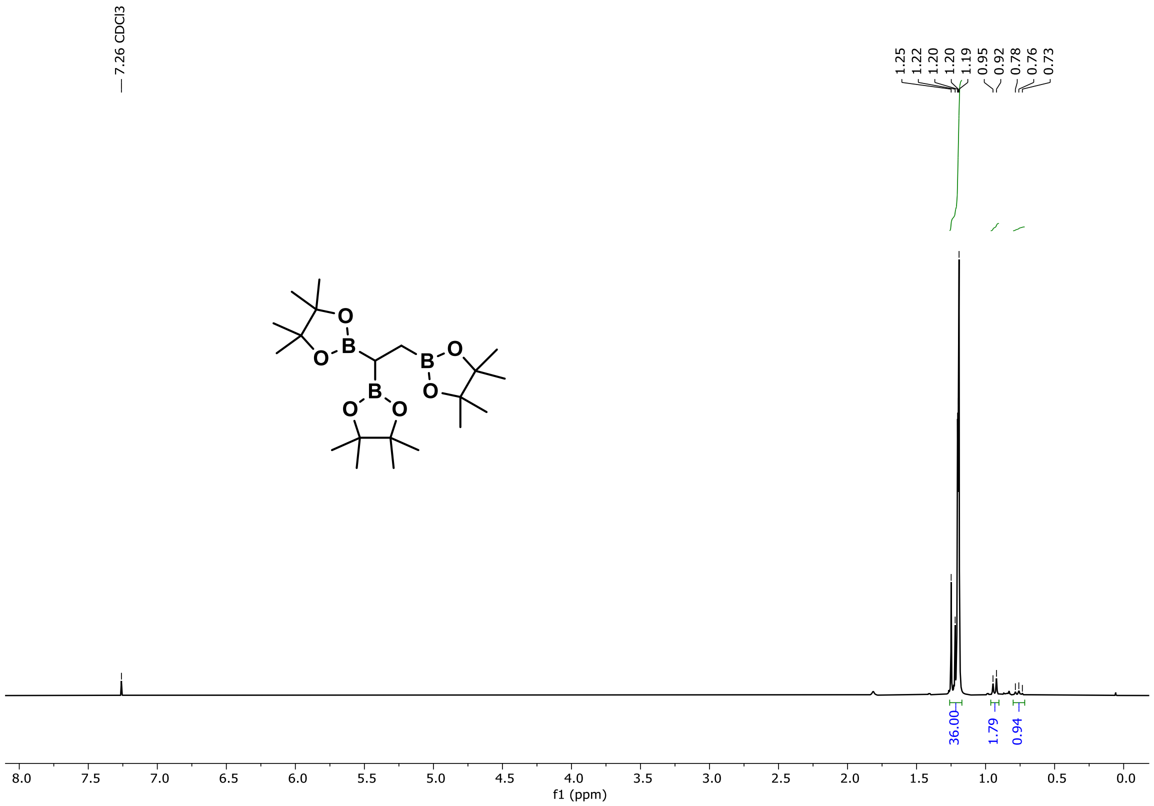

Figure S35. ^{1}H NMR spectrum of *2,2',2''-(ethane-1,1,2-triyl)tris(4,4,5,5-tetramethyl-1,3,2-dioxaborolane)* (**2q**).

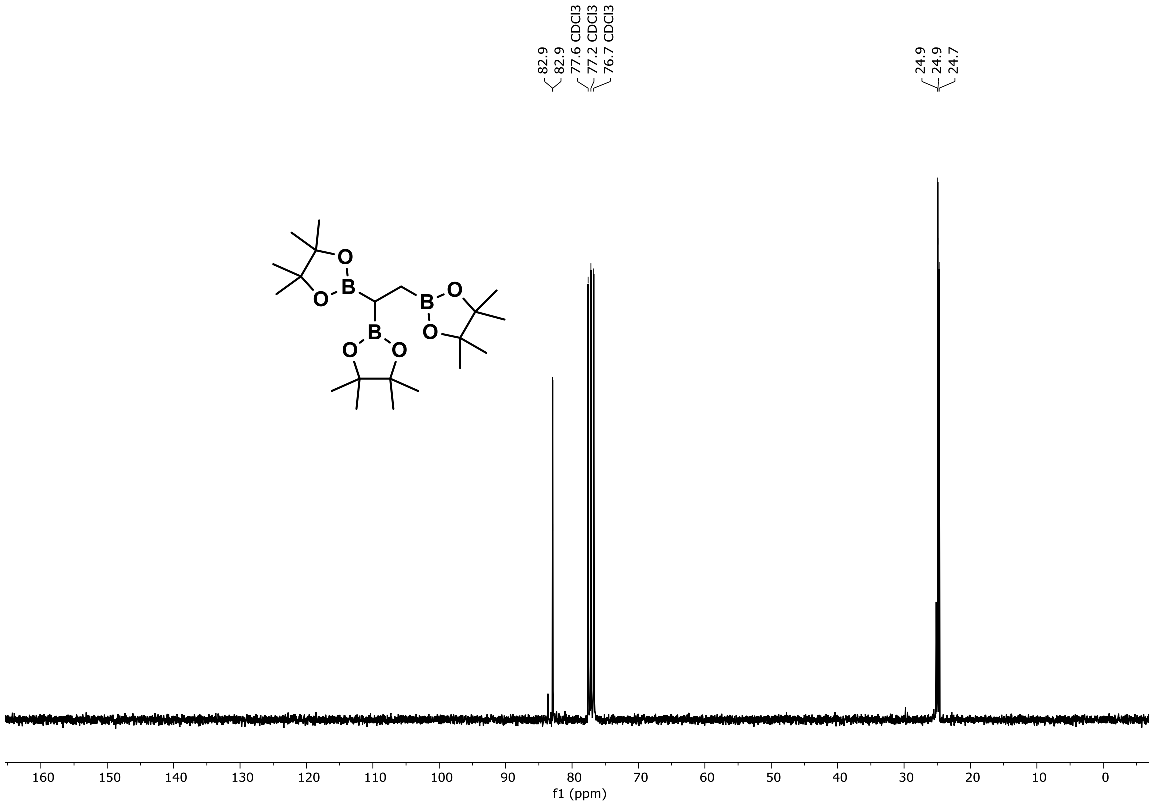

Figure S36. ^{13}C NMR spectrum of *2,2',2''-(ethane-1,1,2-triyl)tris(4,4,5,5-tetramethyl-1,3,2-dioxaborolane)* (**2q**).

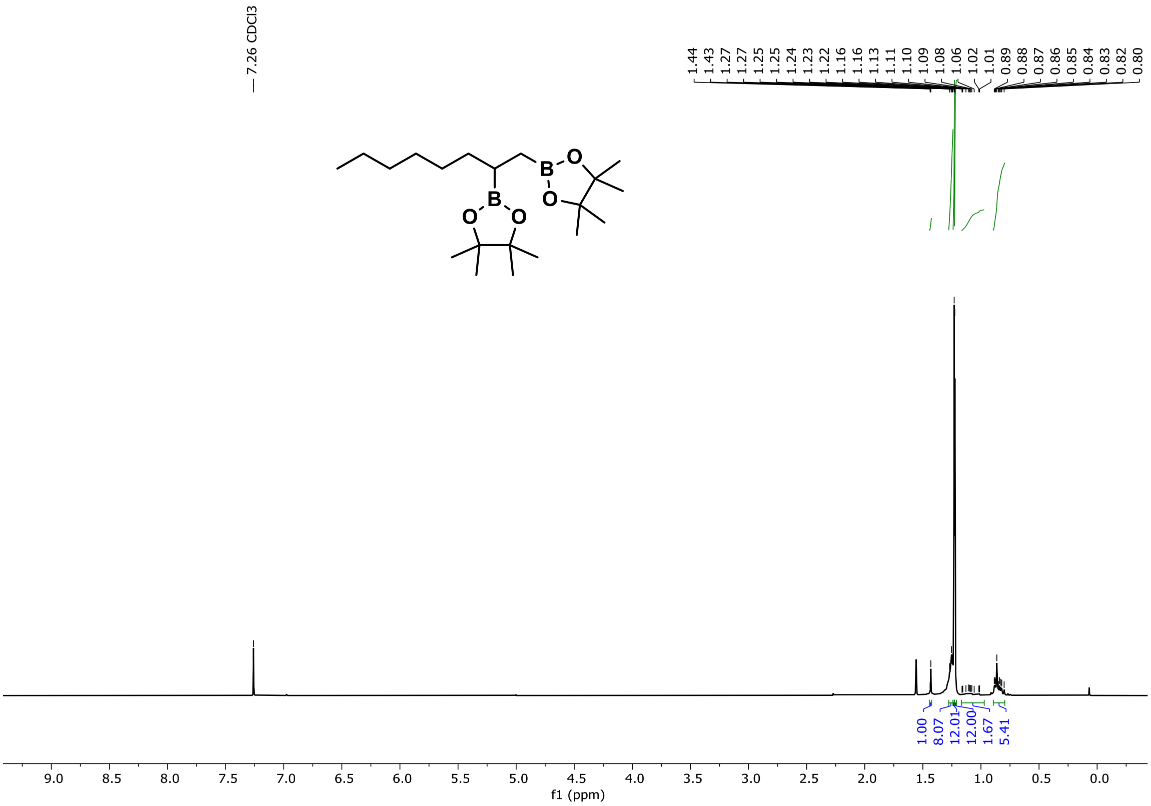

Figure S37. ^{1}H NMR spectrum of *2,2'-(octane-1,2-diyl)bis(4,4,5,5-tetramethyl-1,3,2-dioxaborolane)* (**2s**).

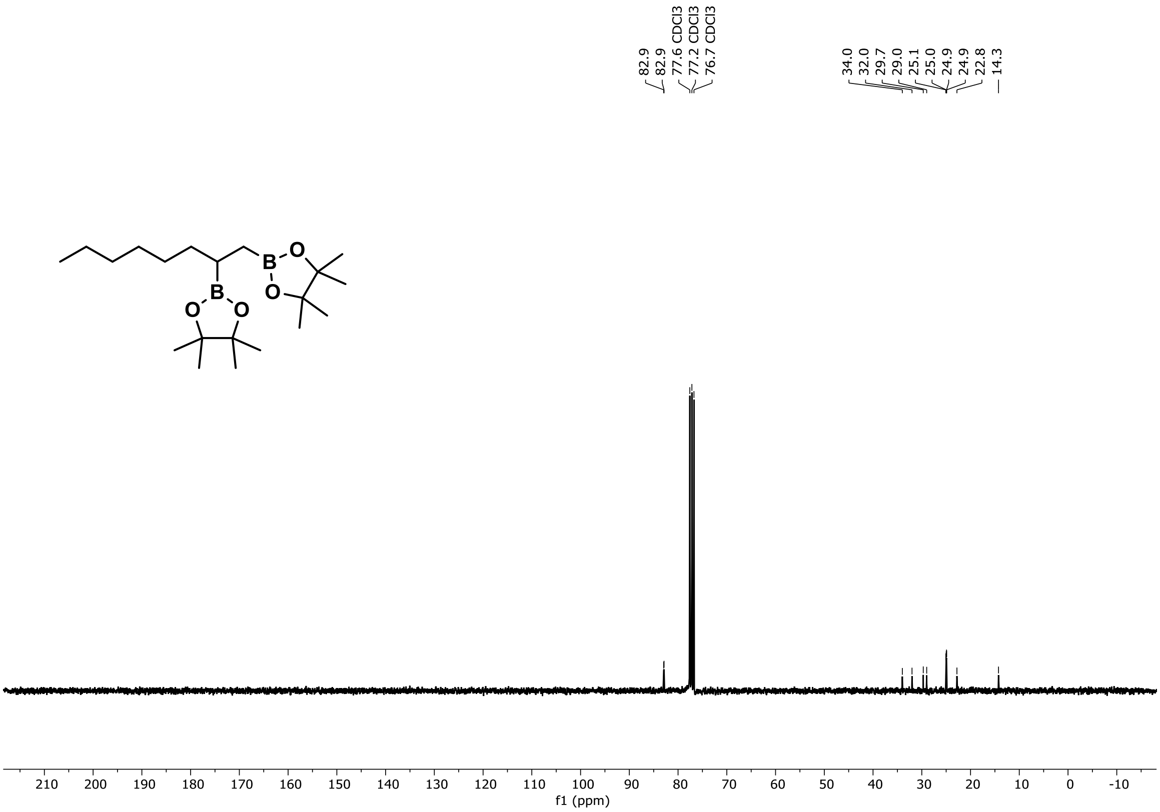

Figure S38. ^{13}C NMR spectrum of *2,2'-(octane-1,2-diyl)bis(4,4,5,5-tetramethyl-1,3,2-dioxaborolane)* (**2s**).

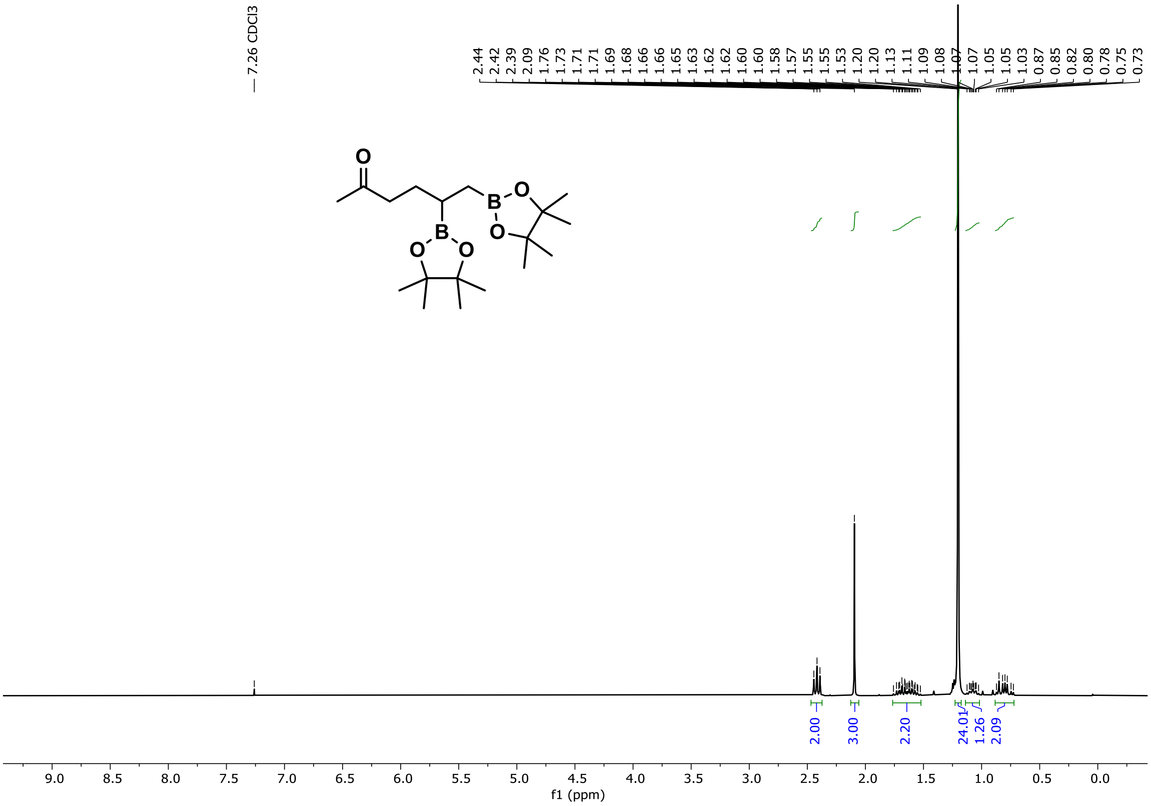

Figure S39. ^{1}H NMR spectrum of *5,6-bis(4,4,5,5-tetramethyl-1,3,2-dioxaborolan-2-yl)hexan-2-one)* (**2t**).

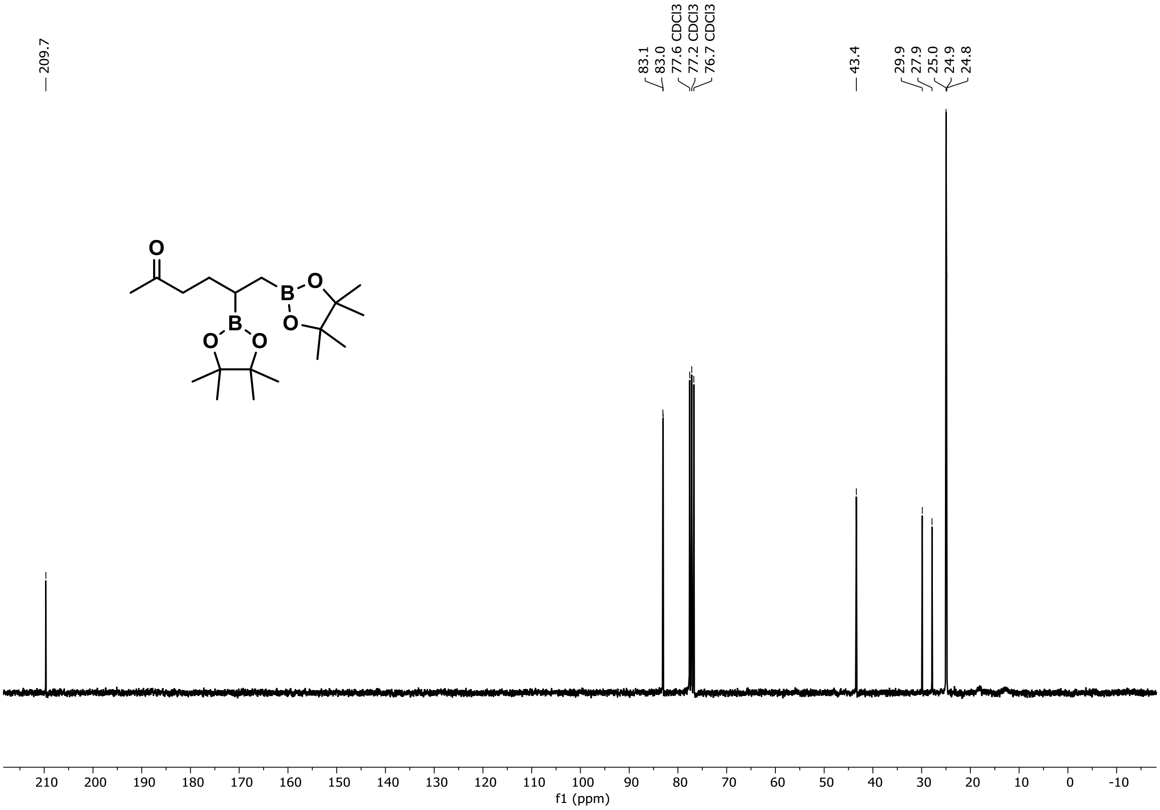

Figure S40. ^{13}C NMR spectrum of *5,6-bis(4,4,5,5-tetramethyl-1,3,2-dioxaborolan-2-yl)hexan-2-one* (**2t**).

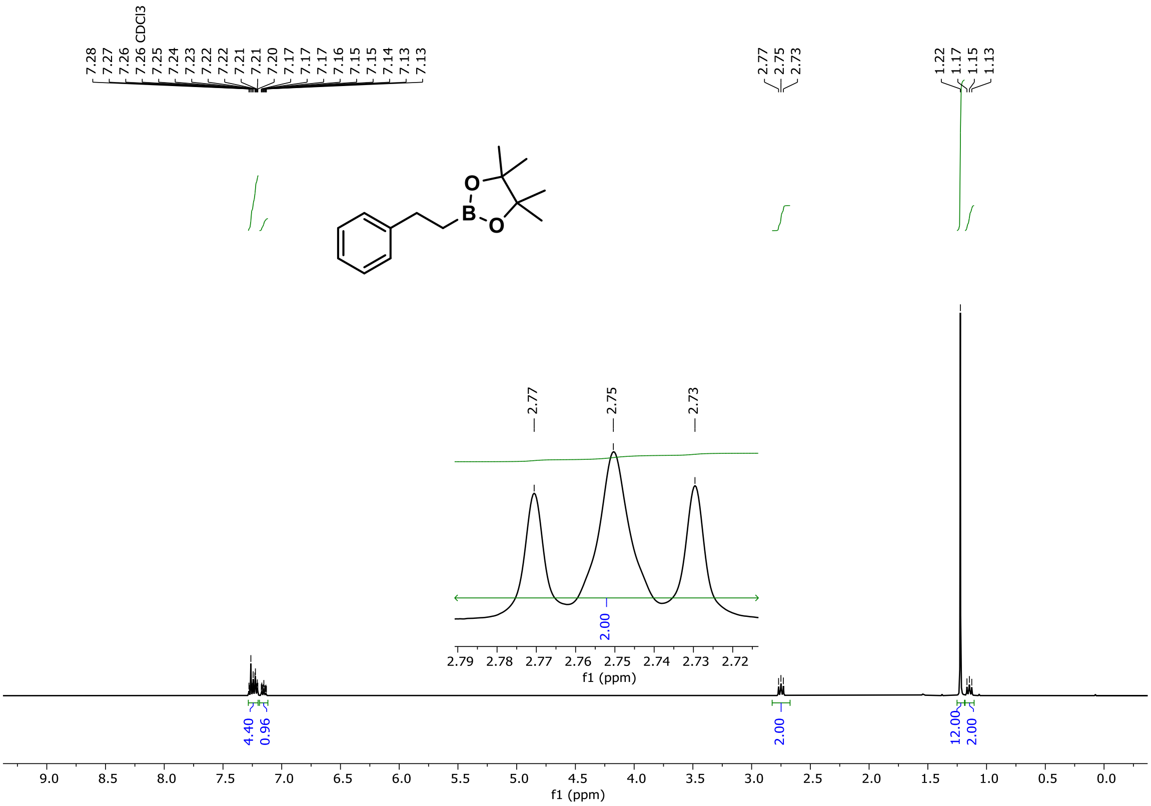

Figure S41. ^{1}H NMR spectrum of *4,4,5,5-tetramethyl-2-phenethyl-1,3,2-dioxaborolane* (**3a**).

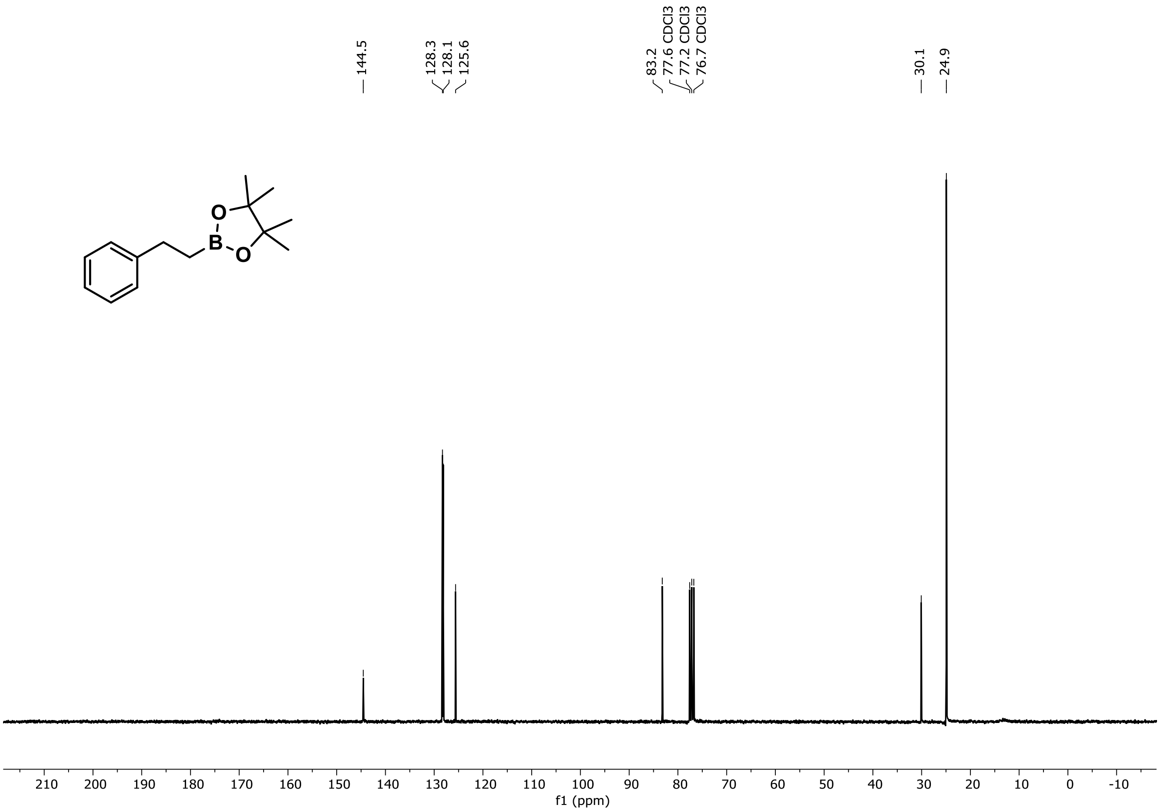

Figure S42. ^{13}C NMR spectrum of *4,4,5,5-tetramethyl-2-phenethyl-1,3,2-dioxaborolane* (**3a**).

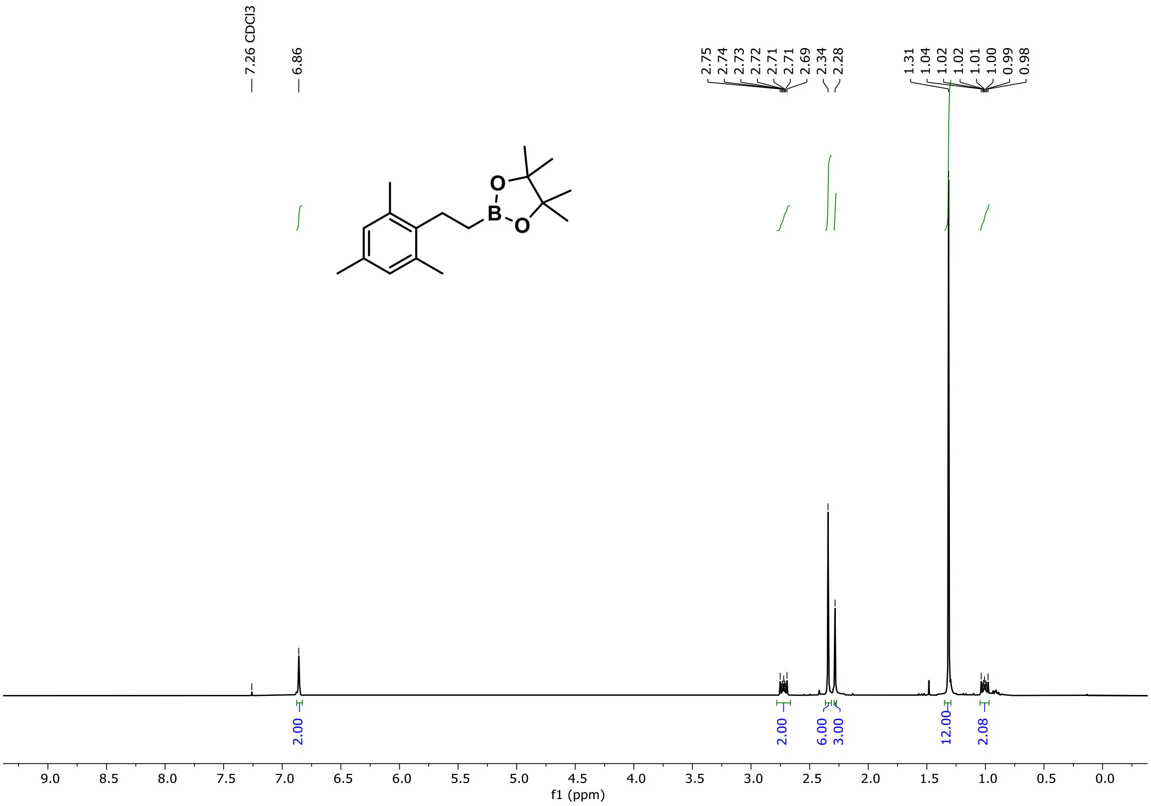

Figure S43. ^{1}H NMR spectrum of *4,4,5,5-tetramethyl-2-(2,4,6-trimethylphenethyl)-1,3,2-dioxaborolane* (**3u**).

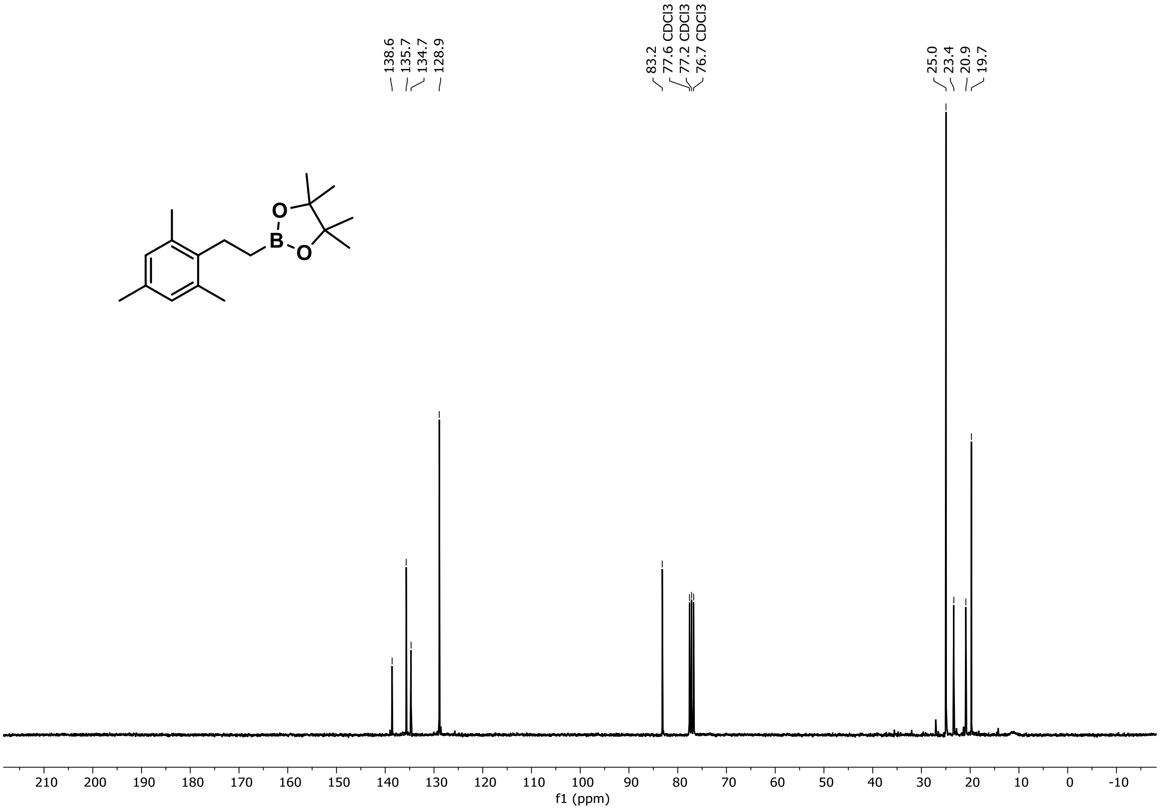

Figure S44. ^{13}C NMR spectrum of *4,4,5,5-tetramethyl-2-(2,4,6-trimethylphenethyl)-1,3,2-dioxaborolane* (**3u**).

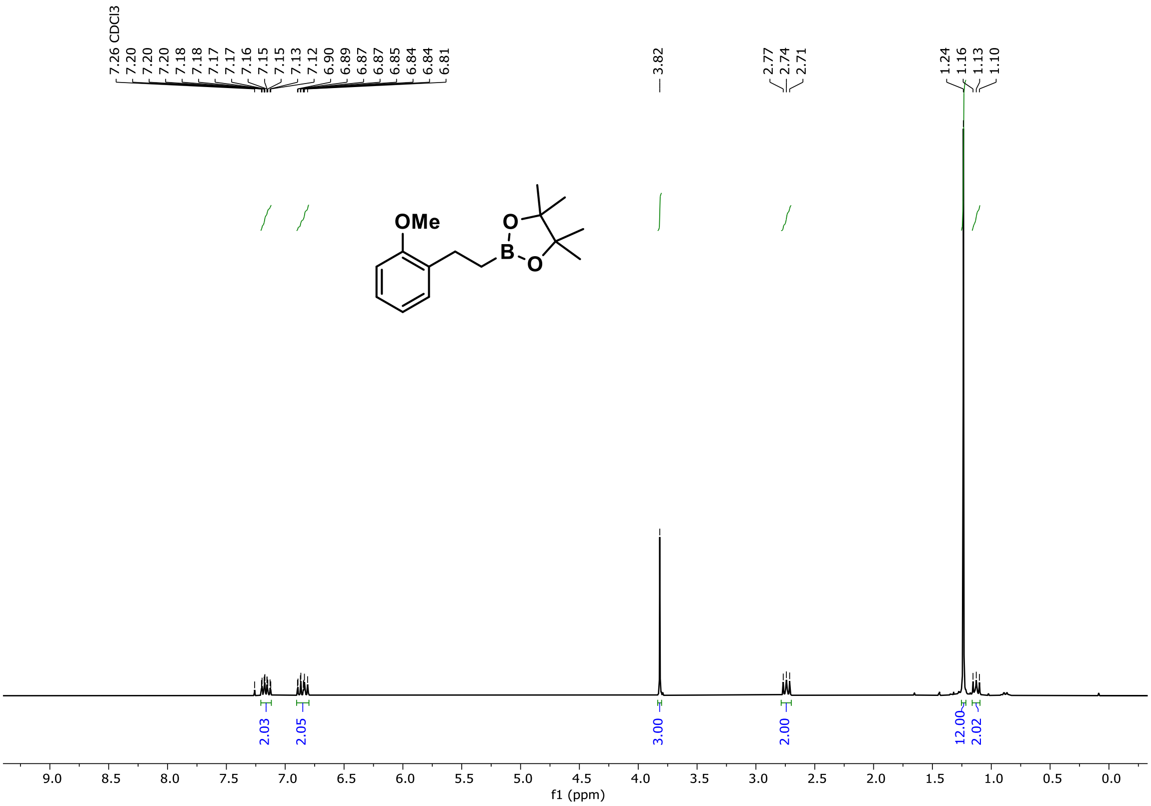

Figure S45. ^{1}H NMR spectrum of *2-(2-methoxyphenethyl)-4,4,5,5-tetramethyl-1,3,2-dioxaborolane* (**3v**).

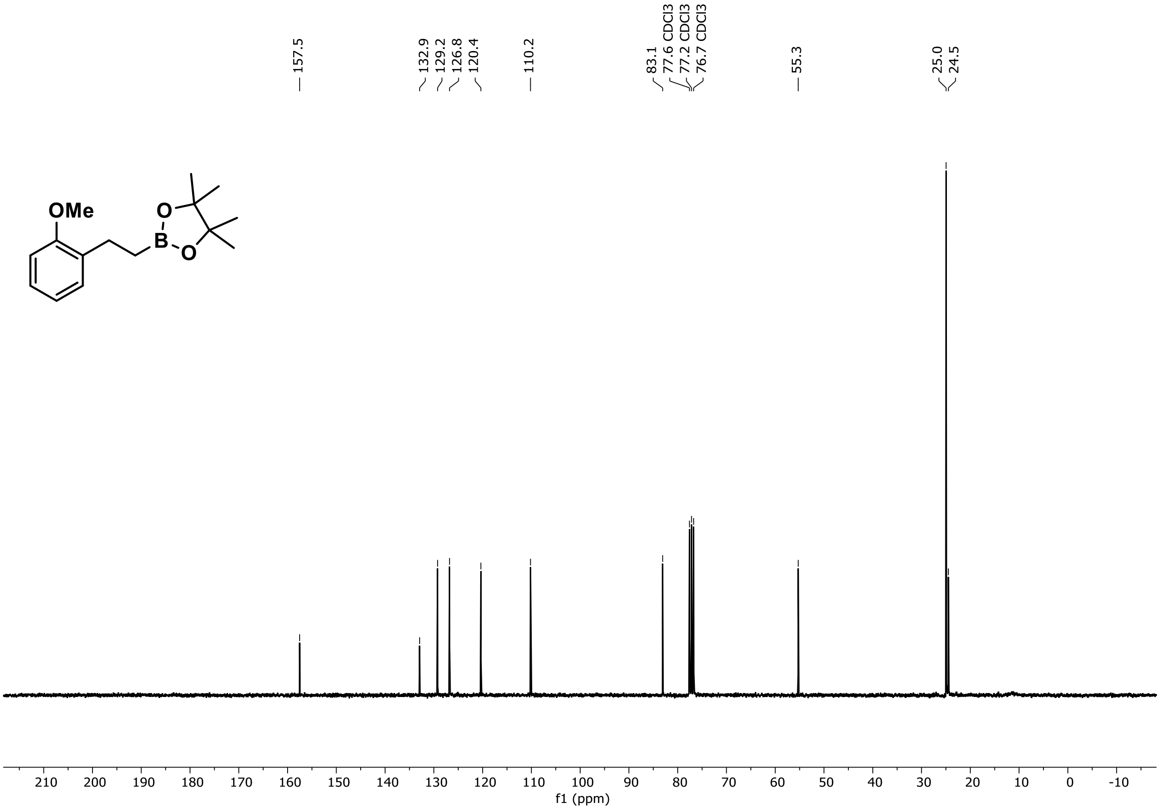

Figure S46. ^{13}C NMR spectrum of *2-(2-methoxyphenethyl)-4,4,5,5-tetramethyl-1,3,2-dioxaborolane* (**3v**).

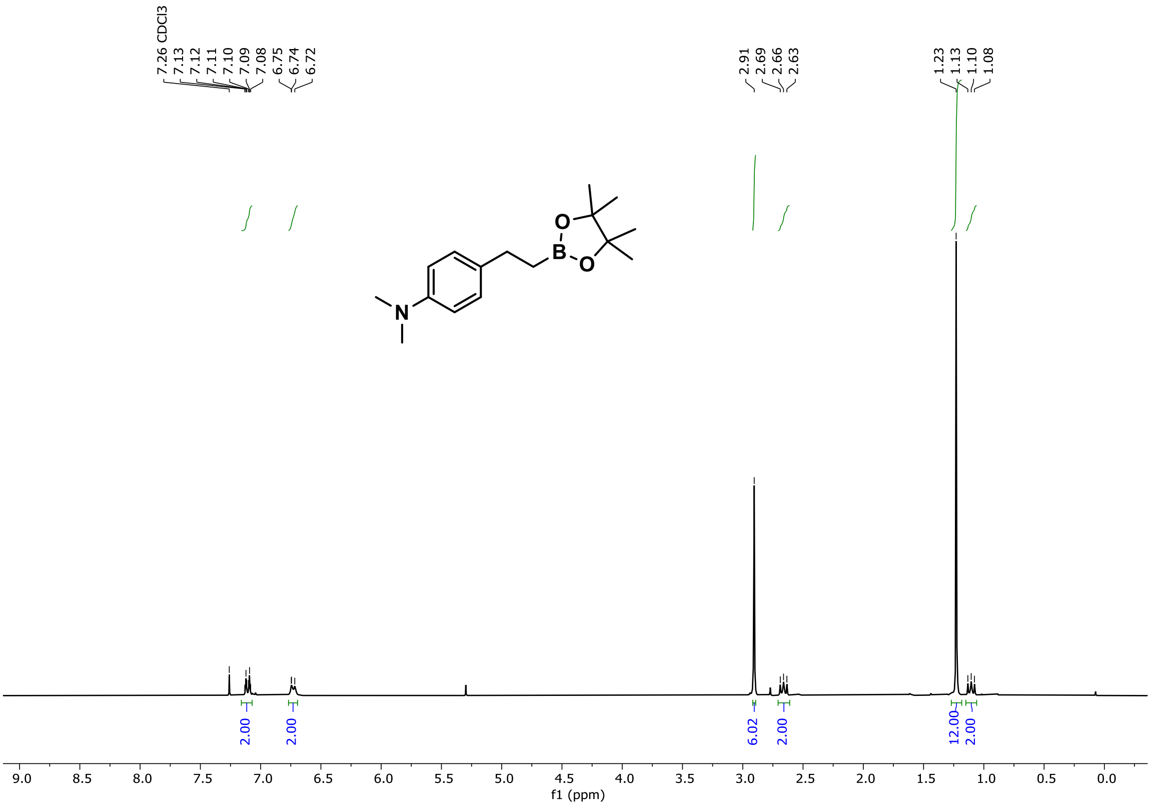

Figure S47. ^{1}H NMR spectrum of *N,N-dimethyl-4-(2-(4,4,5,5-tetramethyl-1,3,2-dioxaborolan-2-yl)ethyl)aniline* (**3w**).

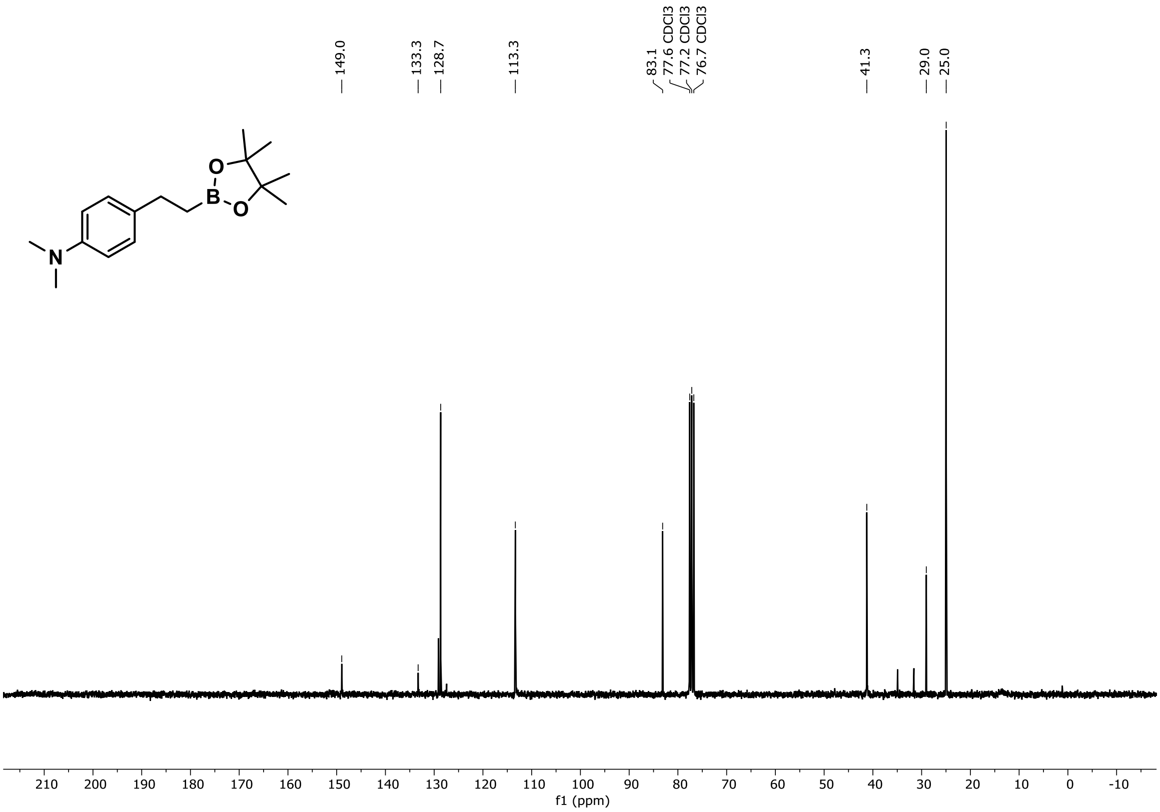

Figure S48. ^{13}C NMR spectrum of *N,N-dimethyl-4-(2-(4,4,5,5-tetramethyl-1,3,2-dioxaborolan-2-yl)ethyl)aniline* (**3w**).

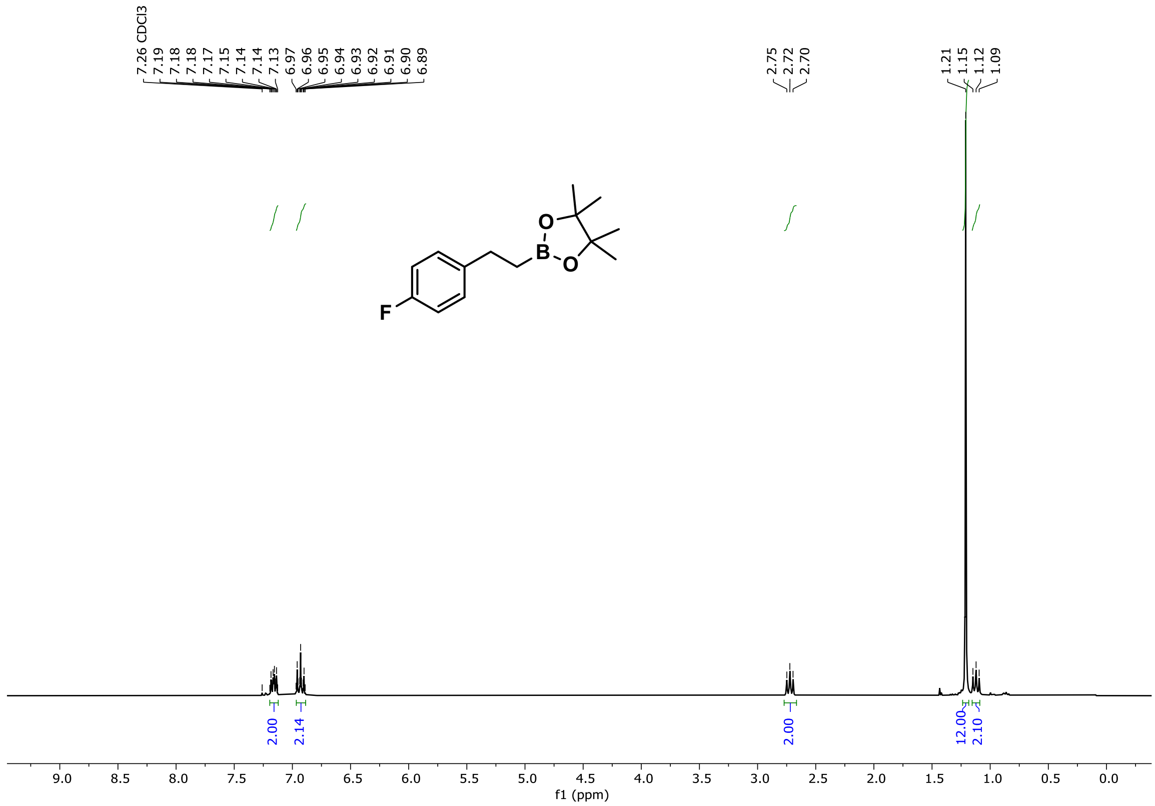

Figure S49. ^{1}H NMR spectrum of *2-(4-fluorophenethyl)-4,4,5,5-tetramethyl-1,3,2-dioxaborolane* (**3x**).

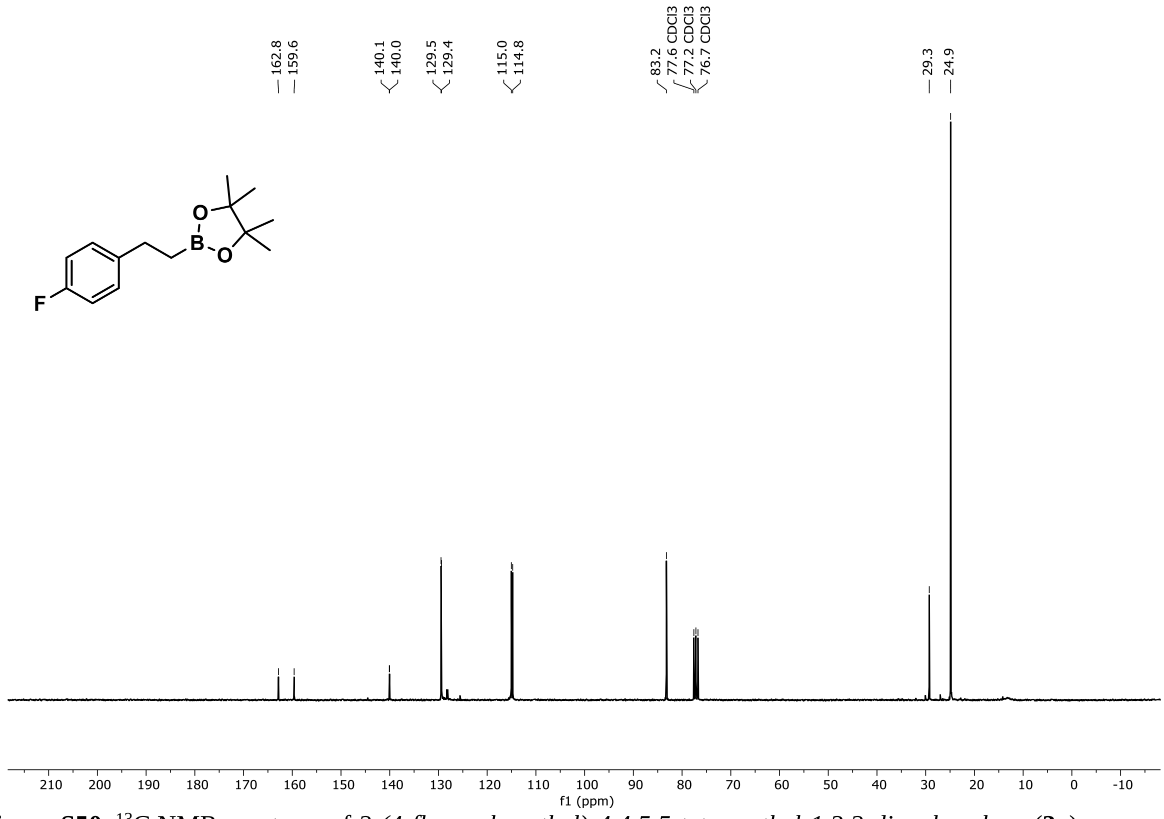

Figure S50. ^{13}C NMR spectrum of *2-(4-fluorophenethyl)-4,4,5,5-tetramethyl-1,3,2-dioxaborolane* (**3x**).

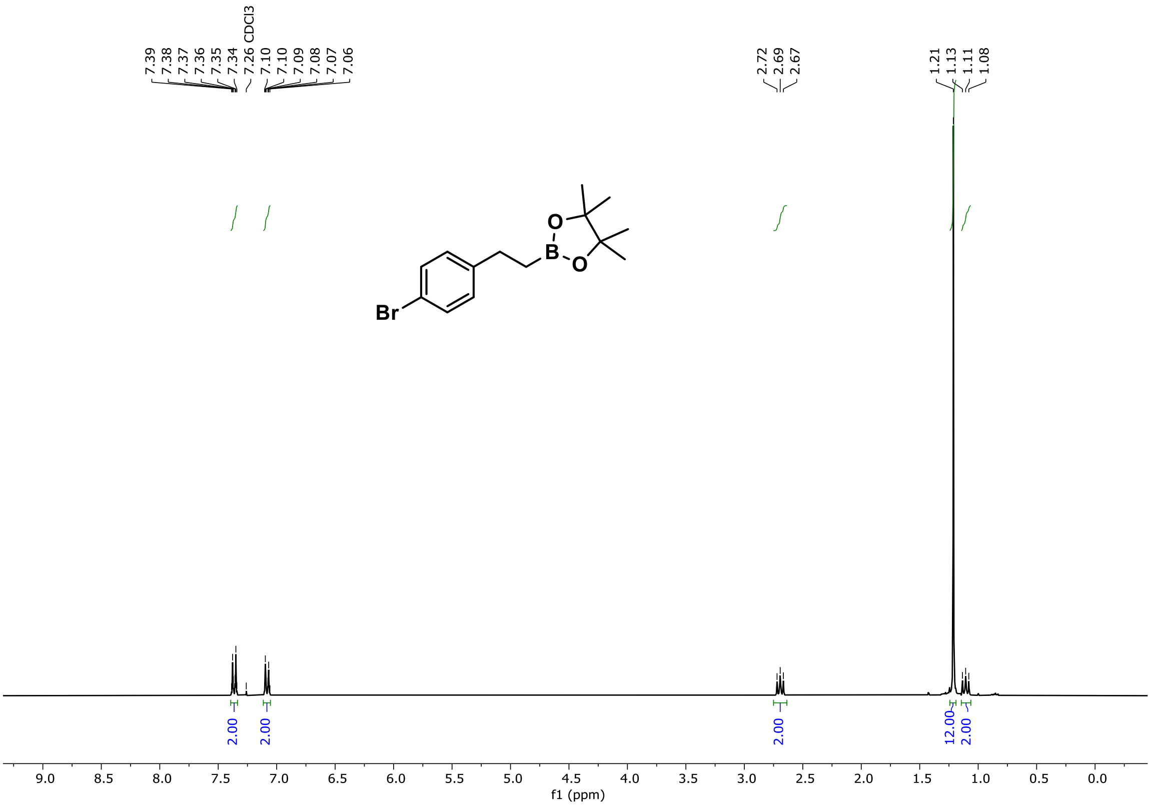

Figure S51. ^{1}H NMR spectrum of *2-(4-bromophenethyl)-4,4,5,5-tetramethyl-1,3,2-dioxaborolane* (**3y**).

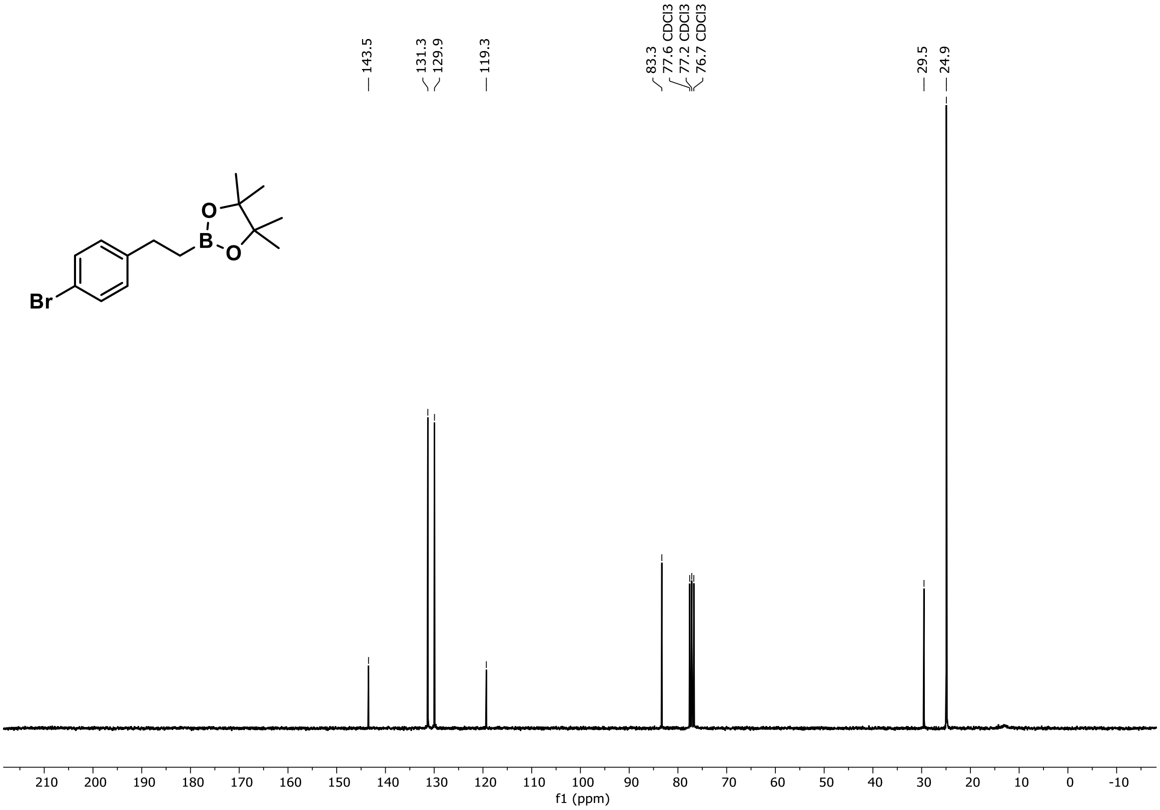

Figure S52. ^{13}C NMR spectrum of *2-(4-bromophenethyl)-4,4,5,5-tetramethyl-1,3,2-dioxaborolane* (**3y**).

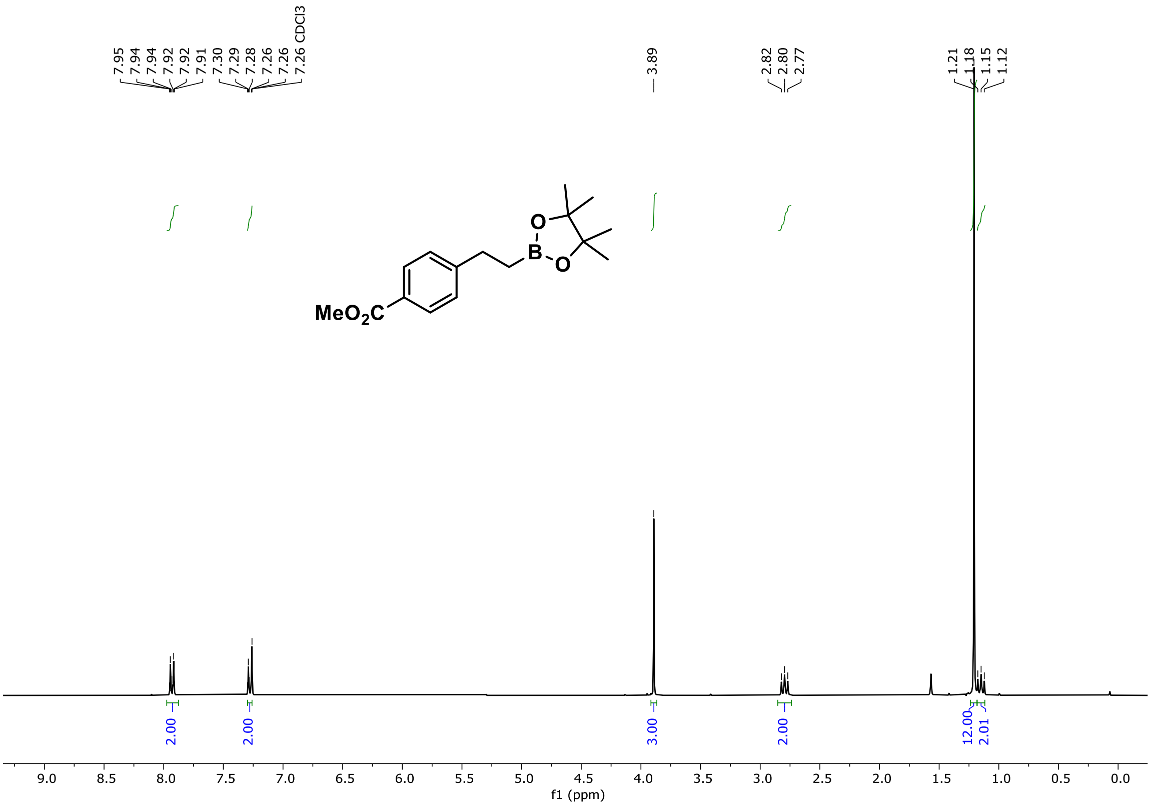

Figure S53. ^{1}H NMR spectrum of *methyl 4-[2-(4,4,5,5-tetramethyl-1,3,2-dioxaborolan-2-yl)ethyl]benzoate* (**3z**).

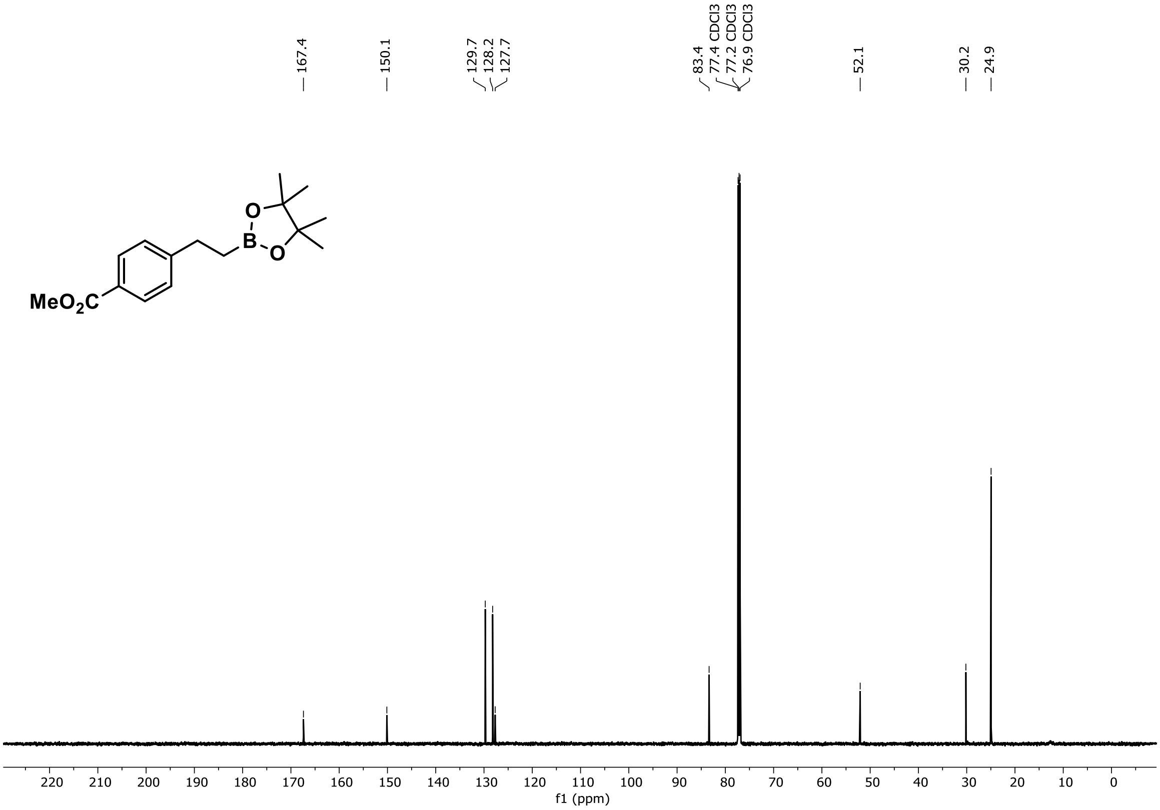

Figure S54. ^{13}C NMR spectrum of *methyl 4-[2-(4,4,5,5-tetramethyl-1,3,2-dioxaborolan-2-yl)ethyl]benzoate* (**3z**).

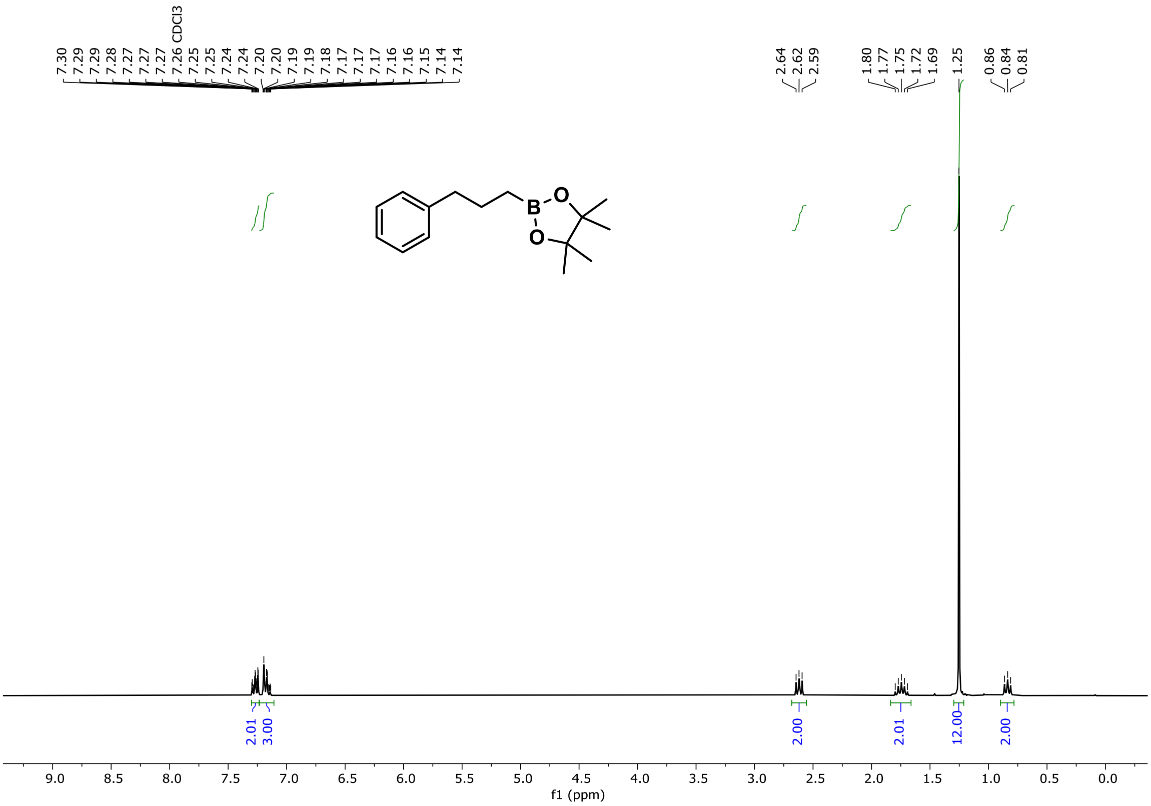

Figure S55. ^{1}H NMR spectrum of *4,4,5,5-tetramethyl-2-[3-(2-methylphenyl)propyl]-1,3,2-dioxaborolane* (**3aa**).

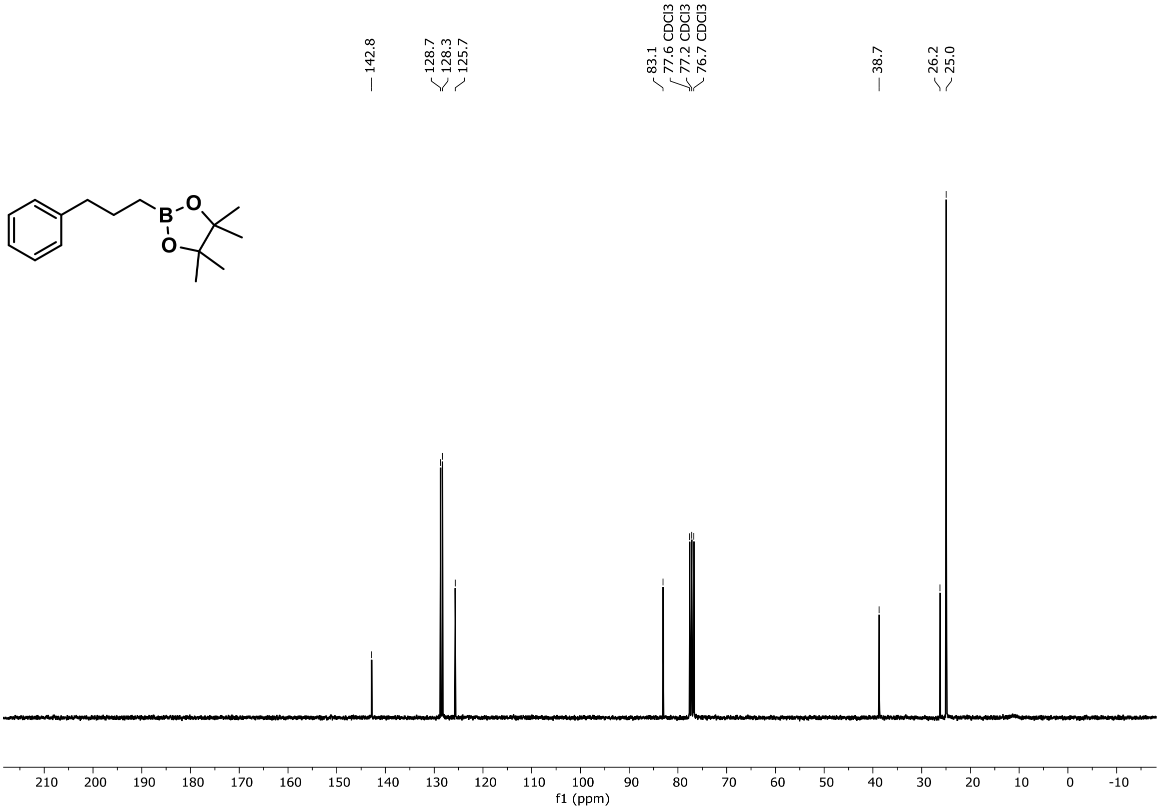

Figure S56. ^{13}C NMR spectrum of *4,4,5,5-tetramethyl-2-[3-(2-methylphenyl)propyl]-1,3,2-dioxaborolane* (**3aa**).

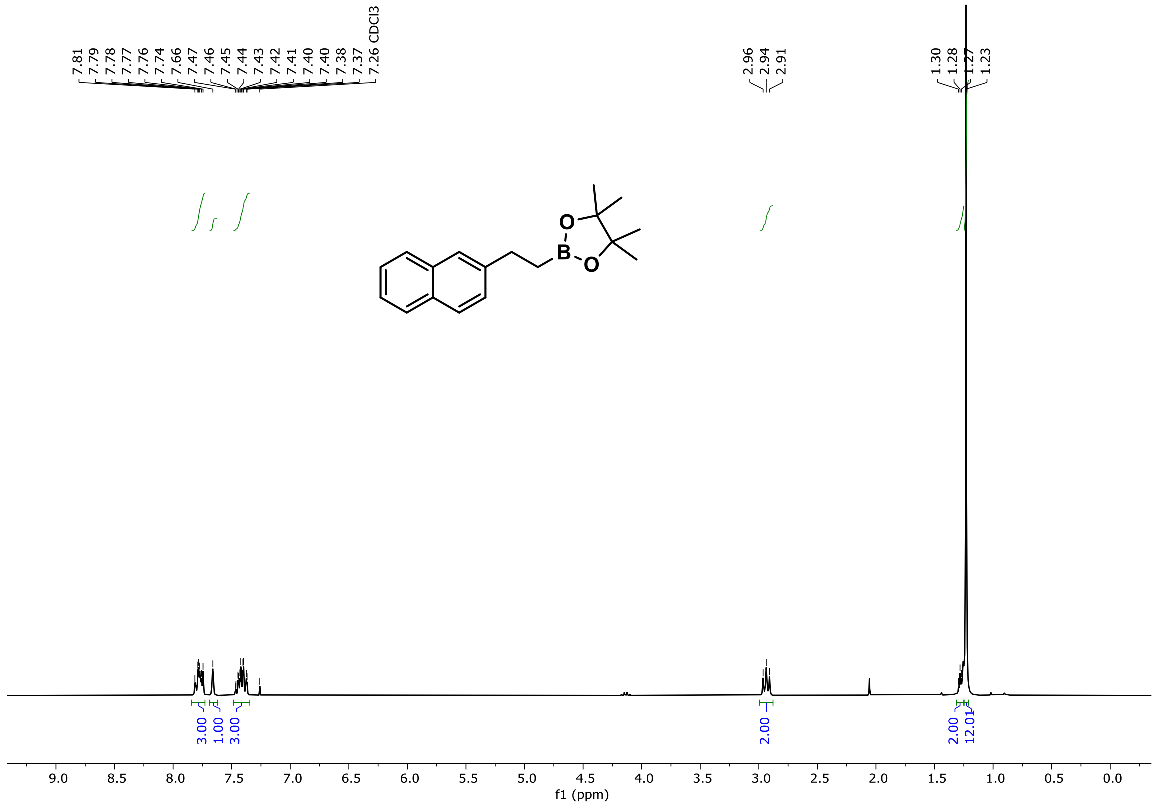

Figure S57. ^{1}H NMR spectrum of *2-(2-(naphthalen-2-yl)ethyl)-4,4,5,5-tetramethyl-1,3,2-dioxaborolane* (**3ab**).

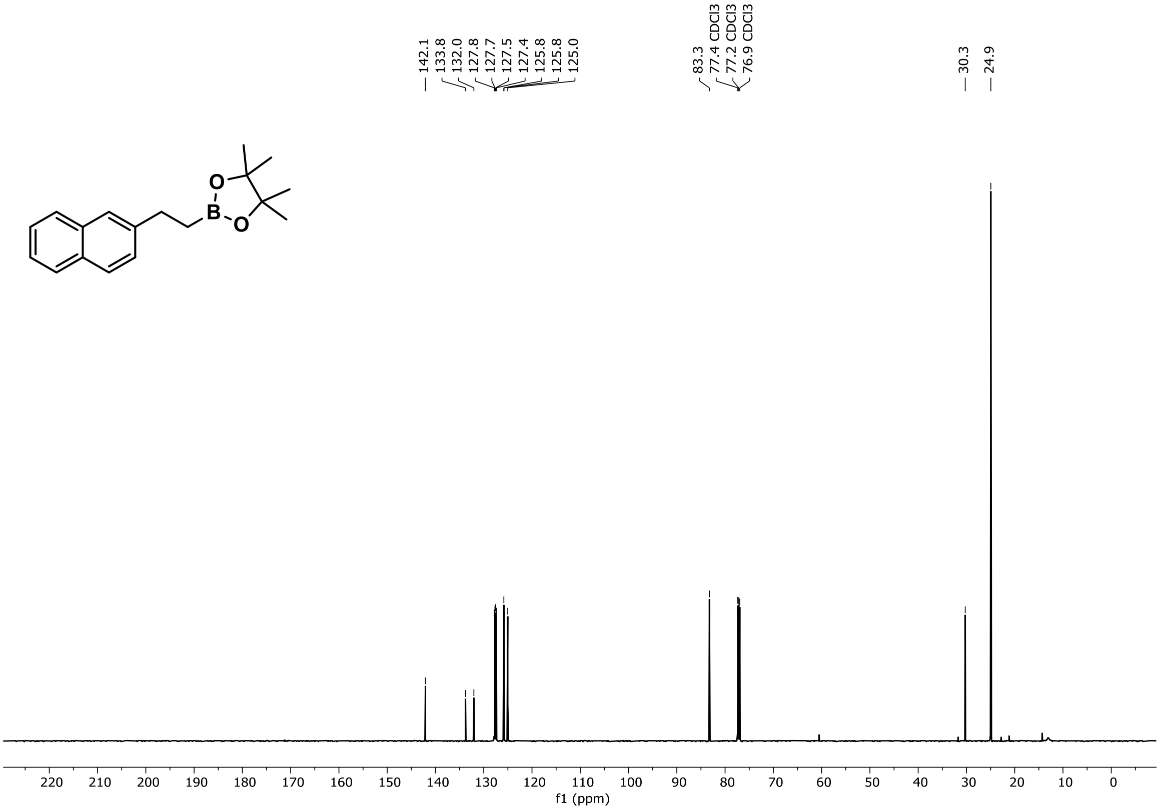

Figure S58. ^{13}C NMR spectrum of *2-(2-(naphthalen-2-yl)ethyl)-4,4,5,5-tetramethyl-1,3,2-dioxaborolane* (**3ab**).

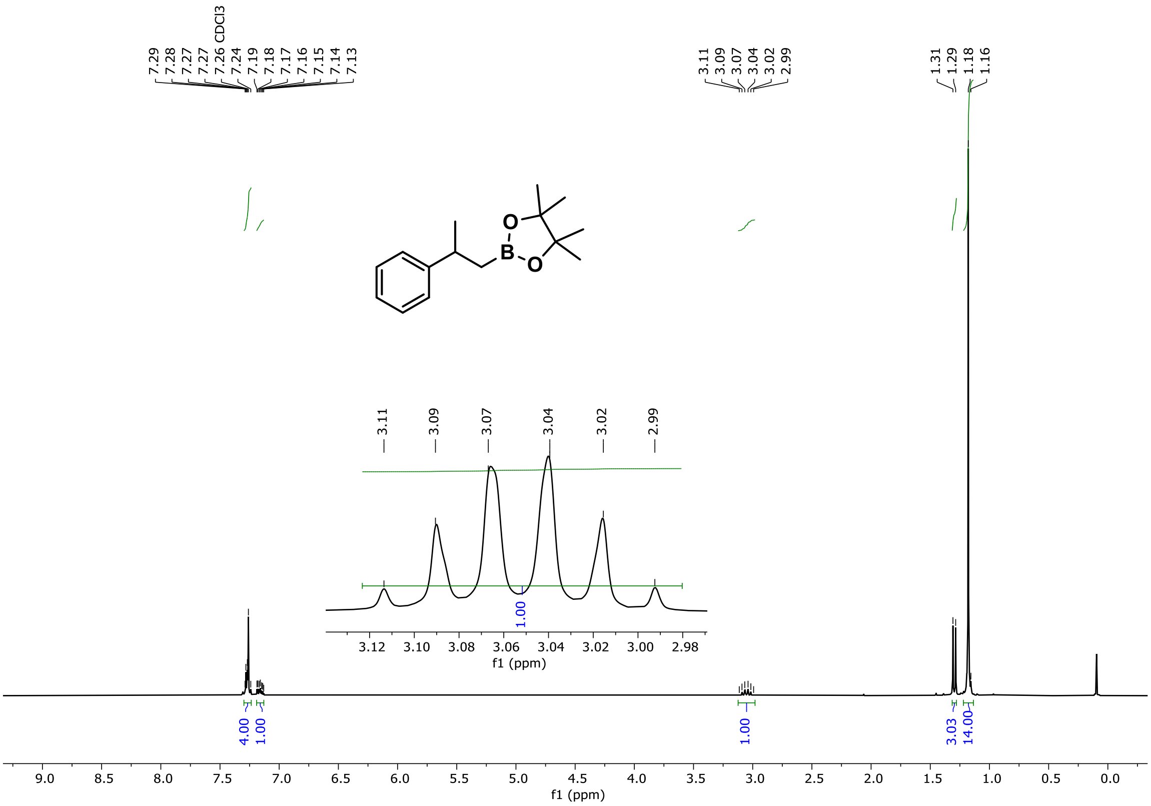

Figure S59. ^{1}H NMR spectrum of *4,4,5,5-tetramethyl-2-(2-phenylpropyl)-1,3,2-dioxaborolane* (**3j**).

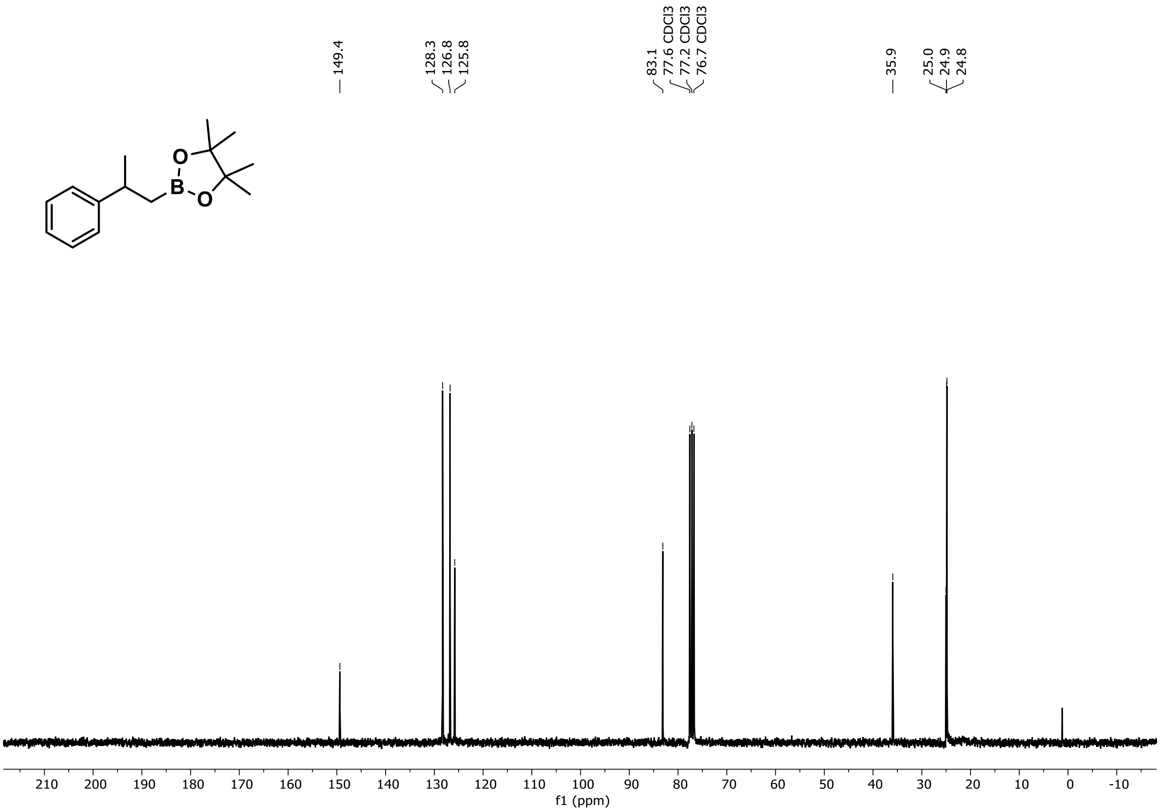

Figure S60. ^{13}C NMR spectrum of *4,4,5,5-tetramethyl-2-(2-phenylpropyl)-1,3,2-dioxaborolane* (**3j**).

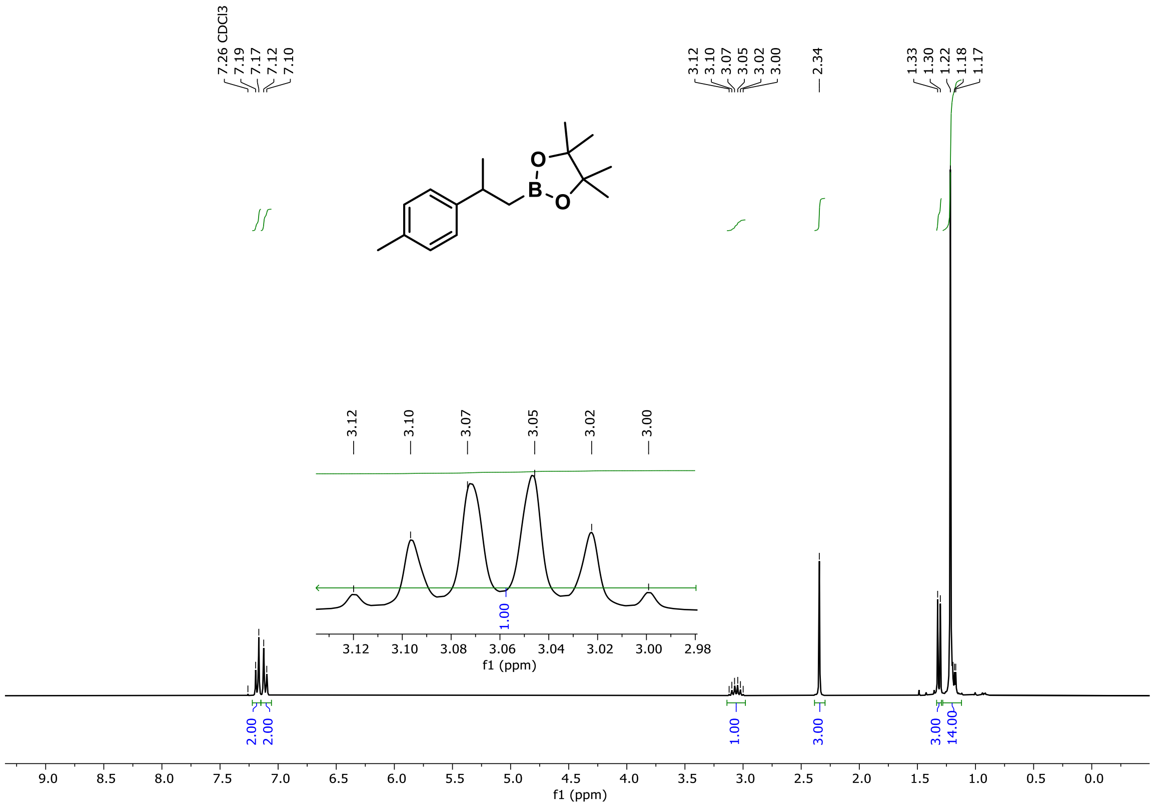

Figure S61. ^{1}H NMR spectrum of *4,4,5,5-tetramethyl-2-(2-(p-tolyl)propyl)-1,3,2-dioxaborolane* (**3k**).

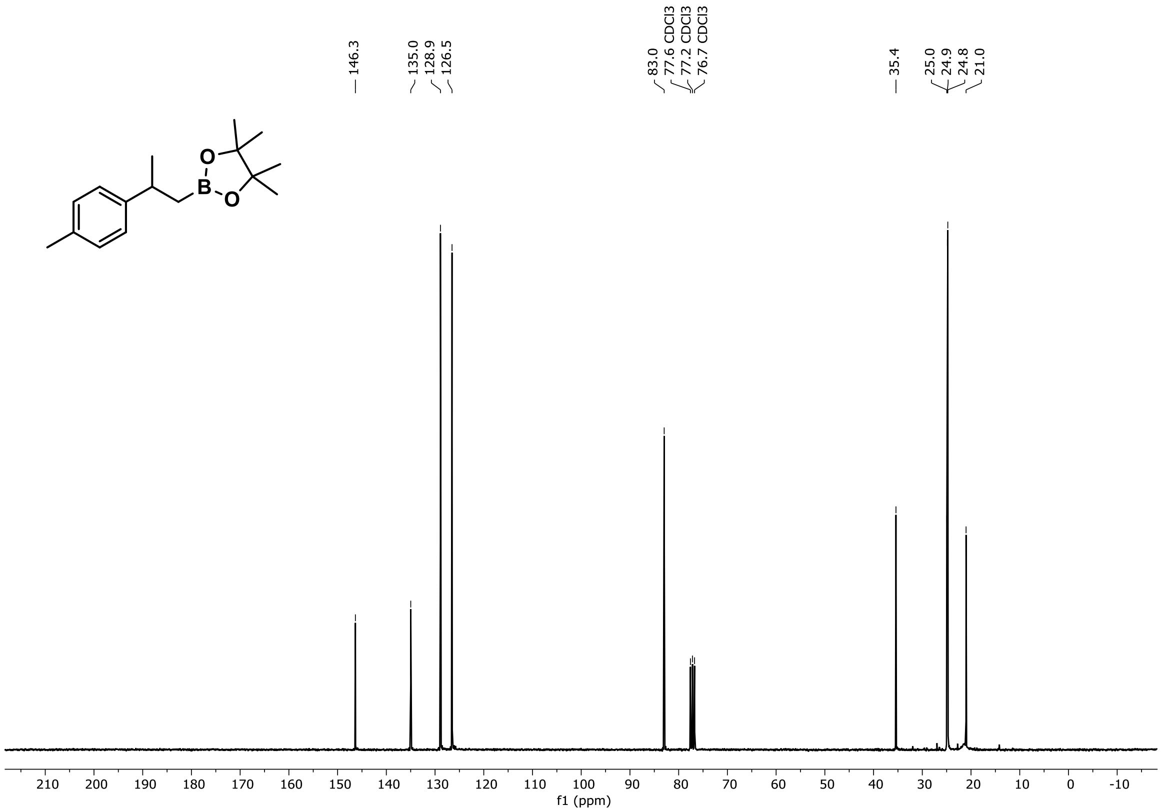

Figure S62. ^{13}C NMR spectrum of *4,4,5,5-tetramethyl-2-(2-(p-tolyl)propyl)-1,3,2-dioxaborolane* (**3k**).

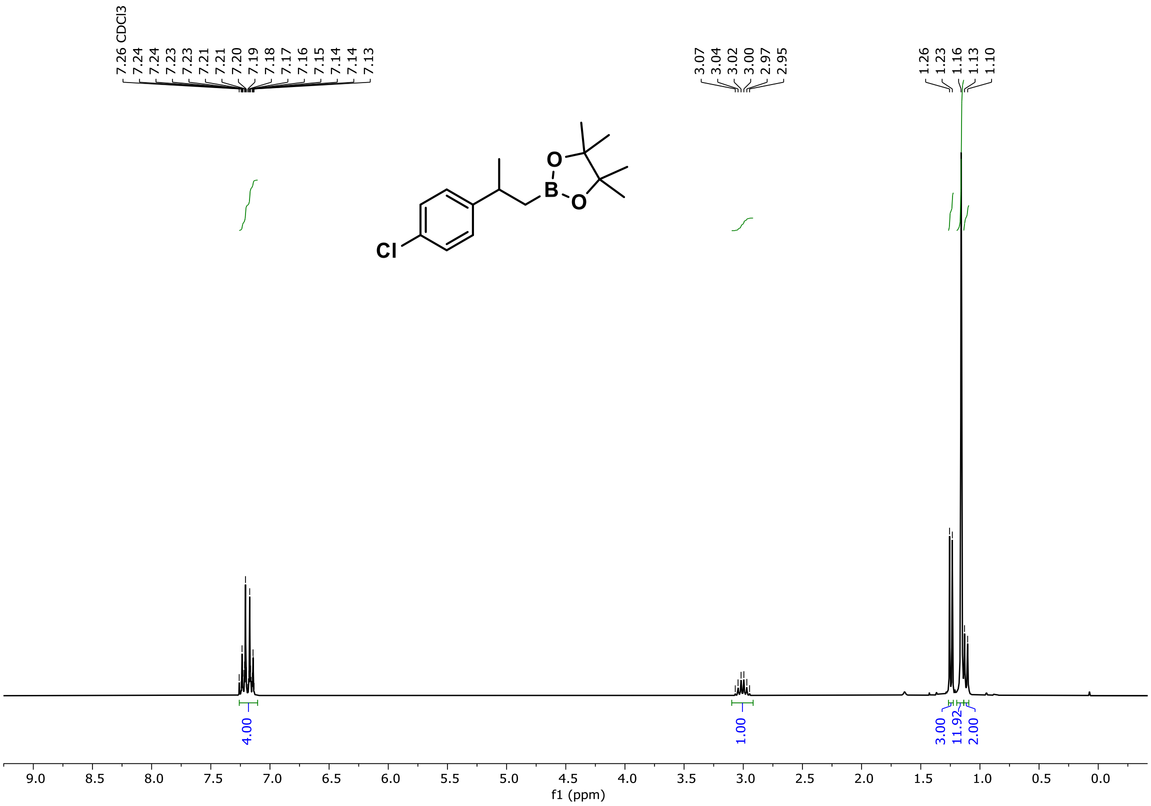

Figure S63. ^{1}H NMR spectrum of *2-(2-(4-chlorophenyl)propyl)-4,4,5,5-tetramethyl-1,3,2-dioxaborolane* (**3ac**).

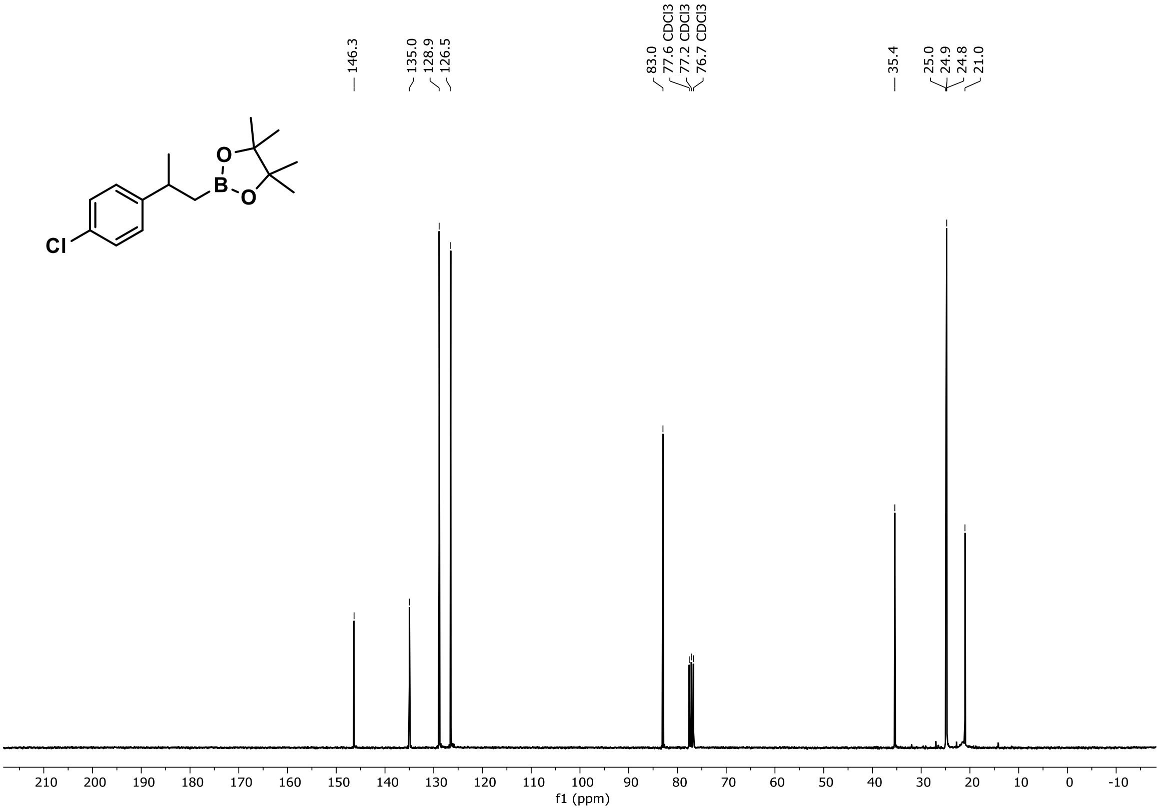

Figure S64. ^{13}C NMR spectrum of *2-(2-(4-chlorophenyl)propyl)-4,4,5,5-tetramethyl-1,3,2-dioxaborolane* (**3ac**).

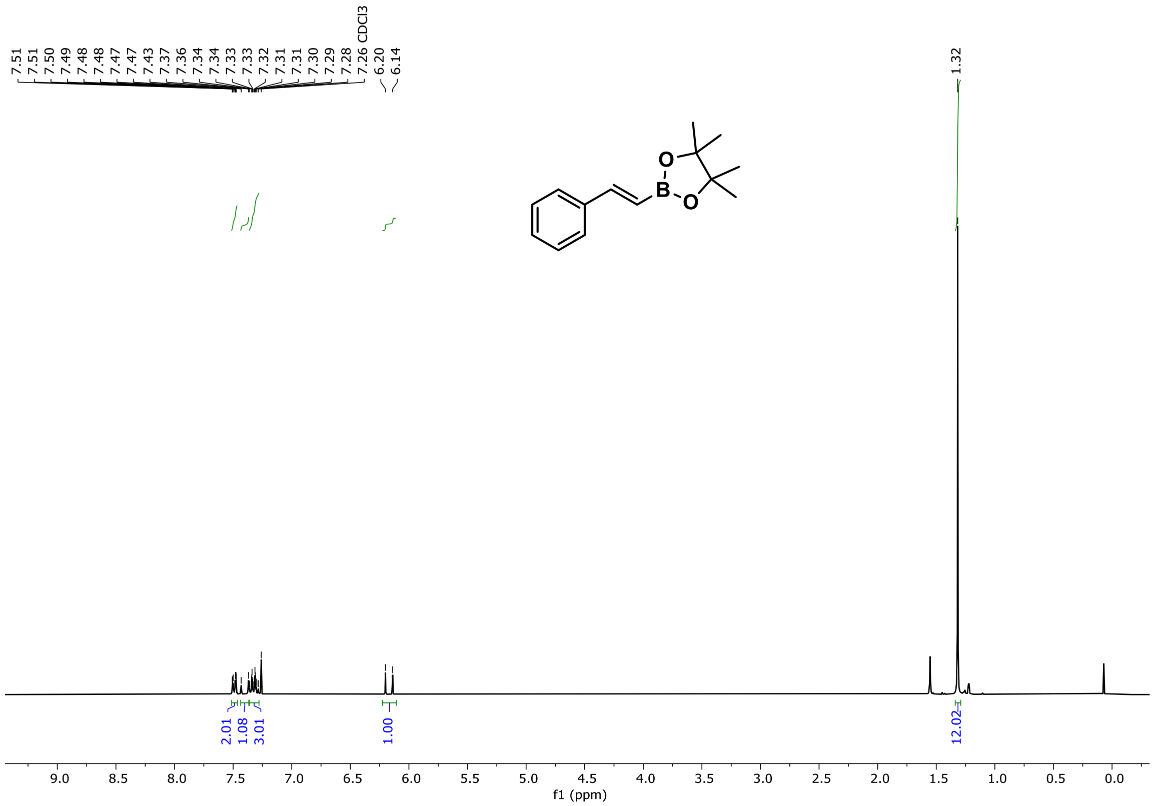

Figure S65. ^{1}H NMR spectrum of *(E)-4,4,5,5-tetramethyl-2-styryl-1,3,2-dioxaborolane* (**8a**).

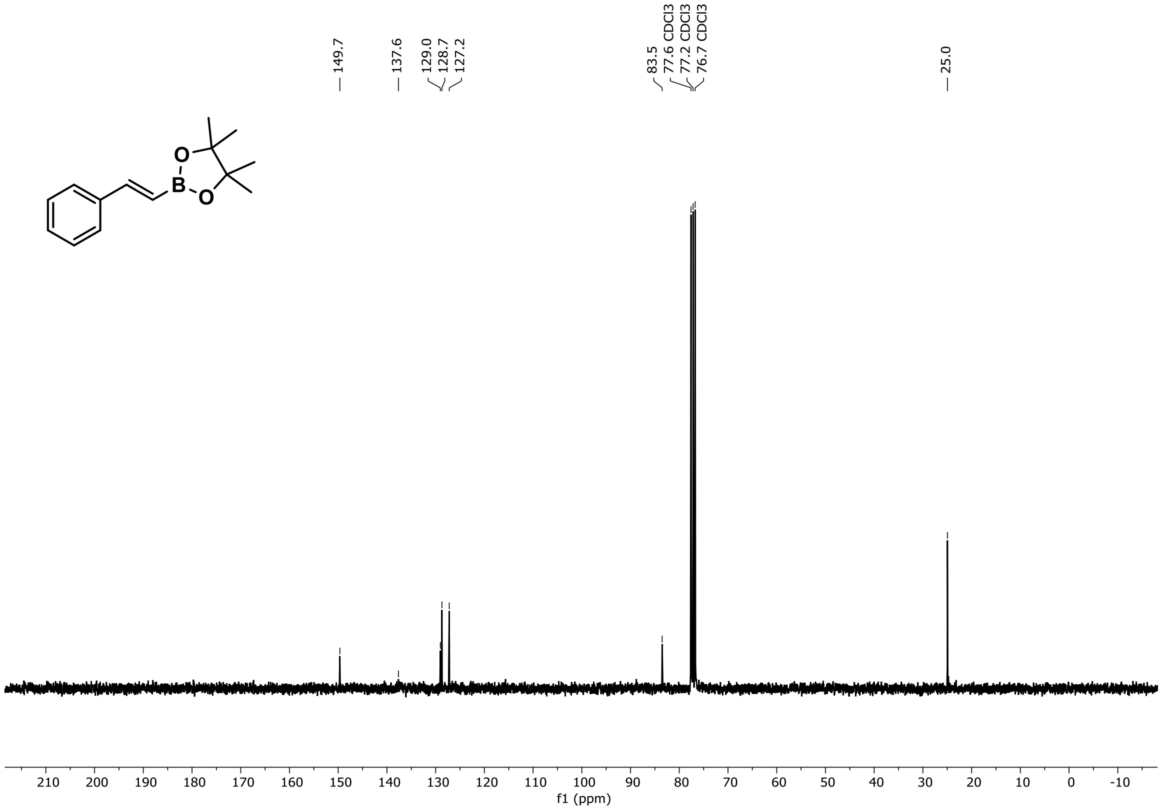

Figure S66. ^{13}C NMR spectrum of *(E)-4,4,5,5-tetramethyl-2-styryl-1,3,2-dioxaborolane* (**8a**).

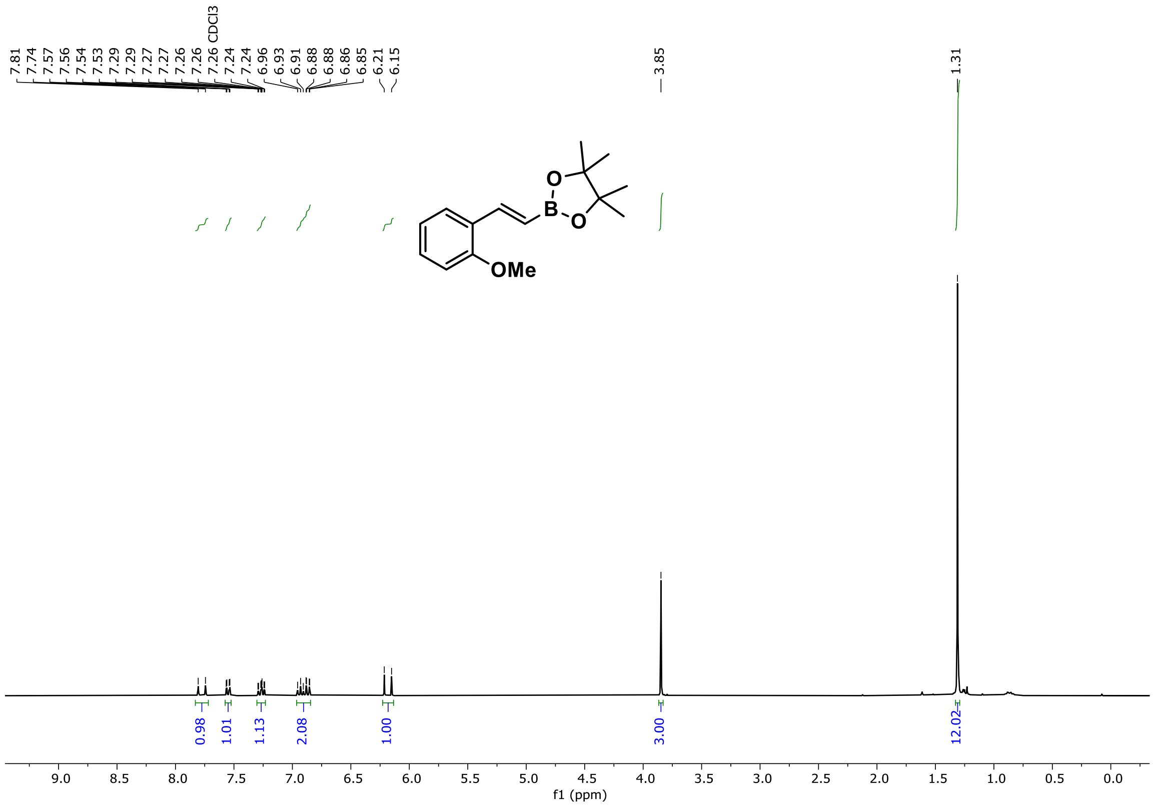

Figure S67. ^{1}H NMR spectrum of *(E)-2-(2-methoxystyryl)-4,4,5,5-tetramethyl-1,3,2-dioxaborolane* (**8h**).

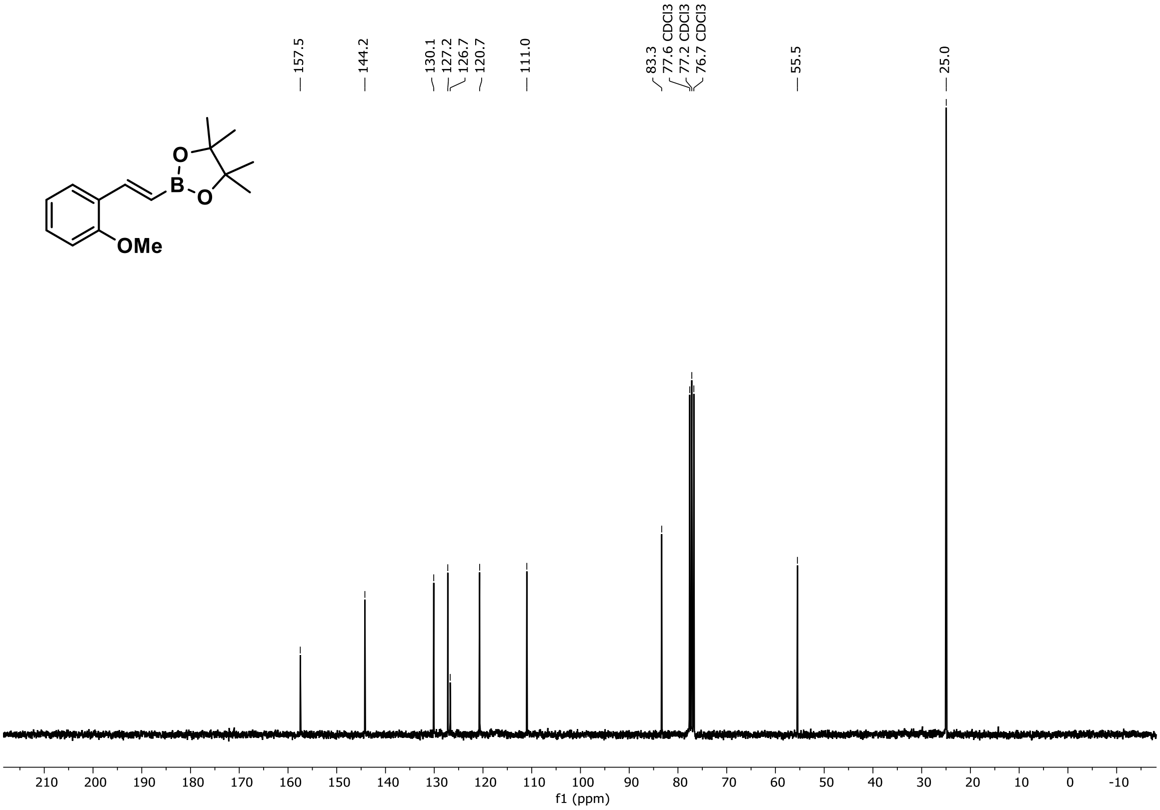

Figure S68. ^{13}C NMR spectrum of *(E)-2-(2-methoxystyryl)-4,4,5,5-tetramethyl-1,3,2-dioxaborolane* (**8h**).

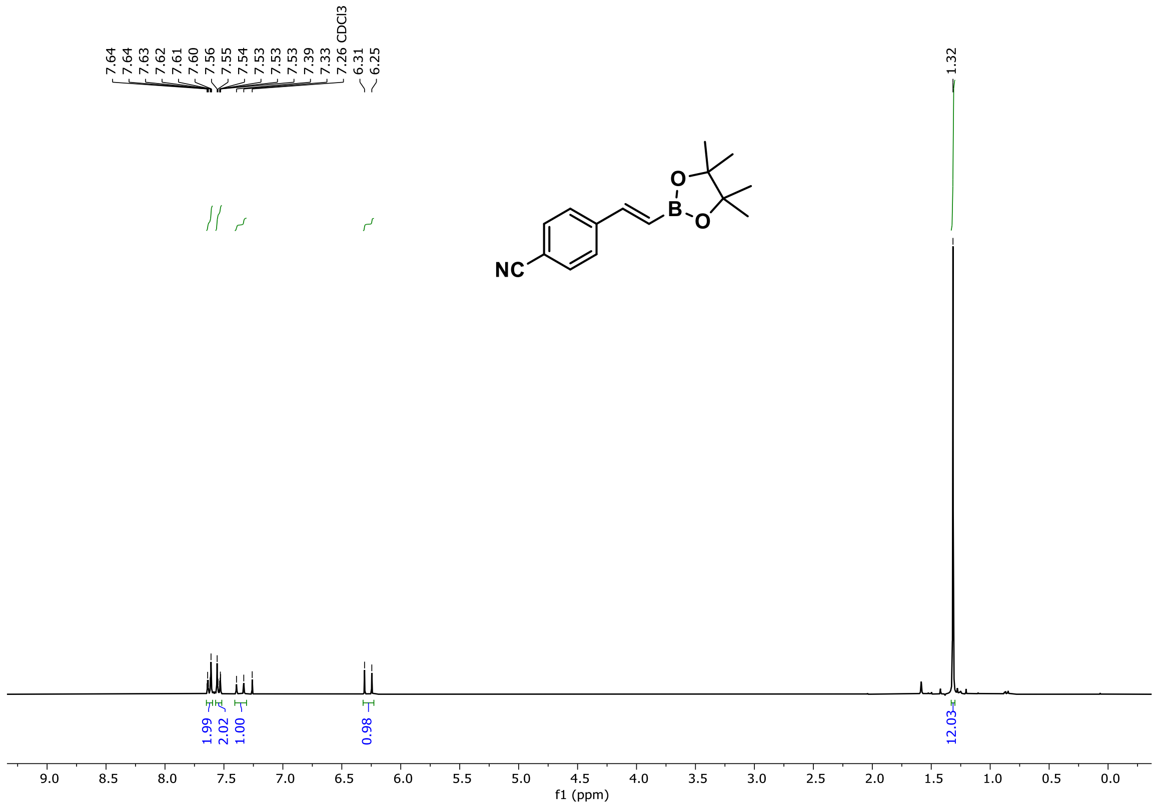

Figure S69. ^{1}H NMR spectrum of *(E)-4-(2-(4,4,5,5-tetramethyl-1,3,2-dioxaborolan-2-yl)vinyl)benzonitrile* (**8i**).

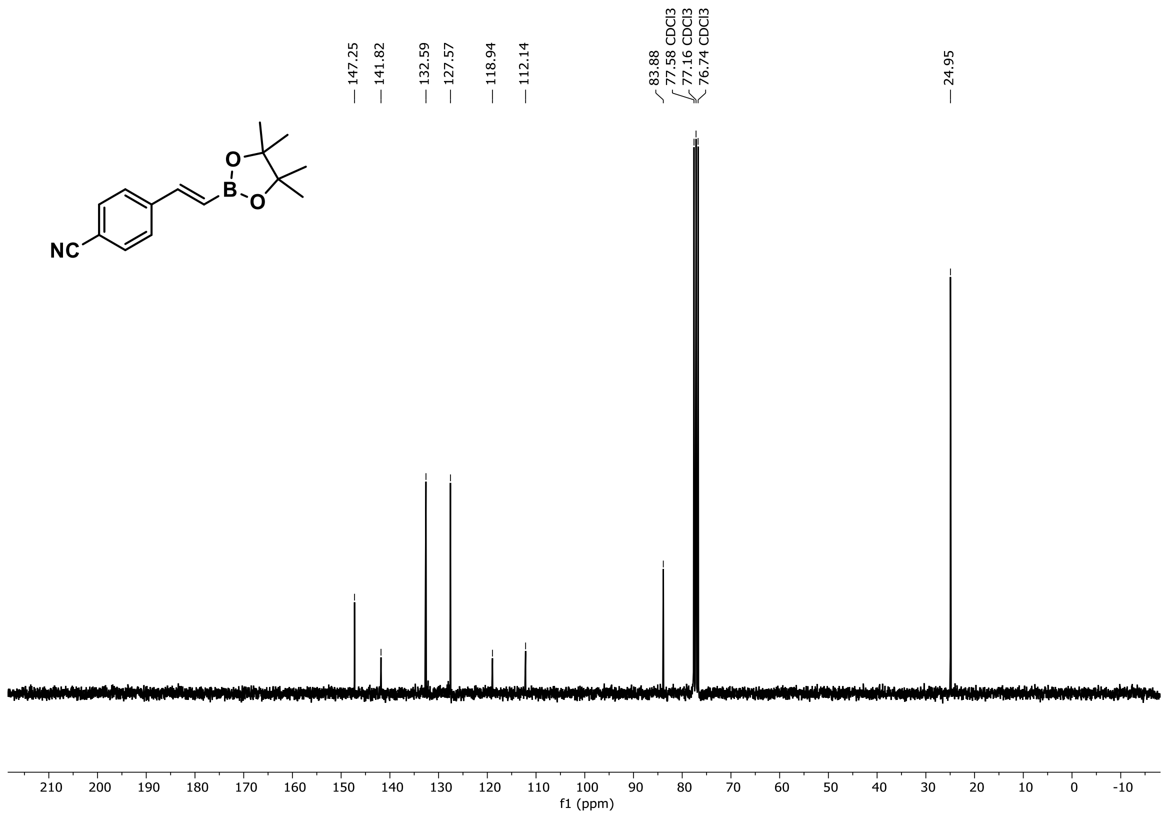

Figure S70. ^{13}C NMR spectrum of *(E)-4-(2-(4,4,5,5-tetramethyl-1,3,2-dioxaborolan-2-yl)vinyl)benzonitrile* (**8i**).

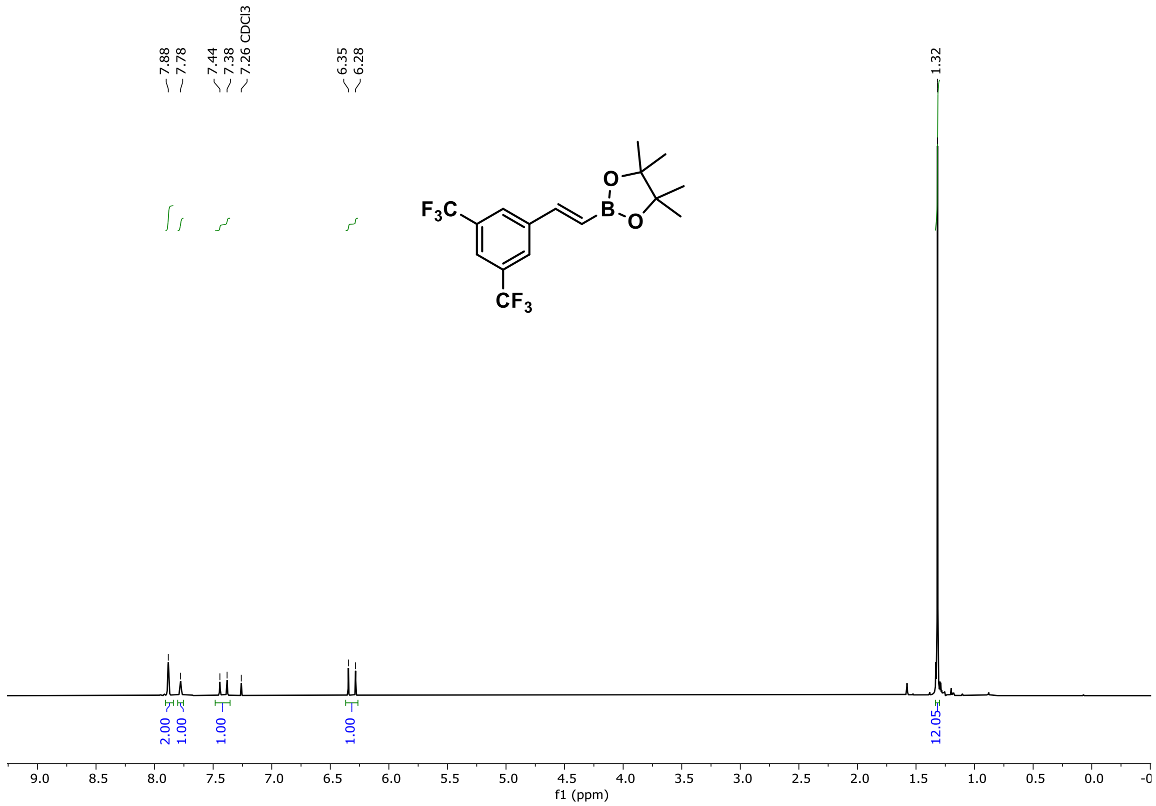

Figure S71. ^{1}H NMR spectrum of *(E)-2-(3,5-bis(trifluoromethyl)styryl)-4,4,5,5-tetramethyl-1,3,2-dioxaborolane* (**8j**).

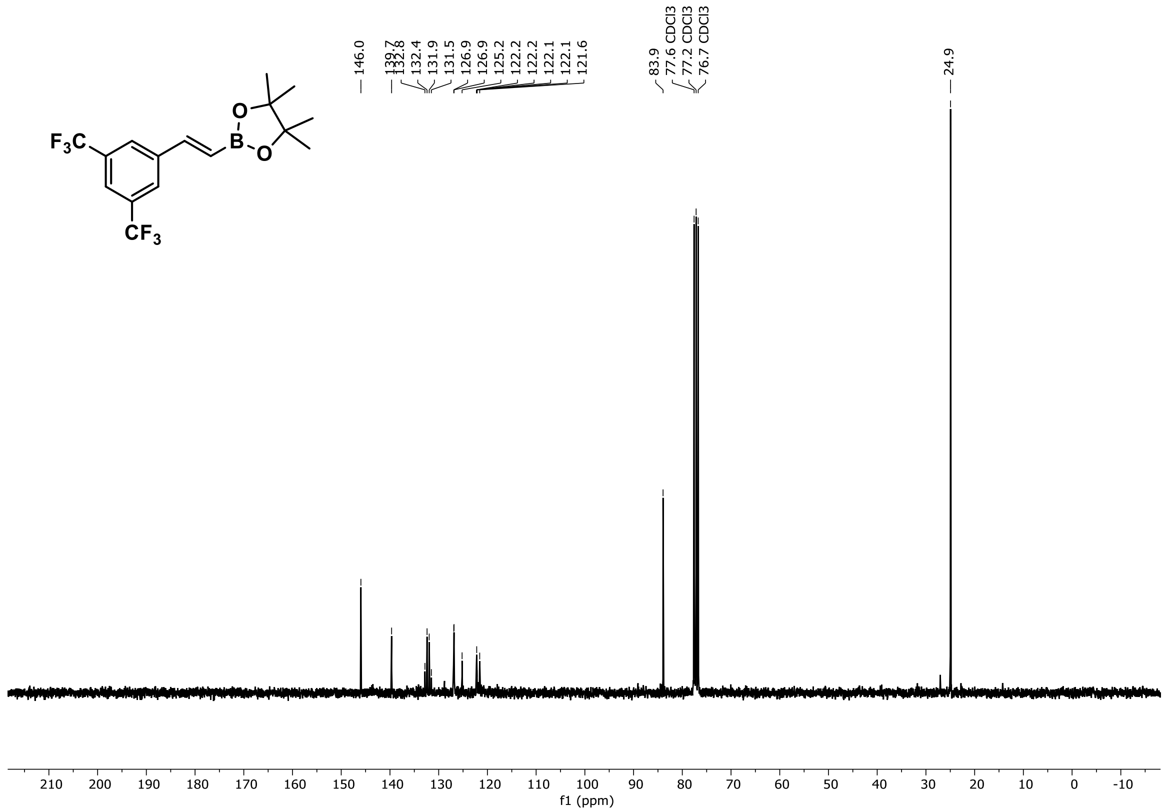

Figure S72. ^{13}C NMR spectrum of *(E)-2-(3,5-bis(trifluoromethyl)styryl)-4,4,5,5-tetramethyl-1,3,2-dioxaborolane* (**8j**).

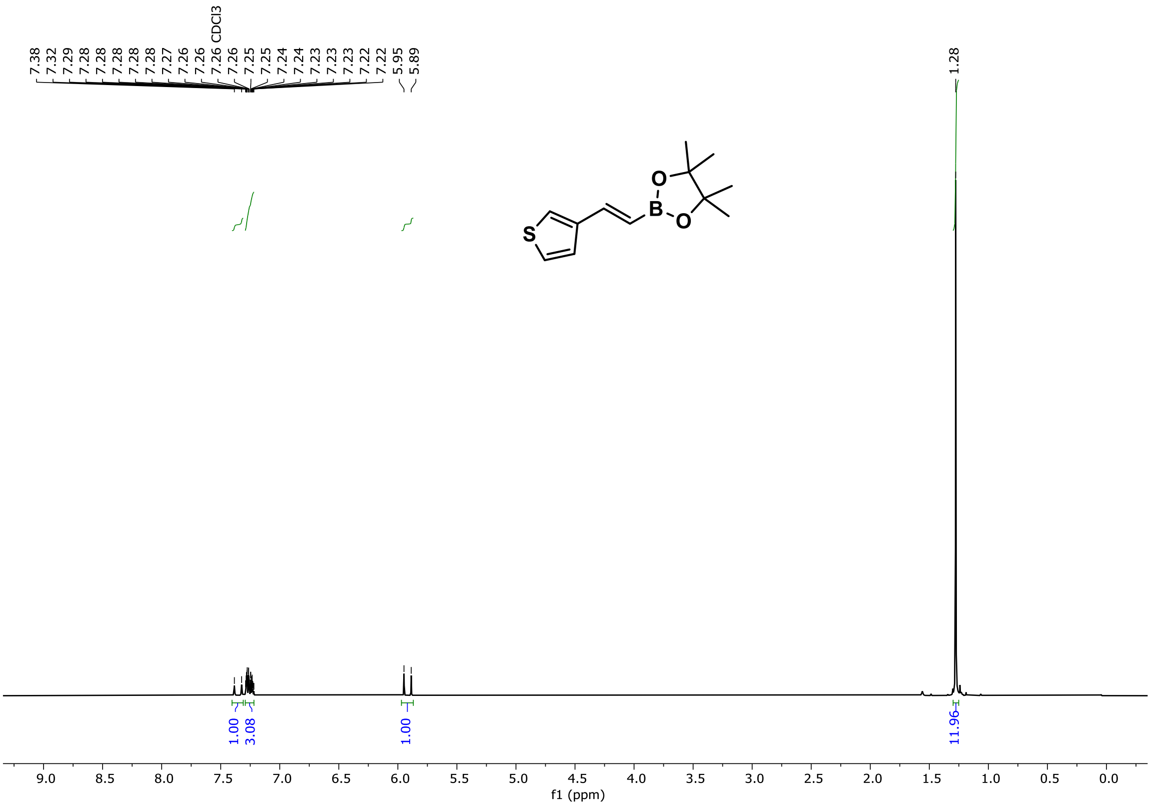

Figure S73. ^{1}H NMR spectrum of *(E)-4,4,5,5-tetramethyl-2-(2-(thiophen-3-yl)vinyl)-1,3,2-dioxaborolane* (**8k**).

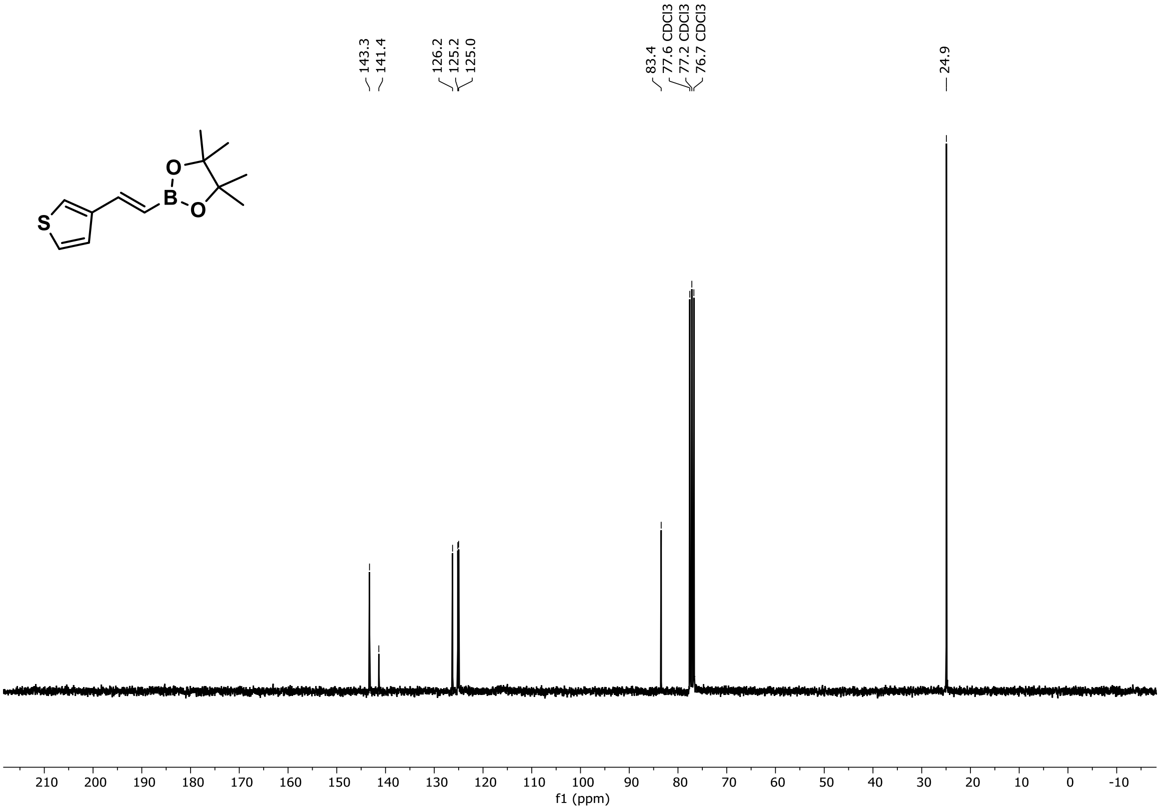

Figure S74. ^{13}C NMR spectrum of *(E)-4,4,5,5-tetramethyl-2-(2-(thiophen-3-yl)vinyl)-1,3,2-dioxaborolane* (**8k**).

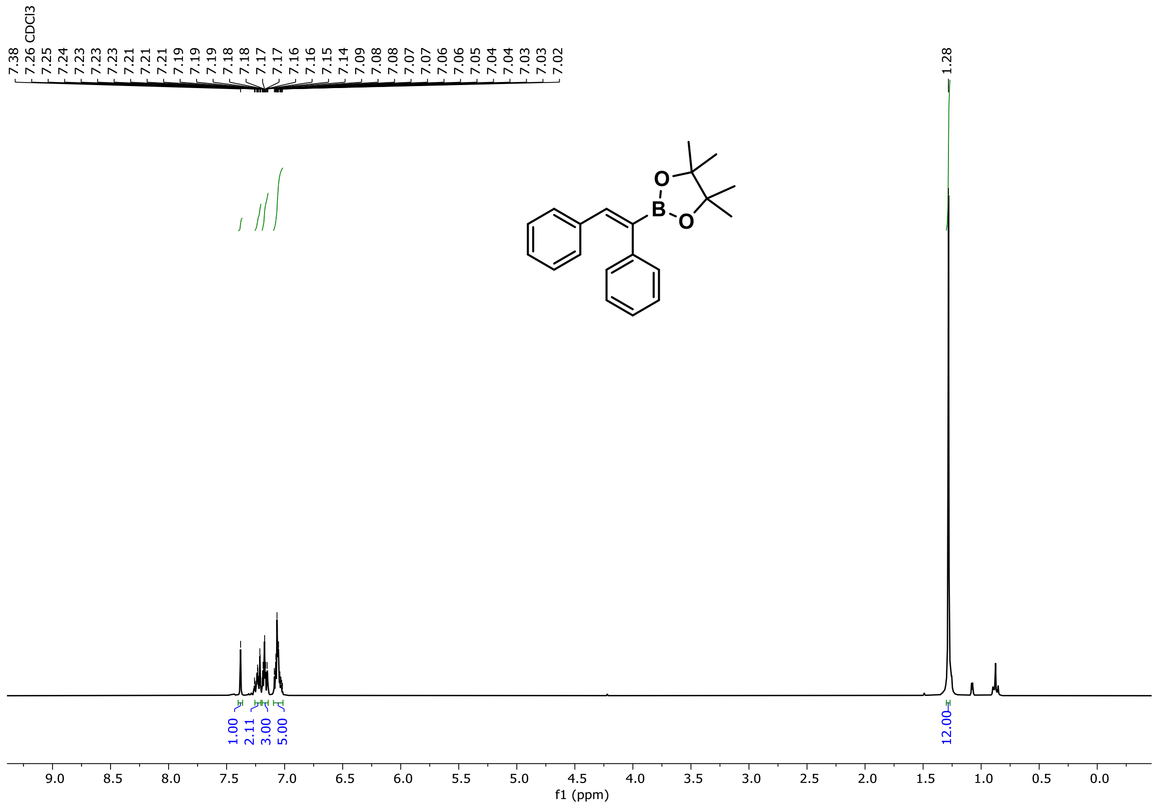

Figure S75. ^{1}H NMR spectrum of *(Z)-2-(1,2-diphenylvinyl)-4,4,5,5-tetramethyl-1,3,2-dioxaborolane* (**8f**).

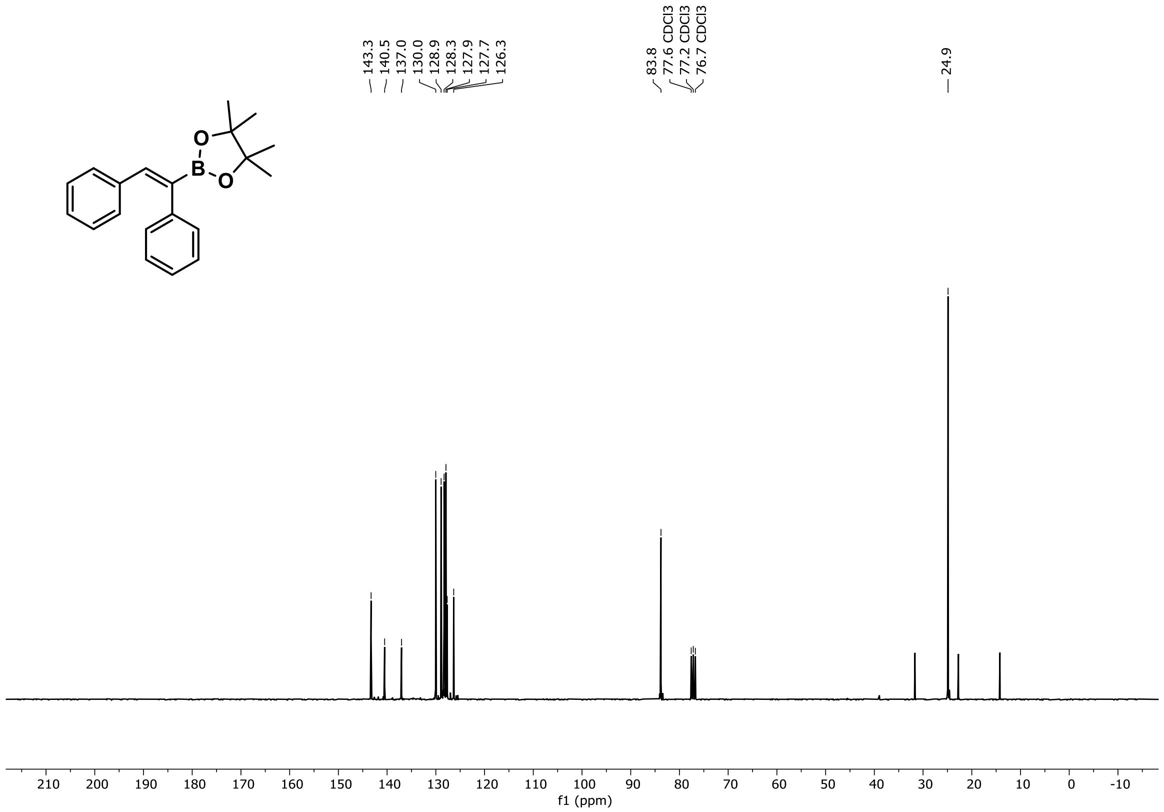

Figure S76. ^{13}C NMR spectrum of *(Z)-2-(1,2-diphenylvinyl)-4,4,5,5-tetramethyl-1,3,2-dioxaborolane* (**8e**).

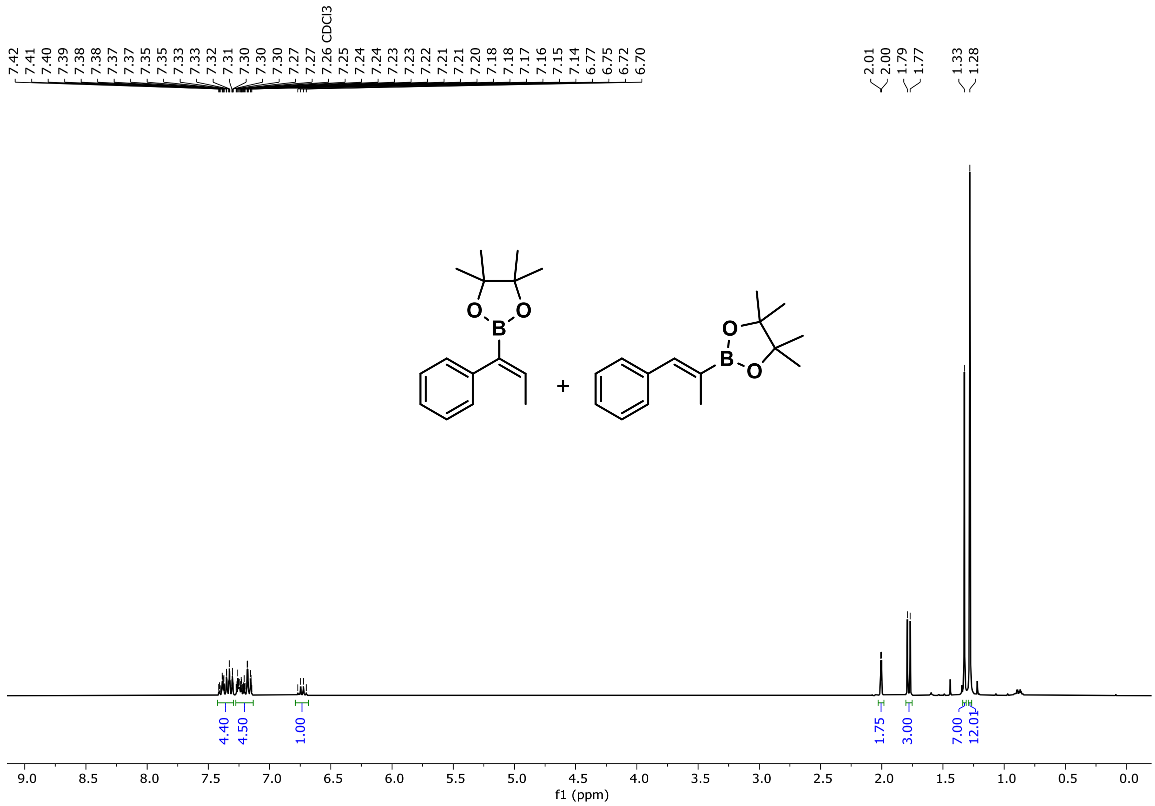

Figure S77. ^{1}H NMR spectrum of *(Z)-4,4,5,5-tetramethyl-2-(1-phenylprop-1-en-1-yl)-1,3,2-dioxaborolane* and *(Z)-4,4,5,5-tetramethyl-2-(1-phenylprop-1-en-2-yl)-1,3,2-dioxaborolane* (**8l**).

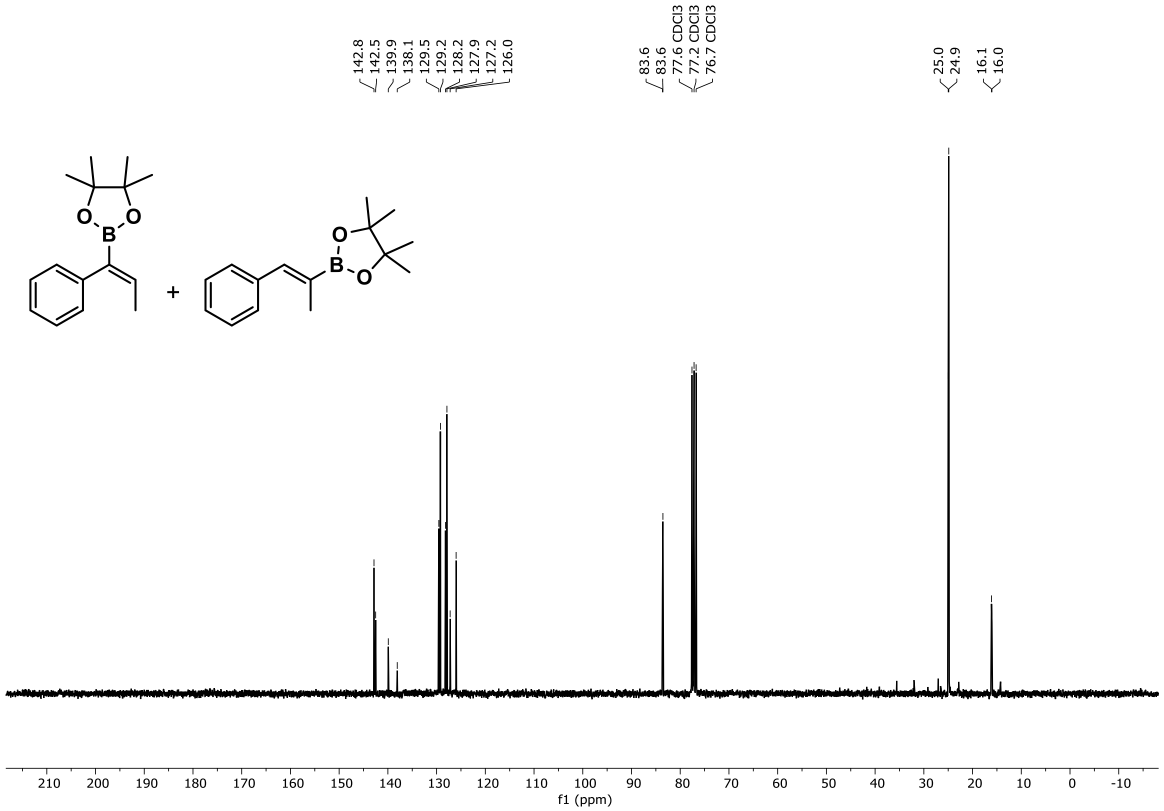

Figure S78. ^{13}C NMR spectrum of *(Z)-4,4,5,5-tetramethyl-2-(1-phenylprop-1-en-1-yl)-1,3,2-dioxaborolane* and *(Z)-4,4,5,5-tetramethyl-2-(1-phenylprop-1-en-2-yl)-1,3,2-dioxaborolane* (**8l**).

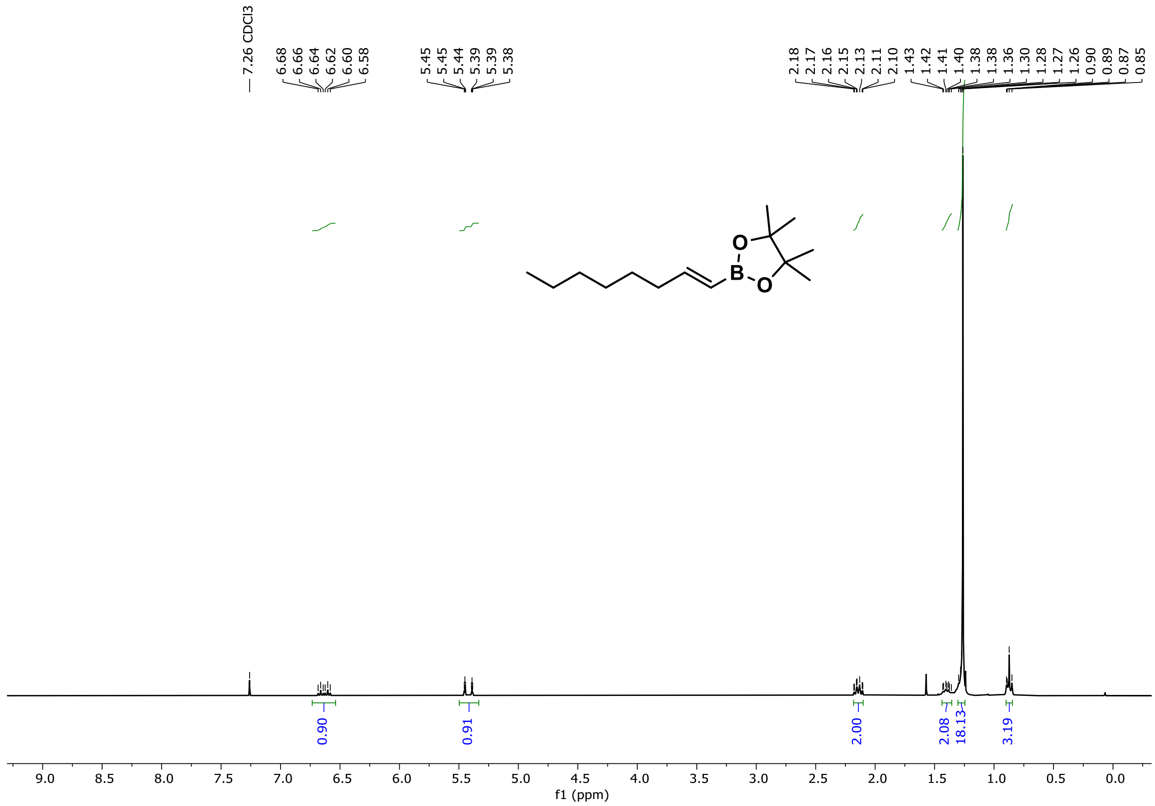

Figure S79. ^{1}H NMR spectrum of *(E)-4,4,5,5-tetramethyl-2-(oct-1-en-1-yl)-1,3,2-dioxaborolane* (**8e**).

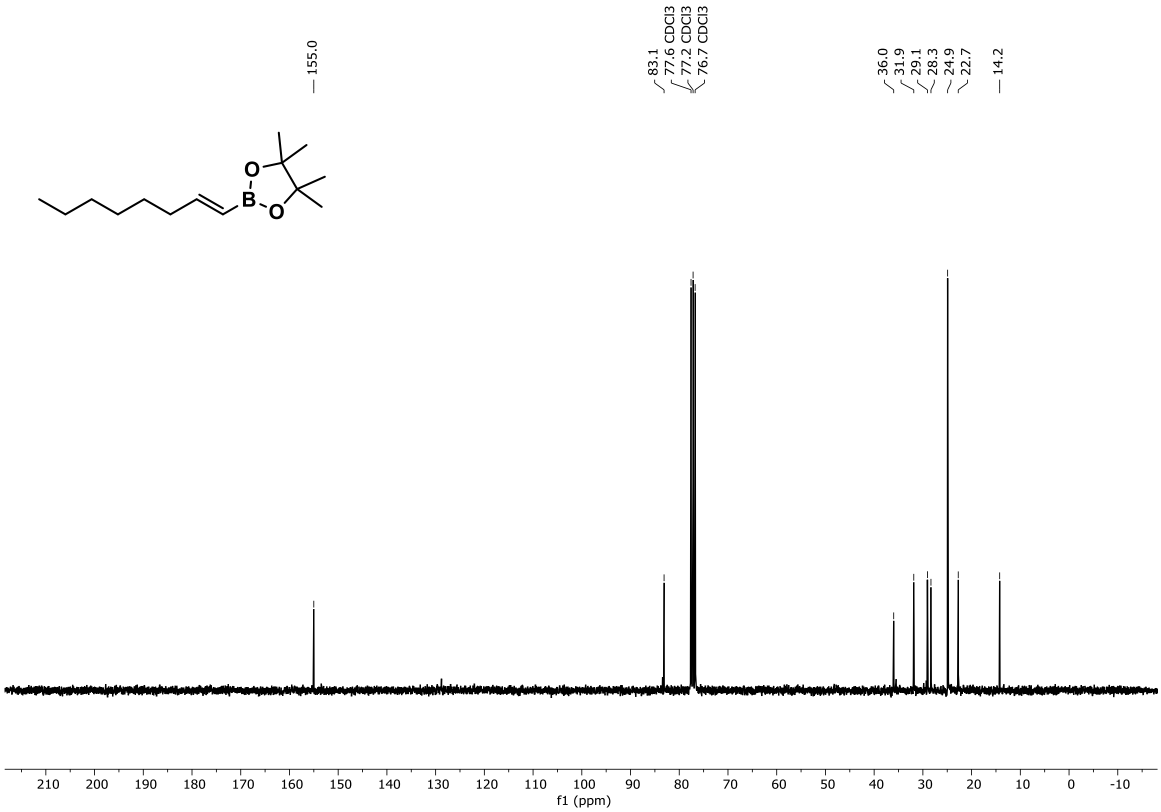

Figure S80. ^{13}C NMR spectrum of *(E)-4,4,5,5-tetramethyl-2-(oct-1-en-1-yl)-1,3,2-dioxaborolane* (**8e**).

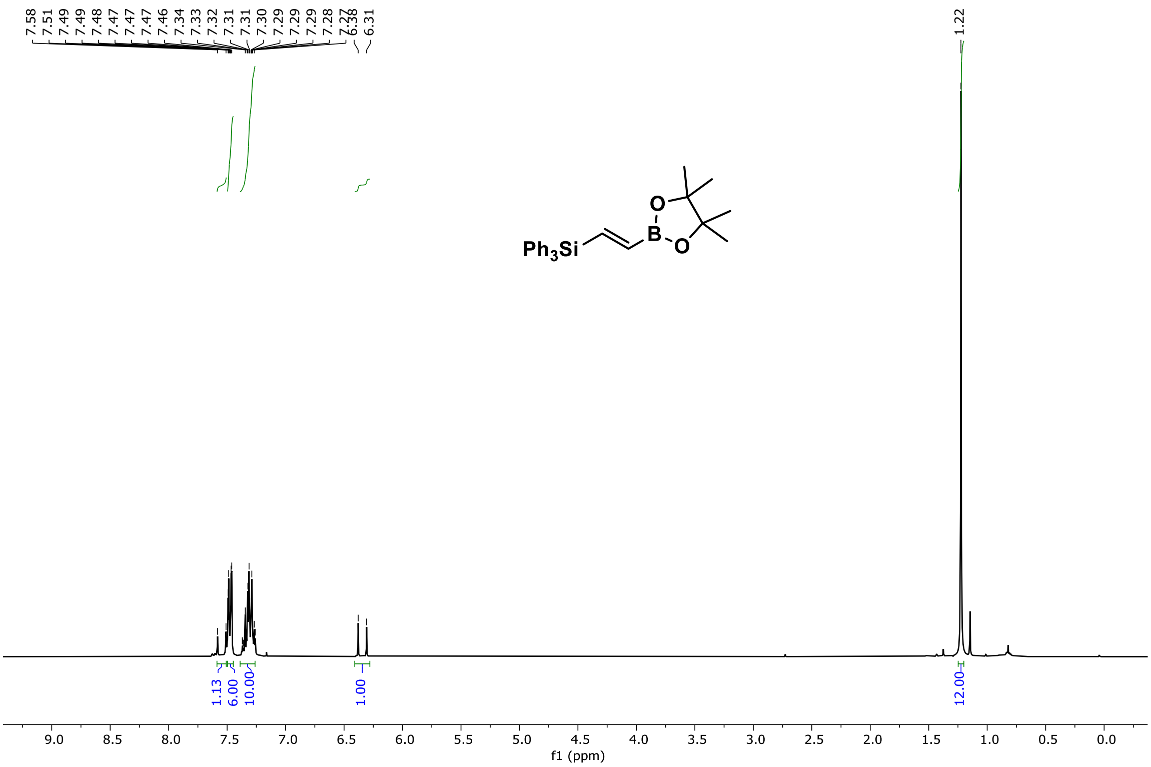

Figure S81. ^{1}H NMR spectrum of *(E)-triphenyl(2-(4,4,5,5-tetramethyl-1,3,2-dioxaborolan-2-yl)vinyl)silane* (**8m**).

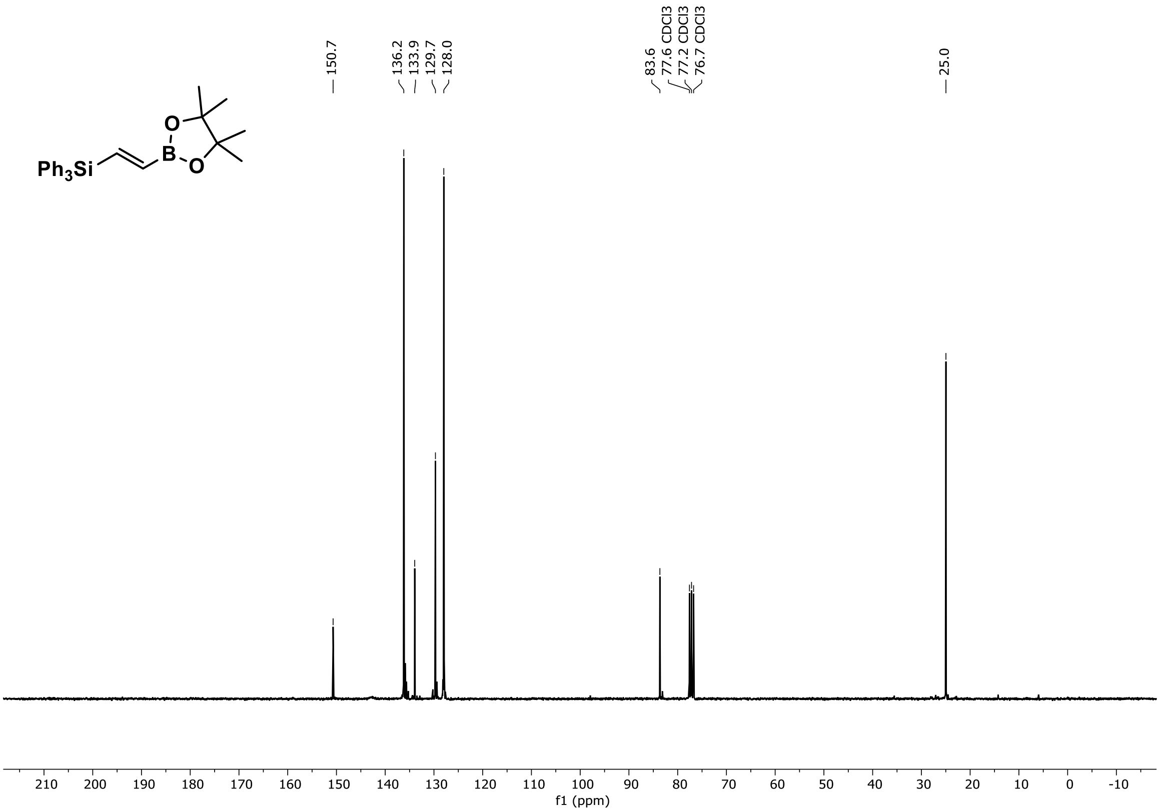

Figure S82. ^{13}C NMR spectrum of *(E)-triphenyl(2-(4,4,5,5-tetramethyl-1,3,2-dioxaborolan-2-yl)vinyl)silane* (**8m**).

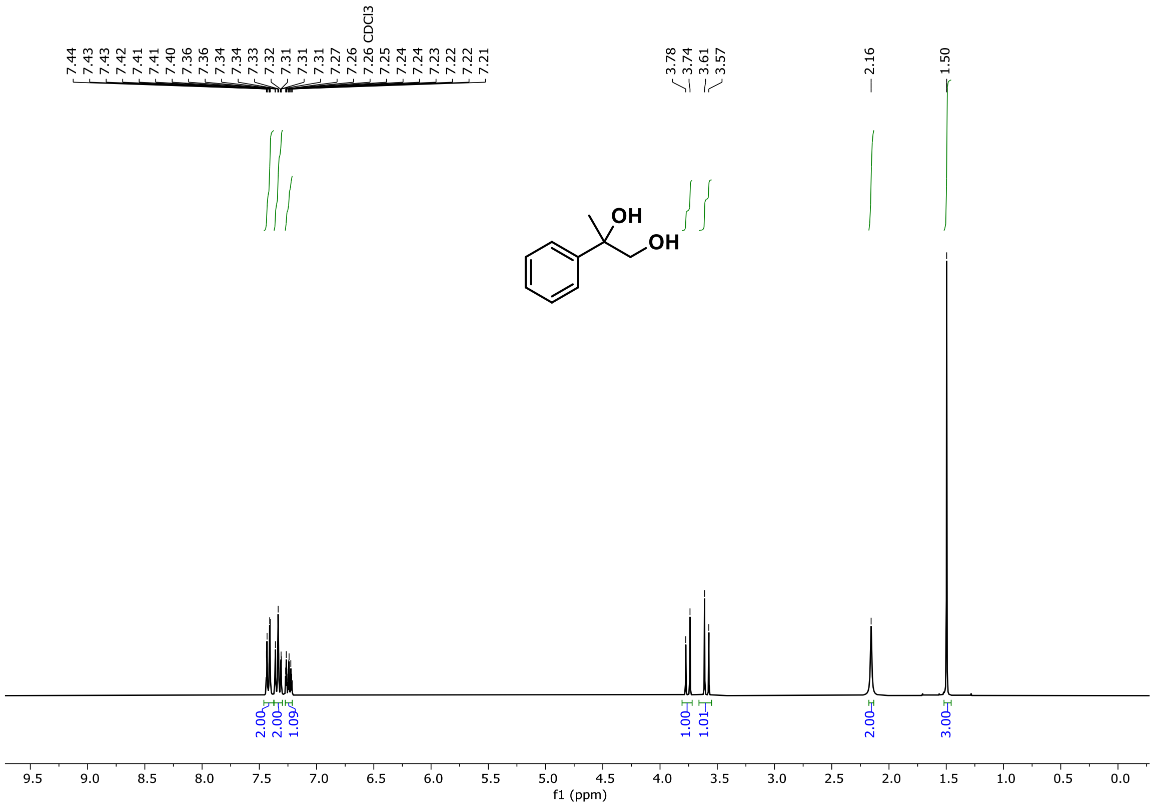

Figure S83. ^{1}H NMR spectrum of *2-phenylpropane-1,2-diol* (**2ja**).

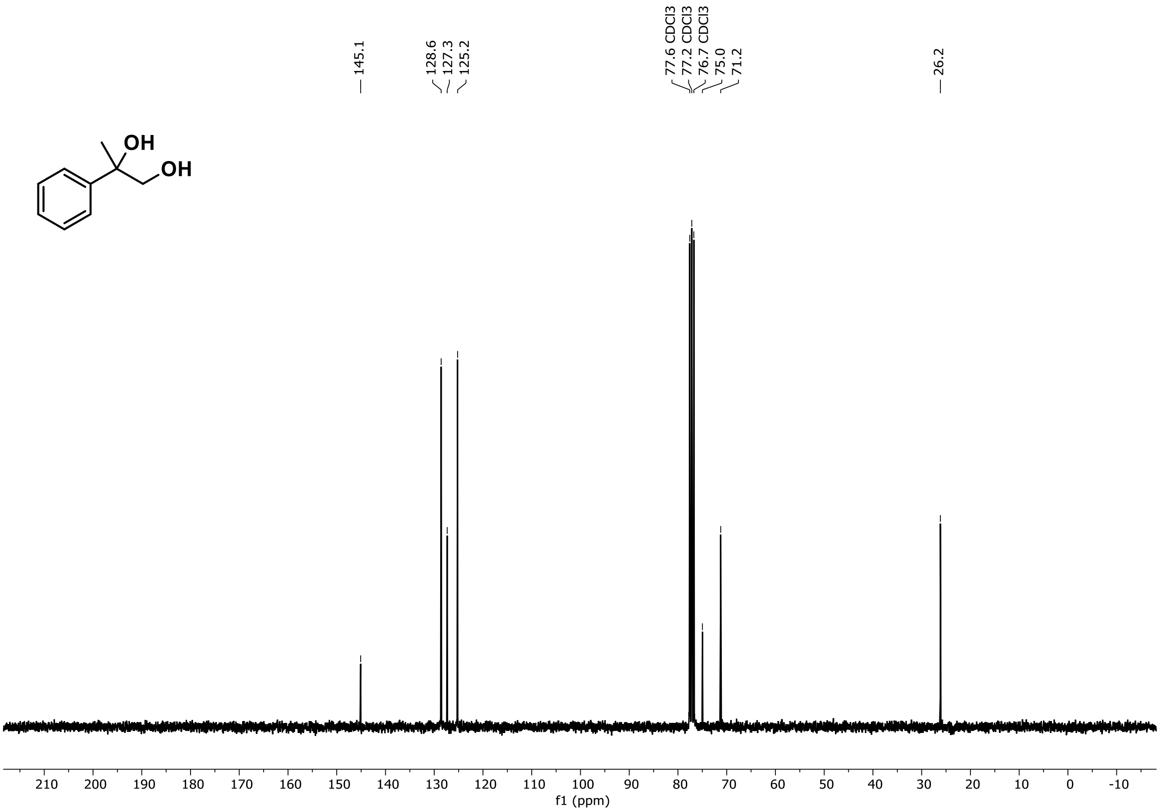

Figure S84. ^{13}C NMR spectrum of *2-phenylpropane-1,2-diol* (**2ja**).

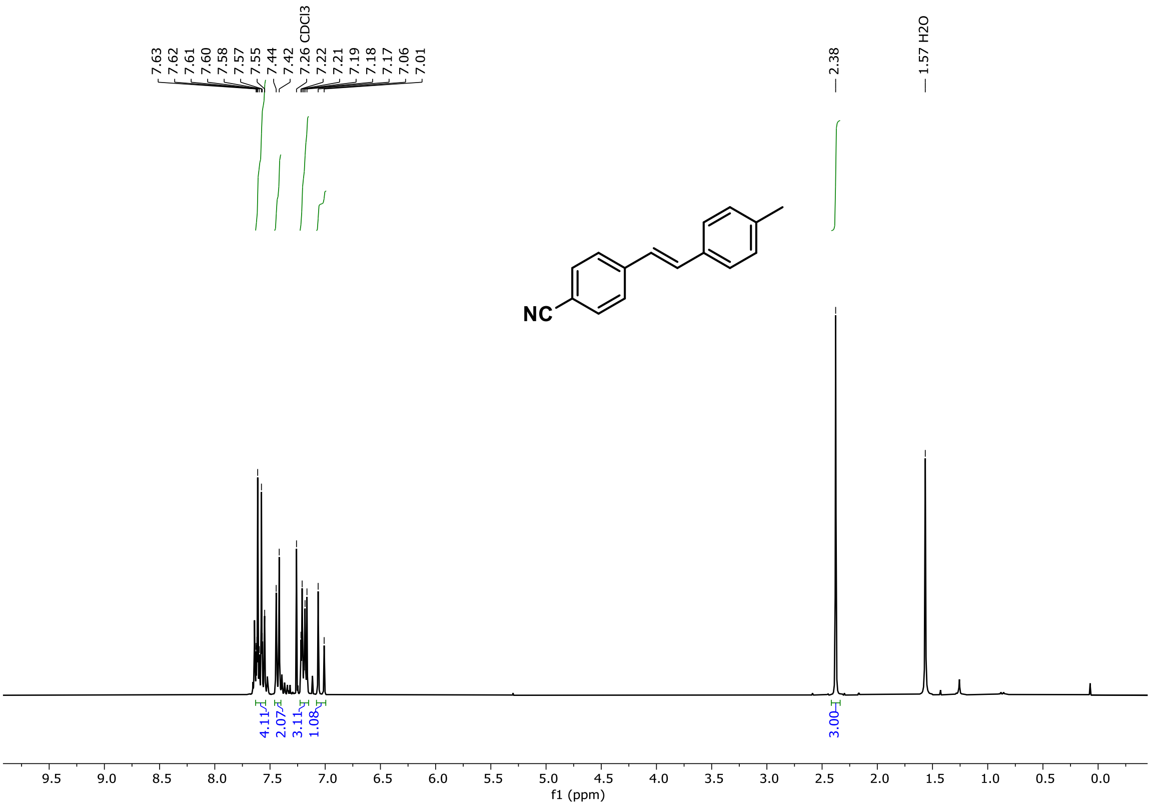

Figure S85. ^{1}H NMR spectrum of *(E)-4-(4-methylstyryl)benzonitrile* (**6ia**).

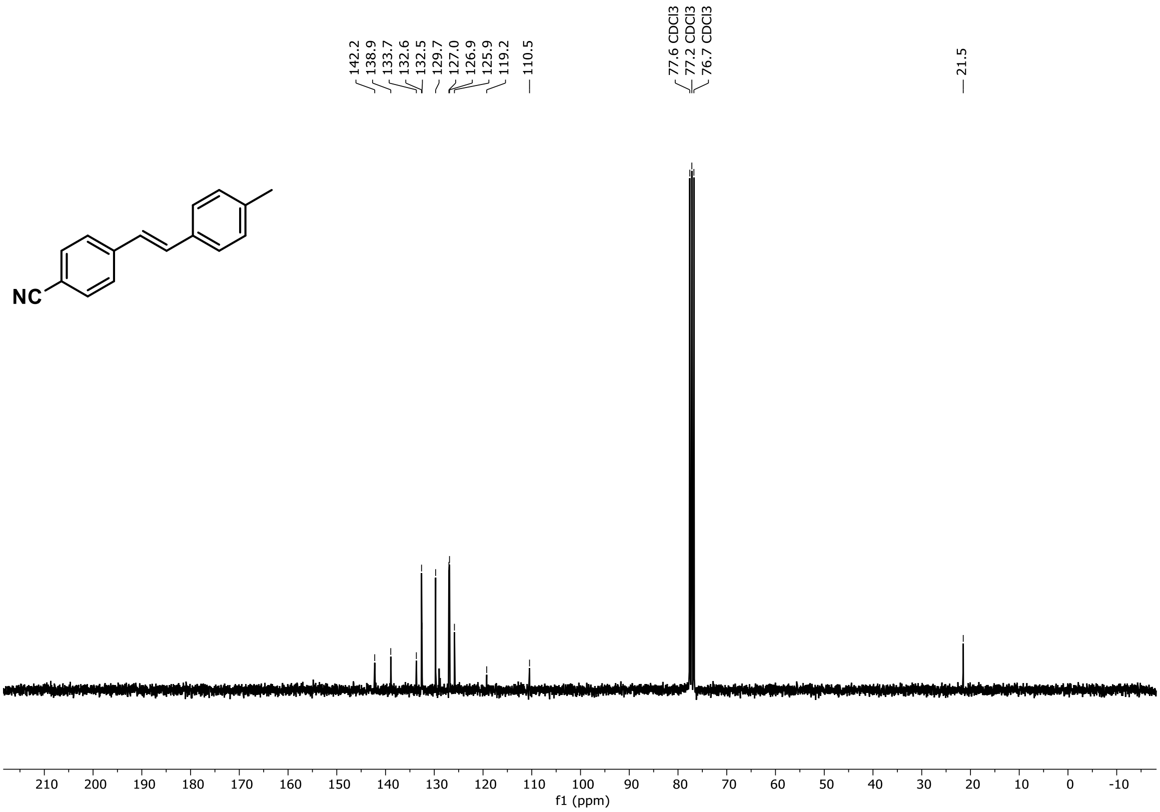

Figure S86. ^{13}C NMR spectrum of *(E)-4-(4-methylstyryl)benzonitrile* (**6ia**).

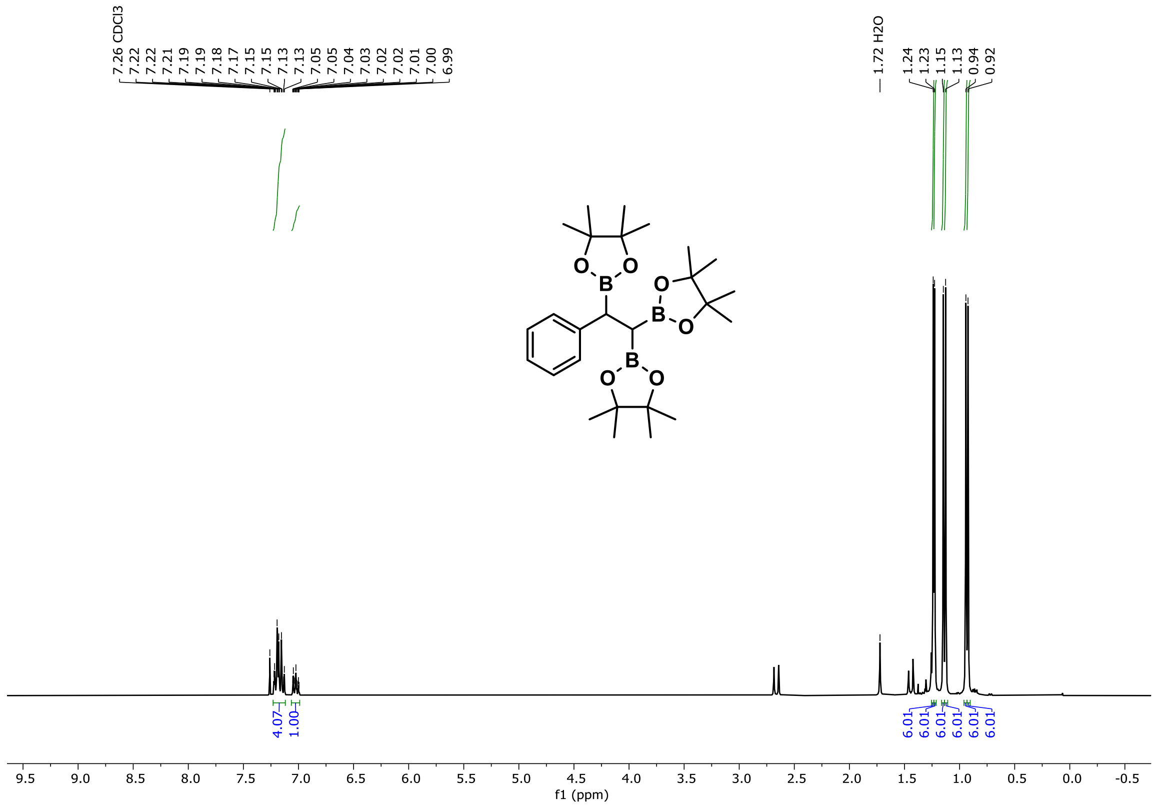

Figure S87. ^{1}H NMR spectrum of *2,2',2''-(2-phenylethane-1,1,2-triyl)tris(4,4,5,5-tetramethyl-1,3,2-dioxaborolane)* (**7a**).

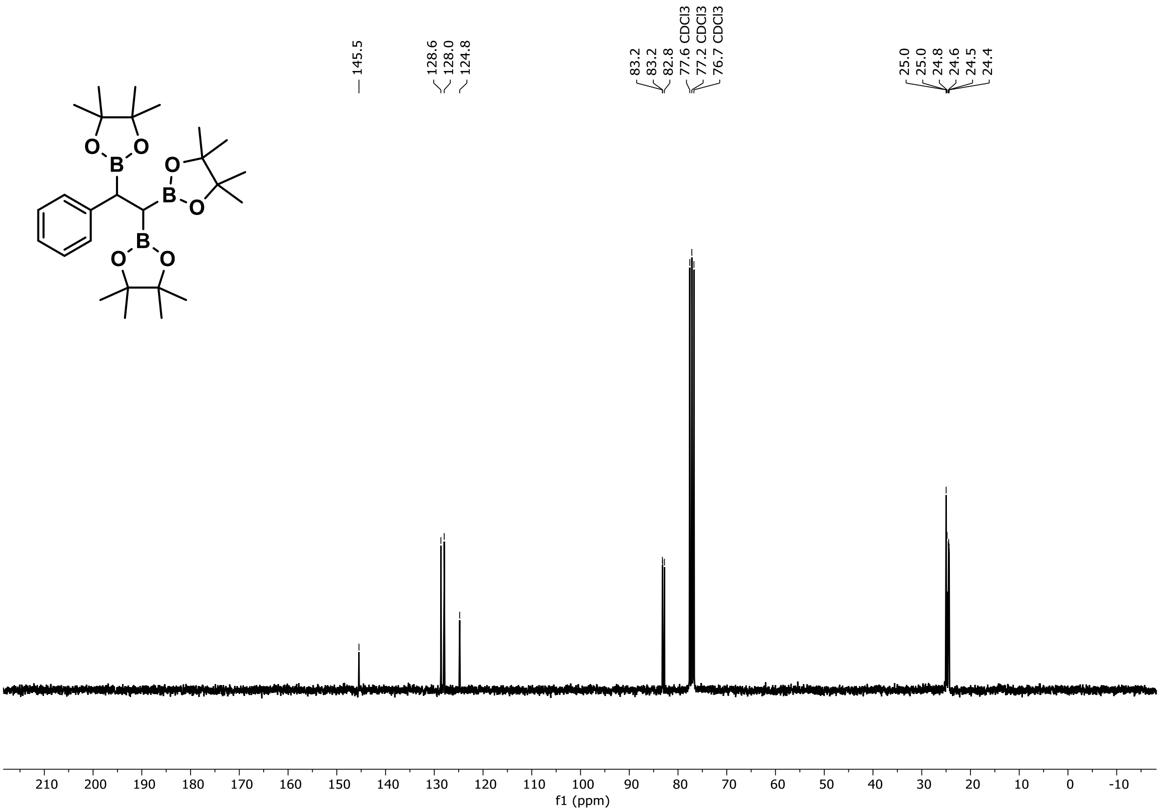

Figure S88. ^{13}C NMR spectrum of *2,2',2''-(2-phenylethane-1,1,2-triyl)tris(4,4,5,5-tetramethyl-1,3,2-dioxaborolane)* (**7a**).

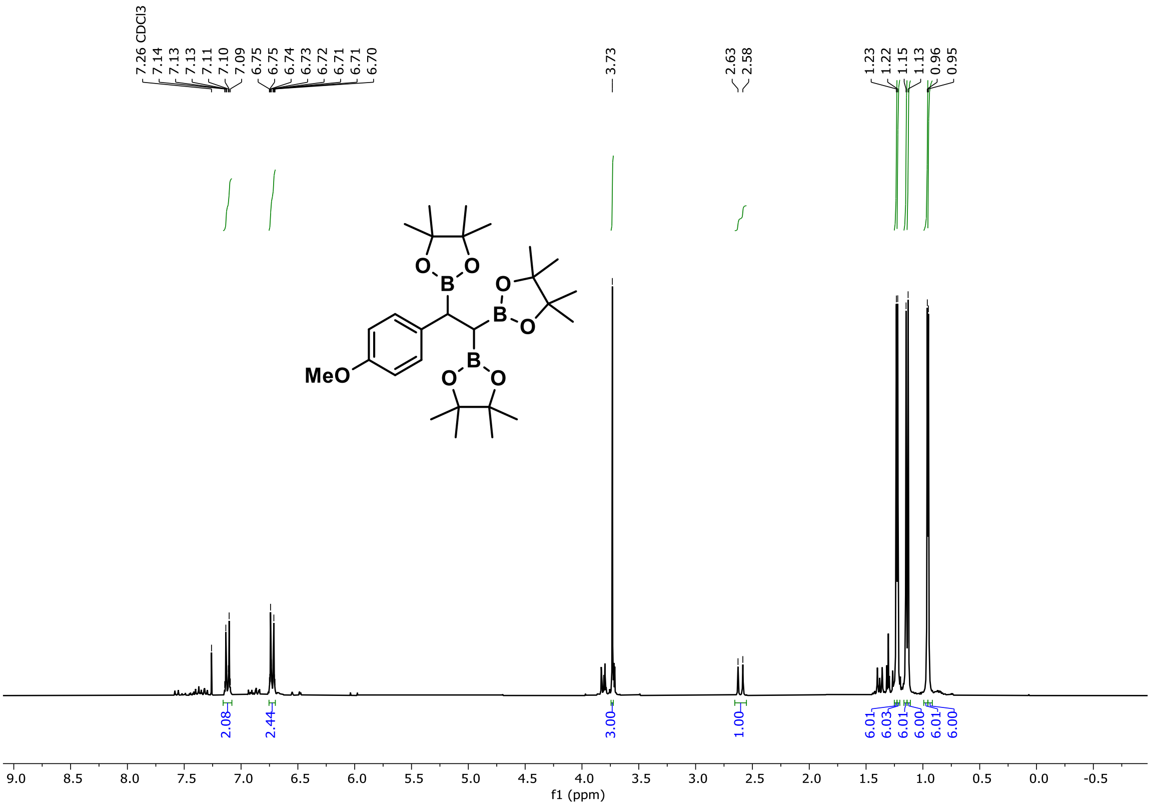

Figure S89. ^{1}H NMR spectrum of *2,2',2''-(2-(4-methoxyphenyl)ethane-1,1,2-triyl)tris(4,4,5,5-tetramethyl-1,3,2-dioxaborolane)* (**7b**).

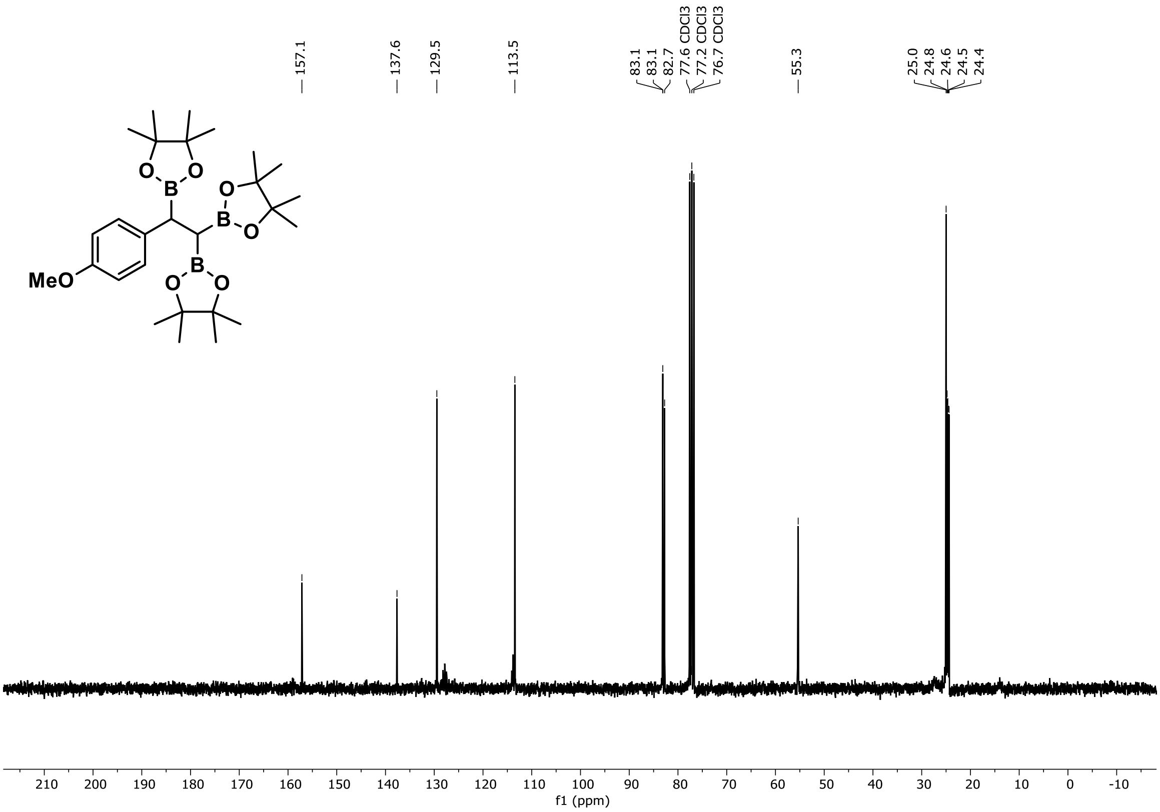

Figure S90. ^{13}C NMR spectrum of *2,2',2''-(2-(4-methoxyphenyl)ethane-1,1,2-triyl)tris(4,4,5,5-tetramethyl-1,3,2-dioxaborolane)* (**7b**).

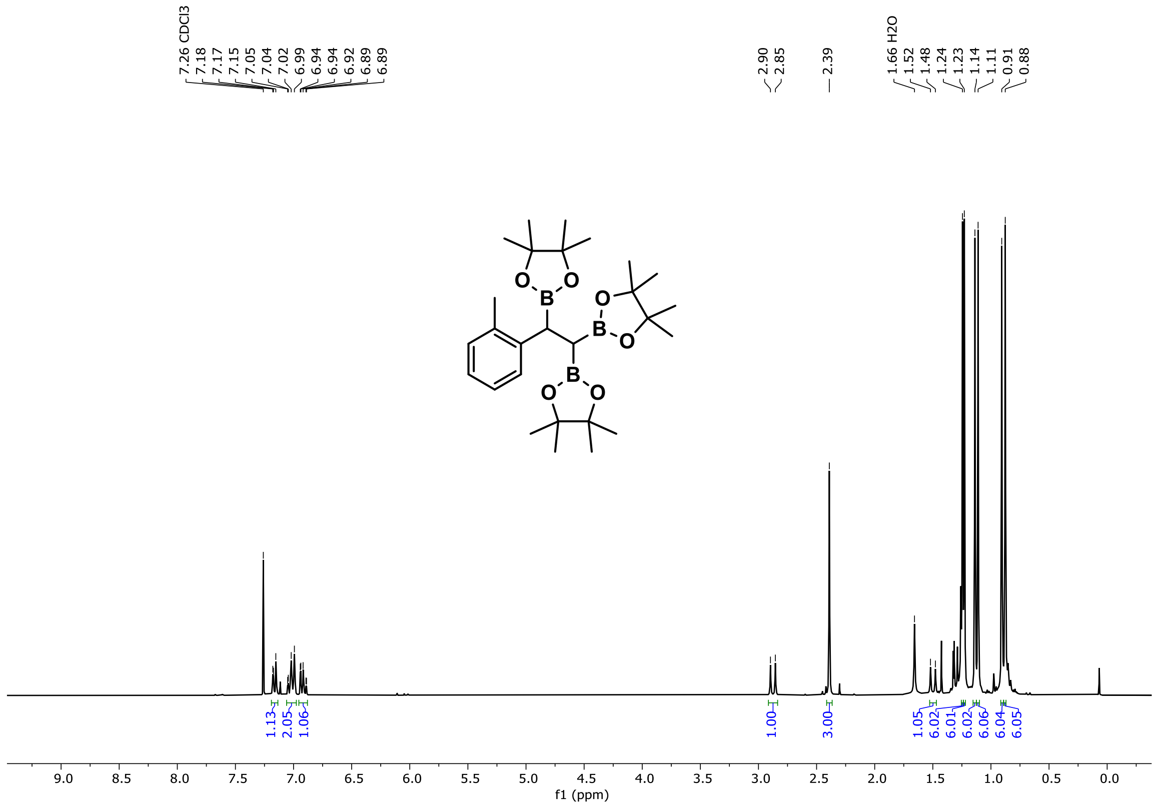

Figure S91. ^{1}H NMR spectrum of *2,2',2''-(2-(o-tolyl)ethane-1,1,2-triyl)tris(4,4,5,5-tetramethyl-1,3,2-dioxaborolane)* (**7c**).

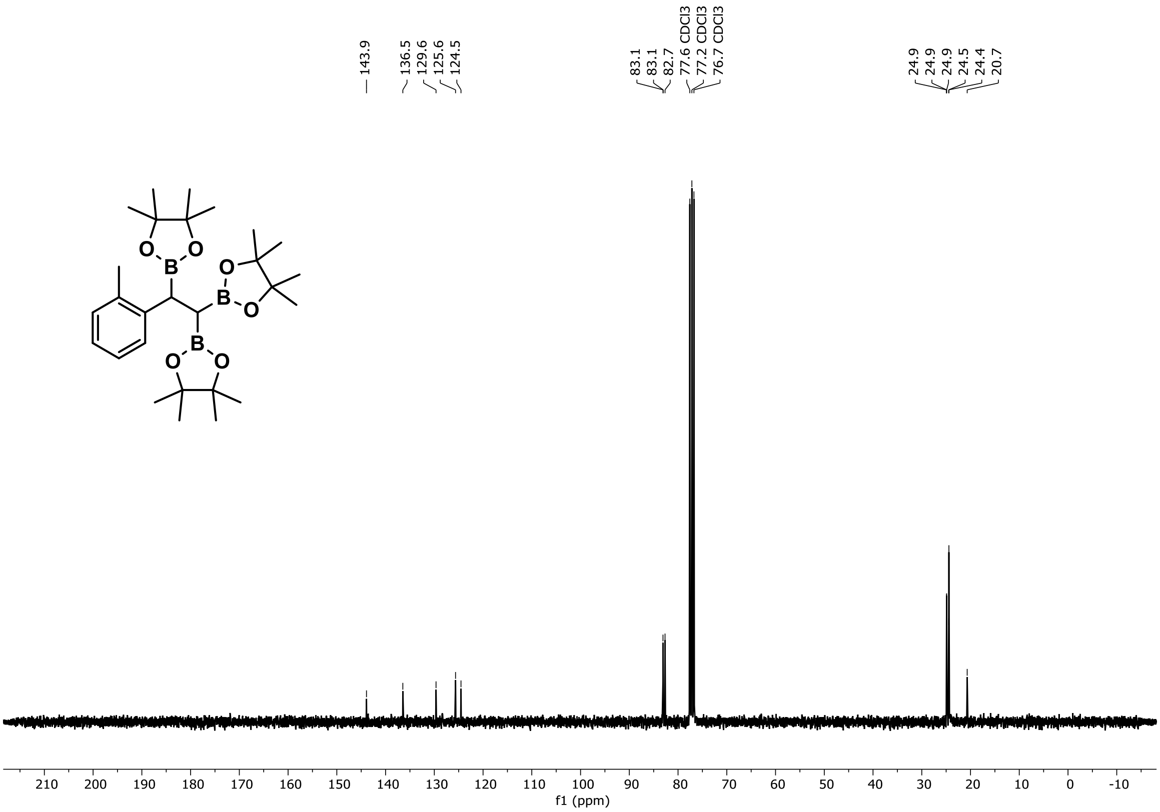

Figure S92. ^{13}C NMR spectrum of *2,2',2''-(2-(o-tolyl)ethane-1,1,2-triyl)tris(4,4,5,5-tetramethyl-1,3,2-dioxaborolane)* (**7c**).

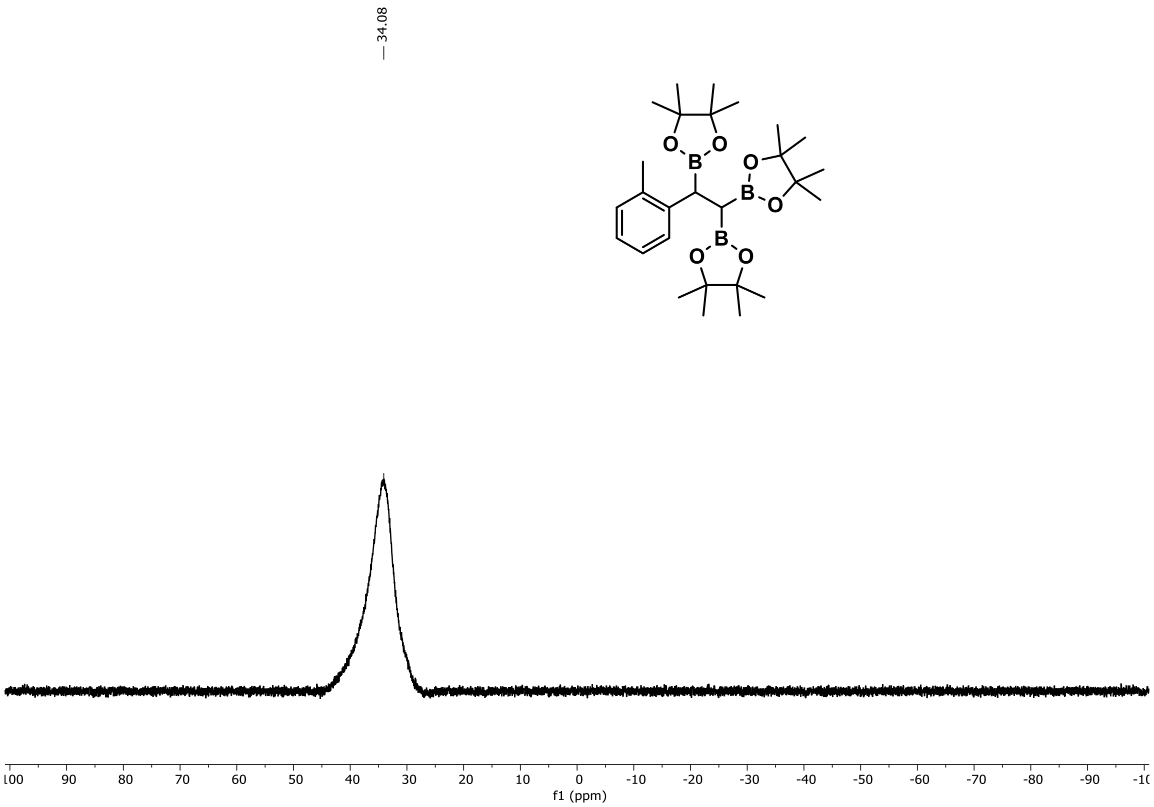

Figure S93. ^{11}B NMR spectrum of *2,2',2''-(2-(o-tolyl)ethane-1,1,2-triyl)tris(4,4,5,5-tetramethyl-1,3,2-dioxaborolane)* (**7c**).

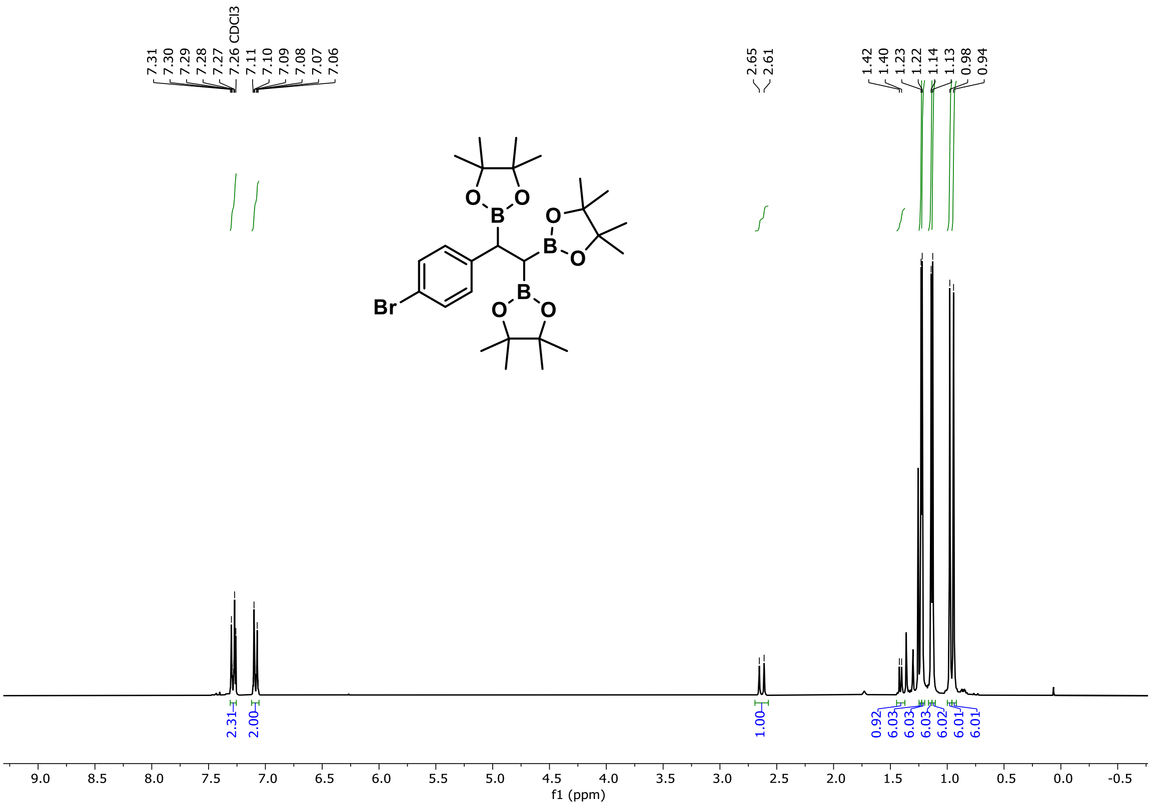

Figure S94. ^{1}H NMR spectrum of *2,2',2''-(2-(4-bromophenyl)ethane-1,1,2-triyl)tris(4,4,5,5-tetramethyl-1,3,2-dioxaborolane)* (**7d**).

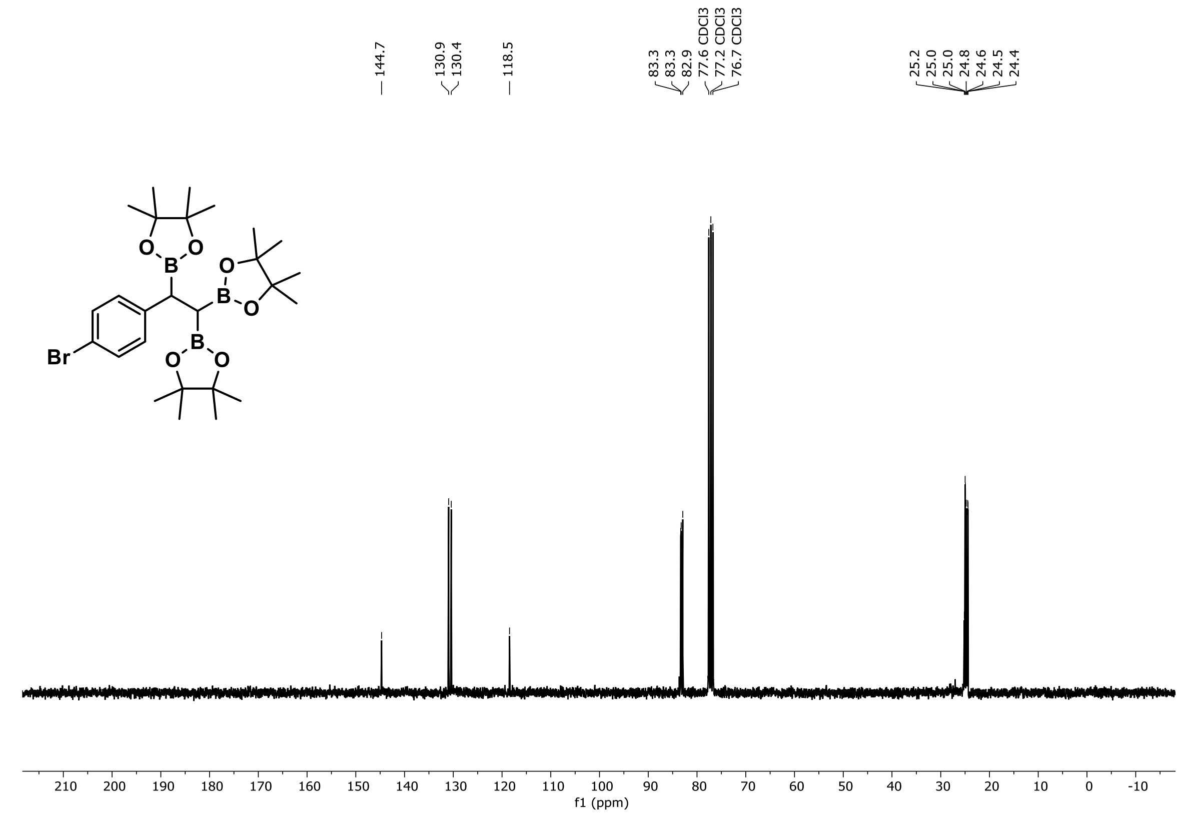

Figure S95. ^{13}C NMR spectrum of *2,2',2''-(2-(4-bromophenyl)ethane-1,1,2-triyl)tris(4,4,5,5-tetramethyl-1,3,2-dioxaborolane)* (**7d**).

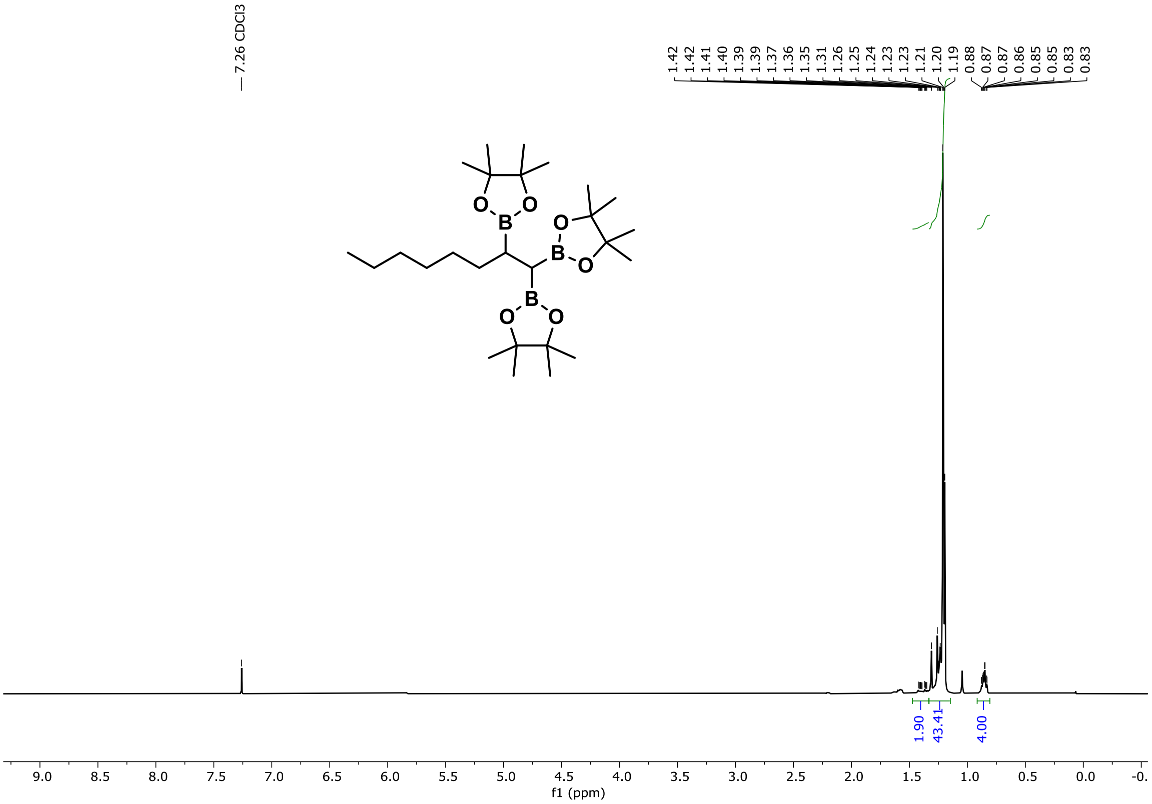

Figure S96. ^{1}H NMR spectrum of *2,2',2''-(octane-1,1,2-triyl)tris(4,4,5,5-tetramethyl-1,3,2-dioxaborolane)* (**7e**).

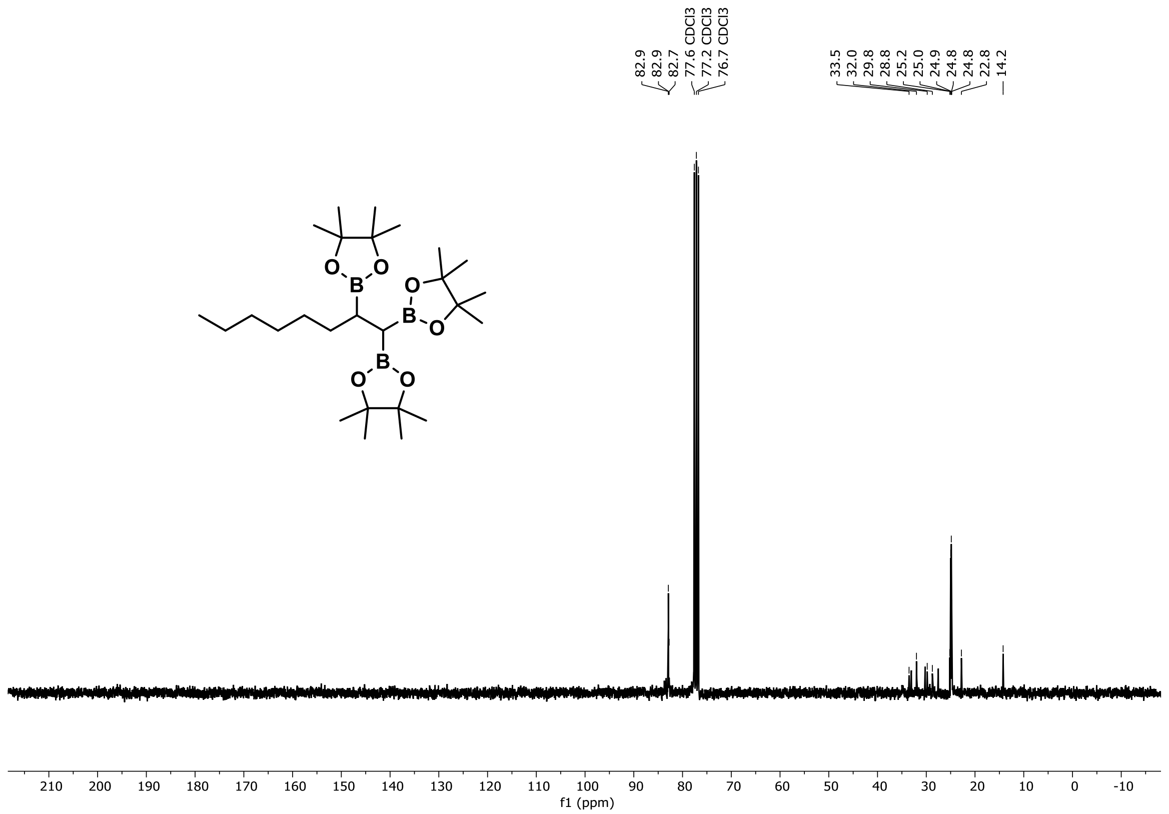

Figure S97. ^{13}C NMR spectrum of *2,2',2''-(octane-1,1,2-triyl)tris(4,4,5,5-tetramethyl-1,3,2-dioxaborolane)* (**7e**).

Table S18. Comparison of selective alkene diboration reaction performance with selected reported catalytic systems.

Catalyst	Reaction Conditions	Yield / Selectivity (%)	Time	Type of Boration	Reference
**Pt-SA-C_3N_4 (*First Pt Single Atom System*)**	50 °C, MeOH	99 / 99	15 h	Alkene, 1,2	Current work
**Pt_1/Ni(OH)x catalyst**	120 °C, Mesitylene	99 / 93	0.3 h	Alkyne, Alkene, 1,2	*26*
**PtSA over Fe_2O_3@rGO**	100 °C, Toluene	98 / 88	2 h	Alkyne, 1,2	*27*
**NaOAc/Au complex**	RT, THF	90 / 99	2 h	Alkene, 1,2	*28*
**Pt_1-PMo@MIL-101**	100 °C, Toluene	90 / 99	3 h	Alkene, 1,2	*29*
**NHC-Cu complex**	-15 °C, THF	98 / 93	48 h	Alkyne, 1,2	*30*
**1% $Pd(OAc)_2$, 1% RuPhos, PhBr**	70 °C, H_2O, THF	92 / 97	1 h	Alkene, 1,2	*31*
**$Ni(COD)_2$, Cy-XantPhos**	130 °C, PhMe/THF	78 / 93	1 h	Alkene, 1,1	*32*
**B_2pin_2, [Co] Cat.**	25 °C, Pentane	94 / 54	1 h	Alkene, Triboration (1,1,1)	*33*
**HBpin, CuOAc, Ligand, NaOtBu**	RT, THF	99 / 90	16 h	Ring opening, Diboration (1,3 or 1,4)	*34*
**$Ni(COD)_2$, 1 mol % PCy_3**	60 °C, Toluene	84 / 87	12 h	1,4-Diboration	*35*
**$Pt_2(dba)_3$, Ligand**	60 °C, Toluene	87 / 88	12 h	1,2-Diboration	*36*
**$[Cu(NHC)_2(NCMe)]BF_4$**	Reflux, THF	99 / 99	4 h	1,2-Diboration	37

7. References

[1] Chai, J.-D.; Head-Gordon,"Long-range corrected hybrid density functionals with damped atom–atom dispersion corrections" *Phys. Chem. Chem. Phys.* **2008**, *10*, 6615-6620.

[2] Weigend, F.; Ahlrichs, R. "Balanced basis sets of split valence, triple zeta valence and quadruple zeta valence quality for H to Rn: Design and assessment of accuracy" *Phys. Chem. Chem. Phys.* **2005**, *7*, 3297-3305.

[3] Frisch, M. J.; Trucks, G. W.; Schlegel, H. B.; Scuseria, G. E.; Robb, M. A.; Cheeseman, J. R.; et al. Gaussian 16, Revision C.01; Gaussian, Inc.: Wallingford CT, **2016**.

[4] Ditchfield, R.; Hehre, W. J.; Pople, J. A. "Self-Consistent Molecular-Orbital Methods. IX. An Extended Gaussian-Type Basis for Molecular-Orbital Studies of Organic Molecules" *J. Chem. Phys.* **1971**, *54*, 724-728.

[5] Reed, A. E.; Curtiss, L. A.; Weinhold, F. "Intermolecular interactions from a natural bond orbital, donor-acceptor viewpoint" *Chem. Rev.* **1988**, *88*, 899-926.

[6] Wiberg, K. B. "Application of the pople-santry-segal CNDO method to the cyclopropylcarbinyl and cyclobutyl cation and to bicyclobutane" *Tetrahedron* **1968**, *24*, 1083-1096.

[7] Yin, C.; Luo, L.; Zhang, H. Zhang,"Iodine-Catalyzed Borylation of Benzylic Alcohols" *Org. Lett.* **2023**, *25*, 1701-1705.

[8] Rzhevskiy, S. A.; Topchiy, M. A.; Lyssenko, K. A.; et al. "New expanded-ring NHC platinum(0) complexes: Synthesis, structure and highly efficient diboration of terminal alkenes" *J. Organomet. Chem.* **2020**, *912*, 121140.

[9] Huang, M.; Hu, J.; Shi, S.; Friedrich, A.; Krebs, J.; Westcott, S. A.; Radius, U.; Marder, T. B."Selective, Transition Metal-free 1,2-Diboration of Alkyl Halides, Tosylates, and Alcohols" *Chem. – Eur. J.* **2022**, *28*, e202200480.

[10] Takahashi, F.; Nogi, K.; Sasamori, T.; Yorimitsu, H."Diborative Reduction of Alkynes to 1,2-Diboryl-1,2-Dimetalloalkanes: Its Application for the Synthesis of Diverse 1,2-Bis(boronate)s" *Org. Lett.* **2019**, *21*, 4739-4744.

[11] Willems, S.; Toupalas, G.; Reisenbauer, J. C.; Morandi, B. "A site-selective and stereospecific cascade Suzuki–Miyaura annulation of alkyl 1,2-bisboronic esters and 2,2′-dihalo 1,1′-biaryls" *Chem. Commun.* **2021**, *57*, 3909-3912.

[12] Yuan, Q.; Zhang, X.; Guo, L.; Yang, C.; Xia, W."Metal-Free Electrochemical Hydroboration of Olefins" *Adv. Synth. Catal.* **2023**, *365*, 1788-1793.

[13] Bhawar, R.; Saini, S.; Nagaraju, D. H.; Bose, S. K."CeO2-Nanorods-Catalyzed Protoboration of Alkenes and Alkynes with Bis(pinacolato)diboron" *Adv. Synth. Catal.* **2023**, *365*, 584-593.

[14] Fasano, V.; Winter, N.; Noble, A.; Aggarwal, V. K. "Divergent, Stereospecific Mono- and Difluoromethylation of Boronic Esters" *Angew. Chem., Int. Ed.* **2020**, *59*, 8502-8506.

[15] Alamer, B.; Sagadevan, A.; Bodiuzzaman, M.; Murugesan, K.; Alsharif, S.; Huang, R.-W.; Ghosh, A.; Naveen, M. H.; Dong, C.; Nematulloev, S.; Yin, J.; Shkurenko, A.; Abulikemu, M.; Dong, X.; Han, Y.; Eddaoudi, M.; Rueping, M.; Bakr, O. M. " *J. Am. Chem. Soc.* **2024**, *146*, 16295-16305.

[16] Xu, J.; Lan, Y.; Liu, B."Activation of Aryl and Alkyl Halides Enabled by Strong Photoreduction Potentials of a Hantzsch Ester/Cs2CO3 System" *J. Org. Chem.* **2024**, *89*, 599-604.

[17] Kondo, H.; Miyamura, S.; Matsushita, K.; Kato, H.; Kobayashi, C.; Arifin; Itami, K.; Yokogawa, D.; Yamaguchi, J."σ-Bond Hydroboration of Cyclopropanes" *J. Am. Chem. Soc.* **2020**, *142*, 11306-11313.

[18] Aelterman, M.; Sayes, M.; Jubault, P.; Poisson, T."Electrochemical Hydroboration of Alkynes" *Chem. – Eur. J.* **2021**, *27*, 8277-8282.

[19] Ton, N. N. H.; Mai, B. K.; Nguyen, T. V. "Tropylium-Promoted Hydroboration Reactions: Mechanistic Insights Via Experimental and Computational Studies" *J. Org. Chem.* **2021**, *86*, 9117-9133.

[20] Stefanowska, K.; Sokolnicki, T.; Walkowiak, J.; Czapik, A.; Franczyk, A."Directed cis-hydrosilylation of borylalkynes to borylsilylalkenes" *Chem. Commun.* **2022**, *58*, 12046-12049.

[21] Hampton, C.; Simonetti, M.; Leonori, D."Olefin Dihydroxylation Using Nitroarenes as Photoresponsive Oxidants" *Angew. Chem., Int. Ed.* **2023**, *62*, e202214508.

[22] Murakami, S.; Matsubara, R.; Hayashi, M. "Diboration of Alkynes Accelerated by Synergistic Effects of Ruthenium Complexes and Phenoxides" *Eur. J. Org. Chem.* **2022**, e202200905.

[23] Kita, Y.; Tobisu, M.; Chatani, N."Rhodium-Catalyzed Alkenylation of Nitriles via Silicon-Assisted C−CN Bond Cleavage" *Org. Lett.* **2010**, *12*, 1864-1867.

[24] Zhang, J., Wu, X.; Cheong, W.-C., Chen, W., Lin, R., Li, J., Zheng, L., Yan, W., Gu, L., Chen, C., Peng, Q., Wang, D., Li, Y. Cation Vacancy Stabilization of Single-Atomic-Site $Pt_1/Ni(OH)_x$ Catalyst for Diboration of Alkynes and Alkenes. *Nat. Commun.* **2018**, *9*, 1002.

[25] Miao, X., Chen, W., Lv, S., Li, A., Li, Y., Zhang, Q., Yue, Y., Zhao, H., Liu, L., Guo, S., Guo, L. Stabilizing Single-Atomic Pt by Forming Pt□Fe Bonds for Efficient Diboration of Alkynes. *Adv. Mater.* **2023**, *35*, 2211790.

[26] Ramírez, J., Sanaú, M., Fernández, E. Gold(0) Nanoparticles for Selective Catalytic Diboration. *Angew. Chem., Int. Ed.* **2008**, *47*, 5194–5197.

[27] Liu, Y., Wu, X., Li, Z., Zhang, J., Liu, S.-X., Liu, S., Gu, L., Zheng, L. R., Li, J., Wang, D., Li, Y. Fabricating Polyoxometalates-Stabilized Single-Atom Site Catalysts in Confined Space with Enhanced Activity for Alkynes Diboration. *Nat. Commun.* **2021**, *12*, 4205.

[28] Lee, Y., Jang, H., Hoveyda, A. H. Vicinal Diboronates in High Enantiomeric Purity through Tandem Site-Selective NHC−Cu-Catalyzed Boron−Copper Additions to Terminal Alkynes. *J. Am. Chem. Soc.* **2009**, *131*, 18234–18235

[29] Mlynarski, S. N., Schuster, C. H., Morken, J. P. Asymmetric Synthesis from Terminal Alkenes by Cascades of Diboration and Cross-Coupling. *Nature* **2014**, *505*, 386–390.

[30] Li, L., Gong, T., Lu, X., Xiao, B., Fu, Y. Nickel-Catalyzed Synthesis of 1,1-Diborylalkanes from Terminal Alkenes. *Nat. Commun.* **2017**, *8*, 345.

[31] Zhang, L., Huang, Z. Synthesis of 1,1,1-Tris(boronates) from Vinylarenes by Co-Catalyzed Dehydrogenative Borylations–Hydroboration. *J. Am. Chem. Soc.* **2015**, *137*, 15600–15603.

[34] Liang, H., Morken, J. P**.** Stereospecific Transformations of Alkylboronic Esters Enabled by Direct Boron-to-Zinc Transmetalation. *J. Am. Chem. Soc.* **2024**, *146*, 5366–5374.

[35] Ely, R. J., Morken, J. P. Ni(0)-Catalyzed 1,4-Selective Diboration of Conjugated Dienes. *Org. Lett.* **2010**, *12*, 4348–4351.

[36] Kliman, L. T., Mlynarski, S. N., Morken, J. P. Pt-Catalyzed Enantioselective Diboration of Terminal Alkenes with $B_2(pin)_2$. *J. Am. Chem. Soc.* **2009**, *131*, 13210–13211.

[37] Lillo, V., Fructos, M. R., Ramírez, J., Braga, A. A. C., Maseras, F., Díaz-Requejo, M. M., Pérez, P. J., Fernández, E. A Valuable, Inexpensive CuI/N-Heterocyclic Carbene Catalyst for the Selective Diboration of Styrene. *Chem. Eur. J.* **2007**, *13*, 2614–2621.

[38] Coombs, R. J., Zhang, L., Morken, J. P. Enantiomerically Enriched Tris(boronates): Readily Accessible Conjunctive Reagents for Asymmetric Synthesis. *J. Am. Chem. Soc.* **2014**, *136*, 46, 16140–16143.

[39] Patil, K. S., Reddappa, S., Kumar, R., Mane, M. V., Bose. S. K. Synthesis of 1,2-Bis- and 1,1,2-Tris-Borylalkanes under Transition Metal-Free and Solvent-Free Conditions. *J. Org. Chem.* **2025**, 90, 12, 4140–4148.

[40] Endo, K., Sakamoto A., Ohkubo, T., Shibata, T. Stereoselective Synthesis of Allylsilanes Bearing Tetrasubstituted Olefin via 2,2-Diborylethylsilane. *Chem. Lett.* **2011**, 40, 12, 1440–1442.
